# Transaminase‐Triggered Cascades for the Synthesis and Dynamic Kinetic Resolution of Chiral *N*‐Heterocycles

**DOI:** 10.1002/anie.202422584

**Published:** 2025-04-18

**Authors:** Adam O'Connell, Marianne B. Haarr, James Ryan, Xingxing Xu, Aoife Martin, Simon N. Smith, Nadia Elghobashi‐Meinhardt, Patricia Fleming, Beatriz Maciá, Vittorio Caprio, Elaine O'Reilly

**Affiliations:** ^1^ School of Chemistry University College Dublin Belfield Dublin 4 Ireland; ^2^ Faculty of Science & Engineering Division of Chemistry & Environmental Science Manchester Metropolitan University Chester Street Manchester M1 5GD UK

**Keywords:** Asymmetric synthesis, Aza‐Michael reaction, Biocatalysis, Heterocycles, Transaminase

## Abstract

Biocatalysis is now a well‐established branch of catalysis and the growing toolbox of natural, evolved and designer enzymes is enabling chemistry previously deemed inaccessible. However, most enzyme methodologies have been developed for functional group interconversions, such as the conversion of a ketone into an amine or alcohol, and do not result in the generation of significant 3D molecular complexity. The application of enzyme‐triggered reaction cascade methodologies has the potential to transform simple substrates into complex sp^3^‐rich molecules in one step. Herein, we describe a single‐step biocatalytic route to high‐value, complex indolizidine, and quinolizidine alkaloids, which relies on a transaminase‐triggered double intramolecular aza‐Michael reaction. This approach allows access to architecturally complex, natural‐product‐like *N*‐heterocycles and reveals intriguing examples of diastereoselectivity in these enzyme‐triggered reactions. Significantly, we demonstrate an elegant example of a biocatalytic cascade where the transaminase plays a dual role in generating complex *N*‐heterocycles and where a retro‐double intramolecular aza‐Michael reaction mediates a dynamic kinetic resolution and enables the isolation of sp^3^‐rich indolizidine diastereoisomers containing five stereocenters, as single isomers.

## Graphical abstract

A single‐step biocatalytic route to complex indolizidine and quinolizidine alkaloids is described that relies on transaminase‐triggered double intramolecular aza‐Michael methodology. In one case, a retro‐double intramolecular aza‐Michael reaction enables dynamic kinetic resolution.

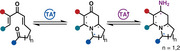

In recent years, biocatalysis has made possible a wealth of novel chemistry not previously accessible, thanks to the unrivalled regio‐, chemo‐ and stereoselectivity associated with enzymes.^[^ ^1^, ^2^ ^]^ Approximately 40% of the top 200 small molecule drugs by retail sales in 2022 contain one or more chiral amine moieties in their structures,^[^ ^3^ ^]^ yet traditional approaches for synthesising these amines often rely on precious metal catalysts, employed at elevated temperatures and in environmentally harmful solvents.^[^ ^4^, ^5^ ^]^ A growing demand for more sustainable synthetic methodology has spearheaded the discovery and development of enzymes for numerous transformations, not least for the asymmetric synthesis of optically pure amines.^[^ ^6^, ^7^ ^]^

Substituted *N*‐heterocycles are important building blocks for many natural products, pharmaceuticals, and other industrially relevant chemicals. When synthesising *N*‐heterocyclic compounds, installation of the (chiral) amine moiety is often the most straightforward synthetic approach, and given the importance of these nitrogen‐containing molecules, there have been several enzymes discovered and developed for their asymmetric preparation.^[^ ^8^, ^9^, ^10^, ^11^, ^12^, ^13^, ^14^, ^15^, ^16^ ^]^ A prominent class of enzymes for amine synthesis are the ω‐transaminases (TAs), which rely on a pyridoxyl‐5‐phosphate coenzyme to mediate the reversible transfer of an amino group from a sacrificial amine donor to an amine acceptor. Although TAs, like many enzymes, are traditionally employed for their ability to mediate functional group interconversions (FGIs) with unrivalled selectivity, some biocatalytic methodologies have been developed that extend beyond single FGIs to encompass innovative and complexity‐generating methodologies, such as multi‐step chemoenzymatic cascades and the application of enzymes to trigger subsequent cascade reactions.^[^ ^17^ ^]^

Among the *N*‐heterocycles, quinolizidine,‐ and indolizidine‐containing alkaloids represent a class of azabicyclic compounds that have gained significant interest due to their low natural abundance and interesting biological activities (Scheme 1a).^[^ ^18^, ^19^ ^]^ However, many total syntheses targeting these alkaloid scaffolds involve step‐by‐step syntheses, where chirality is either introduced into the molecule in an asymmetric reaction, or assembled from chiral building blocks, with the potential for scrambling of the installed stereocentre requiring constant monitoring.^[^ ^20^ ^]^ One synthetic strategy that has found widespread application for the preparation of heterocycles bearing nitrogen‐substituted stereocentres is the intramolecular aza‐Michael reaction (IMAMR).^[^ ^21^ ^]^ The reaction relies on the conjugate addition of nitrogen nucleophiles, and due to the spontaneous nature of the reaction, obtaining a stable starting material, where both the nitrogen nucleophile and electrophile coexist in an unreactive substrate so as not to compromise selectivity, has been a major obstacle. Although the double intramolecular aza‐Michael reaction (DIMAMR) is a powerful approach for accessing quinolizidine alkaloids, it faces the same set of challenges as the single IMAMR equivalent. Strategies typically rely on employing a nucleophile with diminished reactivity, such as sulfinamides and carbamates.^[^ ^22^, ^23^, ^24^ ^]^

**Scheme 1:**
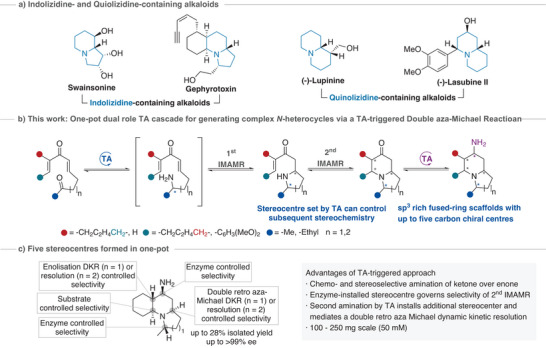
a) Alkaloids containing an indolizidine or quinolizidine moiety. b) One‐pot dual cascade for generating complex *N*‐heterocycles through a TA‐triggered double aza‐Michael reaction. c) Origin of the stereocentres formed using the DIMAMR methodology.

Building upon methodology developed in our group,^[^ ^12^ ^]^ we describe an enzyme‐induced DIMAMR for the formation of quinolizidine‐ and indolizidine‐containing compounds, starting from suitably designed dienone precursors (Scheme 1b). The spontaneous double cyclisation is initiated by the regio‐ and stereoselective installation of a chiral amine nucleophile using a TA, and represents the first example of an enzyme‐triggered DIMAMR. Furthermore, the reversibility of the non‐stereoselective aza‐Michael reaction, combined with the substrate selectivity of a second TA, is used to showcase an enzyme‐triggered DIMAMR coupled to a double retro aza‐Michael dynamic kinetic resolution (DKR) for the asymmetric installation of five chiral centres in a one‐pot cascade (Scheme 1c).

To test the feasibility of using TA‐triggered DIMAMR methodology to access complex heterocycles, a panel of nine novel dienone substrates (**1a‐i**, Figure 1) were synthesised and tested with a selection of commercially available ω‐TA biocatalysts from Codexis, where two enzymes with complementary stereoselectivity were selected. The substrates were prepared from commercially available methyl ketones (see Supporting Information for full details) and were designed to ensure that, following enzymatic amination, the desired reactive intermediate would form, thereby spontaneously inducing a DIMAMR. The substrates were selected to target known natural product scaffolds, with **1e‐f** being designed to obtain precursors for lasubine or subcosine analogues,^[^ ^25^, ^26^ ^]^ and **1a‐d** targeting the analogues of the psychostimulant drug gephyrotoxin.^[^ ^27^, ^28^ ^]^

**Figure 1:**
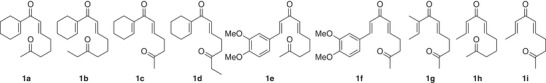
Substrate panel for the enzyme‐triggered double intramolecular aza‐Michael methodology.

Following initial optimisation of the biotransformation conditions using **1a** as the model substrate (Table S1), ketoenones (**1a**‐**i**) were evaluated with (*S*)‐selective ATA256 (Scheme 2a), which for substrates **1a‐f** were aminated at the methyl or ethyl ketone regioselectively.

**Scheme 2:**
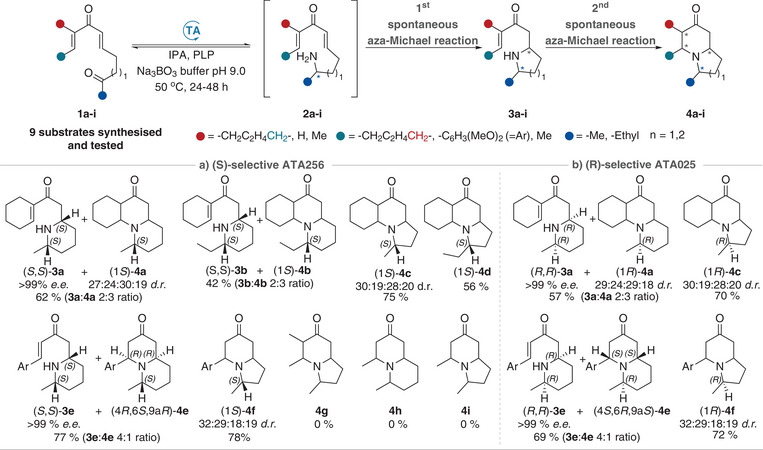
TA‐triggered DIMAMR of the enone substrates **1a**‐**i** to form indolizidine and quinolizidine products **4a**‐**i** with a) (*S*)‐selective ATA256 and b) (*R*)‐selective ATA025.

As previously shown, any amination at the enone carbonyl is shuttled back to the productive aza‐Michael substrate **2a‐f**.^[^ ^12^, ^16^ ^]^ Once formed, these reactive chiral amines **2** undergo an initial IMAMR to give the corresponding 2,5‐disubstituted pyrrolidines **3** (*n* = 1) or 2,6‐disubstituted piperidines **3** (*n* = 2), which then undergo a second IMAMR to form the indolizidine **4** (*n* = 1) or quinolizidine **4** (*n* = 2) products, respectively. Most likely due to substrate instability, the substrates **1g‐i** decomposed under biotransformation conditions and no product (**4g‐i**) formation was observed in these reactions. The biotransformation products presented in Scheme 2 are novel compounds, with no prior literature references available for their structural analyses. All isomers from the mixtures of **4a**, **4c**, **4e**, and **4f** were purified or enriched by flash column chromatography with neutral alumina as the stationary phase in order to characterise the diastereomeric composition of the mixtures by 1D and 2D NMR spectroscopic analysis. Due to the stereochemical complexity of the mixture of tricyclic compounds, the structure elucidation of **4a** and **4c** was further corroborated by proton proximity calculations for all possible stereoisomers (see Supporting Information for full set of calculations). We also showed that the methodology is scalable for preparative synthesis up to 250 mg of product. With ATA256 we could access the ethyl derivatives **4b** and **4d** as a mixture of four diastereoisomers. Peak overlap in the ^1^H NMR spectra did, however, not allow for the accurate calculation of the *d.r*.

From analysis of the biotransformation products, we made three key observations. First, the indolizidines and quinolizidines **4** displayed a significant difference in the second aza‐Michael cyclisation reaction. In the case of pyrrolidines **3** (*n* = 1), the second IMAMR was rapid, resulting in the formation of the indolizidine products **4** (*n* = 1) exclusively. For piperidines **3** (*n* = 2), however, both the quinolizidines **4** (*n* = 2), arising from the second IMAMR, and the intermediate 2,6‐*cis*‐disubstituted piperidines **3** (*n* = 2) were isolated from the biotransformations. The slower cyclisation observed with the piperidines **3** (*n* = 2) compared to the pyrrolidines **3** (*n* = 1) can be rationalised by the difference in nucleophilicity between the pyrrolidine and piperidine nitrogen, and this allowed us to isolate and characterise the “single aza‐Michael” products (i.e., (*S*,*S*)‐**3a**, (*R*,*R*)‐**3a**, (*S*,*S*)‐**3e**, and (*R*,*R*)‐**3e**). Only the *cis*‐2,6‐disubstituted piperidines were observed in the crude reaction mixtures, and we hypothesised that this is due to a retro aza‐Michael epimerisation from the *trans*‐2,6‐disubstituted piperidines to the more thermodynamically stable *cis*‐isomer under biotransformation conditions, and/or because the second aza‐Michael reaction is more favourable for the *trans*‐2,6‐disubstituted piperidine, leaving behind the *cis*‐isomer as one of the reaction products. The latter was evident in the formation of the aromatic quinolizidine **4e**, which arises from the unobserved *trans*‐2,6‐disubstituted piperidine (*S*,*R*)‐**3e** (Scheme 3a). However, an in situ epimerisation was most likely the faster reaction given the significant amount of (*S*,*S)*‐**3e** observed in the crude biotransformation mixture (**3e**:**4e** 4:1, Scheme 2). Retro aza‐Michael epimerisation was also evident when purified (*S*,*S)*‐**3a** was stirred in buffer at 50 °C for 48 h to give tricyclic quinolizidines **4a** arising from both the *cis*‐2,6‐ and the *trans*‐2,6‐disubstituted piperidines (*S*,*S*)‐**3a** and (*S*,*R*)‐**3a** (Scheme 3b).

**Scheme 3:**
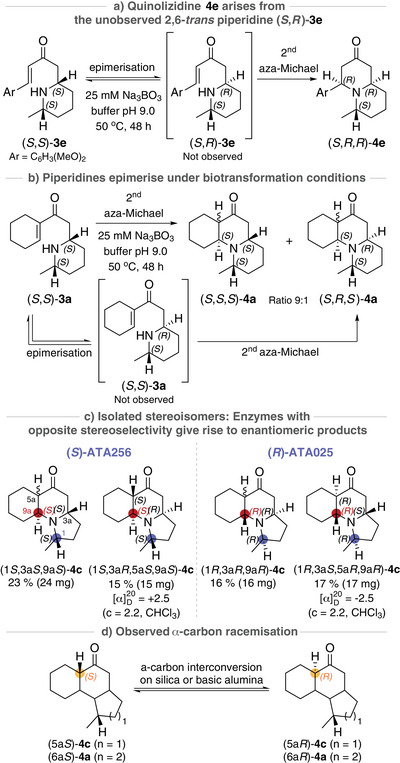
Key observations from the TA‐triggered double intramolecular aza‐Michael methodology.

Secondly, of eight possible stereoisomers that can be formed from the chiral amine intermediates **2a**‐**d**, only four diastereomers **4a‐d** were observed. As seen for quinolizidine **4e**, the stereoselective outcome from the second aza‐Michael reaction also appeared to be substrate controlled for products **4a‐d** (Scheme 2). This was evident from the equivalent stereochemical pattern of purified isomers (1*S*)‐**4a**/**4c** and (1*R*)‐**4a**/**4c**, produced with an (*S*)‐selective and (*R*)‐selective TA, respectively (Scheme 3c). The additional substrate‐controlled selectivity was rationalised by identifying the most favourable transition states of the second aza‐Michael reaction (see Supporting Information for more information).

Thirdly, the tertiary α‐carbon of the tricyclic double aza‐Michael products (**4a**/**4c**) readily interconverted between (*S*)‐ and (*R*)‐configurations when treated with silica or basic alumina (Scheme 3d). The (*S*)‐selective ATA256 catalysed reaction allowed for the isolation of (1*S*,3a*S*,9a*S*)‐**4c** consisting of two diastereomers and of the enantiomerically pure (1*S*,3a*R*,5a*S*,9a*S*)‐**4c** (Scheme 3c), in which the α‐epimerisation provided the more thermodynamically stable (*trans*‐fused) 5a*S*,9a*S*‐isomer (see Supporting Information for calculated energy differences between *cis‐* and *trans*‐fused isomers **5a**‐**9a**). Conversely, the opposite enantiomers (1*R*,3a*R*,9a*R*)‐**4c** and (1*R*,3a*S*,5a*R*,9a*R*)‐**4c** were isolated from the biotransformation catalysed by the (*R*)‐selective ATA025, and equivalent trends were observed for the tricyclic quinolizidines **4a**.

Having established TA methodology that goes far beyond the typical FGIs, enabling the generation of a panel of complex heterocycles from simple non‐chiral substrates, we next wanted to demonstrate that the enzyme could be used for further complexity generation. From the four stereocentres installed in the TA‐triggered DIMAMR (Scheme 4a), two centres were controlled by the enzyme's selectivity: C1 installed in the TA catalysed amination reaction, and the centre installed in the second aza‐Michael reaction, which was under substrate control and thus indirectly controlled by the enzyme. We envisioned that the two unresolved stereocentres, the tertiary α‐carbon and the β‐carbon set in the first aza‐Michael reaction, could be resolved in a TA catalysed DKR, *via* a base promoted double retro aza‐Michael racemisation (Scheme 4b). This type of racemisation had been observed when we attempted to epimerise the **4f** diastereomeric mixture under basic conditions (see Supporting Information).

**Scheme 4:**
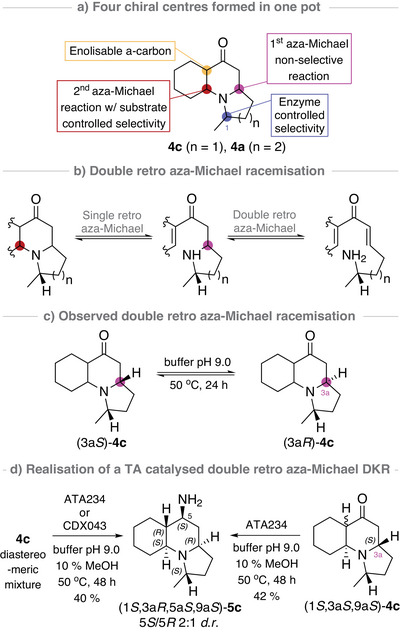
Observations of racemisation reactions and the realisation of an ATA catalysed double retro aza‐Michael dynamic kinetic resolution (DKR).

We therefore decided to record the stereoselective outcome from subjecting the purified isomer (1*S*, 3a*S*, 9a*S*)‐**4c** to the same conditions used during the biotransformation reactions but with no enzyme present (Scheme 4c). Pleasingly, after aging the compound in buffer at pH 9.0 for 24 h at 50 °C, the double retro aza‐Michael racemisation was evident by the formation of product (3a*R*‐**4c**) where C‐3a displayed (*R*)‐configuration, as determined by ^1^H NMR spectroscopic analysis, although this process was accompanied by some decomposition. Enzymatic DKR processes *via* α‐carbonyl racemisation are well established in the literature,^[^ ^29^, ^30^, ^31^ ^]^ and a TA‐mediated retro aza‐Michael DKR was presented by Peng *et al.* in the synthesis of a drug precursor.^[^ ^32^ ^]^ However, an enzymatic double retro aza‐Michael DKR has (to the best of our knowledge) not been reported.

We continued our investigation into the proposed enzymatic DKR by screening a panel of commercial TAs known to accept more sterically encumbered ketone substrates,^[^ ^33^ ^]^ using the diastereomeric mixture **4c** produced from the TA‐triggered DIMAMR methodology as our model compound (see Supporting Information for full results from the screening). From the panel, the two enzymes ATA234 and CDX043 were identified as capable of efficient amination of diastereomers **4c** (Scheme 4d). NMR spectroscopic analysis of the major product from preparative scale reactions, revealed the accumulation of (1*S*,3a*R*,5a*S*,9a*S*)‐**5c** in 40% isolated yield. Both enzymes (ATA234 and CDX043) demonstrated the ability to discriminate between the configurational isomers, selectively accepting the *trans*‐fused indolizidine (1*S*,3a*R*,5a*S*,9a*S*)‐**4c** to form diamine (1*S*,3a*R*,5a*S*,9a*S*)‐**5c** with moderate C‐5 selectivity. The two C‐5 isomers could be readily separated by flash column chromatography. Epimerisation of the β‐carbon C‐3a was furthermore verified when we obtained the same stereoisomeric product (1*S*,3a*R*,5a*S*,9a*S*)‐**5c,** with the (*R*)‐configured C‐3a, from the ATA234 catalysed biotransformation of purified (1*S*,3a*R*,5a*S*,9a*S*)‐**4c**, containing the (*S*)‐configured C‐3a (Scheme 4d, please see Section 5 in the Supporting Information for details).^[^ ^34^ ^]^

Next, the ketoenone precursor **1c** was tested with ATA‐256 and ATA‐234 in a one‐pot two enzyme reaction, yielding the major aminated product (1*S*,3a*R*,5a*S*,9a*S*)‐**5c**, representing a truly unique example of a one‐pot dual‐role ATA‐DKR cascade, in which a prochiral substrate is converted to a chiral natural product scaffold with five stereocentres under enzyme DKR and substrate control (Scheme 5). When the cascade was tested with ketoenone **1a**, a single enantiomerically pure aminated product (1*S*,5a*R*,6*S*,6a*S*,10a*S*)‐**5a** was obtained, meaning that the ATA‐234 can also selectively discriminate between the four quinolizidine stereoisomers (1*S*)‐**4a** in a resolution process and additionally install the primary amine with high stereoselectivity (Scheme 5). Interestingly, no DKR was observed for this substrate under the biotransformation conditions. From the cascade reaction, **5a** was separated from the remaining DIMAMR products **3a** and **4a** and acquired in 10% isolated yield and >99% *e*.*e*.

**Scheme 5:**
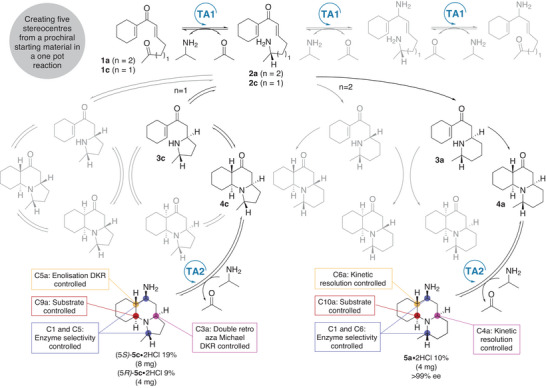
Realisation of a two enzyme one‐pot cascade for the TA‐triggered DIMAMR methodology coupled with a TA catalysed deracemisation step for the formation of **5c** by double retro aza‐Michael dynamic kinetic resolution or by TA‐catalysed kinetic resolution for the formation of **5a**.

In conclusion, we have developed the first single‐step biocatalytic route to high‐value indolizidine and quinolizidine moieties, demonstrated the first example of an enzyme‐triggered DIMAMR, and established a one‐pot two enzyme cascade capable of converting simple prochiral starting materials into highly complex novel *N*‐heterocycles containing up to five chiral centres; all achieved with a high degree of regio‐, chemo‐, and stereoselectivity. This work expands the repertoire of enzyme‐triggered reaction methodologies as well as enzymatic cascade processes, representing a truly unique example of a dual role TA cascade. Despite the success of the methodology, challenges remain, particularly in achieving complete double cyclisation for the quinolizidine systems. Additionally, the further development of the double retro aza‐Michael DKR process could significantly enhance the utility of this methodology. Screening or engineering a more selective TA for the amination of one DIMAMR product would strengthen the impact of the DKR process dramatically. This would yield a highly enantioselective process capable of obtaining a single indolizidine‐ or quinolizidine‐containing product with five chiral centres, all achieved in a one‐pot cascade, eliminating many of the inefficiencies associated with traditional, multistep methodologies including the need for stepwise stereocontrol, protecting groups and intermediate purification steps, increasing the overall sustainability and practicality of the methodology.

## Supporting Information

The authors have cited additional references within the Supporting Information.^[^ ^35^, ^36^, ^37^, ^38^, ^39^, ^40^, ^41^, ^42^, ^43^, ^44^, ^45^ ^]^

## Conflict of Interests

The authors declare no conflict of interest.

## Supporting information



Supporting Information

## Data Availability

The data that support the findings of this study are available in the supplementary material of this article.
