# Supplementary material for: Transaminase‐Triggered Cascades for the Synthesis and Dynamic Kinetic Resolution of Chiral N‐Heterocycles
Source: Angew Chem Int Ed Engl. 2025 Apr 18;64(21):e202422584. doi: 10.1002/anie.202422584 (PMC12087864; doi:10.1002/anie.202422584)
Supplement: Supplementary file 1 — Supporting Information [file ANIE-64-e202422584-s001.pdf]

Contents

1. General Methods 1
1.1. Preparation of aldehyde precursors 2
1.2. Substrate synthesis 5
1.3. Optimisation of biotransformation conditions 21
2. DIMAMR biotransformations 24
2.1. One-pot two enzyme biotransformations 31
3. Additional selectivity 33
4. Attempted epimerisation of 4f 34
5. Enzyme screen for the formation of diamine 5c from 4c 35
6. Study of reaction conditions for the two-enzyme cascade 36
7. NMR Spectra 40
8. Chiral GC-MS Chromatograms 123
9. Chiral HPLC Chromatograms 124
10. Energy calculations 125
11. H-H proximity calculation 127
12. Atomic coordinates 145
13. References 180

1. General Methods

NMR spectra were recorded on a Bruker Avance 400 spectrometer (^{1}H 400 MHz, ^{13}C 101 MHz). The chemical shifts were recorded in ppm with the residual $CHCl_3$ signal referenced to 7.26 ppm and 77.16 ppm for ^{1}H and ^{13}C, respectively. When D_2O was used, the residual signal was referenced to 4.79 ppm for ^{1}H NMR. Coupling constants (*J*) are reported in Hz and refer to the observed peak multiplicities, where needed, 2D NMR was preformed to assign specific carbon atoms and stereochemistry of compounds. Thin layer chromatography was performed on Merck silica gel 60 F_{254} plates or Merck aluminium oxide 60 F_{254} neutral plates. Flash column chromatography was performed on silica gel (60 Å, 230-400 mesh) or Aluminium oxide 90 standardised.

Materials: Commercially available reagents, purchased from Sigma Aldrich or Acros, were used throughout without further purification. Anhydrous THF and CH_2Cl_2 were

obtained from a Pure Solvent apparatus. Commercially available transaminases were purchased from Codexis® in the form of lyophilised cell extract.

1.1. Preparation of aldehyde precursors

Hexane-1,4-diol (S1)

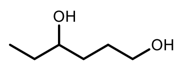

A solution of γ-caprolactone (1.6 mL, 14.3 mmol, 1.0 equiv.) in Et_2O (30 mL) was added dropwise to a suspension of $LiAlH_4$ (2.0 g, 57.2 mmol, 4.0 equiv.) in Et_2O (70 mL) at 0 °C. The reaction was stirred at rt for 19 h and quenched with the dropwise addition of sat. aq. Na_2SO_4 (4.5 mL) at 0 °C. The resulting white suspension was stirred vigorously for 30 min and then filtered through Celite. The filtrate was dried over $MgSO_4$, filtered, and concentrated under reduced pressure. The title product (0.783 g, 46%) was used directly in the next step without further purification. **^{1}H NMR** (400 MHz, $CDCl_3$) δ 3.71 – 3.64 (m, 1H), 3.64 – 3.58 (m, 1H), 3.58 – 3.50 (m, 1H), 1.74 – 1.57 (m, 3H), 1.56 – 1.38 (m, 3H), 0.92 (t, *J* = 7.5 Hz, 3H). **^{13}C NMR** (101 MHz, $CDCl_3$) δ 73.3, 63.0, 34.0, 30.4, 29.2, 10.1; **HRMS-ESI (m/z):** $C_6H_{15}O_2^+$ $[M+H]^+$ theoretical 119.1027, found 119.1042. Data is consistent with literature.[1]

4-Oxohexanal (S2)

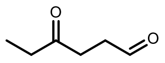

A solution of $(COCl)_2$ (2.2 mL, 25.2 mmol, 4.26 equiv.) in DCM (50 mL) was added a solution of DMSO (3.4 mL, 47.8 mmol, 8.07 equiv.) in DCM (3.4 mL) at -78 °C under nitrogen and stirred at this temperature for 10 min. The mixture was then dropwise added a solution of hexane-1,4-diol (0.700 g, 5.92 mmol, 1.0 equiv.) in DCM (25 mL) and stirred for 30 min at -78 °C. After this time the reaction was dropwise added Et_3N (12 mL, 86.1 mmol, 13.78 equiv.) and allowed to reach rt over 15 h. The reaction mixture was then added sat. aq. $NaHCO_3$ (20 mL) and extracted with EtOAc (50 mL x 4). The combined organic fractions were dried over $MgSO_4$, filtered, and concentrated under reduced pressure. The title product was used in the next step without further purification. **^{1}H NMR** (400 MHz, $CDCl_3$) δ 9.79 (1H, s), 2.78 – 2.66 (4H, m), 2.47 (2H, q, *J* = 7.3 Hz), 1.05 (3H, t, *J*

= 7.3 Hz); **^{13}C NMR** (101 MHz, $CDCl_3$) δ 209.4, 200.7, 37.6, 36.0, 34.3, 7.9; **HRMS-ESI (m/z):** $C_6H_{11}O_2^+$ [M+H]$^+$ theoretical 115.0714, found 115.0754. Data is consistent with literature.[2]

General Procedure for the oxidative cleavage of 1-ethylcyclopent-1-ene, 1-methylcyclopentene and 6-methyl-5-hepten-2-one.

A stream of ozone was bubbled through a solution of the corresponding vinyl reagent (1 eq, 11.39 mmol) in CH_2Cl_2 (100 mL) at -78 °C for approx. 15 minutes until the solution turned a pale blue colour. Triphenylphosphine (1.1 eq, 3.286 g, 12.53 mmol) was then added, and the solution was left to react with stirring at r.t. for 16 h. The solution was concentrated *in vacuo* and the residue was purified by flash chromatography to provide the corresponding ketoaldehyde.

5-Oxohexanal (S3)

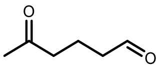

Derived from 1-methylcyclopentene. Colourless oil (0.870 g, 67 % yield) eluted in EtOAc / Cyclohexane (2:1) with an R_f = 0.42. **^{1}H NMR** (400 MHz, $CDCl_3$) δ_H 9.53 (1H, t, *J* = 1.4 Hz), 2.35-2.24 (4H, m), 1.92 (3H, s), 1.65 (2H, ap. quin, *J* = 7.1 Hz); **^{13}C NMR** (101 MHz, $CDCl_3$) δ_C 207.8, 201.7, 42.5, 41.8, 29.4, 15.6; **HRMS-ESI** (m/z): $C_6H_{11}O_2^+$ [M+H]$^+$ theoretical 115.0754, found 115.0764.

4-Oxopentanal (S4)

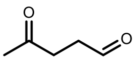

Derived from 6-methyl-5-hepten-2-one. Colourless oil (0.584 g, 43 %) eluted in EtOAc / Cyclohexane (1:9) with an R_f = 0.30. **^{1}H NMR** (400 MHz, $CDCl_3$) δ_H 9.66 (1H, s), 2.63 (4H, m), 2.08 (3H, s); **^{13}C NMR** (101 MHz, $CDCl_3$) δ_C 206.5, 200.5, 37.3, 35.4, 29.6; **HRMS-ESI** (m/z): $C_5H_9O_2^+$ [M+H]$^+$ theoretical 101.0558, found 101.0539.

1-Ethylcyclopent-1-ene (S5)

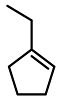

Ethyl magnesium bromide (16.0 mL, 3 M in Et_2O, 48.00 mmol, 1.2 equiv.) was added to cyclopentanone (3.54 mL, 40.00 mmol, 1.0 equiv.) in Et_2O (40 mL) under nitrogen at 0 °C. The mixture was then allowed to warm to room temperature and stirred for a further 2 h. The reaction mixture was then carefully quenched with cold water followed by sat. NH_4.Cl solution. The organic phase was separated, and the aqueous layer extracted with Et2O (2 × 50 mL). The combined organic extracts were dried over $MgSO_4$ and concentrated under reduced pressure to provide the crude 1-ethylcyclopentanol (3.884 g, 85% yield). To this crude, 85% wt. phosphoric acid (0.20 mL, 1.71 mmol, 0.05 equiv.) was added and the mixture was heated to 115 °C. The product was distilled off as it formed, and the biphasic solution was separated to provide crude 1-ethylcyclopentene as a colourless oil (1.042 g, 27% yield). **^{1}H NMR** (400 MHz, $CDCl_3$) δ_H 5.35-5.29 (1H, m), 2.35-2.26 (2H, m), 2.26-2.19 (2H, m), 2.11-2.02 (2H, m), 1.89-1.80 (2H, m), 1.04 (3H, t, J = 7.3 Hz); **^{13}C NMR** (101 MHz, $CDCl_3$) δ_C 146.8, 122.1, 35.2, 32.5, 24.3, 23.6, 12.5; **HRMS-ESI (m/z):** $C_7H_{13}^+$ $[M+H]^+$ theoretical 97.0973, found 97.0999. Data is consistent with literature.[3]

5-Oxoheptanal (S6)

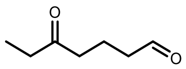

Derived from 1-Ethylcyclopent-1-ene. Colourless oil (0.666 g, 48 %) eluted in EtOAc / Cyclohexane (2:1) with an R_f = 0.60. **^{1}H NMR** (400 MHz, $CDCl_3$) δ_H 9.74 (1H, t, *J* = 1.45 Hz), 2.49-2.44 (4H, m), 2.40 (2H, q, *J* = 7.28 Hz), 1.88 (2H, quin, *J* = 7.09 Hz), 1.03 (3H, t, *J* = 7.40 Hz); **^{13}C NMR** (101 MHz, $CDCl_3$) δ_C 210.8, 202.0, 43.1, 41.0, 36.0, 16.2, 7.9; **HRMS-ESI** (m/z): $C_7H_{13}O_2^+$ $[M+H]^+$ theoretical 129.0871, found 129.0892.

1.2. Substrate synthesis

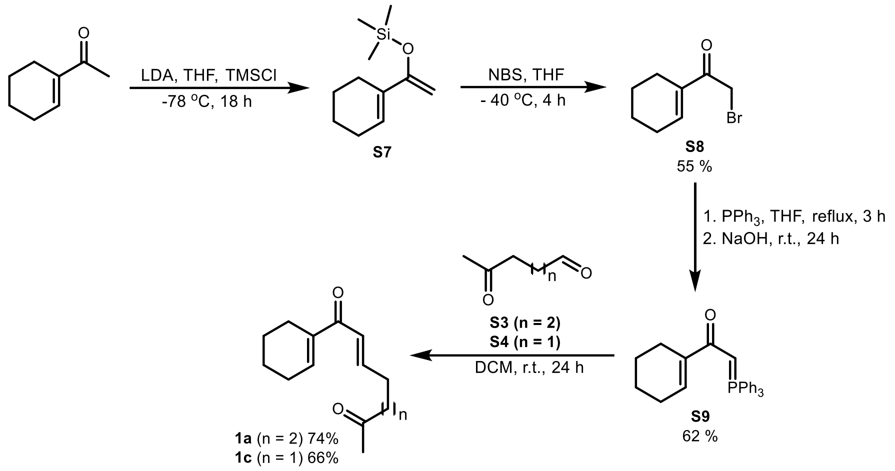

Scheme S1: *Synthesis of the bis-conjugated enone substrates **1a** and **1c** starting from 1-acetyl-1-cyclohexene.*

1-(1-Trimethylsilyloxy)vinyl-1-cyclohexene (S7)

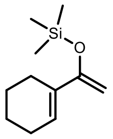

Lithium diisopropylamine (22.5 mL, 2M sol., 45.00 mmol, 1.45 equiv.) was dissolved in dry THF (20 mL) under inert conditions at -78 °C. 1-acetyl-1-cyclohexene (4.00 mL, 31.11 mmol, 1.0 equiv.) in dry THF (7 mL) was added dropwise and was stirred for 1 hr. Trimethylsilyl chloride (5.92 mL, 46.67 mmol, 1.5 equiv.) was then added, and the mixture was allowed to warm to r.t. overnight. The solution was then cooled to 0 °C, quenched with sat. ammonium chloride solution (25 mL) and extracted with ethyl acetate (3 x 15 mL). The organic fractions were combined and dried over $MgSO_4$ and concentrated *in vacuo* to give the crude title compound as an orange oil (R_f = 0.61 with EtOAc / Cyclohexane (1:9)). **^{1}H NMR** (400 MHz, $CDCl_3$) δ_H 6.23 - 6.17 (1H, m), 4.27 (2H, d, *J* = 63.7 Hz), 2.18 - 2.10 (4H, m), 1.72 - 1.53 (4H, m), 0.22 (9H, s); **^{13}C NMR** (101 MHz, $CDCl_3$) δ_C 156.7, 133.2, 125.4, 89.8, 25.6, 25.0, 22.9, 22.2, 0.1. Data is consistent with literature.[4]

1-Bromo-2-(cyclohex-1-enyl)ethan-one (S8)

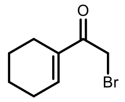

N-bromosuccinimide (6.092 g, 34.23 mmol, 1.1 equiv.) was added to a THF solution (45 mL) of the crude silyl enol ether **S7** at -40 °C and the mixture was stirred overnight at the same temperature. The mixture was then poured into sat. aq. $Na_2S_2O_3$ solution (60 mL) and the aqueous layer extracted with EtOAc (3 x 30 mL). The combined organic extracts were then washed with brine (2 ×60 mL), dried over $MgSO_4$ and concentrated *in vacuo*. The crude material was purified by column chromatography on silica gel (EtOAc / Cyclohexane (1:20)) to provide the title compound as an orange oil (3.49 g, 55 % yield). R_f 0.31 (EtOAc / Cyclohexane (1:9)). **^{1}H NMR** (400 MHz, $CDCl_3$) δ_H 6.99 - 6.95 (1H, m), 4.15 (2H, s), 2.31 - 2.22 (4H, m), 1.68 - 1.57 (4H, m); **^{13}C NMR** (101 MHz, $CDCl_3$) δ_C 199.3, 142.9, 136.8, 30.2, 26.2, 23.2, 21.7, 21.3. Data is consistent with literature.[4]

1-Cyclohex-1-enyl-2-(triphenylphosphanylidene)ethenone (S9)

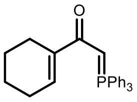

To 1-bromo-2-(cyclohex-1-enyl)ethan-2-one (**S8**) (3.490 g, 17.18 mmol, 1.0 equiv.) in THF (35 mL) was added triphenylphosphine (4.507 g, 17.18 mmol, 1.0 equiv.) and heated to reflux for 4 h. The reaction was allowed to cool to r.t. and the precipitate was collected by filtration. The residue was then suspended in MeOH:H_2O (1:1, 75 mL) and NaOH solution (1M) was added until the mixture became slightly basic and stirred for 24 h. The MeOH was removed under reduced pressure and the aqueous solution was extracted with DCM (3 x 50 mL). The combined organic extracts were then washed with brine (50 mL), dried with $MgSO_4$, filtered, and concentrated *in vacuo* to provide the title compound as a beige solid (4.128 g, 62 %). **^{1}H NMR** (400 MHz, $CDCl_3$) δ_H 7.69 - 7.62 (6H, m), 7.55 - 7.49 (3H, m), 7.47 - 7.41 (6H, m), 6.64 (1H, m), 3.92 (1H,m), 2.42 - 2.36 (2H, m), 2.18 - 2.11 (2H, m), 1.71 - 1.63 (2H, m), 1.62 - 1.54 (2H, m); **^{13}C NMR** (101 MHz, $CDCl_3$) δ_C 187.2,133.2 (3C), 133.1 (6C), 131.9 (3C), 128.8 (6C), 128.0, 127.1, 50.2 (d, *J* = 110 Hz), 25.8, 25.7, 23.0, 22.2; **HRMS-ESI (m/z):** $C_{26}H_{26}OP^+$ $[M+H]^+$ theoretical 385.1677, found 385.1721.

(2*E*)-1-(Cyclohex-1-en-1-yl)oct-2-ene-1,7-dione (1a)

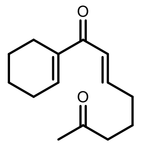

1-cyclohex-1-enyl-2-(triphenylphosphanylidene)ethenone (1.301 g, 3.38 mmol, 1.1 equiv.) was added to a solution of 5-oxohexanal (0.351 g, 3.07 mmol, 1.0 equiv.) in DCM (20 mL) and allowed to react at r.t. for 18 hrs. The DCM was removed under reduced pressure and purified via column chromatography on silica gel (EtOAc / Cyclohexane (1:19)) to provide the title compound as a yellow oil (0.503 g, 74 % yield). **^{1}H NMR** (400 MHz, $CDCl_3$) δ_H 6.84-6.78 (1H, m), 6.75-6.66 (1H, dt, *J* = 15.31, 6.84 Hz), 6.60-6.53 (1H, d, *J* = 15.31 Hz), 2.38 (2H, t, *J* = 7.31 Hz), 2.21-2.11 (6H, m), 2.04 (3H, s), 1.67 (2H, quin, *J* = 5.89), 1.60-1.48 (4H, m); **^{13}C NMR** (101 MHz, $CDCl_3$) δ_C 208.2, 191.0, 145.4, 140.1, 139.6, 125.4, 42.5, 31.6, 29.87, 26.0, 23.3, 22.1, 21.8, 21.5; **HRMS-ESI (m/z):** $C_{14}H_{21}O_2^+$ $[M+H]^+$ theoretical 221.1497, found 221.1537.

(2*E*)-1-(Cyclohex-1-en-1-yl)hept-2-ene-1,6-dione (1c)

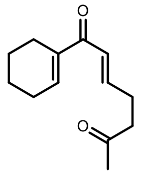

1-cyclohex-1-enyl-2-(triphenylphosphanylidene)ethenone (2.691 g, 6.99 mmol, 1.2 equiv.) was added to a solution of 4-oxopentanal (0.584 g, 5.833 mmol, 1.0 equiv.) in DCM (20 mL) and allowed to react at r.t. for 18 hrs. The DCM was removed under reduced pressure and purified via column chromatography on silica gel (EtOAc / Cyclohexane (1:9)) to provide the title compound as a yellow oil (0.793 g, 66 % yield). **^{1}H NMR** (400 MHz, $CDCl_3$) δ_H 6.90-6.86 (1H, m), 6.77 (1H, dt, *J* = 15.2, 6.6 Hz), 6.69-6.62 (1H, d, J = 15.3 Hz), 2.64-2.57 (2H, m), 2.52-2.45 (2H, m), 2.30-2.22 (4H, m), 2.15 (3H, s), 1.68-1.57 (4H, m); **^{13}C NMR** (101 MHz, $CDCl_3$) δ_C 207.2, 191.2, 144.5, 140.4, 139.9, 125.7, 41.9, 30.1, 26.6, 26.3, 23.5, 22.0, 21.7; **HRMS-ESI (m/z):** $C_{13}H_{19}O_2^+$ $[M+H]^+$ theoretical 207.1340, found 207.1381.

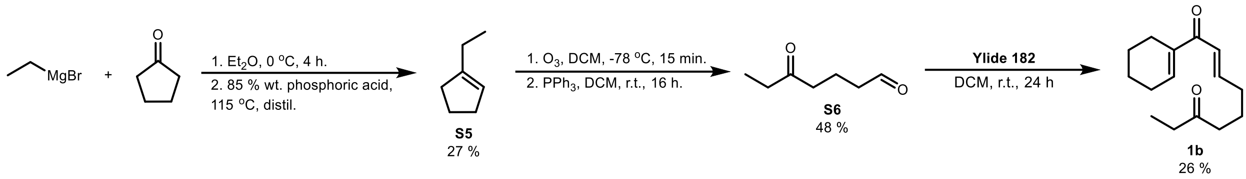

Scheme S2: *Synthesis of the bis-conjugated ethyl derivative substrate **193** starting from cyclopentanone **190**.*

(*E*)-1-(Cyclohex-1-en-1-yl)non-2-ene-1,7-dione (1b)

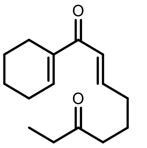

1-cyclohex-1-enyl-2-(triphenylphosphanylidene)ethenone (2.198 g, 5.72 mmol, 1.1 equiv.) was added to a solution of 5-oxoheptanal (0.666 g, 5.20 mmol, 1.0 eqiuv.) in DCM (20 mL) and allowed to react at r.t. for 18 hrs. The DCM was removed under reduced pressure and purified via column chromatography on silica gel (EtOAc / Cyclohexane (1:19)) to provide the title compound as a yellow oil (0.310 g, 26 % yield). **^{1}H NMR** (400 MHz, $CDCl_3$) δ_H 6.90-6.86 (1H, m), 6.79 (1H, dt, *J* = 15.26, 6.84 Hz), 6.66-6.61 (1H, m), 2.46-2.37 (4H, m), 2.30-2.19 (6H, m), 1.76 (2H, quin, *J* = 7.37 Hz), 1.69-1.56 (4H, m), 1.04 (3H, t, *J* = 7.40 Hz); **^{13}C NMR** (101 MHz, $CDCl_3$) δ_C 211.2, 191.3, 145.7, 140.3, 139.9, 125.6, 41.5, 36.1, 31.9, 26.3, 23.5, 22.4, 22.1, 21.7, 7.9; **HRMS-ESI (m/z):** $C_{15}H_{23}O_2^+$ $[M+H]^+$ theoretical 235.1653, found 235.1694.

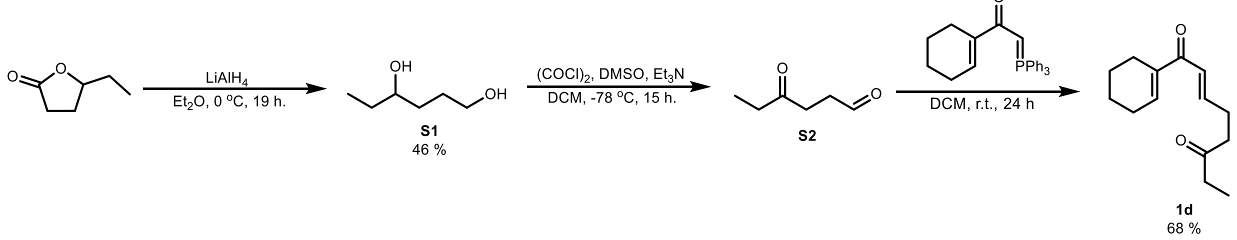

Scheme S3: *Synthesis of the bis-conjugated ethyl derivative substrate **1d** from 5-ethyldihydrofuran-2-one.*

(*E*)-1-(Cyclohex-1-en-1-yl)oct-2-ene-1,6-dione (1d)

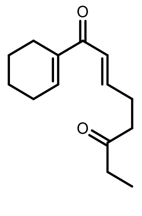

1-cyclohex-1-enyl-2-(triphenylphosphanylidene)ethenone (1.36 g, 3.54 mmol, 1.2 equiv.) was added to a solution of 4-Oxohexanal (0.336 g, 2.95 mmol, 1.0 equiv.) in DCM (10 mL) and allowed to react at r.t. for 18 hrs. The DCM was removed under reduced pressure and purified via column chromatography on silica gel (EtOAc / cyclohexane (1:7)) to provide the title compound (R_f = 0.5, EtOAc / Cyclohexane (1:2)) as a yellow oil (0.440 g, 68 % yield). **^{1}H NMR** (400 MHz, CDCl3) δ 6.87 (1H, t, *J* = 3.8 Hz), 6.77 (1H, dt, J = 15.3, 6.5 Hz), 6.65 (1H, d, *J* = 15.3 Hz), 2.62 – 2.53 (2H, m), 2.49 (2H, dt, J = 7.9, 6.6 Hz), 2.42 (2H, q, J = 7.3 Hz), 2.28-2.22 (4H, m), 1.67-1.57 (4H, m), 1.05 (3H, t, J = 7.3 Hz); **^{13}C NMR** (101 MHz, CDCl3) δ 209.9, 191.3, 144.8, 140.4, 139.9, 125.7, 40.6, 36.2, 26.7, 26.3, 23.5, 22.1, 21.7, 7.9; **HRMS-ESI (m/z):** $C_{14}H_{21}O_2^+$ [M+H]$^+$ theoretical 221.1497, found 221.1537.

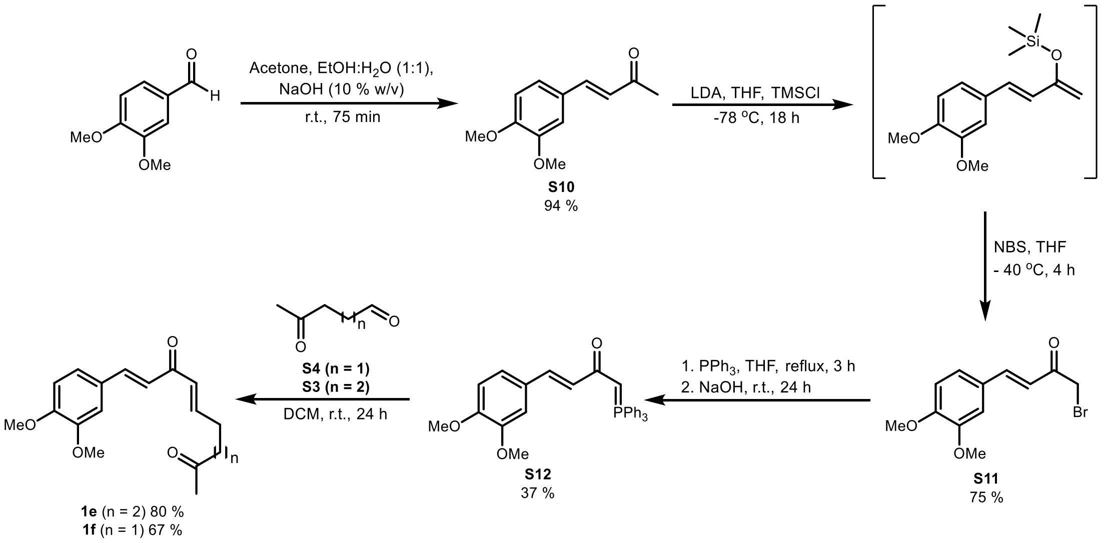

***Scheme S4:** Synthesis of the bis-conjugated enone substrates **1e** and **1f** starting from 3,4-dimethoxybenzaldehyde.*

3,4-Dimethoxybenzylideneacetone (S10)

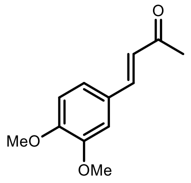

To a solution of 3,4-dimethoxybenzaldehyde (3.380 g, 20.4 mmol, 1.0 equiv.) dissolved in aq. ethanol (270 mL, 50 % v/v), acetone (7.56 mL, 102.0 mmol, 5.0 equiv.) and then NaOH solution (25 mL, 10% w/v) was added dropwise and left to react for 70 min. The reaction mixture was neutralised to pH 7.0 using 4M HCl, extracted with CH_2Cl_2, dried using $MgSO_4$ and concentrated *in vacuo*. The crude product was recrystalised diethyl ether: cyclohexane (1:1) to yield the product as a yellow crystal (3.96 g, 94 % yield). **^{1}H NMR** (400 MHz, $CDCl_3$) δ_H 7.46 (1H, d, *J* = 12.95 Hz), 7.12 (1H, dd, *J* = 6.63, 1.58 Hz), 7.07 (1H, d, *J* = 1.62 Hz), 6.88 (1H, d, *J* = 6.62 Hz), 6.60 (1H, d, *J* = 12.82 Hz), 3.92 (6H, s), 2.37 (3H, s); **^{13}C NMR** (101 MHz, $CDCl_3$) δ_C 198.5, 151.5, 149.4, 143.6, 127.5, 125.4, 123.15, 111.2, 109.7, 56.1, 56.0, 27.5; **HRMS-ESI (m/z):** $C_{12}H_{15}O_3^+$ $[M+H]^+$ theoretical 207.0976, found 207.1017. Data is consistent with literature.[5]

(*E*)-1-Bromo-4-(3,4-dimethoxyphenyl)but-3-en-2-one (S11)

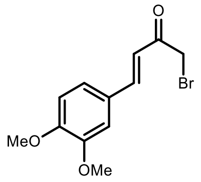

Lithium diisopropylamine (7.25 mL, 14.50 mmol, 1.45 equiv.) was dissolved in dry THF (10 mL) under inert conditions at -78 °C. 3,4-dimethoxybenzylideacetone (2.062 g, 10.00 mmol, 1.0 equiv.) in dry THF (5 mL) was added dropwise and was stirred for 1 hr. Trimethylsilyl chloride (1.903 mL, 15.00 mmol, 1.5 equiv.) was then added, and the mixture was allowed to warm to r.t. overnight. The solution was then cooled to 0 °C, quenched with sat. ammonium chloride solution (25 mL) and extracted with ethyl acetate (3 x 15 mL). The organic fractions were combined and dried over $MgSO_4$ and concentrated *in vacuo* to give the crude silyl enol ether as an orange oil. N-bromosuccinimide (1.958 g, 11.00 mmol, 1.1 equiv.) was added to a THF solution (15 mL) of the silyl enol ether at -40 °C and the mixture was stirred overnight at the same temperature. The mixture was then

poured into sat. aq. $Na_2S_2O_3$ solution (30 mL) and the aqueous layer extracted with EtOAc (3 x 20 mL). The combined organic extracts were then washed with brine (30 mL), dried over $MgSO_4$ and concentrated *in vacuo*. The crude material was purified by column chromatography on silica gel (EtOAc / Cyclohexane (1:9)) to provide the title compound as a brown oil (2.148 g, 75 % yield). R_f 0.70 (EtOAc / Cyclohexane (1:9)). **^{1}H NMR** (400 MHz, $CDCl_3$) δ_H 7.63 (1H, d, J = 15.98 Hz), 7.16 (1H, dd, J = 8.31, 1.97 Hz), 7.07 (1H, d, J = 2.00 Hz), 6.87 (1H, d, J = 8.28 Hz), 6.79 (1H, d, J = 15.92 Hz), 4.07 (2H, s), 3.91-3.90 (6H, m); **^{13}C NMR** (101 MHz, $CDCl_3$) δ_C 191.0, 152.0, 149.4, 145.6, 126.9, 123.8, 120.2, 111.2, 110.0, 56.1, 56.0, 33.1.

(*E*)-4-(3,4-Dimethoxyphenyl)-1-(triphenylphosphaneylidene)but-3-en-2-one (S12)

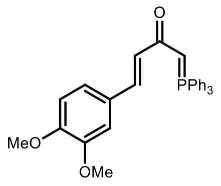

To 1-bromo-4-(3,4-dimethoxyphenyl)but-3-en-2-one (2.148 g, 7.533 mmol, 1.0 equiv.) in THF (15 mL) was added triphenylphosphine (2.173 g, 8.286 mmol, 1.1 equiv.) and heated to reflux for 4 h. The reaction was allowed to cool to r.t. and the precipitate was collected by filtration. The residue was then suspended in MeOH:H_2O (1:1, 40 mL) and NaOH solution (1M) was added until the mixture became slightly basic and stirred for 24 h. The MeOH was removed under reduced pressure and the aqueous solution was extracted with DCM (3 x 30 mL). The combined organic extracts were then washed with brine (30 mL), dried with $MgSO_4$, filtered, and concentrated *in vacuo* to provide the title compound as a beige solid (1.275 g, 37 %). **^{1}H NMR** (400 MHz, $CDCl_3$) δ_H 7.69 (6H, m), 7.55 (3H, m), 7.47 (6H, m), 7.35 (1H, d, J = 15.61 Hz), 7.06 (2H, m), 6.79 (2H, m), 5.29 (1H, s), 3.88 (3H, s), 3.87 (3H, s); **^{13}C NMR** (101 MHz, $CDCl_3$) δ_C 183.0, 149.3, 148.9, 133.8, 133.2 (6C), 132.1 (3C), 132.0 (3C), 128.9 (6C), 127.3, 126.4, 121.2, 111.0, 109.4, 55.8, 55.7, 53.4; **HRMS-ESI (m/z):** $C_{30}H_{28}O_3P^+$ $[M+H]^+$ theoretical 467.1731, found 467.1772.

(6*E*,9*E*)-10-(3,4-Dimethoxyphenyl)deca-6,9-diene-2,8-dione (1e)

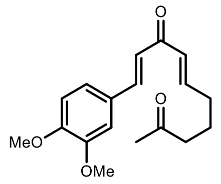

4-(3,4-dimethoxyphenyl)-1-(triphenylphosphanylidene)but-3-en-2-one (0.300 g, 0.64 mmol, 1.0 equiv.) was added to a solution of 4-oxopentanal (0.073 g, 0.64 mmol, 1.0 equiv.) in DCM (5 mL) and allowed to react at r.t. for 24 hrs. The DCM was removed under reduced pressure and purified via column chromatography on silica gel (EtOAc / Cyclohexane (1:4)) to provide the title compound as a yellow crystal (0.194 g, 80 % yield). **^{1}H NMR** (400 MHz, $CDCl_3$) δ_H 7.60 (1H, d, *J* = 12.7 Hz), 7.16 (1H, dd, *J* = 6.6, 1.5 Hz), 7.10 (1H, d, *J* = 1.5 Hz), 6.94 (1H, dt, *J* = 12.5, 5.4 Hz), 6.88 (1H, d, *J* = 6.7), 6.82 (1H, d, *J* = 12.7 Hz), 6.46 (1H, dt, *J* = 12.5, 1.2 Hz), 3.93 (3H, s), 3.92 (3H, s), 2.49 (2H, t, *J* = 5.8 Hz), 2.32-2.26 (2H, m), 2.15 (3H, s), 1.81 (2H, quin, *J* = 5.9 Hz); **^{13}C NMR** (101 MHz, $CDCl_3$) δ_C 208.4, 189.1, 151.5, 149.4, 146.5, 143.5, 129.7, 127.9, 123.2, 123.2, 111.3, 109.9, 56.1, 56.1, 42.8, 31.9, 30.2, 22.2; **HRMS-ESI (m/z):** $C_{18}H_{23}O_4^+$ $[M+H]^+$ theoretical 303.1552, found 303.1591.

(5*E*,8*E*)-9-(3,4-Dimethoxyphenyl)nona-5,8-diene-2,7-dione (1f)

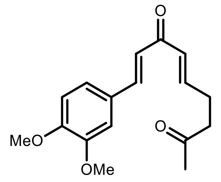

4-(3,4-dimethoxyphenyl)-1-(triphenylphosphanylidene)but-3-en-2-one (1.633 g, 3.50 mmol, 1.0 equiv.) was added to a solution of 4-oxopentanal (0.350 g, 3.50 mmol, 1.0 equiv.) in DCM (20 mL) and allowed to react at r.t. for 24 hrs. The DCM was removed under reduced pressure and purified via column chromatography on silica gel (EtOAc / Cyclohexane (1:9)) to provide the title compound as a yellow crystal (0.674 g, 67 % yield). **^{1}H NMR** (400 MHz, $CDCl_3$) δ_H 7.59 (1H, d, *J* = 15.9 Hz), 7.16 (1H, dd, *J* = 8.4, 1.9 Hz), 7.09 (1H, d, *J* = 1.9 Hz), 6.94 (1H, dt, *J* = 15.6, 6.8 Hz), 6.87 (1H, d, J= 8.3 Hz), 6.81 (1H, d, *J* = 15.9 Hz), 6.46 (1H, dt, *J* = 15.6, 1.5 Hz), 3.93 (3H, s,), 3.92 (3H, s), 2.66 (2H, t, *J* = 6.6 Hz), 2.55 (2H, m), 2.18 (3H, s); **^{13}C NMR** (101 MHz, $CDCl_3$) δ_C 207.1, 189.0, 151.5, 149.4, 145.5,

143.6, 129.8, 127.8, 123.2, 123.1, 111.2, 109.9, 56.1, 56.0, 41.8, 30.1, 26.5; **HRMS-ESI (m/z):** $C_{17}H_{21}O_4^+$ $[M+H]^+$ theoretical 289.1395, found 289.1434.

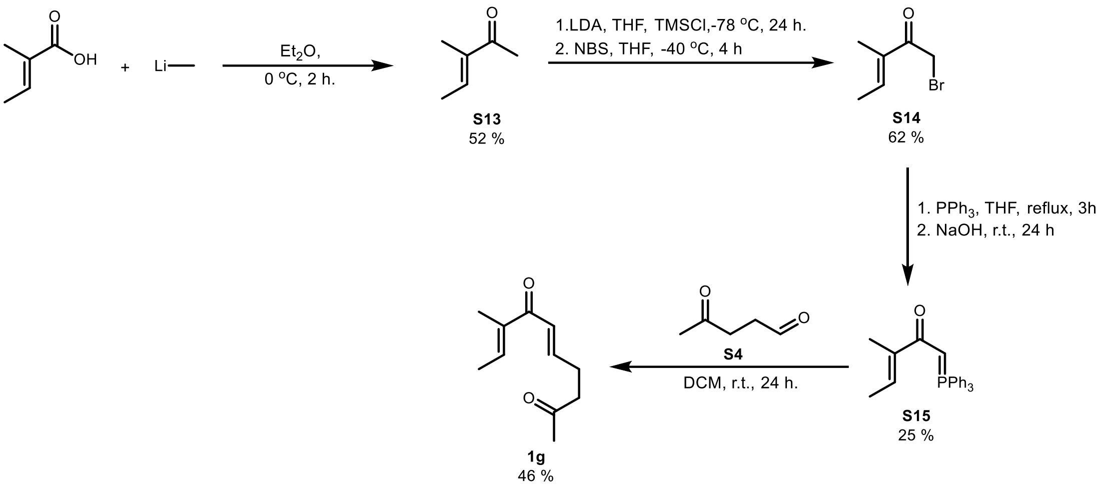

Scheme S5: *Synthesis of the bis-conjugated enone substrate **1g** starting from tiglic acid.*

(*E*)-3-Methylpent-3-en-2-one (S13)

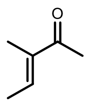

A solution of methyllithium (25 ml, 1.6 M in diethyl ether, 40 mmol, 2.0 equiv.) was added to a solution of tiglic acid (2.002 g, 20 mmol, 1.0 equiv.) in dry diethyl ether (46 ml) at 0 °C, and the mixture was stirred at the same temperature for 2 h. To the resulting mixture at 0 °C was gradually added 0.4 M hydrochloric acid (80 ml), and the mixture was extracted with diethyl ether. The extract was washed with brine, dried over $MgSO_4$, and concentrated. The residue was distilled under reduced pressure to give pentanone (0.773 g, 52 %) as an oil. **^{1}H NMR** (400 MHz, $CDCl_3$) δ_H 6.73 (1H, qq, *J* = 6.9, 1.3 Hz), 2.29 (3H, s), 1.87 – 1.84 (3H, m), 1.77 – 1.75 (3H, m); **^{13}C NMR** (101 MHz, $CDCl_3$) δ_C 199.9, 139.9, 138.5, 25.5, 14.7, 10.9; Data is consistent with literature.[6]

(*E*)-1-Bromo-3-methylpent-3-en-2-one (S14)

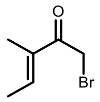

The pentanone **165** (0.773 g, 7.47 mmol, 1.0 equiv.) was dissolved in dry THF (15 ml) and left to the side of LDA (5.415 ml, 2M in THF/hexanes, 10.83 mmol, 1.45 equiv.) at -78 °C. The enone solution was added dropwise to the LDA solution and left to react for 40 min. TMSCl (1.422 ml, 11.20 mmol, 1.5 equiv.) was then added to the mixture and the reaction was left to react for 18 h. The solution was cooled to 0 °C and quenched with sat. NH_4Cl (20 ml). The organic layer was collected and separated. The aqueous layer was extracted with ethyl acetate (3 x 15 ml) and the organic layers were combined and concentrated to yield an orange oil. This crude silyl enol ether was dissolved in dry THF (15 ml) and N-bromosuccinimide (1.462 g, 1.1 equiv., 8.21 mmol) was added at -40 °C and left to react o.n. The reaction was quenched with $Na_2S_2O_3$ and the organic layer was separated. The aqueous layer was extracted with diethyl ether (3 x 15 ml) and the organic layers were combined and concentrated *in vaccuo*. The crude product was purified by flash column chromatography (silica dry-load, cyclohexane: ethyl acetate (9:1)) as a yellow oil (0.858 g, 62 %). **^{1}H NMR** (400 MHz, $CDCl_3$) δ_H 6.80 (1H, ap. qq, *J* = 6.9, 1.3 Hz), 4.15 (2H, s), 1.91 (3H, m), 1.83 (3H, s); **^{13}C NMR** (101 MHz, $CDCl_3$) δ_C 192.7, 140.2, 135.8, 53.4, 15.0, 11.3; Data is consistent with literature.[7]

(*E*)-3-Methyl-1-(triphenylphosphaneylidene)pent-3-en-2-one (S15)

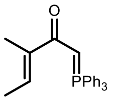

To 1-bromo-3-methylpent-3-en-2-one (0.858 g, 4.846 mmol, 1.0 equiv.) in THF (15 mL) was added triphenylphosphine (1.398 g, 5.331 mmol, 1.1 equiv.) and heated to reflux for 4 h. The reaction was allowed to cool to r.t. and the precipitate was collected by filtration. The residue was then suspended in MeOH:H_2O (1:1, 25 mL) and NaOH solution (1M) was added until the mixture became slightly basic and stirred for 24 h. The MeOH was removed under reduced pressure and the aqueous solution was extracted with DCM (3 x 20 mL). The combined organic extracts were then washed with brine (25 mL), dried with $MgSO_4$, filtered, and concentrated *in vacuo* to provide the title compound as a beige solid

(0.070 g, 25 %). **^{1}H NMR** (400 MHz, $CDCl_3$) δ_H 7.70 - 7.62 (6H, m), 7.55 - 7.49 (3H, m), 7.47 - 7.41 (6H, m), 6.54-6.47 (1H, m), 3.98 (1H, br s), 1.91 (3H, s), 1.73-1.70 (3H, m); **^{13}C NMR** (101 MHz, $CDCl_3$) δ_C 187.8, 133.1, 133.0, 131.8 (3C),131.8 (3C), 128.8 (6C), 128.7 (6C), 50.7, 13.9, 13.4; **HRMS-ESI (m/z):** $C_{24}H_{24}OP^+$ $[M+H]^+$ theoretical 359.1520, found 359.1561. Data is consistent with literature.[7]

8-Methyldeca-5,8-diene-2,7-dione (1g)

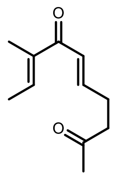

3-methyl-1-(triphenylphosphaneylidene)pent-3-en-2-one (0.070 g, 0.195 mmol, 1.0 equiv.) was added to a solution of 4-oxopentanal (0.020 g, 0.195 mmol, 1.0 equiv.) in DCM (2.5 mL) and allowed to react at r.t. for 18 hrs. The DCM was removed under reduced pressure and purified via column chromatography on silica gel (EtOAc / Cyclohexane (1:19)) to provide the title compound as a yellow oil (0.016 g, 46 % yield). **^{1}H NMR** (400 MHz, $CDCl_3$) δ_H 6.74 (1H, dt, J = 12.3, 5.4 Hz), 6.71-6.66 (1H, m), 6.64 (1H, dt, J = 12.3, 1.0 Hz), 2.59 (2H, t, J = 5.8 Hz), 2.50-2.45 (2H, m), 2.14 (3H, s), 1.86-1.84 (3H, m), 1.81-1.79 (3H, m); **^{13}C NMR** (101 MHz, $CDCl_3$) δ_C 207.1, 191.9, 144.7, 138.9, 137.7, 126.0, 41.9, 30.1, 26.6, 14.9, 11.5; **HRMS-ESI (m/z):** $C_{11}H_{17}O_2$ $[M+H]^+$ theoretical 181.1184, found 181.1160.

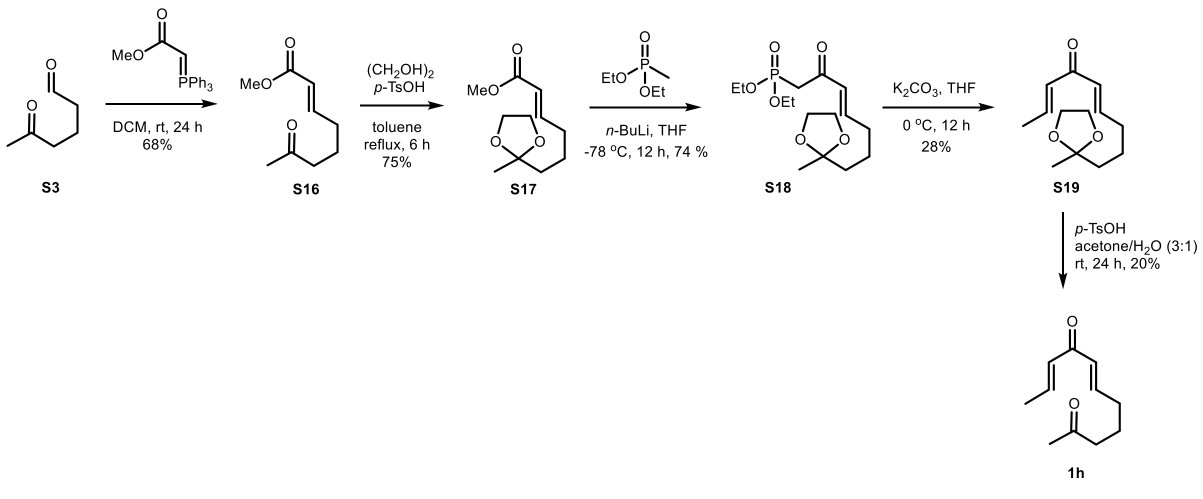

Scheme S6. *Synthesis of the bis-conjugated enone substrate **1h** starting from dicarbonyl **S3**.*

Methyl (2E)-7-oxooct-2-enoate (S16)[8]

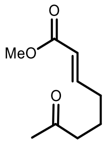

Methyl (triphenylphosphoranylidene)acetate (3.2 g, 9.60 mmol, 1.1 equiv.) was added to a solution of 5-oxohexanal (**S3**) (0.998 g, 8.73 mmol, 1.0 equiv.) in DCM (50 mL) and allowed to react at r.t. for 24 hrs. The DCM was removed under reduced pressure and purified via column chromatography on silica gel (EtOAc / Cyclohexane (1:4)) to provide the title compound as a colourless oil (1.01 g, 5.93 mmol, 68% yield). R.f. 0.42 (EtOAc/cyclohexane 1:1); 1H NMR (400 MHz, CDCl3): δ 6.91 (1H, dt, J = 15.6, 6.9 Hz, 3-H), 5.85-5.80 (1H, m, 2-H), 3.72 (3H, s, 1'-H), 2.45 (2H, t, J = 7.1 Hz, 6-H), 2.24-2.18 (2H, m, 4-H), 2.13 (3H, s, 8-H), 1.78-1.69 (2H, m, 5-H); 13C NMR (100 MHz, CDCl3): δ 208.1 (CO, C-7), 166.9 (CO, C-1), 148.3 (CH, C-3), 121.6 (CH, C-2), 51.4 (CH2, C-1'), 42.5 (CH2, C-6), 31.3 (CH2, C-4), 30.0 (CH3, C-8), 21.8 (CH2, C-5). The spectroscopic data was in accord with the previously reported data.[8]

Methyl (2E)-6-(2-methyl-1,3-dioxolan-2-yl)hex-2-enoate (S17)

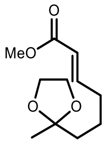

To compound 3g (1.0 eq, 5.29 mmol, 0.90 g) in toluene (50 mL) and pTsOH (0.10 eq, 0.53 mmol, 101 mg) and ethylene glycol (3.0 eq, 15.87 mmol, 0.89 mL) were added and the mixture was refluxed for a period of 6 h in a Dean-Stark apparatus, to remove water from the reaction mixture. The reaction mixture was then concentrated to remove the toluene and the resulting residue was diluted with Et2O (100 mL) and washed successively with sat. NaHCO3 solution, water, and brine (2 ⍰ 50 mL each). The organic layer was then dried over MgSO4, filtered, and concentrated under reduced pressure. The residue was purified by column chromatography on silica gel to provide the title compound as a colourless oil (760 mg, 75% yield). R.f. 0.31 (EtOAc/hexane 1:1); FTIR (neat) Vmax: 2984, 2949, 2876, 1720, 1656, 1436, 1376, 1270, 1197, 1171, 1039, 853 cm-1; 1H NMR (400 MHz, CDCl3): δ 6.95 (1H, dt, J = 15.7, 7.1 Hz, 3-H), 5.87-5.76 (1H, m, 2-H), 3.97-3.87 (4H,

m, 1''-H), 3.72 (3H, s, 1'-H), 2.26-2.17 (2H, m, 4-H), 1.68-1.62 (2H, m, 6-H), 1.60-1.52 (2H, m, 5-H), 1.30 (3H, s, 8-H); 13C NMR (100 MHz, CDCl3): δ 167.1 (CO, C-1), 149.2 (CH, C3), 121.1 (CH, C-2), 109.8 (CO, C-7), 64.7 (CH2, C-1''), 51.4 (CH2, C-1'), 38.5 (CH2, C-6), 32.2 (CH2, C-4), 23.8 (CH3, C-8), 22.4 (CH2, C-5); LC-MS (m/z): Calculated C11H19O4+ [M+H]+: 215.1278, found: 215.1272.

Diethyl [(3E)-7-(2-methyl-1,3-dioxolan-2-yl)-2-oxohept-3-en-1-yl]phosphonate (S18)

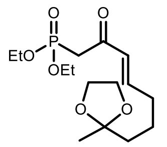

To methyl diethylphosphonate (1.0 eq, 1.78 mmol, 0.26 mL) in THF (10 mL) at –78 °C was added n-BuLi (1.2 eq, 2.13 mmol, 0.79 mL) and the mixture stirred for 15 mins. Compound 22b (1.0 eq, 1.78 mmol, 381 mg) in a solution of THF (2 mL) was then added slowly and the mixture allowed to stir for 12 h. The reaction mixture was then quenched with sat. NH4Cl (10 mL) and extracted with EtOAc (3 x 20 mL). The combined organic layers were washed with brine (20 mL), dried over MgSO4, filtered and concentrated under reduced pressure. The residue was purified by vacuum distillation and then eluted through a silica pad with MeOH/DCM (1:9) to provide the title compound as a pale yellow oil (384 mg, 74% yield). FTIR (neat) Vmax: 2981, 2939, 1730, 1665, 1625, 1376, 1247, 1018, 961, 856, 799 cm-1; 1H NMR (400 MHz, CDCl3): δ 6.97-6.86 (m, 1H), 6.27-6.18 (m, 1H), 4.19-4.00 (m, 4H), 3.99-3.85 (m, 4H), 3.19 (2H, d, J = 22.9 Hz, 1-H), 2.30-2.21 (2H, m, 5-H), 1.74-1.53 (4H, m, 6,7-H), 1.37-1.27 (9H, m); 13C NMR (101 MHz, CDCl3) δ 208.2, 191.3, 149.3 (d, J = 23.9 Hz), 130.5, 109.9, 64.8, 62.7, 42.6, 40.3 (d, J = 128.4 Hz), 31.8, 23.9, 22.0 (d, J = 19.7 Hz), 16.4 (t, J = 6.2 Hz); LC-MS (m/z): Calculated C15H28O6P+ [M+H]+: 335.1618, found: 335.1616.

(2E,5E)-9-(2-methyl-1,3-dioxolan-2-yl)nona-2,5-dien-4-one (S19)

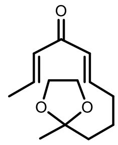

To compound 23b (1.0 eq, 3.11 mmol, 904 mg) dissolved in dry THF (10 mL) at 0 °C was added K2CO3 (5.0 eq, 15.57 mmol, 2.15 mg) and the mixture stirred for 15 mins.

Acetaldehyde (10 eq, 31.14 mmol, 1.74 mL) was then added and the mixture stirred for a further 12 h. The crude reaction mixture was filtered, concentrated under reduced pressure and purified by column chromatography on silica gel to provide the title compound as a colourless oil (195 mg, 28% yield). Rf 0.40 (EtOAc/hexane 1:1); FTIR (neat) Vmax: 2982, 2941, 2877, 1717, 1666, 1636, 1614, 1443, 1375, 1295, 1204, 1060, 976, 862 cm-1; 1H NMR (400 MHz, CDCl3): δ 7.01-6.78 (2H, m, 2,6-H), 6.43-6.22 (2H, m, 3,5-H), 4.03-3.82 (4H, m, 1',3'-H), 2.29-2.19 (2H, m, 7-H), 1.91 (3H, dd, J = 6.9, 1.4 Hz, 1-H), 1.70-1.62 (2H, m, 9-H), 1.62-1.52 (2H, m, 8-H), 1.30 (3H, s, 1''-H); 13C NMR (100 MHz, CDCl3): δ 189.4 (CO, C-4), 147.3 (CH, C-6), 143.0 (CH, C-2), 130.2 (CH, C-3), 128.8 (CH, C-5), 109.8 (C, C-2'), 64.6 (2CH2, C-1',3'), 38.6 (CH2, C-9), 32.6 (CH2, C-7), 23.8 (CH3, C-1''), 22.5 (CH2, C-8), 18.4 (CH3, C-1); LC-MS (m/z): Calculated C13H21O3+ [M+H]+: 225.1485, found: 225.1479.

(6*E*,9*E*)-undeca-6,9-diene-2,8-dione (1h)

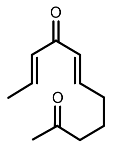

To 24a (1.0 eq, 0.83 mmol, 150 mg) dissolved in acetone:H2O (3:1, 8 mL) was added p-TsOH (0.05 eq, 0.042 mmol, 8.0 mg) and the mixture stirred for 24 h. The reaction mixture was diluted with brine (10 mL) and extracted with CH2Cl2 (3 x 10 mL). The combined organic layers were then washed with brine (10 mL), dried over MgSO4, filtered and concentrated under reduced pressure. The residue was purified by column chromatography on silica gel to provide the title compound as a colourless oil (30 mg, 20% yield). Rf 0.31 (EtOAc/hexane 1:1); FTIR (neat) Vmax: 2937, 1711, 1665, 1636, 1612, 1440, 1357, 1294, 1204, 1161, 979, 746 cm-1; 1H NMR (400 MHz, CDCl3): δ 6.96-6.78 (2H, m, 6,10- H), 6.38-6.26 (2H, m, 7,9-H), 2.45 (2H, t, J = 7.3 Hz, 3-H), 2.29-2.18 (2H, m, 5-H), 2.12 (3H, s, 1-H), 1.91 (3H, dd, J = 6.9, 1.8 Hz, 11-H), 1.81-1.68 (2H, m, 4-H); 13C NMR (101 MHz, CDCl3) δ 189.6, 147.5, 143.2, 130.4, 128.9, 109.9, 64.8, 38.8, 32.7, 23.9, 22.7, 18.5; LC-MS (m/z): Calculated C10H15O2+ [M+H]+: 181.1223, found: 181.1217

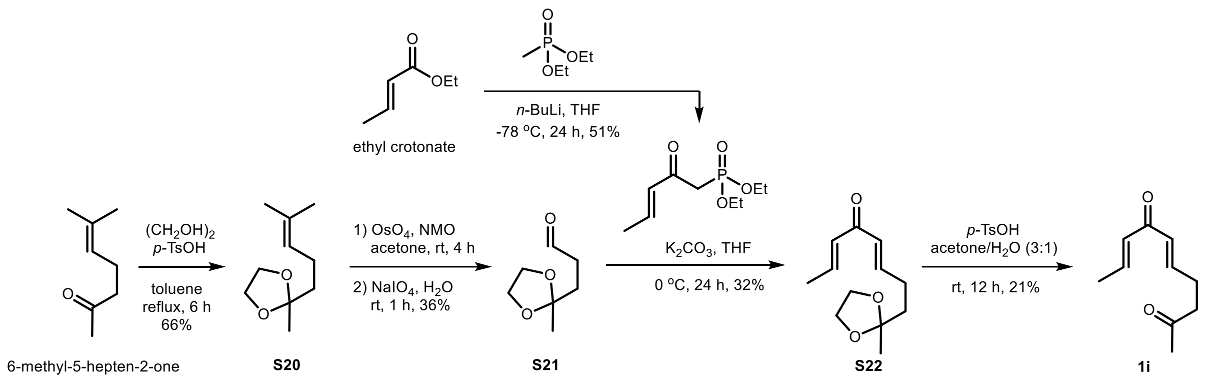

Scheme S7. *Synthesis of the bis-conjugated enone substrate **1i** starting from 6-methyl-5-hepten-2-one.*

2-methyl-2-(4-methylpent-3-en-1-yl)-1,3-dioxolane (S20)[9]

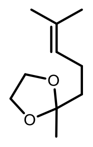

To 6-methyl-5-hepten-2-one (2.95 mL, 20 mmol) in toluene (20 mL), ethylene glycol (4.46 mL, 80 mmol) and p-TsOH (76 mg, 4.0 mmol) were added and the mixture was refluxed for a period of 6 h in a Dean-Stark apparatus, to remove water from the reaction mixture. The reaction mixture was then concentrated to remove the toluene and the resulting residue was diluted with Et2O (100 mL) and washed successively with sat. NaHCO3 solution, water and brine (2 x 50 mL each). The organic layer was then dried over MgSO4, filtered and concentrated under reduced pressure. The residue was purified by column chromatography on silica gel to provide the title compound as a colourless oil (2.25 g, 66% yield). R.f. 0.31 (Et2O/Hexane 1:9); 1H NMR (400 MHz, CDCl3) δ 5.10 (1H, s, 3'- H), 3.99-3.87 (4H, m, 1,3-H), 2.14-1.99 (2H, m, 2'-H), 1.73-1.58 (8H, m, 1',5',1'''-H), 1.32 (3H, s, 1''-H); 13C NMR (100 MHz, CDCl3): δ 131.8 (C, C-4'), 124.2 (CH, C-3'), 110.1 (C, C-2), 64.8 (CH2, C-1,3), 39.2 (CH2, C-1'), 25.8 (CH3), 23.9 (CH3), 22.9 (CH2, C-2'), 17.7 (CH3).). The spectroscopic data was in full accord with the previously reported data.[9]

3-(2-methyl-1,3-dioxolan-2-yl)propanal (S21)[10]

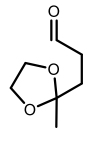

2-methyl-2-(4-methylpent-3-en-1-yl)-1,3-dioxolane (**26**) (1 eq, 16.6 mmol) and OsO4 (4% w.t/v tBuOH, 0.01 eq, 0.17 mmol, 1.06 mL) were added to a solution of N-methylmorpholine N-oxide

(2 eq, 33.2 mmol, 3.89 g) in acetone (20 mL). The mixture was stirred at room temperature for 4 h and then quenched with sat. aq. Na2SO3 solution (80 mL) and extracted with CH2Cl2 (3 ⍰ 60 mL). The combined organic extracts were dried over Na2SO4, filtered and concentrated under reduced pressure. The crude material was dissolved in water (20 mL) and sodium periodate (2 eq, 33.2 mmol, 7.10 g) was added. The resulting mixture was stirred for 1 h at room temperature and then diluted with water (60 mL) and extracted with CH2Cl2 (3 x 30 mL). Next, the combined organic extracts were dried over Na2SO4, filtered and concentrated under reduced pressure to provide **27**, (1.03 g, 36% yield) as a pale, yellow oil which weas used in the next step without further purification. 1H NMR (400 MHz, CDCl3) δ 9.71 (1H, s, 3'-H), 3.98-3.83 (4H, m, 1,3-H), 2.51-2.42 (2H, m, 2'-H), 2.06 (2H, t, J = 7.1 Hz, 1'-H), 1.33 (3H, s, 1''-H); 13C NMR (100 MHz, CDCl3): δ 202.3 (CO, C3'), 109.2 (C, C-2), 64.9 (CH2, C-1,3), 38.6 (CH2, C-2'), 31.9 (CH2, C-1'), 24.3 (CH3, C-1''). The spectroscopic data was in full accord with the previously reported data.[10]

Diethyl [(3E)-2-oxopent-3-en-1-yl]phosphonate[11]

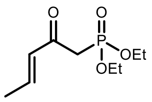

To diethyl methylphosphonate (1.0 eq, 20 mmol, 2.92 mL) in THF (80 mL) at -78 °C was added n-BuLi (1.2 eq, 24 mmol, 2.5 M, 9.60 mL) and the mixture stirred for 30 min. Ethyl crotonate (1.2 eq, 24 mmol, 2.98 mL) in THF (10 mL) was then added slowly and the mixture stirred for 24 h at -78 °C. The reaction mixture was then quenched with sat. NH4Cl and extracted with EtOAc (3 x 50 mL). The combined organic layers were washed with brine (100 mL), dried over MgSO4, filtered, and concentrated under reduced pressure. The residue was purified by vacuum distillation to provide the title compound as a yellow oil (2.23 g, 51% yield). 1H NMR (400 MHz, CDCl3): δ 7.06-6.86 (1H, m, 4-H), 6.34-6.16 (1H, m, 3-H), 4.26-3.96 (4H, m, 1'-H), 3.19 (2H, d, J = 22.9 Hz, 1-H), 1.97-1.90 (3H, m, 5-H), 1.36-1.27 (6H, m, 2'-H). The spectroscopic data was in full accord with the previously reported data.[11]

(2E,5E)-8-(2-methyl-1,3-dioxolan-2-yl)octa-2,5-dien-4-one (S22)

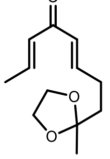

To a solution of **28** (2.0 eq, 2.0 mmol, 304 mg) and K2CO3 (5.0 eq, 5.0 mmol, 691 mg) in dry THF (20 mL) was added 27 (1.0 eq, 1.0 mmol 144 mg) and the mixture stirred at 0 °C for 24 h. The

reaction mixture was then filtered, concentrated under reduced pressure and the residue was purified by column chromatography on silica gel to provide the title compound as a colourless oil (68 mg, 32% yield). FTIR (neat) Vmax: 2936, 1713, 1665, 1376, 1163, 1042, 973, 861 731 cm-1; 1H NMR (400 MHz, CDCl3) δ 6.98-6.82 (2H, m, 2,6-H), 6.38-6.27 (2H, m, 3,5-H), 3.99-3.85 (4H, m, 1',3'-H), 2.38-2.28 (2H, m, 7-H), 1.91 (3H, dd, J = 6.9, 1.4 Hz, 1'-H), 1.85-1.77 (2H, m, 8-H), 1.33 (3H, s, 1''-H); 13C NMR (100 MHz, CDCl3): δ 189.4 (CO, C-4), 147.4 (CH, C-6), 143.0 (CH, C-2), 130.2 (CH, C-3), 128.4 (CH, C-5), 109.4 (C, C-2'), 64.7 (2CH2, C-1',3'), 37.4 (CH2, C-8), 27.2 (CH2, C-7), 24.0 (CH3, C-1''), 18.4 (CH3, C-1); LC-MS (m/z): Calculated $C12H19O3^+$ $[M+H]^+$: 211.1329, found: 211.1326.

(5E,8E)-deca-5,8-diene-2,7-dione (1i)

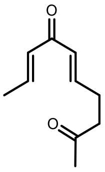

To a solution of **24b** (1 eq, 0.32 mmol, 68 mg) in acetone:H2O (3:1, 4 mL) was added p-TsOH (0.05 eq, 0.016 mmol, 3 mg) and the mixture was stirred at r.t. for 12 h. The resulting mixture was diluted with brine (5 mL) and extracted with EtOAc (3 x 10 mL). The combined organic layers were then washed with brine (20 mL), dried over MgSO4, filtered and concentrated under reduced pressure. The crude material was purified by column chromatography on silica gel to provide the title compound as a colourless oil (11 mg, 21% yield). R.f. 0.28 (EtOAc/hexane 1:1); FTIR (neat) Vmax: 2913, 1712, 1665, 1637, 1613, 1441, 1358, 1288, 1161, 977, 868 cm-1; 1H NMR (400 MHz, CDCl3): δ 7.00-6.73 (2H, m, 5,9-H), 6.40-6.22 (2H, m, 6,8-H), 2.65-2.56 (2H, m, 3-H), 2.52-2.44 (2H, m, 4-H), 2.15 (3H, s, 1-H), 1.90 (3H, dd, J = 6.9, 1.4 Hz, 10-H); 13C NMR (100 MHz, CDCl3): δ 206.9 (CO, C-2), 189.1 (CO, C-7), 145.4 (CH, C-5), 143.3 (CH, C-9), 130.2 (CH, C-8), 129.1 (CH, C-6), 41.6 (CH2, C-3), 29.9 (CH3, C-1), 26.3 (CH2, C-4), 18.4 (CH3, C-10); LCMS (m/z): Calculated $C10H15O2^+$ $[M+H]^+$: 167.1067, found: 161.1060

1.3. Optimisation of biotransformation conditions

Following the successful synthesis of target ketoenones, substrate **1a** was selected as a model substrate for optimisation of the biotransformation conditions due to its promising early results and the added complication of incomplete cyclisation, resulting in the co-formation of **3a**. Although having co-products in the model experiments may not seem a logical choice initially, the rationale was to determine if the conditions could be

optimised to push the second aza-Michael reaction towards the formation of the quinolizidine target structures **4a** (Scheme S8).

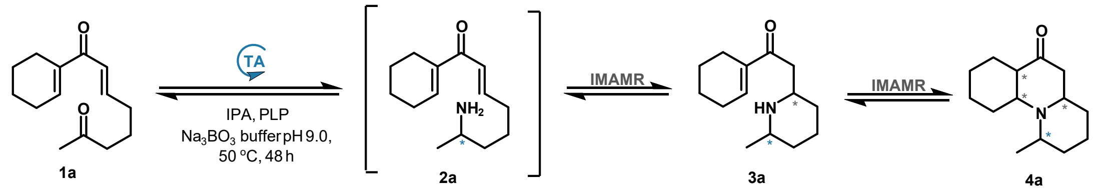

Scheme S8: *The DIMAMR methodology carried out on substrate* ***1a,*** *which was used as the model reaction for optimisation of biotransformation conditions.*

Table S1: Optimisation of reaction conditions.

Entry	TA	Buffer	Temp. (°C)	Time (h)	pH	IPA (equiv.)	Conv. (%)*	Iso. Yield (%)**
**1**	ATA-113	HEPES	30	48	7.5	2	0	-
**2**	ATA-117	HEPES	30	48	7.5	2	0	-
**3**	ATA-256	HEPES	30	24	7.5	4	0	-
**4**	ATA-025	HEPES	30	24	7.5	4	0	-
**5**	ATA-256	HEPES	30	24	7.5	20	0	-
**6**	ATA-025	HEPES	30	24	7.5	20	0	-
**7**	ATA-256	HEPES	50	24	7.5	4	>99.9	69
**8**	ATA-025	HEPES	50	24	7.5	4	>99.9	52
**9**	ATA-256	HEPES	60	24	7.5	4	>99.9	20
**10**	ATA-256	HEPES	50	2	7.5	4	99	-
**11**	ATA-025	HEPES	50	2	7.5	4	37	-
**12**	ATA-256	HEPES	50	4	7.5	4	99	-
**13**	ATA-025	HEPES	50	4	7.5	4	92	-
**14**	ATA-256	HEPES	50	6	7.5	4	99	-
**15**	ATA-025	HEPES	50	6	7.5	4	93	-
**16**	ATA-256	HEPES	50	48	7.5	4	>99.9	72
**17**	ATA-025	HEPES	50	48	7.5	4	>99.9	57
**18**	ATA-256	HEPES	50	48	8.0	4	>99.9	65
**19**	ATA-256	HEPES	50	48	9.0	4	>99.9	72

**20**	ATA-256	HEPES	50	48	10.0	4	>99.9	70
**21**	ATA-256	Na_3BO_3	50	48	9.0	4	>99.9	75
**22**	ATA-025	Na_3BO_3	50	48	9.0	4	>99.9	70
**23**	ATA-256	Na_3BO_3	50	72	9.0	4	>99.9	73

Highlighted optimised reaction conditions were used for all subsequent reactions when testing the methodology.

*By disappearance of starting substrate **1a** monitored using quantitative ^{1}H NMR.

** As a crude product mixture (both **3a** and **4a**).

For compounds **4a** and **4c** four diastereoisomers were observed in the crude reaction mixture extract. Upon attempted purification on silica (acidic) or basic alumina (pH 9.0) a mixture of three diastereoisomers were obtained. Although the R_f values of the isomers were distinct, the isomers could not readily be separated on silica or basic alumina due to observed interconversion. The same conversion (epimerisation) from four isomers to three isomers was observed when the crude reaction mixture was stirred in MeOH with potassium carbonate or with HCl at room temp. for 1 h. Therefore, compounds **4a** and **4c** were purified by column chromatography on neutral alumina.

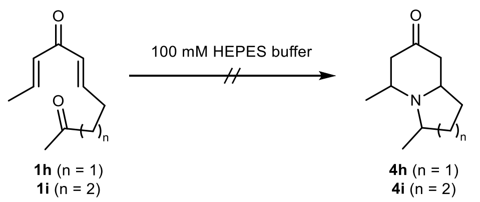

Scheme S9: *Amination of substrates **1h** and **1i** was attempted under conditions presented in table S2.*

Table S2: Reaction conditions for the attempted amination of 1h and 1i

Entry	TA	Buffer	Temp. (°C)	Time (h)	pH	IPA (equiv.)	Conv.*
**1**	ATA-113	HEPES	30	24	7.5	2	decomp
**2**	ATA-117	HEPES	30	24	7.5	2	decomp
**3**	ATA-256	HEPES	50	24	7.5	2	decomp
**4**	ATA-025	HEPES	50	24	7.5	2	decomp
**5**	ATA-117	HEPES	30	24	7.5	20	decomp
**6**	ATA-256	HEPES	50	24	7.0	2	decomp
**7**	ATA-256	HEPES	50	24	9.0	2	decomp
**8**	-	HEPES	30	24	7.5	2	decomp

*No starting material or desired product was detected in any of the attempted biotransformations, including the no-enzyme control reaction. Decomposition products could not be isolated, and we suspect that this is due to formation of decomposition products that are either volatile or do not extract well from the aqueous buffer.

2. DIMAMR biotransformations

General procedure for biotransformations towards single and double IMAMR products

Commercially available (*S*)-selective ATA256 (50 mg) or (*R*)-selective ATA-025 (50 mg) was rehydrated in Na_3BO_3 buffer (8.5 mL, 100 mM, pH 9.0) containing PLP (2 mM) and isopropylamine (4 eq, 200 mM). The pH of the mixture was readjusted to 9.0, using aq. NaOH solution (1 M), and the total volume of the reaction was adjusted to 9 mL by addition of Na_3BO_3 buffer. The ketoenone substrate was added (50 mM, 1.0 mL from a 500 mM stock solution in DMSO) and the reaction mixture incubated at 50 ^{o}C, 220 rpm for 48 h. The reaction was basified to pH 10 and extracted using Et_2O (3 x 5 ml). The combined organic extracts were dried over $MgSO_4$, filtered and concentrated *in vacuo*.

The crude material was purified by flash chromatography on neutral alumina using DCM. All isolated compounds were analysed by NMR.

1-(Cyclohex-1-en-1-yl)-2-[(2*S*, 6*S*)-6-methylpiperidin-2-yl]ethan-1-one ((*S*,*S*)-3a)

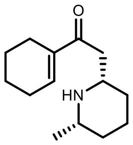

Yellow oil (0.062 g, 13 % yield). **^{1}H NMR** (400 MHz, $CDCl_3$) δ_H 6.92-6.89 (1H, m), 3.08-3.01 (1H, m), 2.90 (1H, br s), 2.77-2.73 (2H, m), 2.72-2.68 (1H, m), 2.25 (4H, m), 1.78-1.72 (1H, m), 1.64-1.51 (6H, m), 1.43-1.32 (1H, m), 1.24-1.09 (2H, m), 1.06 (3H, d, *J* = 5.0 Hz); **^{13}C NMR** (101 MHz, $CDCl_3$) δ_C 200.5, 140.6, 139.4, 53.2, 52.3, 43.6, 33.6, 31.7, 26.1, 24.5, 22.9, 22.7, 21.9, 21.5; **HRMS-ESI (m/z):** $C_{14}H_{24}NO^+$ $[M+H]^+$ theoretical 222.1813, found 222.1853; $[\alpha]_D^{20}$ = +40.0 (c=1.0, CH_3Cl); (*R*,*R*)-**3a**: $[\alpha]_D^{20}$ = -40.0 (c=1.0, CH_3Cl). The spectroscopic data for (*R*,*R*)-**3a** well in full accord with the reported data for (*S*,*S*)-**3a**.

1-(Cyclohex-1-en-1-yl)-2-((2*S*,6*S*)-6-ethylpiperidin-2-yl)ethan-1-one ((*S,S*)-3b)

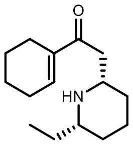

Yellow oil (0.017 g, 40 % yield). **^{1}H NMR** (400 MHz, $CDCl_3$) δ_H 6.92-6.88 (1H, m), 3.03-2.95 (1H, m), 2.72 (1H, s), 2.70 (1H, d, *J* = 1.89 Hz), 2.48-2.40 (1H, m), 2.25-2.17 (4H, m), 1.79-1.72 (1H, m), 1.69-1.50 (6H, m), 1.41-1.29 (3H, m), 1.16 (1H, tdd, *J* = 12.84, 11.12, 3.87 Hz), 1.00 (1H, tdd, *J* = 12.87, 10.95, 3.90 Hz), 0.89 (3H, t, *J* = 7.5 Hz); **^{13}C NMR** (101 MHz, $CDCl_3$) δ_C 200.8, 140.5, 139.6, 58.5, 53.3, 44.0, 32.7, 31.8, 30.1, 26.2, 24.7, 23.1, 22.0, 21.6, 10.5; **HRMS-ESI (m/z):** $C_{15}H_{26}NO^+$ $[M+H]^+$ theoretical 236.1970, found 236.2011.

(*E*)-4-(3,4-Dimethoxyphenyl)-1-((2*S*,6*S*)-6-methylpiperidin-2-yl)but-3-en-2-one ((*S*,*S*)-3e)

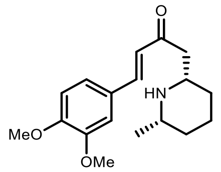

Yellow oil (0.045g, 36 %). **^{1}H NMR** (400 MHz, $CDCl_3$) δ_H 7.50 (1H, d, *J* = 12.9 Hz), 7.10 (1H, dd, *J* = 6.3, 1.9 Hz), 7.04 (1H, d, *J* = 1.9 Hz), 6.86 (1H, d, *J* = 6.8 Hz), 6.59 (1H, d, *J* = 13.0 Hz), 3.90 (6H, s), 3.17 (1H, m), 2.84 (3H, m), 1.79 (1H, m), 1.64 (2H, m), 1.42 (1H, qt, *J* = 12.9, 3.6 Hz), 1.25 (2H, m), 1.12 (3H, d, *J* = 6.30 Hz); **^{13}C NMR** (101 MHz, $CDCl_3$) δ_C 199.3, 151.6, 149.4, 143.5, 127.3, 124.6, 123.3, 111.2, 109.8, 56.1, 56.0, 53.5, 52.6, 46.8, 33.5, 31.6, 24.5, 22.7; **HRMS-ESI (m/z):** $C_{18}H_{26}NO_3^+$ $[M+H]^+$ theoretical 304.1868, found 304.1909. $[\alpha]_D^{22}$ = -10.0 (c=0.3, CH_2Cl_2); (*R*,*R*)-**3e**: $[\alpha]_D^{22}$ = +10.0 (c=0.3, CH_2Cl_2). The spectroscopic data for (*R*,*R*)-**3e** well in full accord with the reported data for (*S*,*S*)-**3e**.

(1*S*,4a*R*,6a*R*,10a*S*)-1-Methyldodecahydro-6H-pyrido[1,2-a]quinolin-6-one (1*S*,4a*R*,6a*R*,10a*S*-4a)

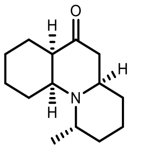

Yellow oil (0.007 g, 7 % yield). **^{1}H NMR** (400 MHz, $CDCl_3$) δ_H 3.64-3.58 (1H, m), 3.47-3.42 (1H, m), 3.06-2.98 (1H, m), 2.75 (1H, t, *J* = 11.00 Hz), 2.72-2.69 (1H, m), 2.33-2.28 (1H, m), 1.94 (1H, dd, *J* = 11.29, 2.53 Hz), 1.89-1.81 (1H, m), 1.80-1.75 (1H, m), 1.72-1.66 (2H, m), 1.57-1.53 (2H, m), 1.47-1.35 (4H, m), 1.32-1.21 (2H, m), 1.16 (3H, d, J = 4.88 Hz), 1.13-1.07 (1H, m); **^{13}C NMR** (101 MHz, $CDCl_3$) δ_C 211.3, 59.6, 53.5, 52.3, 47.4, 44.0, 41.5, 35.4, 30.1, 26.5, 24.5, 22.2, 21.3, 19.4; **HRMS-ESI (m/z):** $C_{14}H_{24}NO^+$ $[M+H]^+$ theoretical 222.1813, found 222.1851.

(1*S*,4a*S*,6a*S*,10a*S*)-1-Methyldodecahydro-6H-pyrido[1,2-a]quinolin-6-one (1*S*,4a*S*,6a*S*,10a*S* -4a)

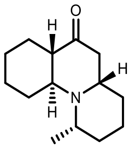

Yellow oil (0.008 g, 8 % yield, increased from NMR yield due to interconversion). **^{1}H NMR** (400 MHz, $CDCl_3$) δ_H 3.04-3.00 (1H, m), 2.99-2.95 (1H, m), 2.95-2.91 (1H, m), 2.57-2.50 (1H, m), 2.35-2.30 (1H, m), 2.29 (1H, d, *J* = 2.29 Hz), 2.25 (1H, d, *J* = 9.11 Hz), 1.78-1.72 (1H, m), 1.69-1.66 (1H, m), 1.65-1.62 (1H, m), 1.62-1.58 (1H, m), 1.58-1.54 (1H, m), 1.46-1.40 (1H, m), 1.33-1.26 (3H, m), 1.23-1.17 (3H, m), 1.15 (3H, d, *J* = 4.90 Hz), 1.02 (1H, ap. q, *J* = 10.15 Hz); **^{13}C NMR** (101 MHz, $CDCl_3$) δ_C 212.7, 58.8, 58.6, 55.6, 47.0, 46.2, 38.1, 36.1, 33.6, 25.5, 24.5, 24.4, 22.1, 21.7; **HRMS-ESI (m/z):** $C_{14}H_{24}NO^+$ $[M+H]^+$ theoretical 222.1813, found 222.1851.

(1*S*,4a*R*,6a*S*,10a*S*)-1-Methyldodecahydro-6H-pyrido[1,2-a]quinolin-6-one (1*S*,4a*R*,6a*S*,10a*S*-4a)

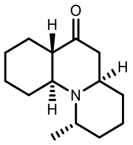

Yellow oil (0.003 g, 3 % yield). **^{1}H NMR** (400 MHz, $CDCl_3$) δ_H 3.58-3.51 (1H, m), 2.69 (1H, tt, *J* = 8.36, 2.48 Hz), 2.29-2.20 (5H, m), 1.96-1.90 (1H, m), 1.82-1.72 (3H, m), 1.66-1.61 (2H, m), 1.57-1.49 (2H, m), 1.40-1.32 (1H, m), 1.26-1.63 (4H, m), 0.91 (3H, d, *J* = 5.33 Hz); **^{13}C NMR** (101 MHz, $CDCl_3$) δ_C 210.0, 63.8, 54.7, 54.1, 49.6, 47.8, 35.5, 32.4, 30.2, 25.2, 24.7, 24.3, 18.3, 8.5; **HRMS-ESI (m/z):** $C_{14}H_{24}NO^+$ $[M+H]^+$ theoretical 222.1813, found 222.1852; $[\alpha]_D^{22}$ = -3.8 (c=0.4, CH_2Cl_2).

(1*S*,3a*R*,5a*S*,9a*S*)-1-Methyldecahydropyrrolo[1,2-a]quinolin-5(1H)-one (1*S*,3a*R*,5a*S*,9a*S*-4c)

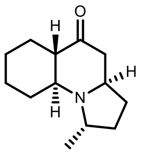

Yellow oil (0.015 g, 15 %). **^{1}H NMR** (500 MHz, $CDCl_3$) δ_H 3.63 (1H, quin, *J* = 6.7 Hz), 2.87-2.80 (1H, m), 2.49 (1H, dd, *J* = 12.9, 3.2 Hz), 2.33 – 2.26 (2H, m), 2.20 – 2.10 (3H, m), 2.04 – 1.94 (2H, m), 1.77 (1H, d, *J* = 10.7 Hz), 1.55 – 1.49 (1H, m), 1.46 – 1.41 (1H, m), 1.39 – 1.30 (1H, m), 1.28 – 1.12 (4H, m), 0.89 (3H, d, *J* = 6.5 Hz); **^{13}C NMR** (101 MHz, $CDCl_3$) δ_C 210.5, 60.8, 58.8, 53.8, 52.6, 48.2, 31.9, 30.3, 29.9, 25.2, 24.9, 23.9, 13.5; **HRMS-ESI (m/z):** $C_{13}H_{22}NO^+$ $[M+H]^+$ theoretical 208.1657, found 208.1697. $[\alpha]_D^{20}$ = +2.5 (c=2.2, CH_3Cl); (1*R*,3a*S*,5a*R*,9a*R*)-**4c**: $[\alpha]_D^{20}$ = -2.5 (c=2.2, CH_3Cl). The spectroscopic data for (1*R*,3a*S*,5a*R*,9a*R*)-**4c** well in full accord with the reported data for 1*S*,3a*R*,5a*S*,9a*S*)-**4c**.

(1*S*,3a*S*,5a*R*,9a*S*)-1-Methyldecahydropyrrolo[1,2-a]quinolin-5(1H)-one (1*S*,3a*S*,5a*R*,9a*S*-4c)

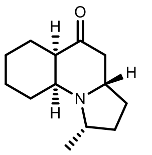

Yellow oil as a mixture with (1*S*,3a*S*,5a*S*,9a*S*)-**4c** (0.024 g, 23 %). **^{1}H NMR** (400 MHz, $CDCl_3$) δ_H 3.34 (1H, m), 2.94 (1H, m), 2.84 (1H, m), 2.75 (1H, m), 2.44 (1H, dd, *J* = 13.66, 3.34 Hz), 2.31 – 2.23 (2H, m), 1.96-1.65 (4H, m), 1.51 – 1.39 (3H, m), 1.39 – 1.18 (3H, m), 1.14 (3H, d, *J* = 6.16 Hz), 1.04 (1H, qd, *J* = 12.49, 3.08 Hz); **^{13}C NMR** (101 MHz, $CDCl_3$) δ_C 210.8, 56.7, 55.7, 52.4, 49.7, 47.9, 31.1, 29.7, 25.0, 24.9, 22.5, 19.9, 18.5; **HRMS-ESI (m/z):** $C_{13}H_{22}NO^+$ $[M+H]^+$ theoretical 208.1657, found 208.1696.

(1*S*,3a*S*,5a*S*,9a*S*)-1-Methyldecahydropyrrolo[1,2-a]quinolin-5(1H)-one (1*S*,3a*S*,5a*S*,9a*S*-4c)

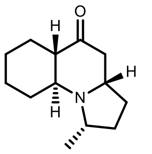

Yellow oil as a mixture with (1*S*,3a*S*,5a*R*,9a*S*)-**4c** (0.024 g, 23 %). ^{1}**H NMR** (400 MHz, $CDCl_3$) δ_H 3.74-3.68 (1H, m), 3.31-3.23 (2H, m), 2.80-2.75 (1H, m), 2.31-2.25 (2H, m), 2.23-2.17 (2H, m), 2.18-2.07 (2H, m), 1.80-1.74 (1H, m), 1.70-1.65 (1H, m), 1.56-1.42 (3H, m), 3.35-1.21 (3H, m), 1.16 (3H, d, *J* = 5.96 Hz); ^{13}**C NMR** (101 MHz, $CDCl_3$) δ_C 210.8, 58.9, 58.3, 52.7, 44.6, 44.0, 31.6, 30.6, 29.1, 26.2, 24.4, 21.7, 20.8; **HRMS-ESI (m/z):** $C_{13}H_{22}NO^+$ $[M+H]^+$ theoretical 208.1657, found 208.1696.

(4*R*,6*S*,9a*R*)-4-(3,4-Dimethoxyphenyl)-6-methyloctahydro-2H-quinolizin-2-one (4*R*,6*S*,9a*R*-4e)

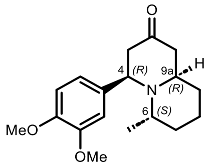

Yellow oil (0.018 g, 14 %). ^{1}**H NMR** (400 MHz, $CDCl_3$) δ_H 6.95 (1H, d, *J* = 1.86 Hz) , 6.86 (1H, dd, *J* = 8.10, 1.95 Hz), 6.81 (1H, d, *J* = 8.2 Hz), 3.90 (3H, s), 3.87 (3H, s), 3.60 (1H, dd, *J* = 11.8, 3.1 Hz), 3.04 (1H, m), 2.84 (1H, m), 2.61 (1H, m), 2.42-2.29 (3H, m), 1.75-1.64 (2H, m), 1.54-1.37 (4H, m), 0.83 (3H, d, *J* = 6.66 Hz); ^{13}**C NMR** (101 MHz, $CDCl_3$) δ_C 208.3, 134.9, 132.1, 132.0, 119.6, 111.1, 110.0, 56.1, 56.0, 53.7, 49.6, 49.0, 41.0, 35.3, 32.2, 29.8, 18.3, 8.3; **HRMS-ESI (m/z):** $C_{18}H_{26}NO_3^+$ $[M+H]^+$ theoretical 304.1868, found 304.1885. $[\alpha]_D^{22}$ = +62.5 (c=0.1, CH_2Cl_2); (4*S*,6*R*,9a*S*)-**4e**: $[\alpha]_D^{22}$ = -62.0 (c=0.1, CH_2Cl_2). The spectroscopic data for (4*R*,6*S*,9a*R*)-**4e** well in full accord with the reported data for (4*R*,6*S*,9a*R*)-**4e**.

(3*S*,5*R*,8a*R*)-5-(3,4-Dimethoxyphenyl)-3-methylhexahydroindolizin-7-one (3*S*,5*R*,8a*R*-4f)

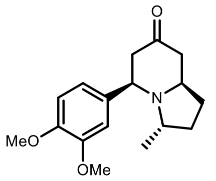

Yellow oil (0.040 g, 31 %). **^{1}H NMR** (400 MHz, $CDCl_3$) δ_H 6.96 (1H, d, *J* = 1.30 Hz, Ar*H*), 6.89 (1H, dd, *J* = 5.45, 1.30 Hz, Ar*H*), 6.82 (1H, d, *J* = 5.44 Hz, Ar*H*), 3.89 (3H, s, -OMe), 3.87 (3H, s, -OMe), 3.66 (1H, dd, *J* = 7.26, 2.48 Hz), 3.33 (1H, m), 2.94 (1H, m), 2.56 (1H, dt, *J* = 8.87, 1.77 Hz), 2.43 (3H, m), 2.10 (2H, m), 1.60 (1H, m), 1.37 (1H, m), 0.74 (3H, d, *J* = 4.36); **^{13}C NMR** (101 MHz, $CDCl_3$) δ_C 209.2, 149.4, 148.4, 134.9, 119.6, 111.1, 110.3, 60.9, 58.1, 56.2, 56.0, 53.5, 51.1, 48.5, 30.5, 30.3, 14.0; **HRMS-ESI (m/z):** $C_{17}H_{24}NO_3^+$ $[M+H]^+$ theoretical 290.1711, found 290.1750.

(3*S*,5*S*,8a*S*)-5-(3,4-Dimethoxyphenyl)-3-methylhexahydroindolizin-7-one (3*S*,5*S*,8a*S*-4f)

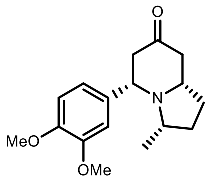

Yellow oil (0.037g, 29 %). **^{1}H NMR** (400 MHz, $CDCl_3$) δ_H 6.90 (1H, d, *J* = 1.35 Hz, Ar*H*), 6.83 (1H, dd, *J* = 5.43, 1.28 Hz, Ar*H*), 6.77 (1H, d, *J* = 5.42 Hz, Ar*H*), 3.87 (3H, s, -OMe), 3.85 (3H, s, -OMe), 3.33 (1H, dd, *J* = 7.50, 2.42 Hz), 2.70 (1H, m), 2.52 (1H, m), 2.49 (3H, m), 2.41 (1H, m), 1.98 (1H, dq, *J* = 8.50, 5.98 Hz), 1.80 (1H, m), 1.57 (1H, m), 1.39 (1H, m), 0.44 (3H, d, *J* = 4.05 Hz); **^{13}C NMR** (101 MHz, $CDCl_3$) δ_C 208.8, 149.0, 148.8, 135.3, 120.4, 111.1, 110.8, 67.7, 66.5, 59.5, 56.1, 55.9, 50.0, 47.4, 32.8, 29.6, 23.0; **HRMS-ESI (m/z):** $C_{17}H_{24}NO_3^+$ $[M+H]^+$ theoretical 290.1711, found 290.1750.

(3*S*,5*R*,8a*S*)-5-(3,4-Dimethoxyphenyl)-3-methylhexahydroindolizin-7-one (3*S*,5*R*,8a*S*-4f)

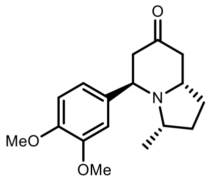

Yellow oil (0.020g, 16 %). **^{1}H NMR** (400 MHz, $CDCl_3$) δ_H 6.78 (1H, d, *J* = 5.47 Hz), 6.58 (1H, dd, *J* = 5.49, 1.37 Hz), 6.53 (1H, d, *J* = 1.34 Hz), 4.48 (1H, dd, *J* = 4.77, 0.78 Hz), 3.84 (3H, s), 3.81 (3H, s), 2.94 (1H, dd, J = 10.34, 4.86 Hz), 2.82-2.77 (1H, m), 2.67-2.61 (2H, m), 2.41-2.36 (2H, m), 1.83-1.71 (2H, m), 1.47-1.40 (2H, m), 1.25 (3H, d, *J* = 3.97 Hz); **^{13}C NMR** (101 MHz, $CDCl_3$) δ_C 211.1, 148.5, 148.4, 129.6, 121.5, 120.4, 112.4, 110.6, 56.2, 55.9, 54.9, 53.3, 48.6, 46.0, 30.9, 29.5, 18.7; **HRMS-ESI (m/z):** $C_{17}H_{24}NO_3^+$ $[M+H]^+$ theoretical 290.1711, found 290.1750.

2.1. One-pot two enzyme biotransformations

General procedure for the one-pot two enzyme biotransformations towards double aminated indolizidine- or quinolizidine-containing structures.

(*S*)-selective ATA256 (50 mg) and ATA-234 (50 mg) were rehydrated in Na_3BO_3 buffer (8 mL, 25 mM, pH 9.0) containing PLP (1 mM) and isopropylamine (4 eq, 80 mM). The pH of the mixture was adjusted to 9.0, using aq. NaOH solution (1 M), and the total volume of the reaction was adjusted to 9 mL by addition of Na_3BO_3 buffer.

The ketoenone substrate was added (20 mM, 1.0 mL from a 200 mM stock solution in MeOH) and the reaction mixture incubated at 50 °C, 220 rpm for 48 h. The reaction was basified to pH 10 and extracted using Et_2O (3 x 5 ml). The combined organic extracts were dried over $MgSO_4$, filtered and concentrated *in vacuo*. The crude material was purified by flash chromatography on neutral alumina (100% DCM to 0.5% MeOH), and the acid salt was formed from the product fractions using HCl (2M in diethyl ether).

(1*S*,3a*R*,5*S*,5a*R*,9a*S*)-1-methyldodecahydropyrrolo[1,2-a]quinolin-5-amine (5a)

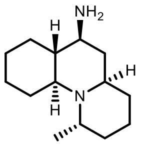

White crystalline salt (0.004 g, 10 % yield). **^{1}H NMR** (400 MHz, $CDCl_3$) δ_H 3.97-3.89 (1H, m), 3.39 (1H, tt, *J* = 11.9, 3.1 Hz), 3.19 (1H, td, *J* = 11.6, 3.4 Hz), 3.03 (1H, td, *J* = 10.8, 4.0 Hz), 2.24-2.17 (2H, m), 1.95-1.36 (13H, m), 1.19 (3H, d, *J* = 7.0 Hz), 1.16-1.00 (2H, m); **^{13}C NMR** (101 MHz, $CDCl_3$) δ_C 62.1, 54.5, 52.3, 49.9, 42.6, 34.8, 31.0, 29.2, 27.3, 25.8, 23.7, 23.2, 15.5, 9.4; **HRMS-ESI (m/z):** $C_{14}H_{27}N_2^+$ $[M+H]^+$ theoretical 223.2130, found 223.2153. $[\alpha]_D^{22}$ = +42.5 (c=0.2, H_2O)

(1*S*,3a*R*,5*R*,5a*R*,9a*S*)-1-Methyldodecahydropyrrolo[1,2-a]quinolin-5-amine ((5*R*)-5c)

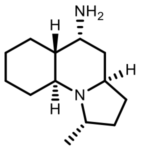

White crystalline salt (0.004 g, 9 % yield). **^{1}H NMR** (400 MHz, $CDCl_3$) δ_H 4.14 (1H, quin, *J* = 7.1 Hz), 3.78-3.74 (1H, m), 3.65-3.55 (1H, m), 3.29 (1H, td, *J* = 11.1, 3.7 Hz), 2.56-2.49 (1H, m), 2.46-2.37 (1H, m), 2.35-2.24 (2H, m), 2.17-2.02 (2H, m), 1.95-1.83 (4H, m), 1.79-1.69 (1H, m), 1.50-1.36 (3H, m), 1.33 (3H, d, *J* = 6.92 Hz), 1.31-1.23 (1H, m); **^{13}C NMR** (101 MHz, $CDCl_3$) δ_C 57.2, 56.0, 55.1, 48.2, 40.8, 31.2, 28.0, 27.2, 27.2, 26.5, 24.3, 23.5, 13.7; **HRMS-ESI (m/z):** $C_{13}H_{25}N_2^+$ $[M+H]^+$ theoretical 209.1973, found 209.2014. $[\alpha]_D^{22}$ = -15.0 (c=0.2, H_2O)

(1*S*,3a*R*,5*S*,5a*R*,9a*S*)-1-Methyldodecahydropyrrolo[1,2-a]quinolin-5-amine ((5*S*)-5c)

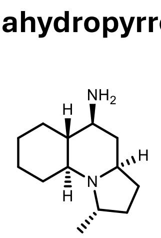

White crystalline salt (0.008 g, 19 % yield). **^{1}H NMR** (400 MHz, $CDCl_3$) δ_H 4.12 (1H, quin, *J* = 7.08 Hz), 3.66 (1H, m), 3.37 (1H, td, *J* = 11.3, 3.9 Hz), 3.19-3.12 (1H, m), 2.64 (1H, dt, *J* = 13.7, 3.3 Hz), 2.45-2.21 (3H, m), 2.10-2.03 (1H, m), 1.95-1.69 (6H, m), 1.53-1.35 (3H, m), 1.29 (3H, d, J = 6.9 Hz), 1.27-1.17 (1H, m); **^{13}C NMR** (101 MHz, $CDCl_3$) δ_C 59.1, 58.8, 56.9,

50.5, 42.9, 32.2, 27.5, 27.3, 26.6, 26.1, 23.6, 23.3, 13.5; **HRMS-ESI (m/z):** $C_{13}H_{25}N_2^+$ $[M+H]^+$ theoretical 209.1973, found 209.2014. $[\alpha]_D^{22}$ = +27.5 (c=0.2, H_2O)

3. Additional selectivity

The apparent additional selectivity observed in many of the reactions leads to the formation of a reduced number of diastereoisomers. To rationalise this trend, the hypothesised transition states of the second IMAMR for one substrate, **1c**, will be discussed in detail. After transamination of the ketoenone **1c**, a reactive nitrogen nucleophile is stereoselectively generated in situ, which then undergoes two consecutive IMAMRs. Deconvolution of the crude reaction mixture via ^{1}H NMR spectroscopic analysis reveals that the initial IMAMR occurs at both faces of the C-3 prochiral aza-Michael acceptor, affording the pyrrolidine intermediates (*S*,*S*)-**3c** and (*S*,*R*)-**3c** (*re*- and *si*-addition, respectively). In contrast, the second aza-Michael reaction occurs with exclusively pro-(*S*) selectivity, proceeding *via* putative aminium enolate intermediates.

To rationalise this substrate-controlled selectivity, possible transition states **TS I-IV** and **TS A-D** were devised. **TS I-II** and **TS A-B** exhibit the incorrect angle of approach of the non-bonding electrons on the nitrogen (Michael donor) to the π* orbital of the enone (Michael acceptor), leading to disfavoured orbital overlap and discounting these transition states. Unfavourable steric interactions during pyramidalisation occur *via* **TS-III** and **D**, leading to the aminium enolate intermediates **S23** and **S26** and disfavouring their formation. Considering this, the transition states **TS IV** and **C** are favoured, leading to the aminium intermediates **S24** and **S25**, respectively, which correspond to the observed products (**4c**). Considering this hypothesised explanation of the additional selectivity is correct, the selectivity can be seen as analogous to organocatalytic approaches but relying purely on the enzyme-installed chiral centre to achieve it.

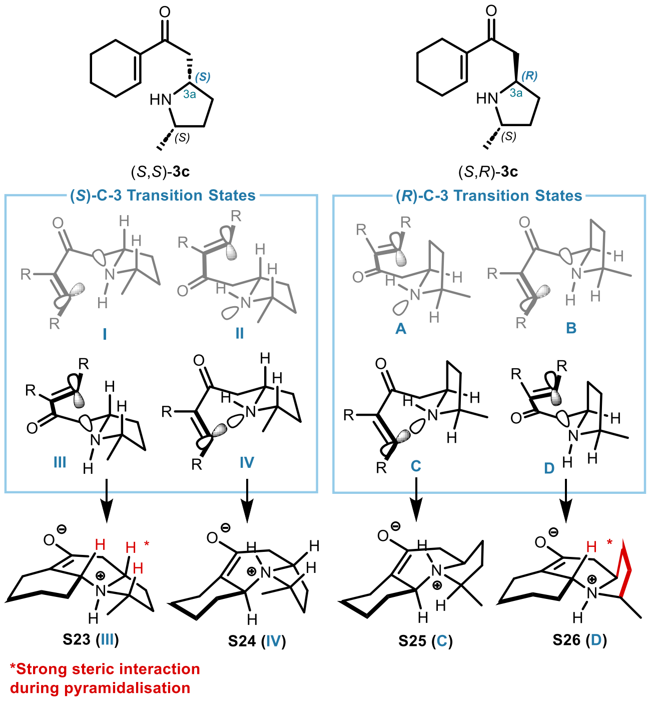

Figure S1. *Stereochemical model of the possible transition states corresponding to the second intramolecular aza-Michael reaction from the reactive intermediate (S,S)-**3c** or (S,R)-**3c**.*

4. Attempted epimerisation of 4f

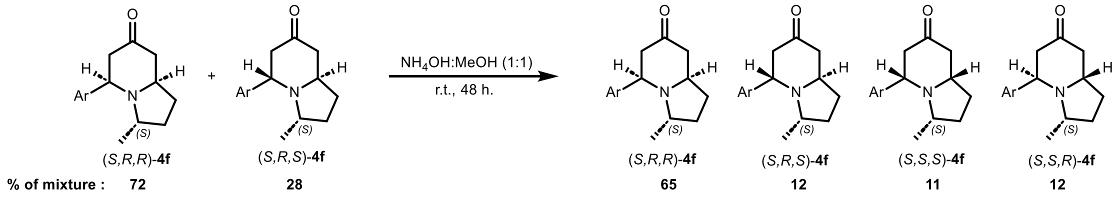

Scheme S10: *Attempted epimerisation of a mixture of **4f** displaying a possible double retro-aza-Michael reaction process.*

A solution of 35% aq. NH_4OH/MeOH 1:1 (5 mL) was added a a mixture of (*S*,*R*,*R*)-**4f** and (*S*,*R*,*S*)-**4f** (18 mg, mmol) and stirred at room temperature for 48 h. The solution was then extracted with dichloromethane (10 mL x 3), dried over MgSO4, filtered, and concentrated under reduced pressure. The crude residue was dissolved in $CDCl_3$ and analysed by ^{1}H NMR.

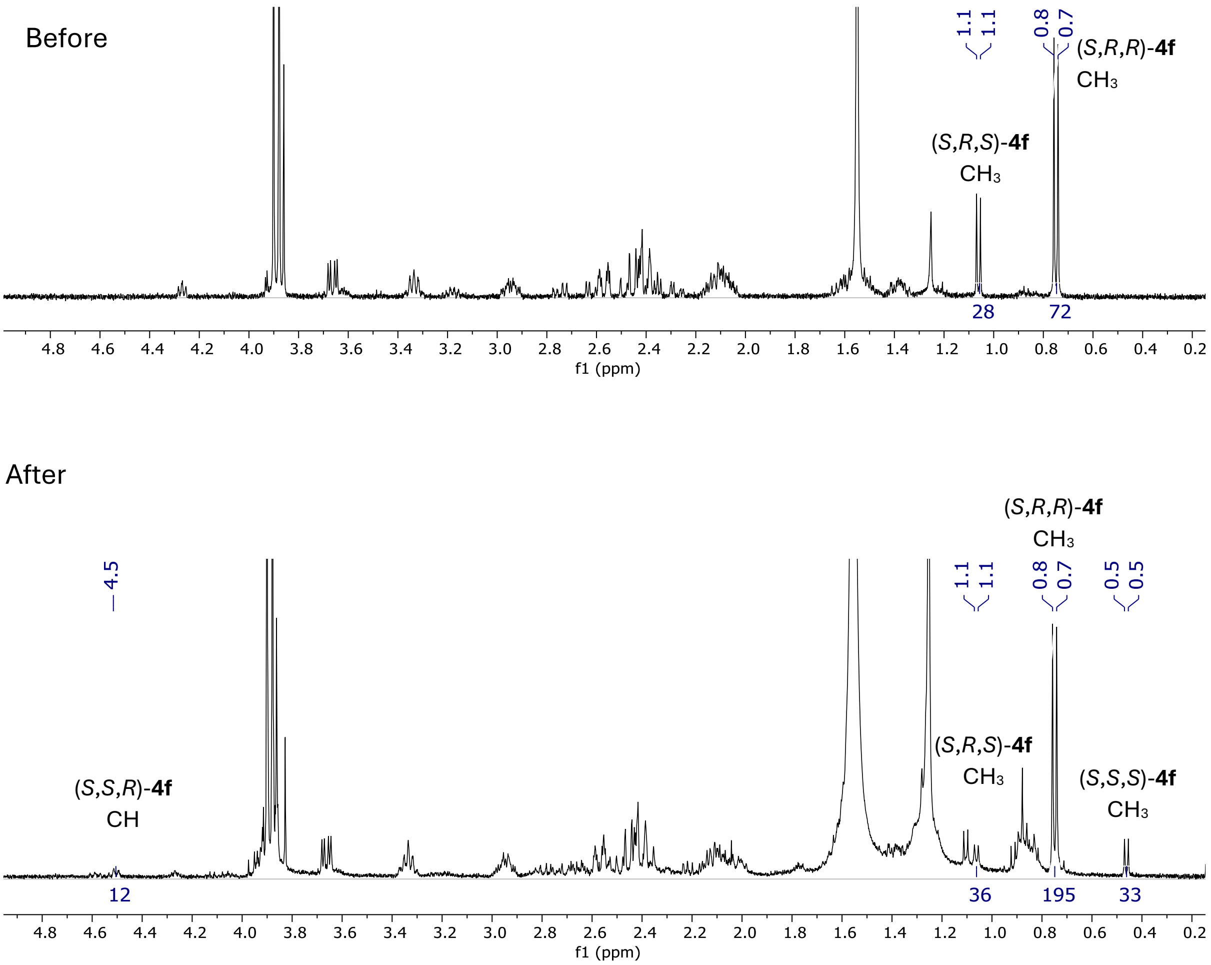

Figure S2: *^{1}H NMR analysis before and after attempted epimerisation with NH_4OH in methanol.*

5. Enzyme screen for the formation of diamine 5c from 4c

The enzyme (5 mg) were rehydrated in Na_3BO_3 buffer (800 μL, 25 mM, pH 9.0) containing PLP (1 mM) and isopropylamine (4 eq, 80 mM). The pH of the mixture was adjusted to 9.0, using aq. NaOH solution (1 M), and the total volume of the reaction was adjusted to 900 μL by addition of Na_3BO_3 buffer. Indolizidine (1*S*)-**4c** was added (20 mM, 100 μL from a 200 mM stock solution in MeOH) and the reaction mixture incubated at 50 °C, 220 rpm for 48 h. The reaction was basified to pH 10 and extracted using Et_2O (3 x 2 ml). The combined organic extracts were dried over $MgSO_4$, filtered and concentrated *in vacuo*. The crude residue was dissolved in $CDCl_3$ and analysed by ^{1}H NMR (Figure S3).

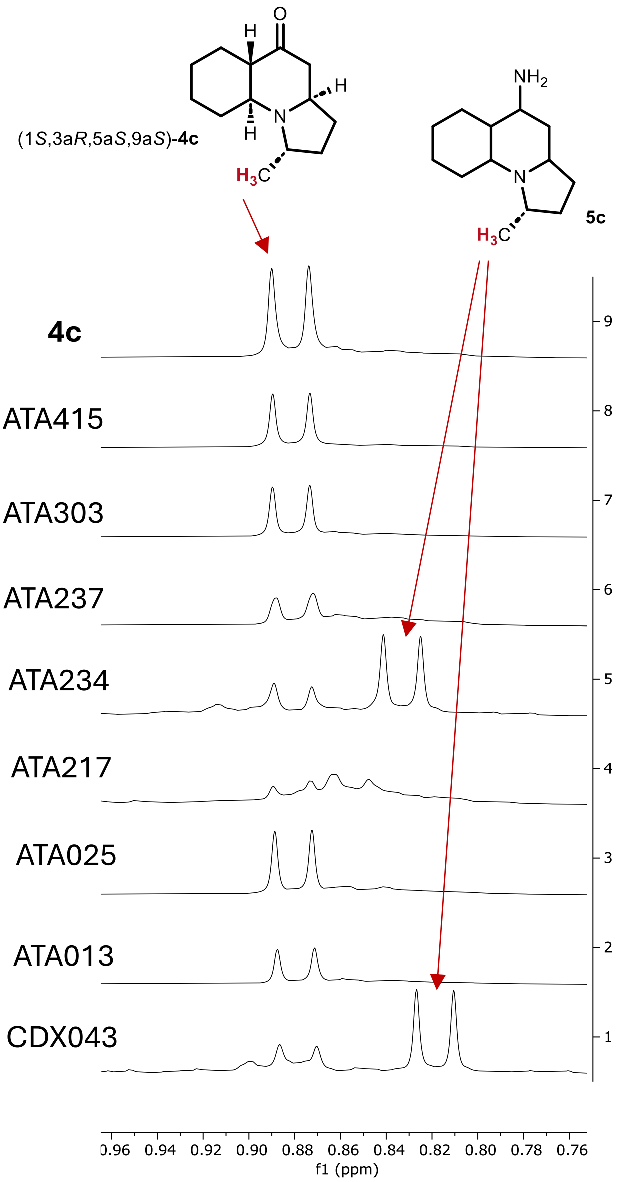

Figure S3. *^{1}H NMR analysis of the enzyme screen for the transformation of indolizidine **4c** to diamine **5c**.*

6. Study of reaction conditions for the two-enzyme cascade

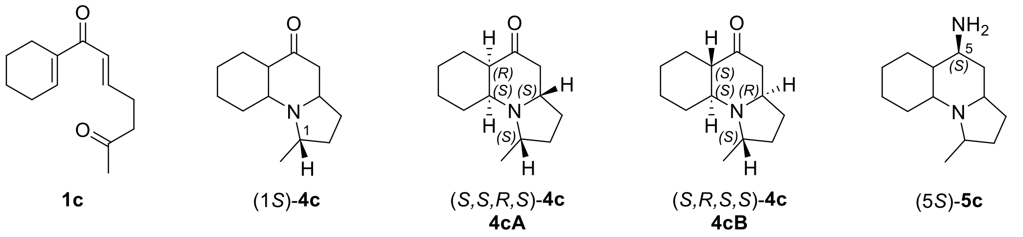

Figure S4: *Starting materials and target reaction products for the two-enzyme cascade.*

Table S3: Reaction conditions tested for the formation of diamine 5c

Entry	Starting material	Enzyme	Time	Buffer	IPA equiv.	Cosolvent	Reaction product(s)
1	**4c**	CDX043	48 h	100 mM HEPES pH 7.5	20	DMSO	**4c** (no reaction)
2	**4c**	CDX043	48 h	25 mM Na_3BO_3 pH 9.0	20	DMSO	**5c** 5*S*/5*R* 2:1.3 dr 39% isol. yield
3	**4c**	CDX043	48 h	25 mM Na_3BO_3 pH 9.0	20	MeOH	**5c** 5*S*/5*R* 2:1 dr 40% isol. yield
4	**4c**	ATA234	48 h	25 mM Na_3BO_3 pH 9.0	20	MeOH	**5c** 5*S*/5*R* 2:1 dr 40% isol. yield
5	**1c**	CDX043	48 h	25 mM Na_3BO_3 pH 9.0	20	MeOH	decomp.
6	**1c**	ATA234	48 h	25 mM Na_3BO_3 pH 9.0	20	MeOH	decomp.
7	**1c**	ATA256 +[a] CDX043	72 h	25 mM Na_3BO_3 pH 9.0	20	MeOH	**4c** (no amination) 72% isol. yield
8	**1c**	ATA256 then[b] ATA234	24h then[b] 24h	25 mM Na_3BO_3 pH 9.0	4 then[b] 16	MeOH	**4cA**:**4cB**:**5c** (1 : 0.4 : 0.96) 39% isol. yield
9	**1c**	ATA256 then[b] ATA234	24h then[b] 24h	25 mM Na_3BO_3 pH 9.0	4 then[b] 4	MeOH	**4cA**:**4cB**:**5c** (1 : 0.33 : 1.08) 43% isol. yield
10	**1c**	ATA256 then[b] ATA234	24h then[b] 24h	25 mM Na_3BO_3 pH 9.0	4	MeOH	**4cA**:**4cB**:**5c** (1 : 0.52 : 1.15) 51% isol. yield
11	**1c**	ATA256 +[a] ATA234	48 h	25 mM Na_3BO_3 pH 9.0	4	MeOH	**4cA**:**4cB**:**5c** (1 : 0.65 : 1.17) 47% isol. yield
12	**1c**	ATA256 then[b] ATA234	24h then[b] 48h	25 mM Na_3BO_3 pH 9.0	4	MeOH	**5c** 45% isol. yield
13	**1c**	ATA256 +[a] ATA234	65 h	25 mM Na_3BO_3 pH 9.0	4	MeOH	**4cA**:**5c** (0.66 : 1) 42% isol. yield
14	**1c**	ATA256 +[a] ATA234	72 h	25 mM Na_3BO_3 pH 9.0	4	MeOH	**5c** 42% isol. yield
15	(1*R*)-**4c**	ATA234	48 h	25 mM Na_3BO_3 pH 9.0	4	MeOH	(1*R*)-**4c** (no reaction)

Consistent reaction conditions: 6 mL reaction; 40 mM starting material, 5 mg/mL enzyme, 1 mM PLP, 10% cosolvent, 50 °C, 220 rpm. [a]One pot reaction, [b]Sequential reaction. Isolated yields are reported of the non-purified products.

The ATA234 catalysed amination of the crude isomeric mixture **4a**, gave products **5aA**, **4aB**, **4aC**, and **4aD** (Scheme S11). The isomeric ratio of the unreacted species **4a** were the same in the biotransformation product mixture as in the starting material. This indicates a kinetic resolution process without any dynamic interconversion present under the tested biotransformation conditions. The same product mixture was evident in the two-enzyme cascade (Scheme S12), where the partially cyclised piperidine (*S*,*S*)-**3a** was also present.

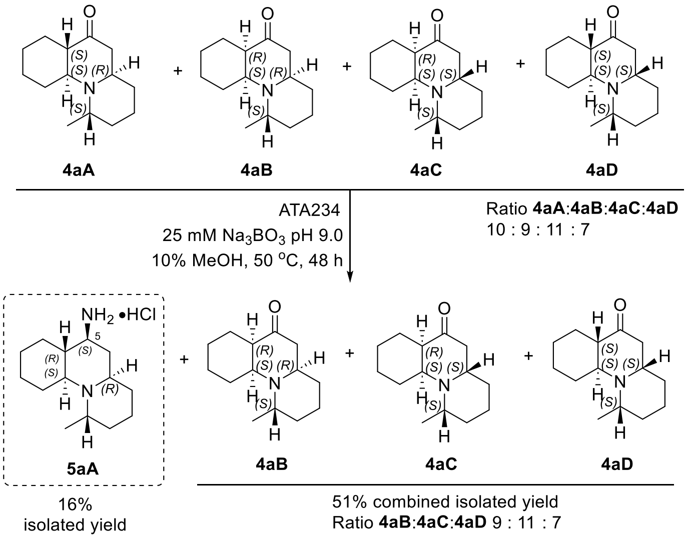

Scheme S11: *ATA234 catalysed kinetic resolution of the **4a** diastereomeric mixture.*

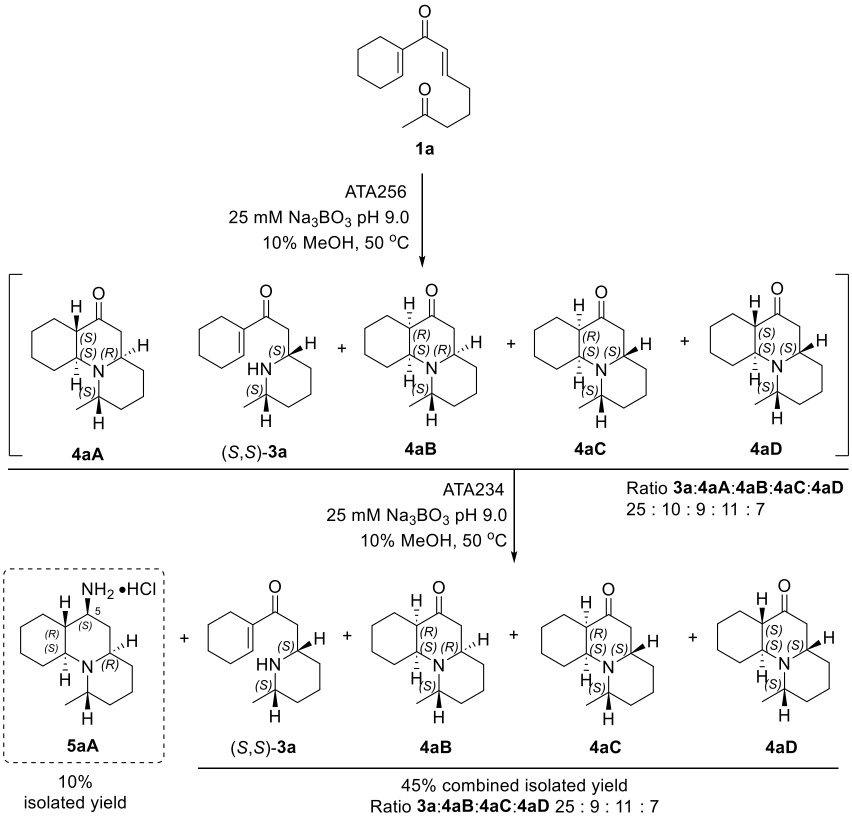

Scheme S12: *ATA234 catalysed kinetic resolution of the **4a** diastereomeric mixture in a one pot cascade from substrate **1a**.*

Full conversion of the indolizidine **4c** could be achieved, but the reaction yield from the two-enzyme cascade could not account for full mass balance. We therefore wanted to examine if the full conversion of starting material was due to a dynamic resolution or if the stereoisomers not accepted by the second ATA in the cascade were simply decomposing under biotransformation conditions.

First, the isolated yields presented in Table S3 (Entries 2-4 and 8-14) are approx 45% regardless of product distribution, which may indicate a dynamic process. Second, the retro aza Michael

mechanism is supported by the observation presented in Scheme 4c (main text), in which epimerisation of the β-carbon of compound **4c** was observed under biotransformation conditions. Third, we obtain the same diamine stereoisomer **5c** regardless of the stereochemistry of the **4c** isomer starting material (Scheme S13). We ascribe the dynamic process which interconverts the α- and β- stereocentres to the double retro aza Michael reaction, depicted in Scheme S13. As shown in scheme 13 it is also possible that the reaction mechanism is a retro aza Michael/Mannich reaction.

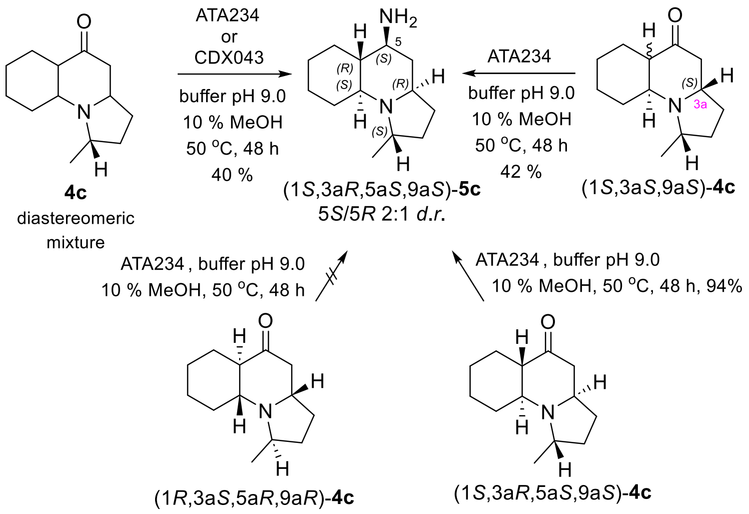

Scheme S13: *The same* ***5c*** *stereoisomer is produced in the ATA catalysed biotransformation regardless of the stereochemistry of the starting material.*

***Retro*-aza-Michael stereochemical scrambling**

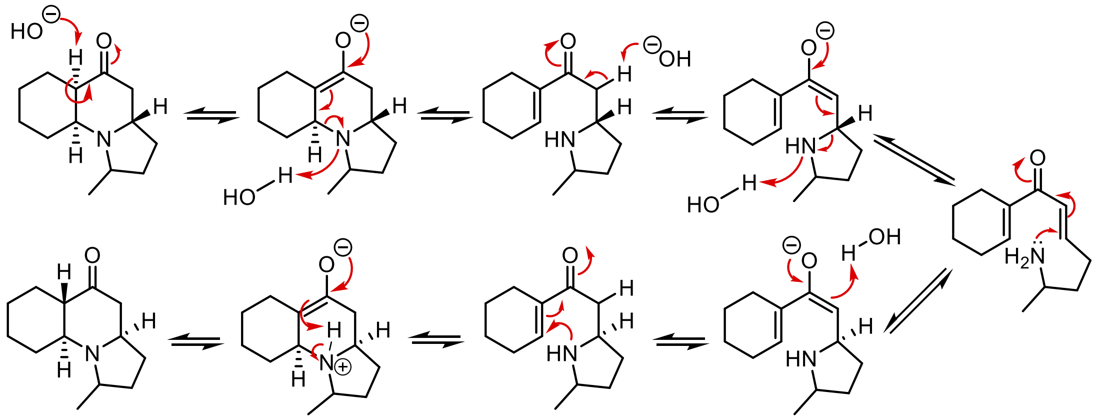

Scheme S14: *Scheme S13: Proposed mechanism for the stereochemical scrambling of stereocentres under basic conditions, via the double retro aza-Michael reaction. Other mechanisms are also possible.*[12]

7. NMR Spectra

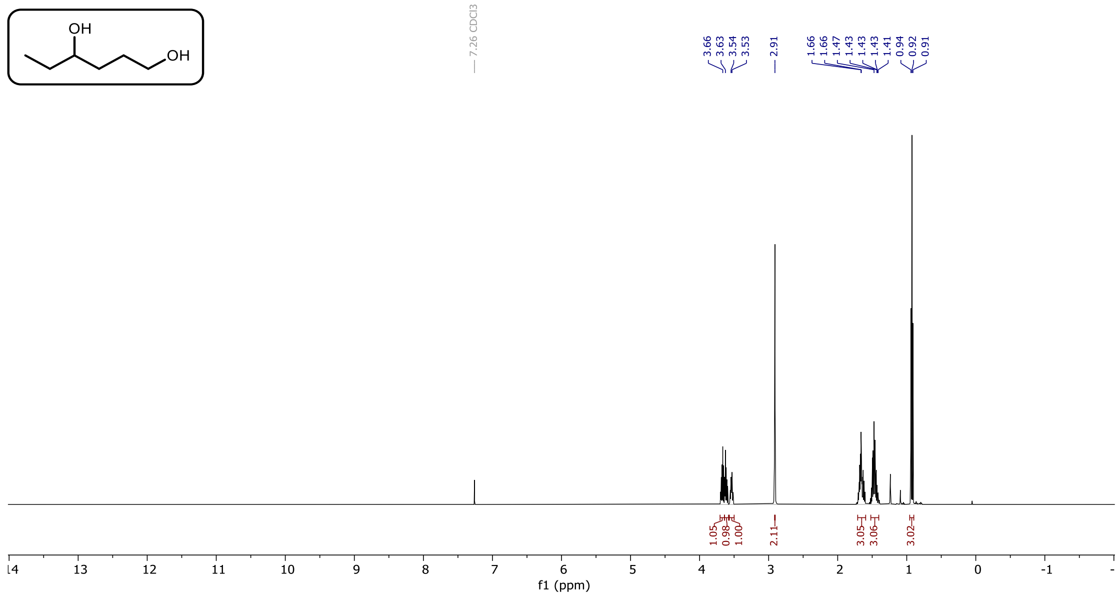

A1: *^{1}H NMR ($CDCl_3$) of compound* ***S1***.

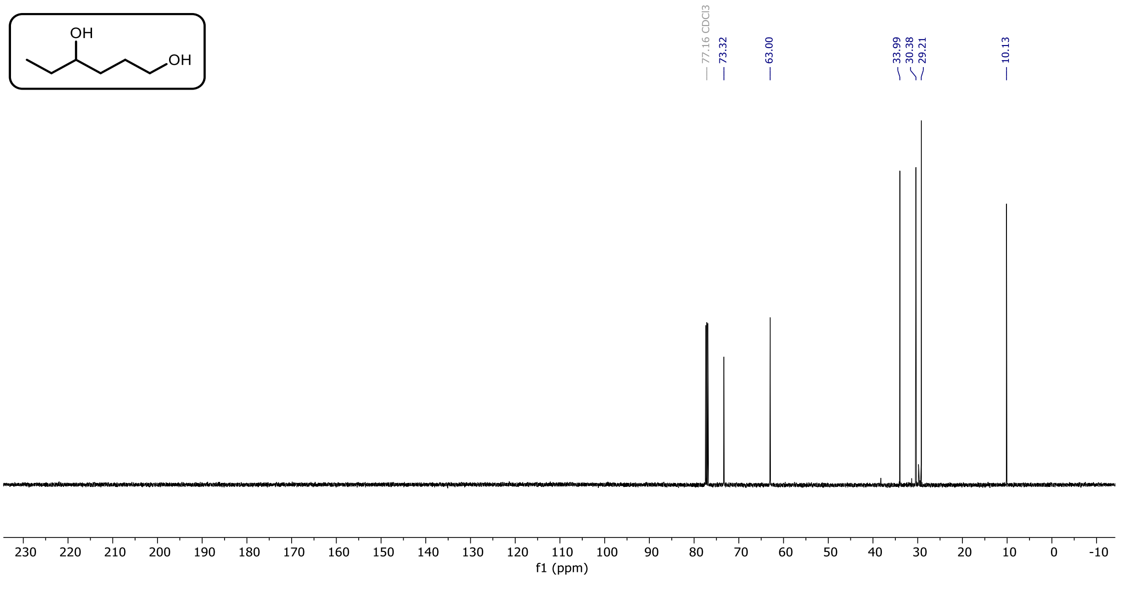

A2: *^{13}C NMR ($CDCl_3$) of compound* ***S1***.

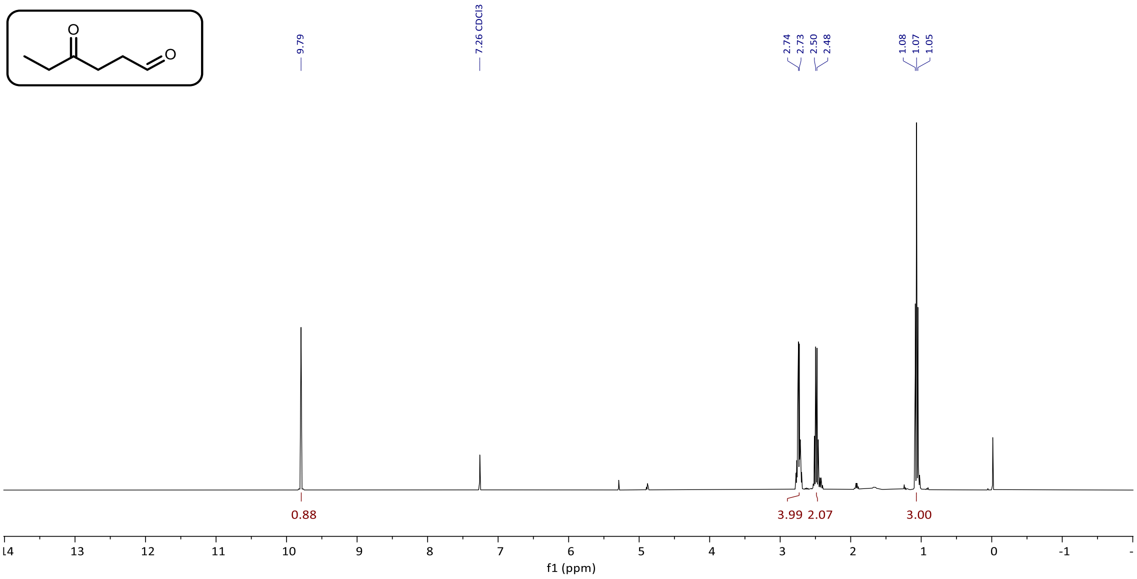

A3: *^{1}H NMR (CDCl₃) of compound* ***S2****.*

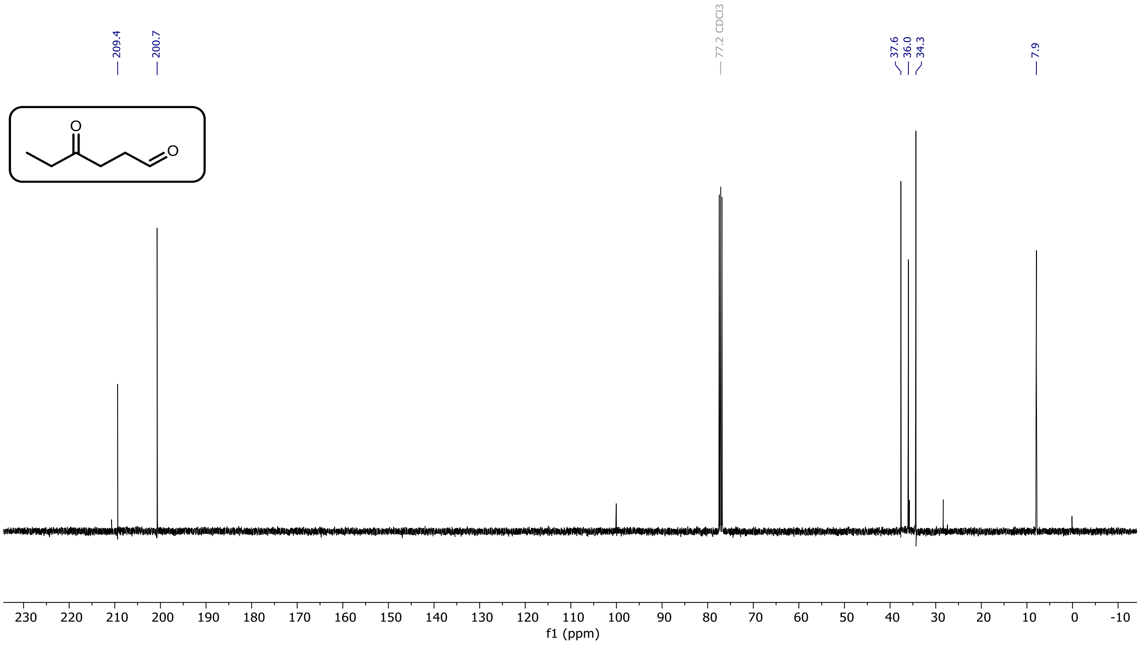

A4: *^{13}C NMR (CDCl₃) of compound* ***S2****.*

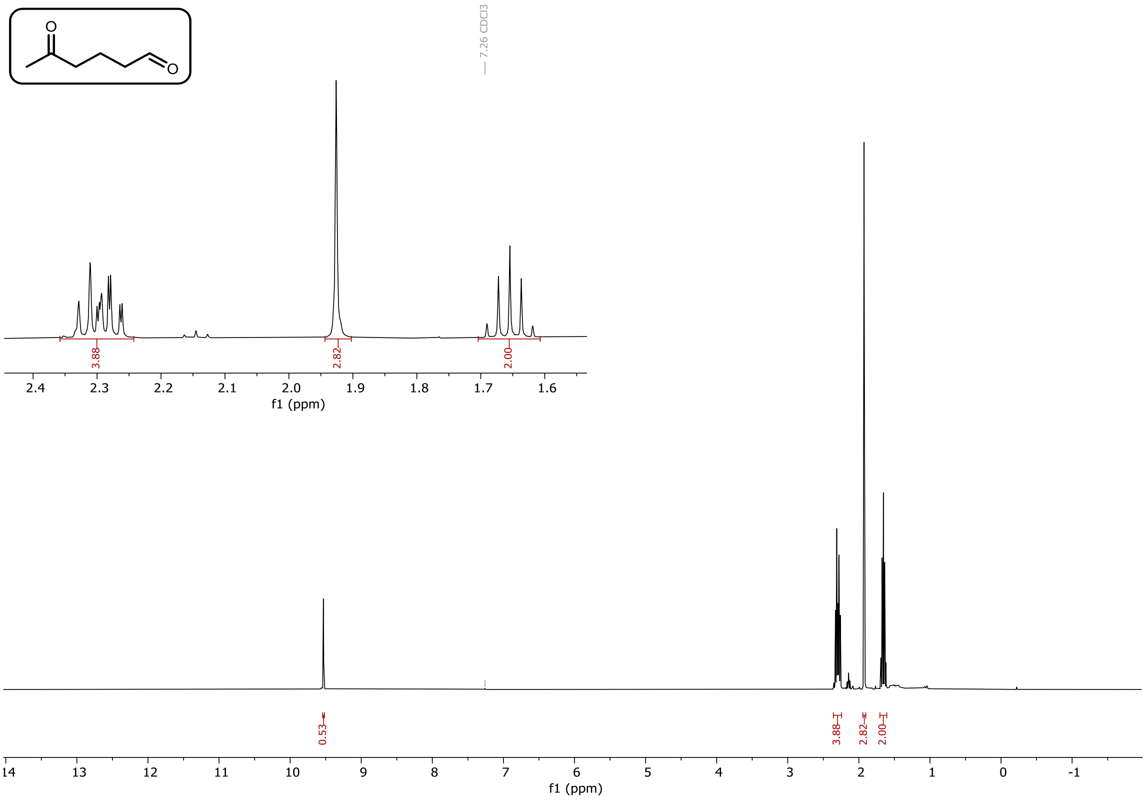

A5: *¹H NMR (CDCl₃) of compound* ***S3****.*

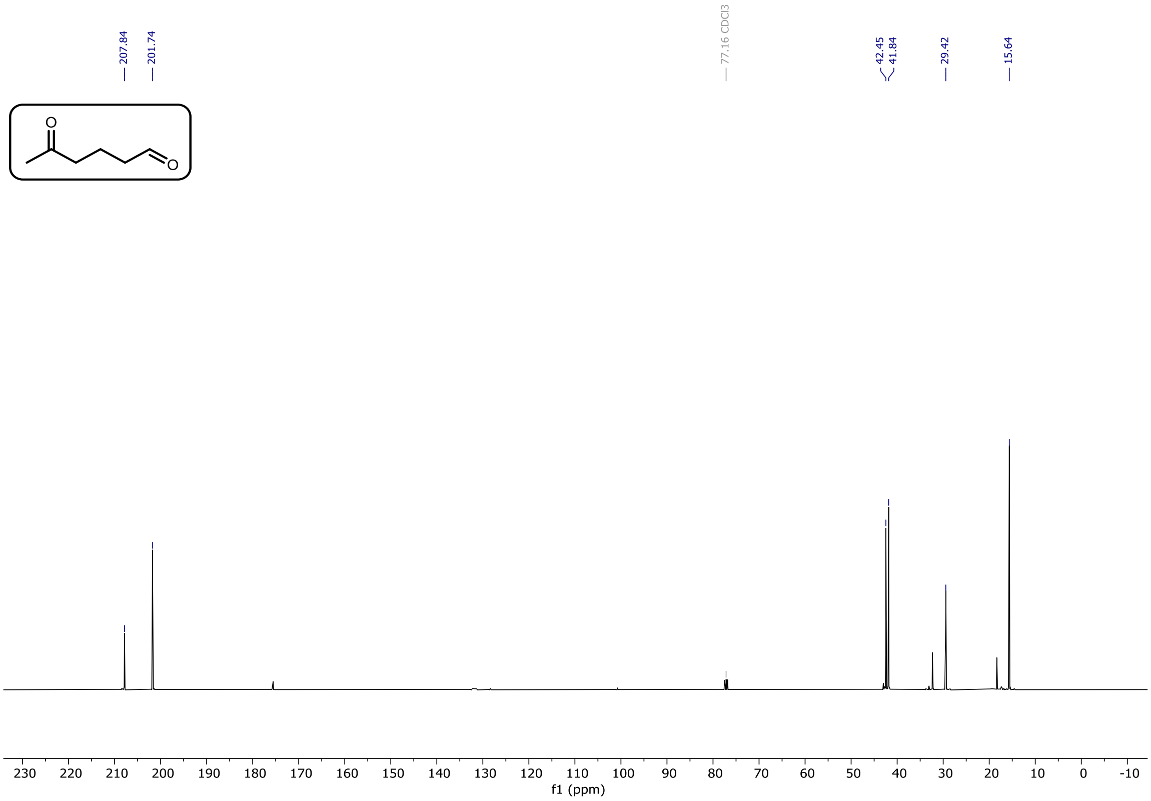

A6: *¹³C NMR (CDCl₃) of compound* ***S3****.*

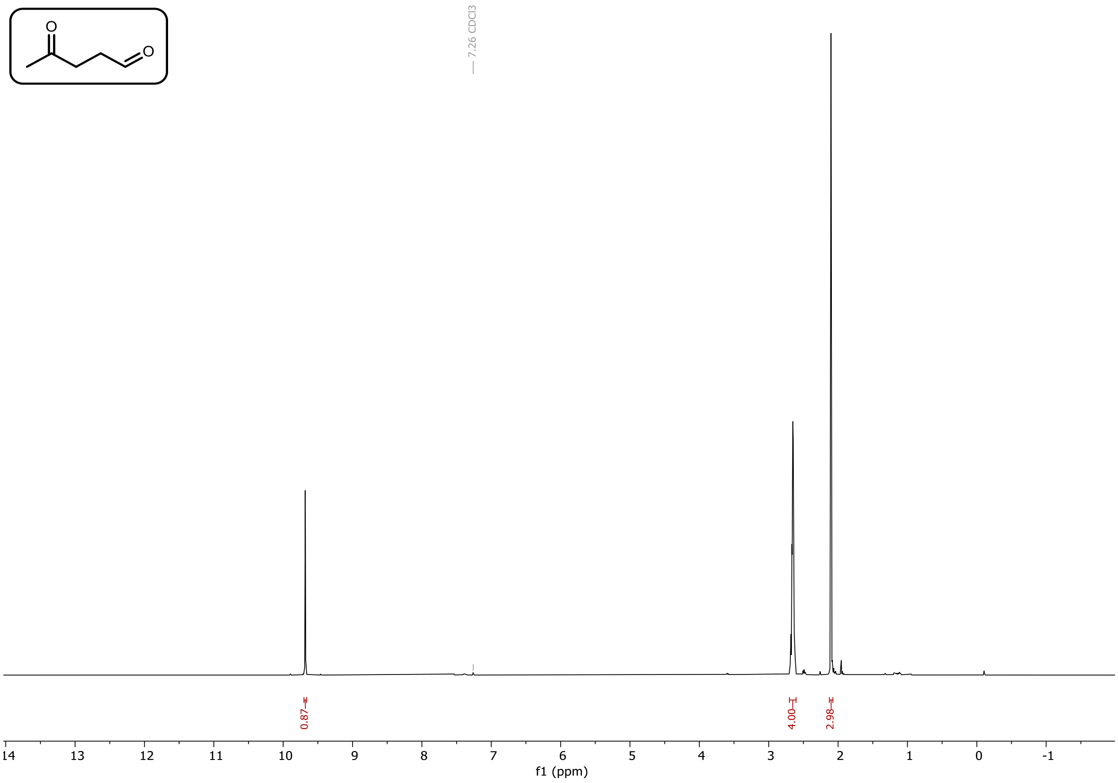

A7: *^{1}H NMR (CDCl$_3$) of compound* ***S4****.*

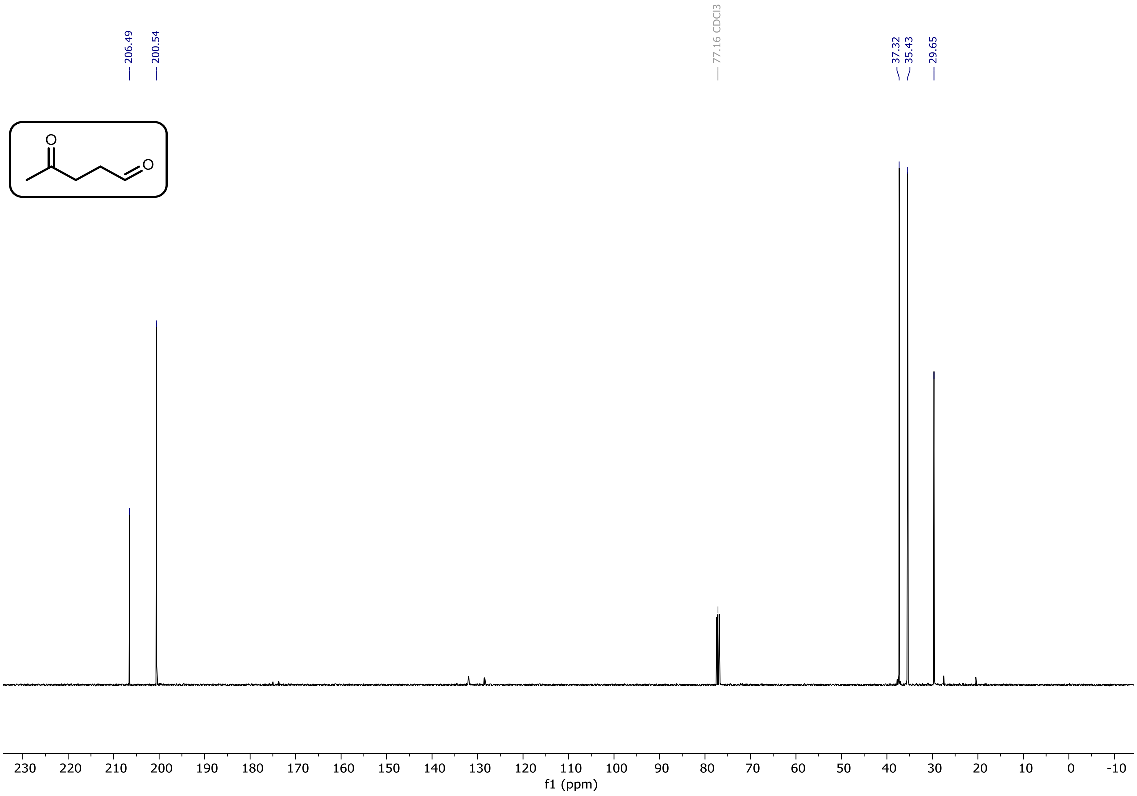

A8: *^{13}C NMR (CDCl$_3$) of compound* ***S4****.*

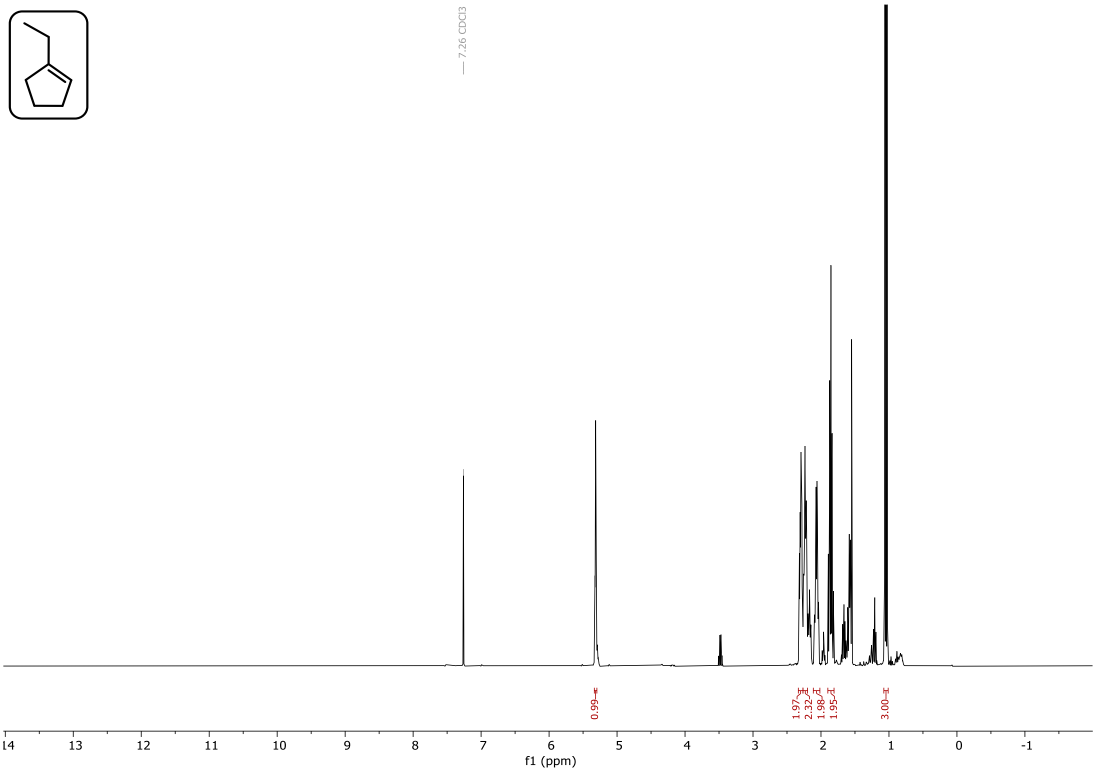

A9: *^{1}H NMR ($CDCl_3$) of compound* ***S5****.*

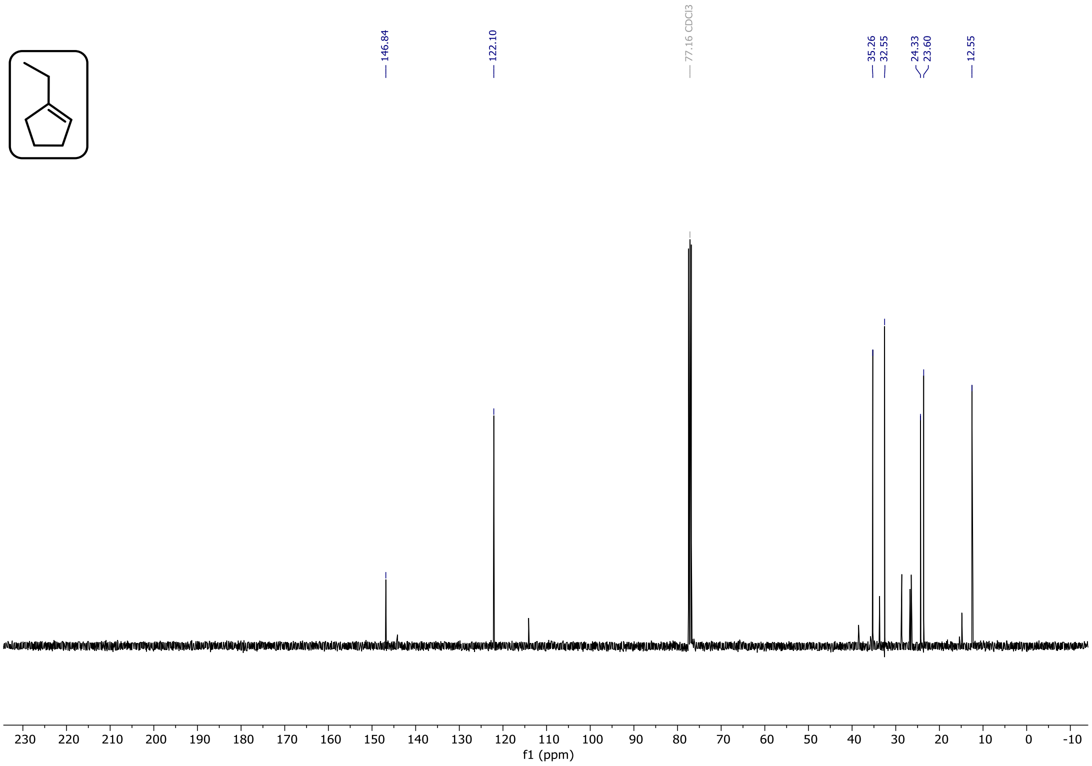

A10: *^{13}C NMR ($CDCl_3$) of compound* ***S5****.*

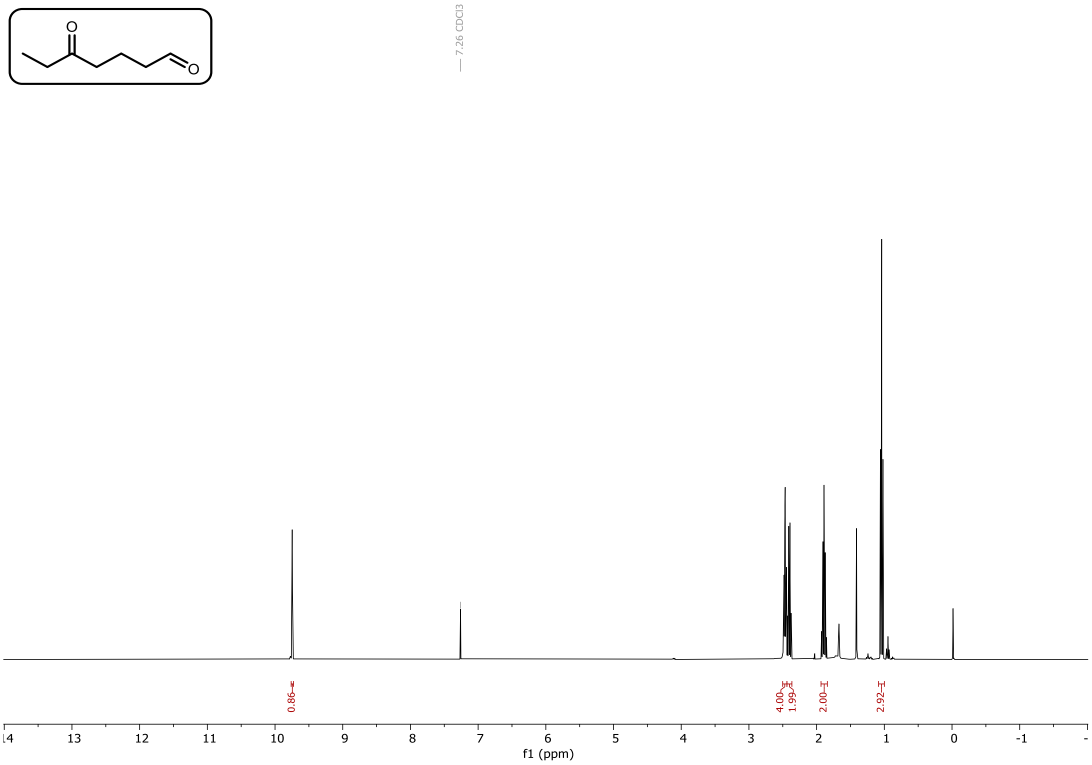

A11: ^{1}H *NMR ($CDCl_3$) of compound* ***S6****.*

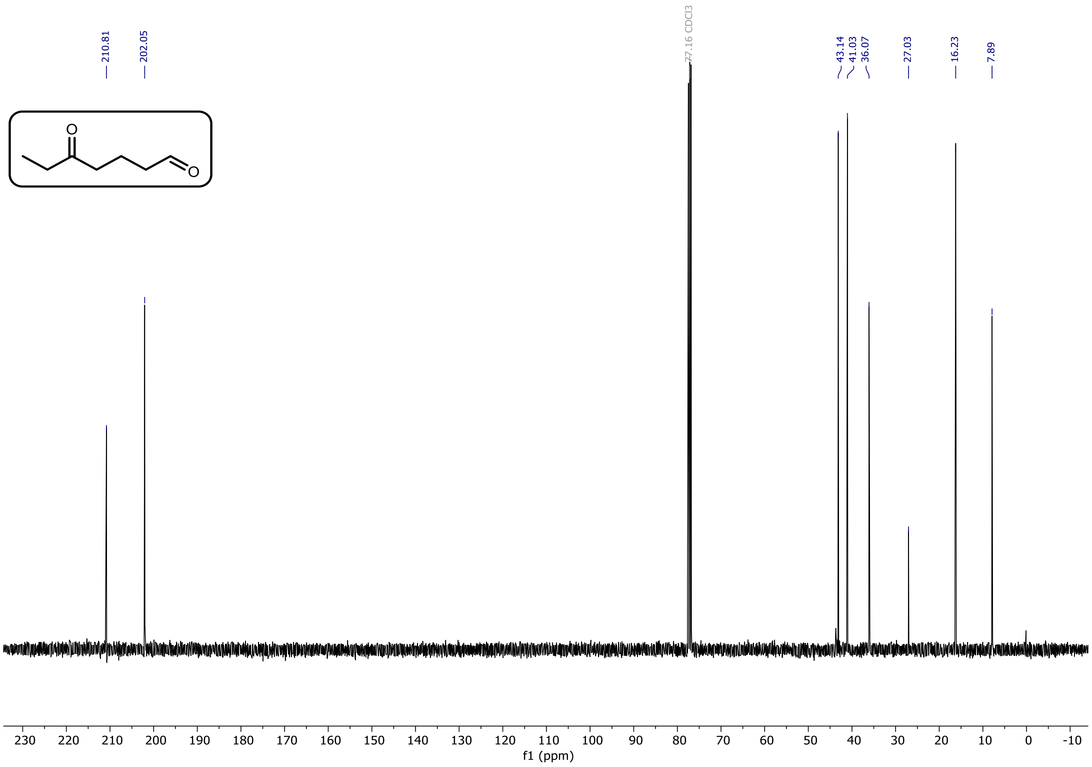

A12: ^{13}C *NMR ($CDCl_3$) of compound* ***S6****.*

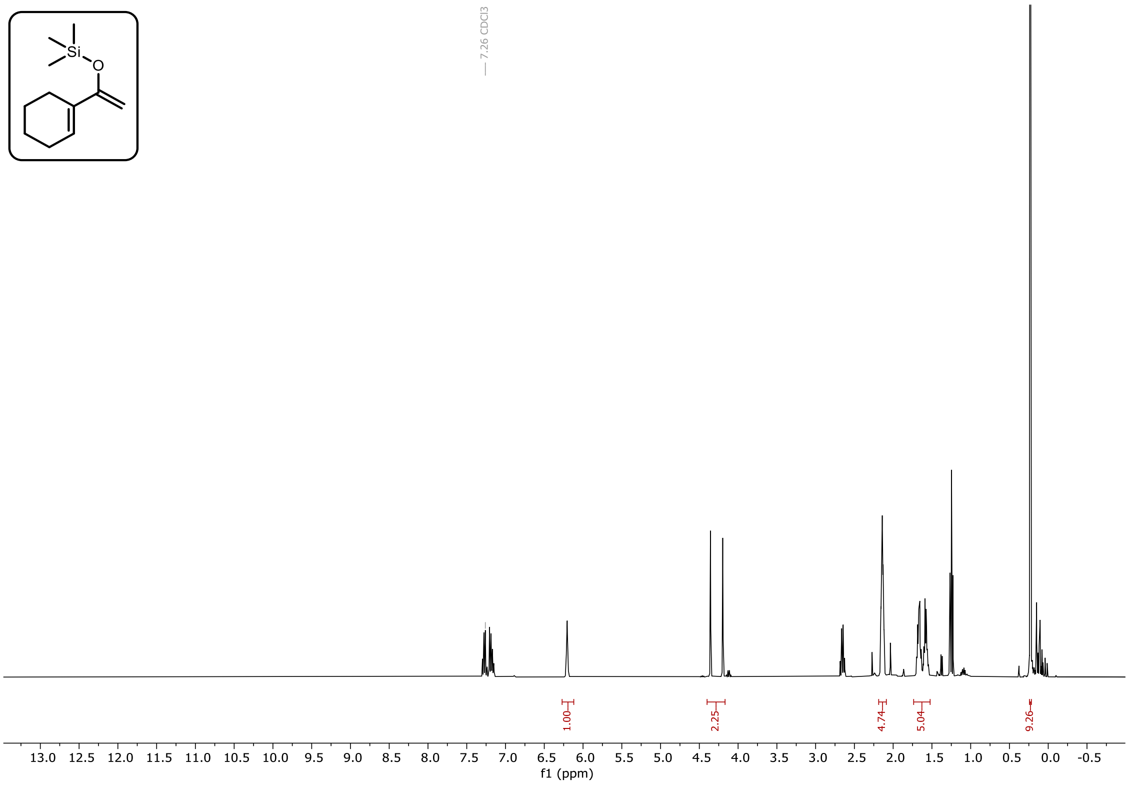

A13: *^{1}H NMR ($CDCl_3$) of compound* ***S7***.

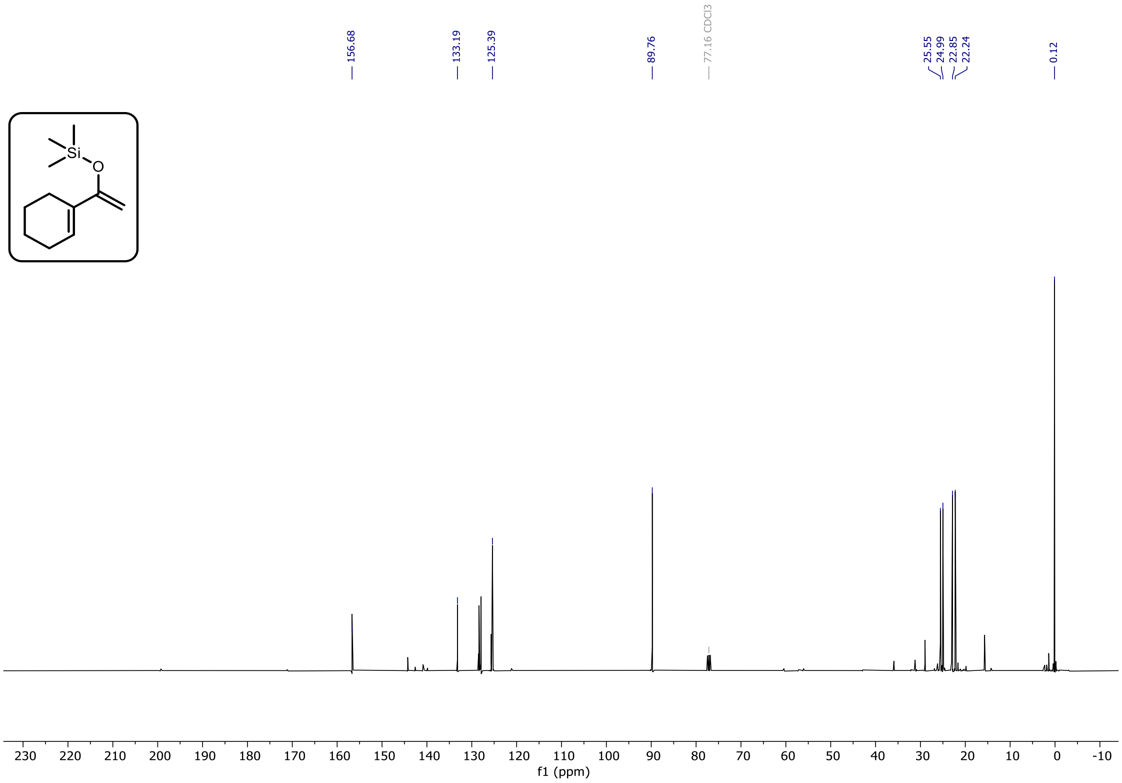

A14: *^{13}C NMR ($CDCl_3$) of compound* ***S7***.

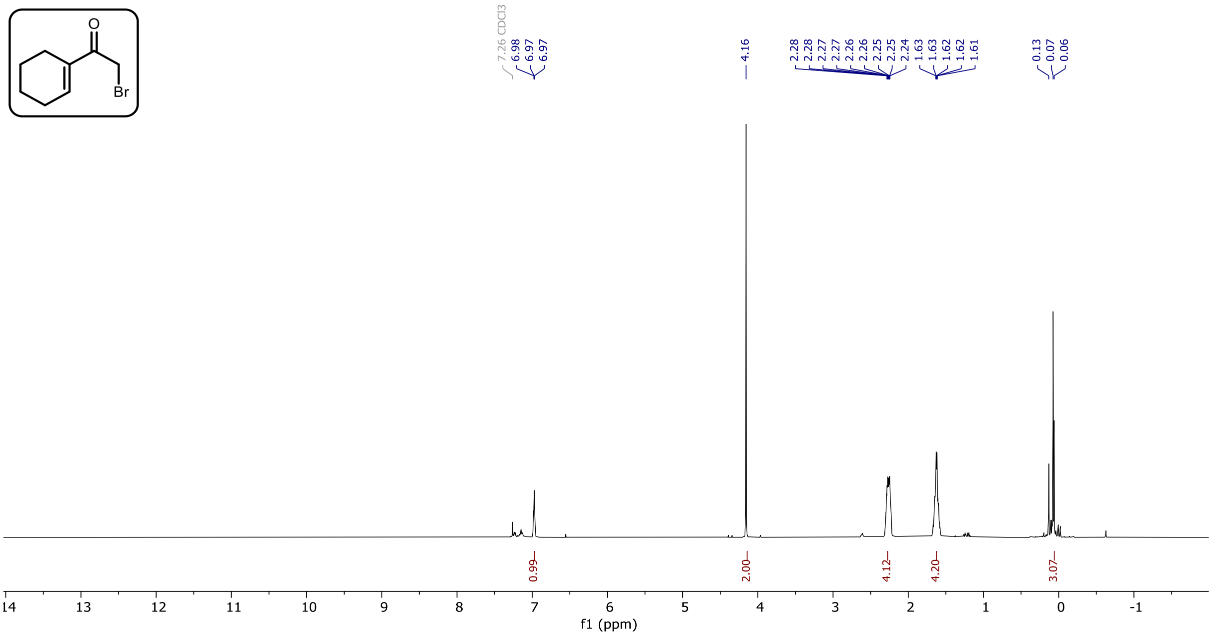

A15: *^{1}H NMR (CDCl$_3$) of compound* ***S8****.*

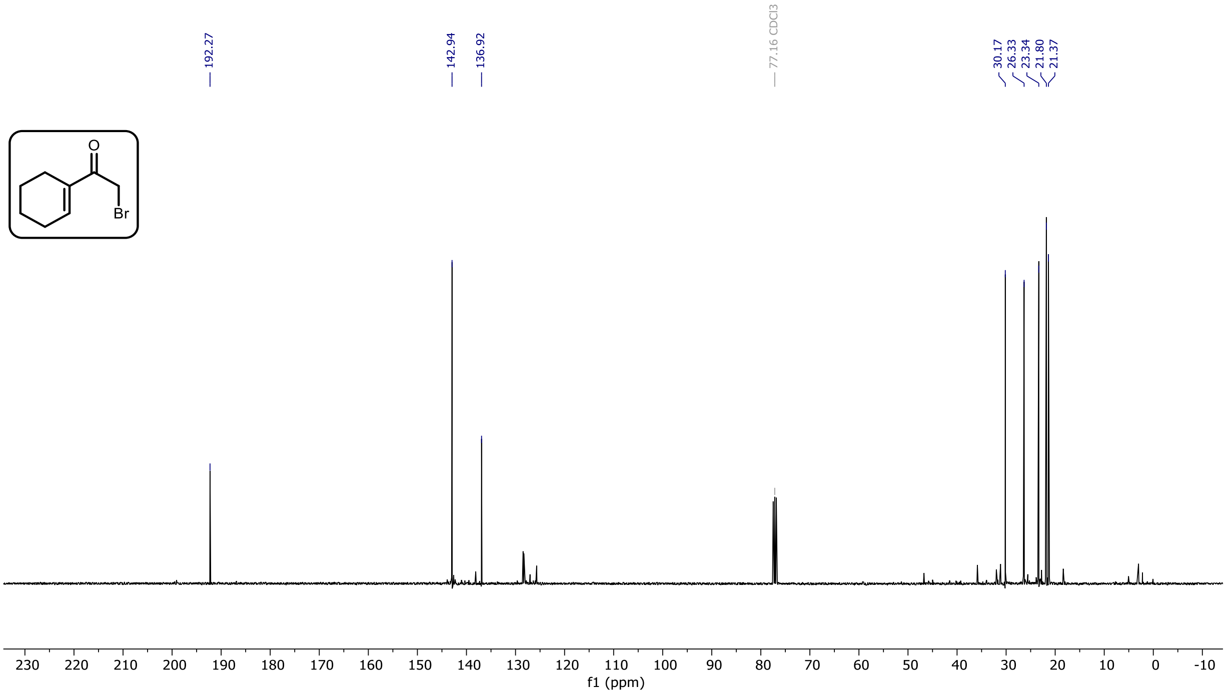

A16: *^{13}C NMR (CDCl$_3$) of compound* ***S8****.*

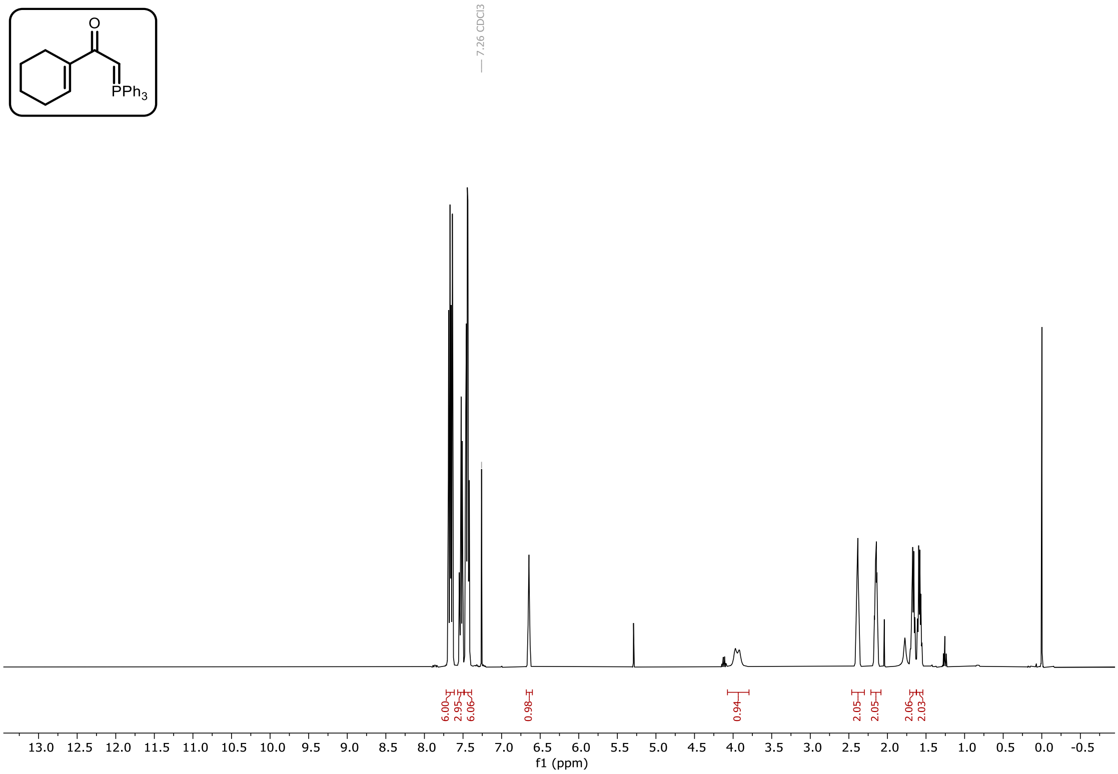

A17: *^{1}H NMR ($CDCl_3$) of compound* ***S9***.

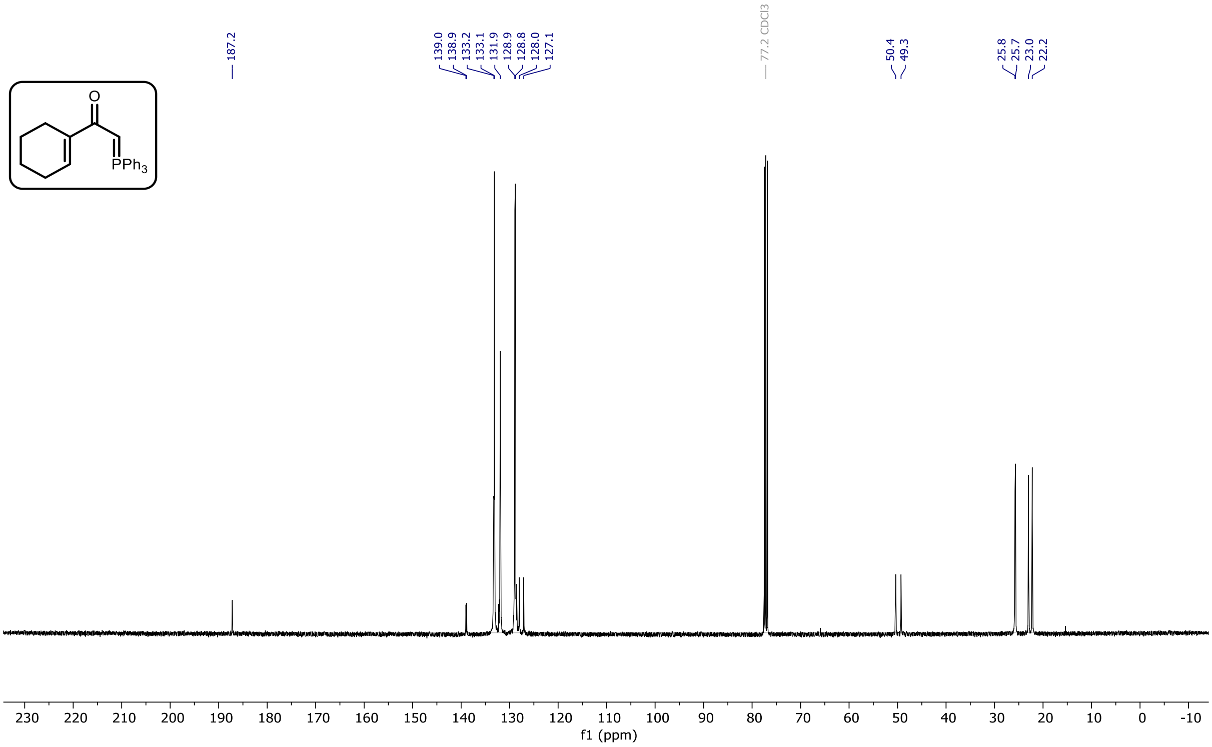

A18: *^{13}C NMR ($CDCl_3$) of compound* ***S9***.

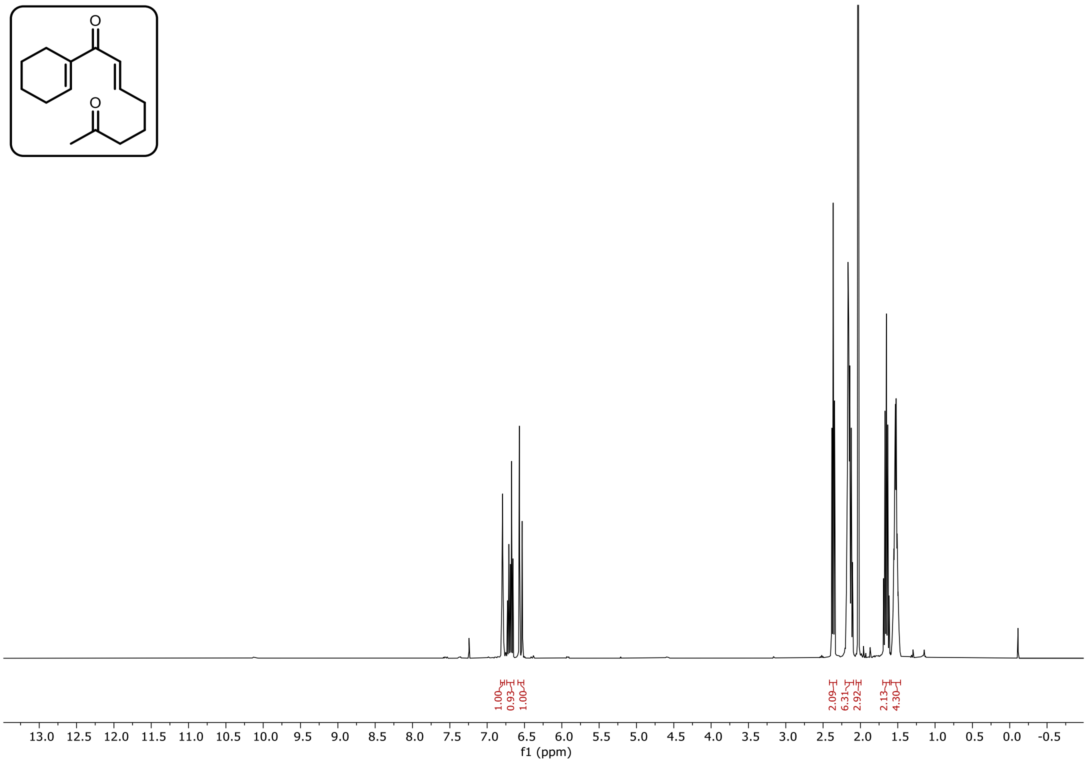

A19: *^{1}H NMR (CDCl$_3$) of compound **1a**.*

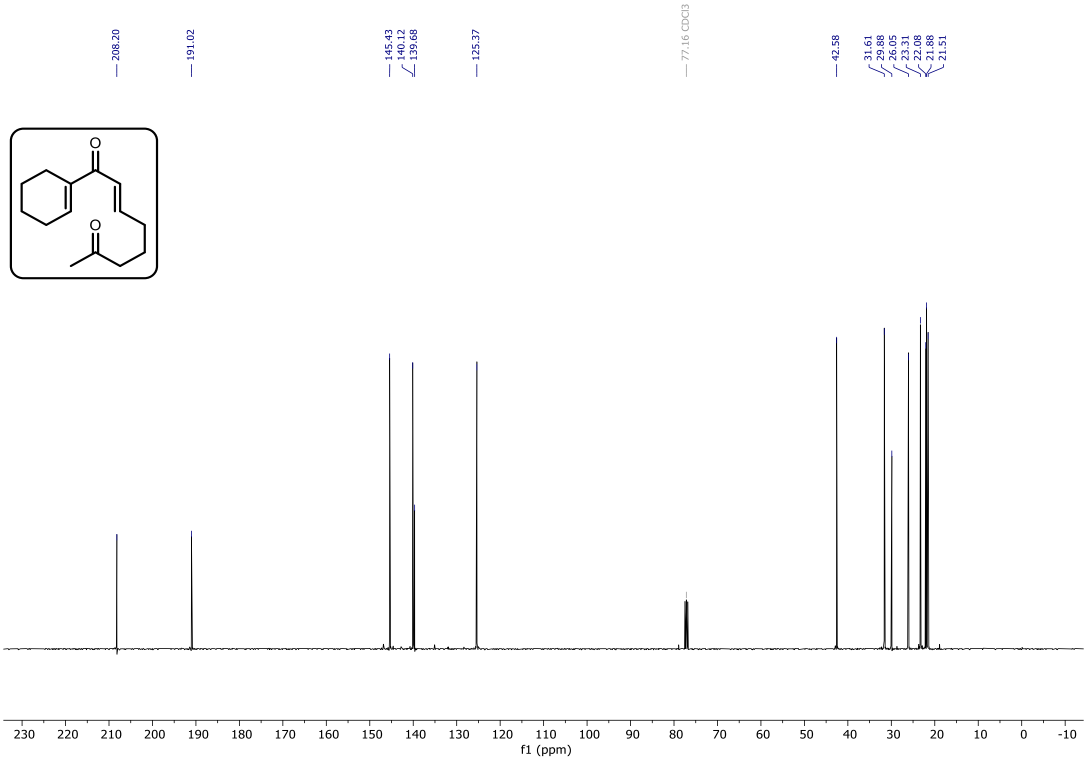

A20: *^{13}C NMR (CDCl$_3$) of compound **1a**.*

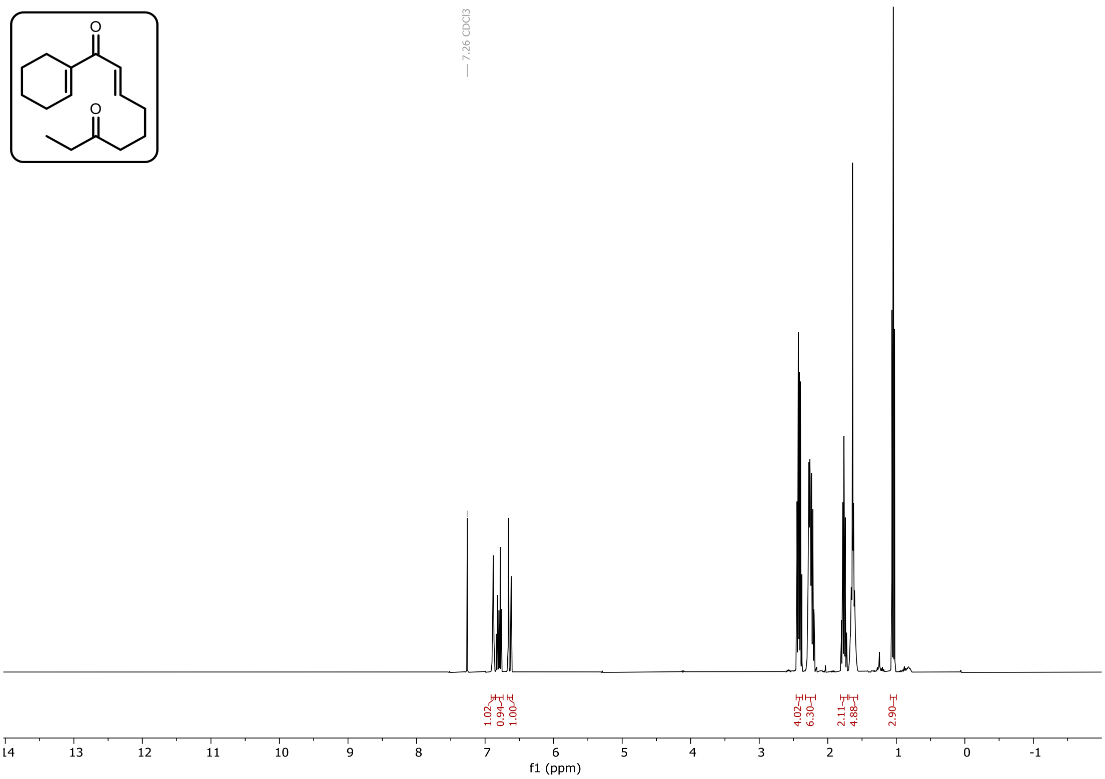

A21: *^{1}H NMR (CDCl₃) of compound* ***1b*****.**

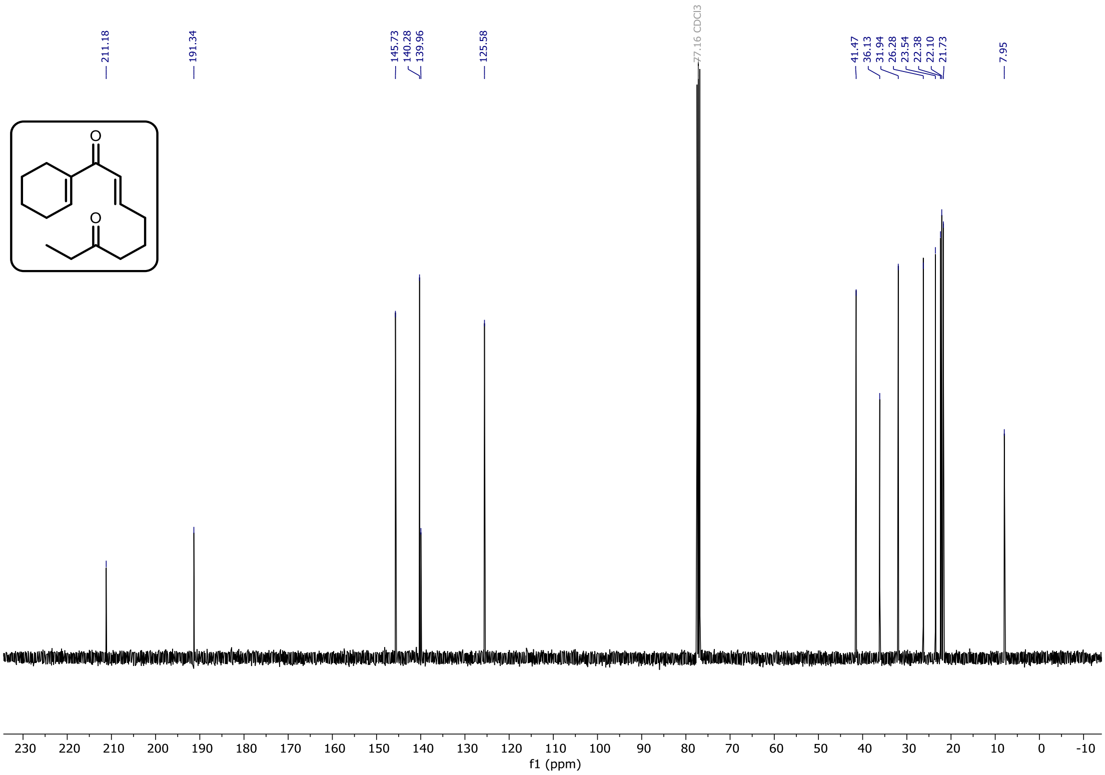

A22: *^{13}C NMR (CDCl₃) of compound* ***1b*****.**

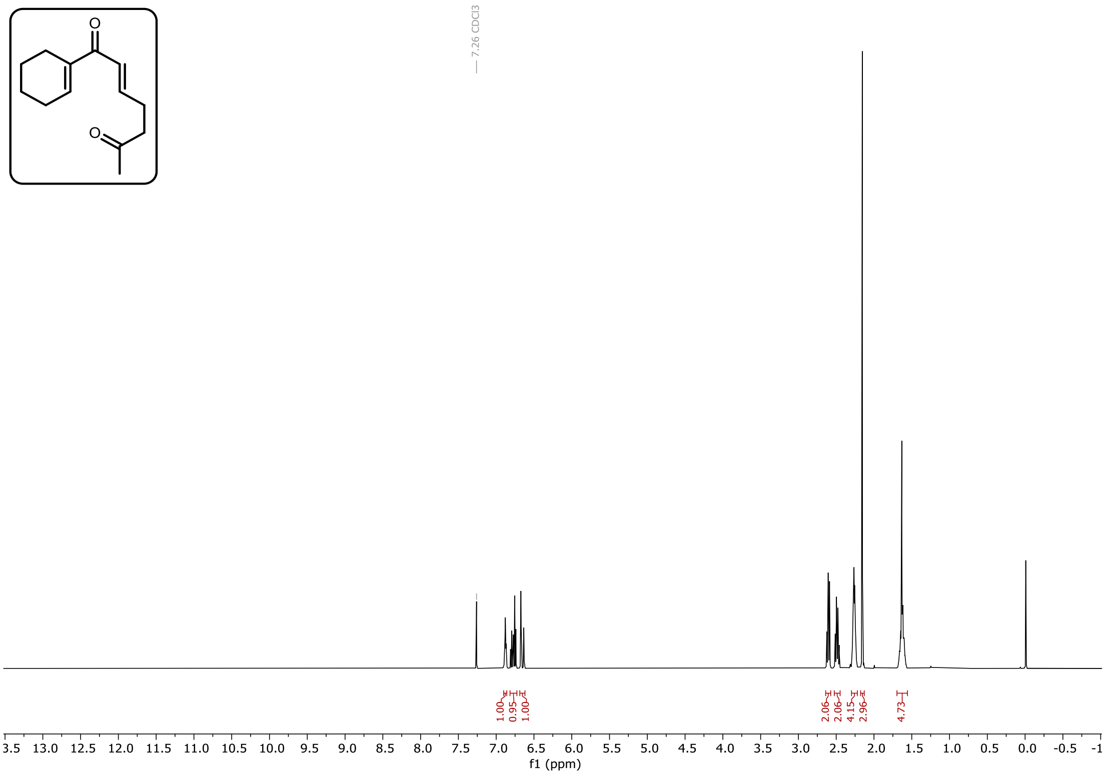

A23: *^{1}H NMR ($CDCl_3$) of compound* ***1c****.*

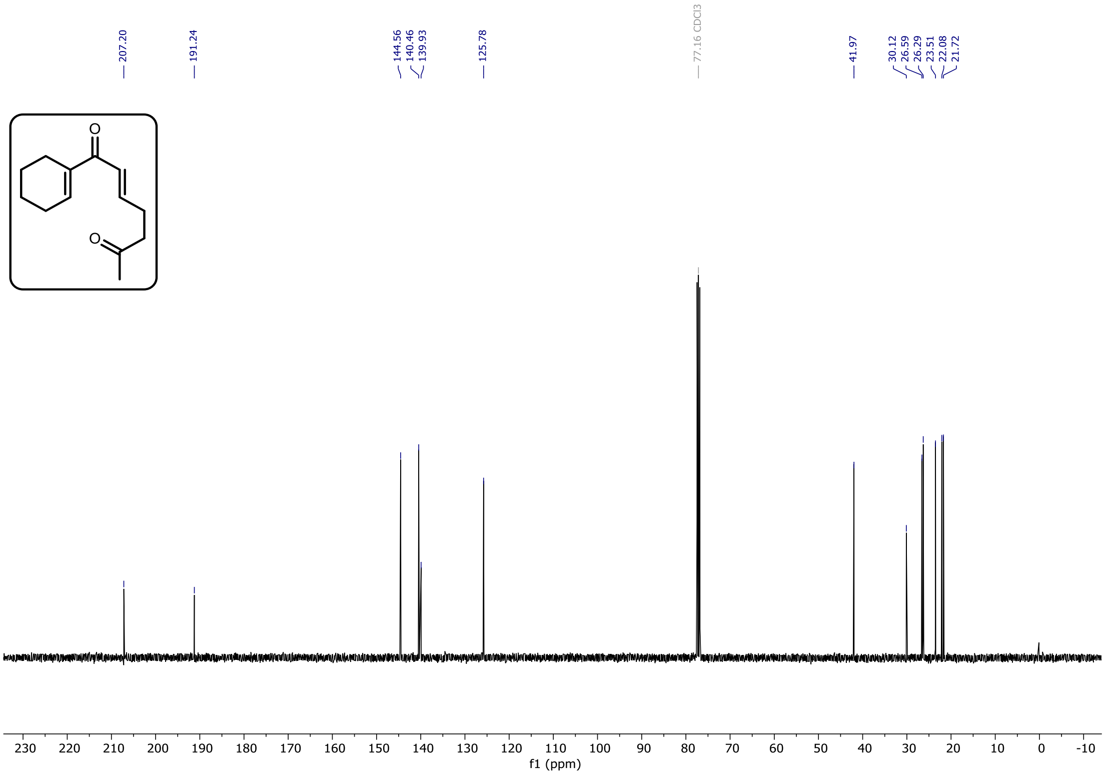

A24: *^{13}C NMR ($CDCl_3$) of compound* ***1c****.*

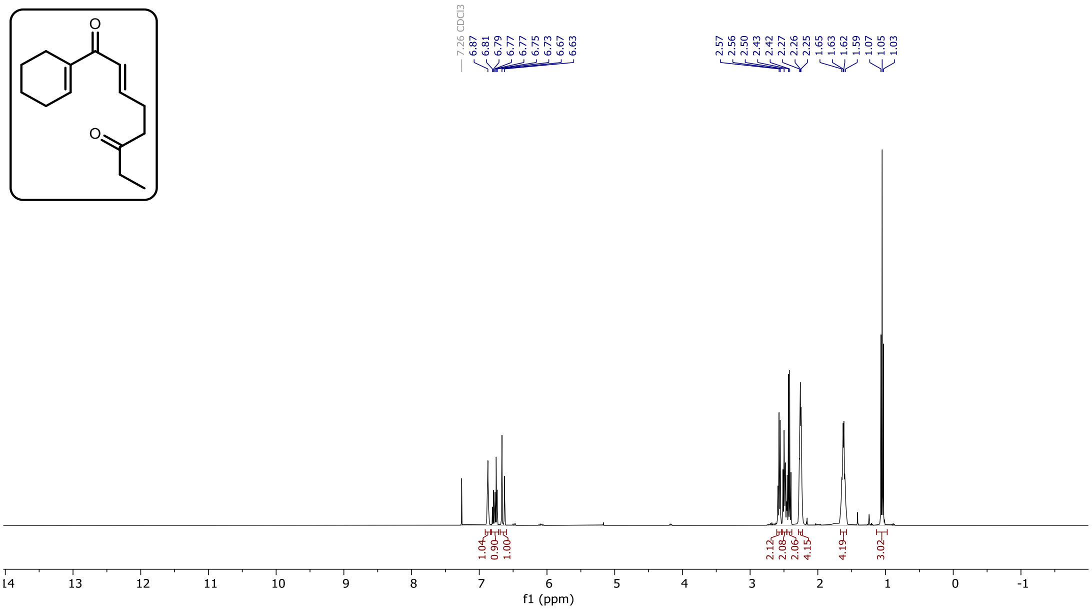

A25: *^{1}H NMR (CDCl$_3$) of compound* ***1d***.

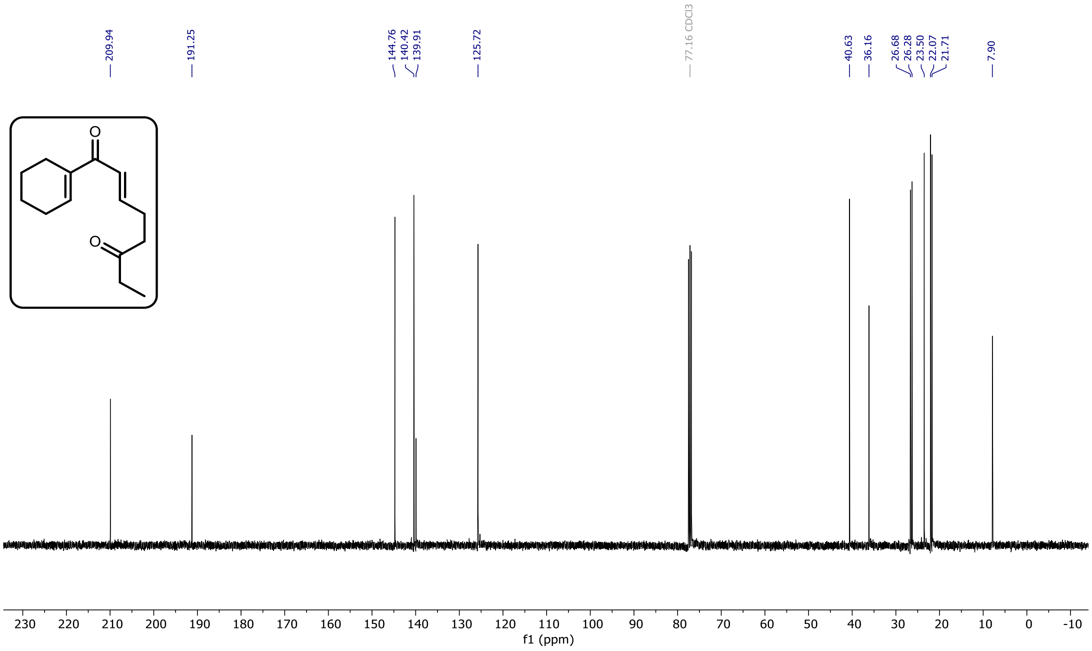

A26: *^{13}C NMR (CDCl$_3$) of compound* ***1d***.

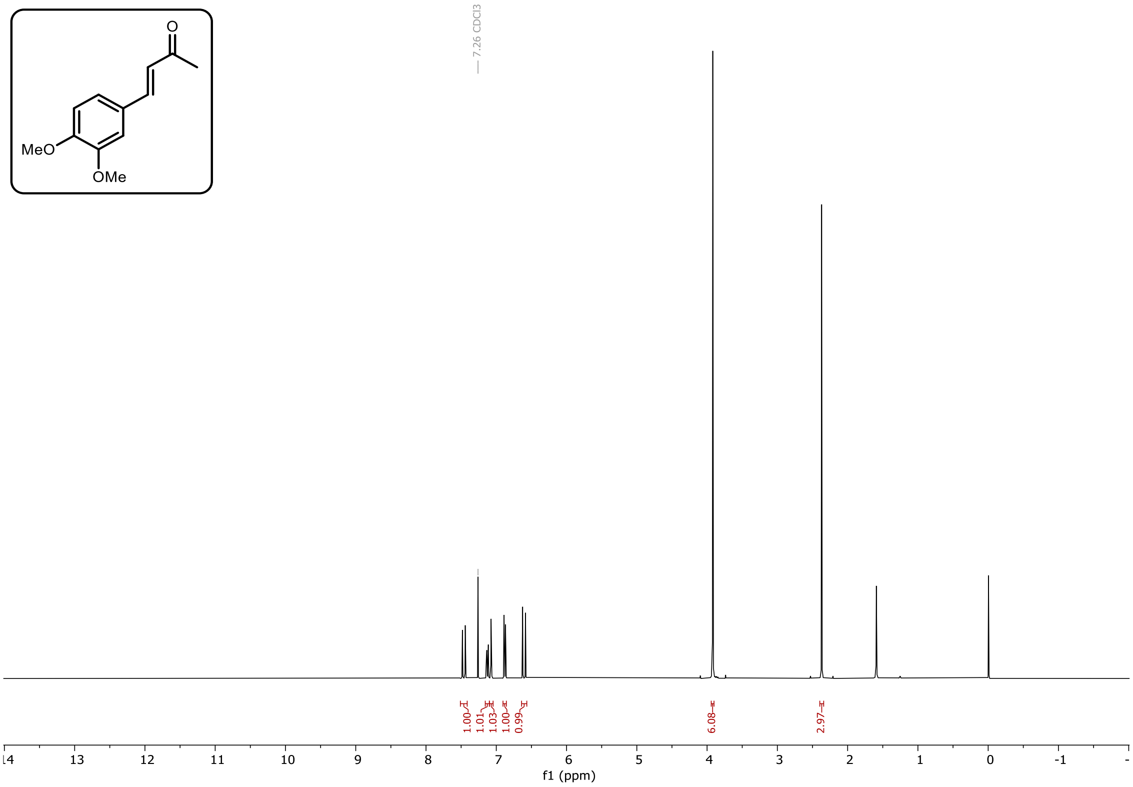

A27: *^{1}H NMR (CDCl$_3$) of compound* ***S10***.

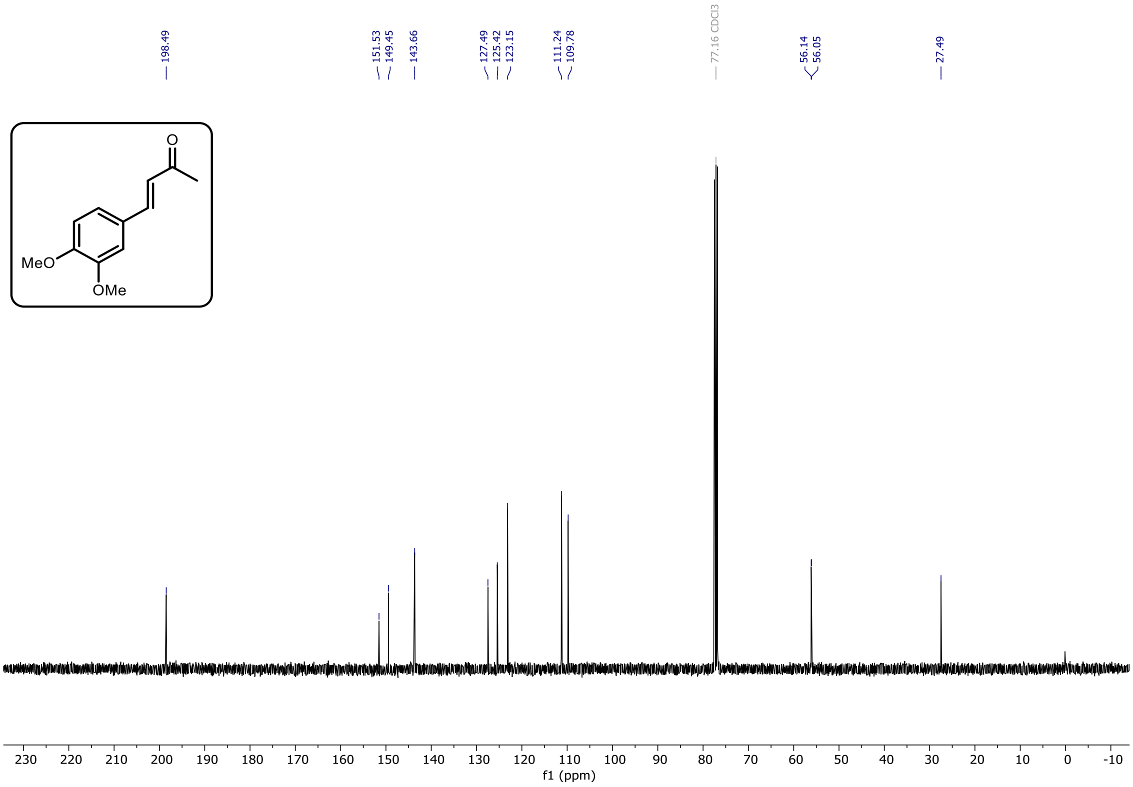

A28: *^{13}C NMR (CDCl$_3$) of compound* ***S10***.

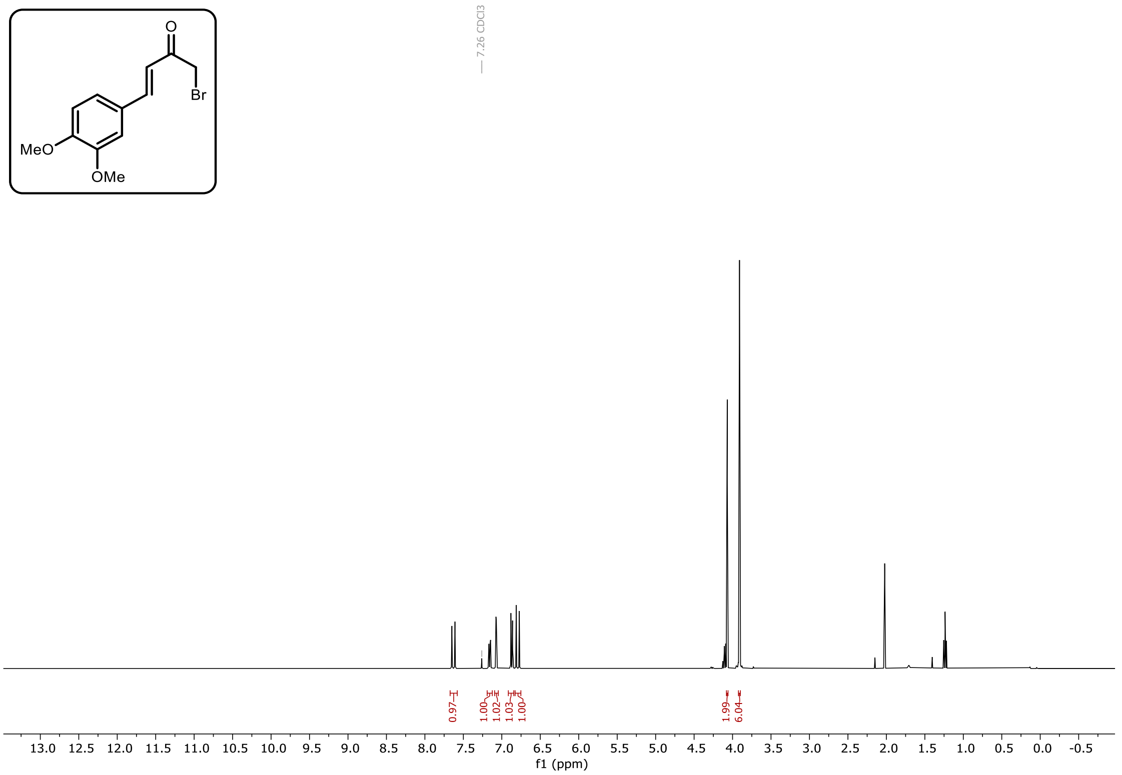

A29: ^{1}H *NMR (CDCl$_3$) of compound* ***S11***.

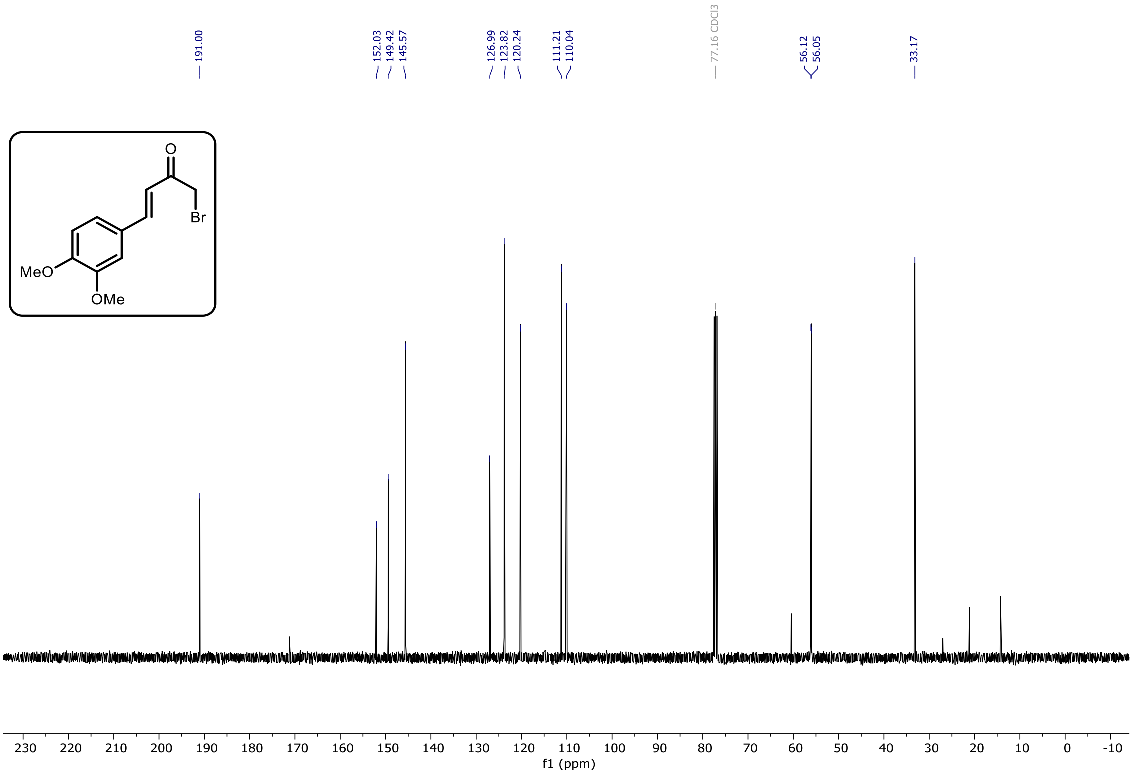

A30: ^{13}C *NMR (CDCl$_3$) of compound* ***S11***.

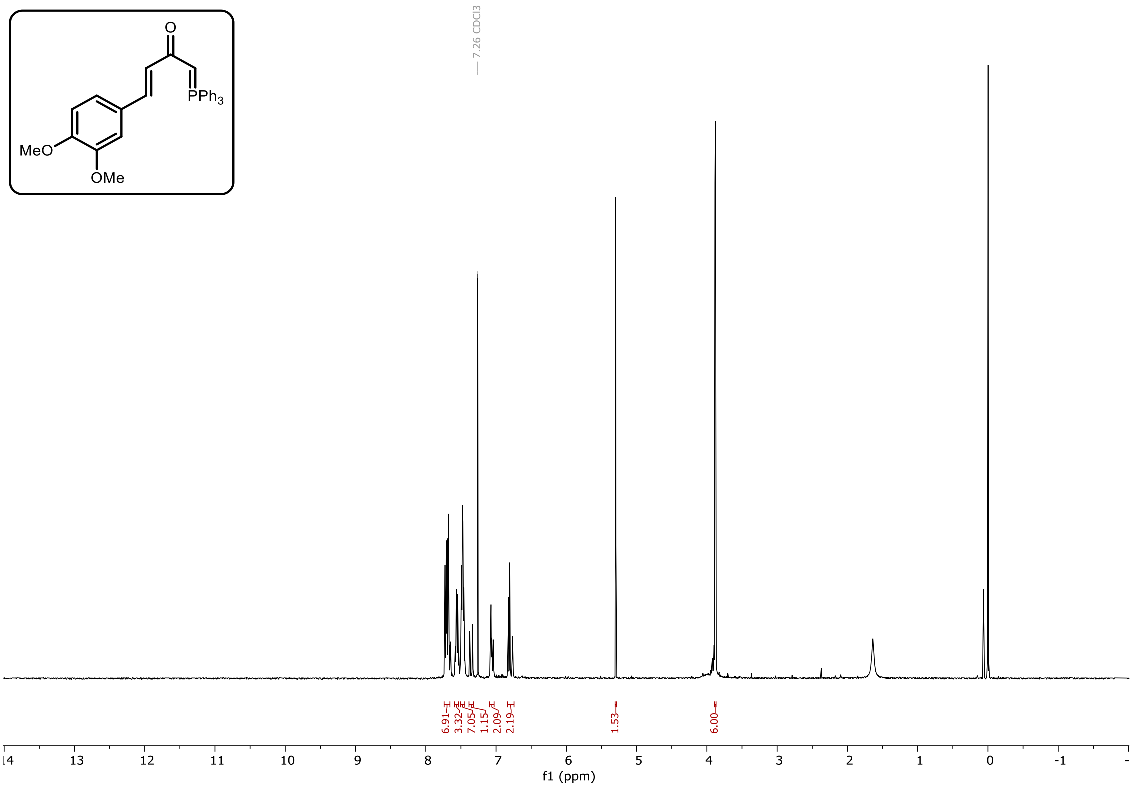

A31: *^{1}H NMR ($CDCl_3$) of compound* ***S12*.**

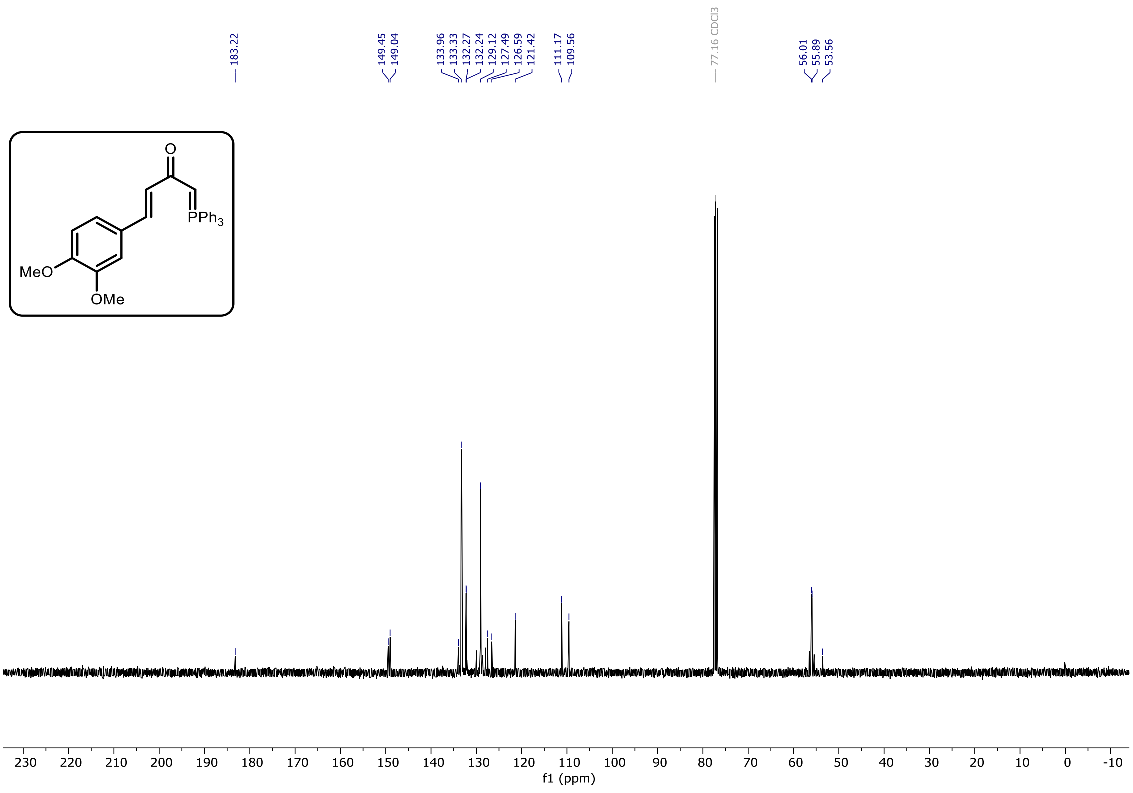

A32: *^{13}C NMR ($CDCl_3$) of compound* ***S12*.**

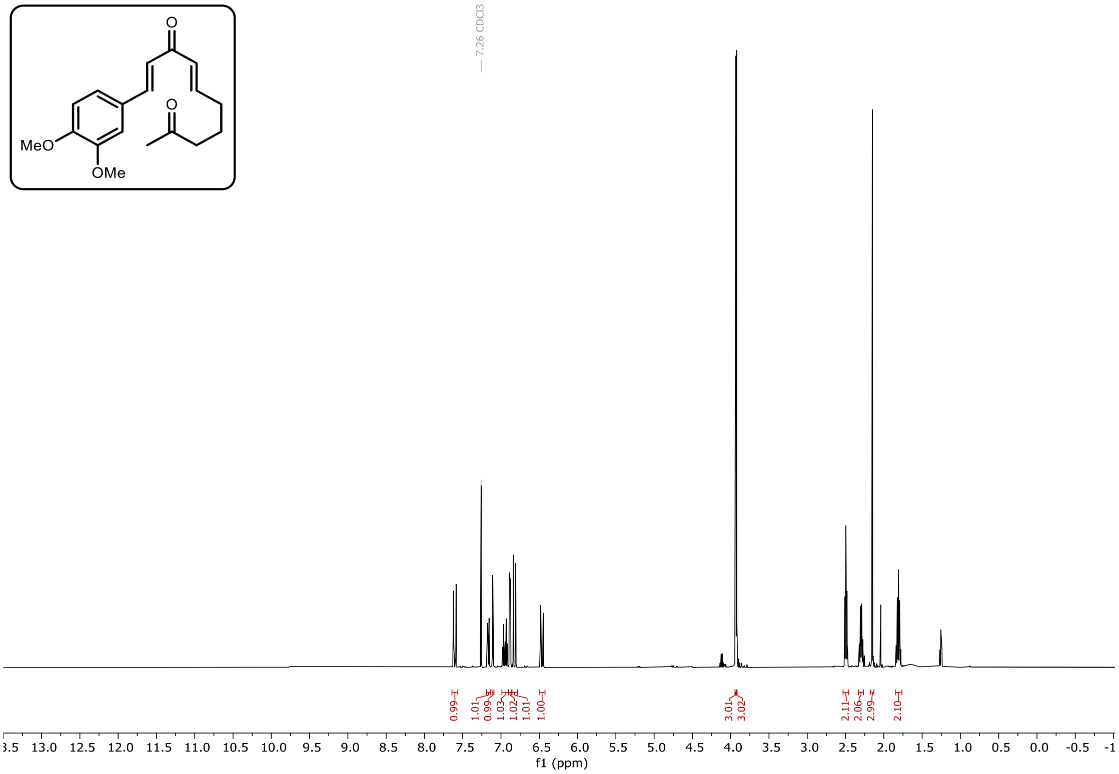

A33: *^{1}H NMR (CDCl$_3$) of compound* ***1e****.*

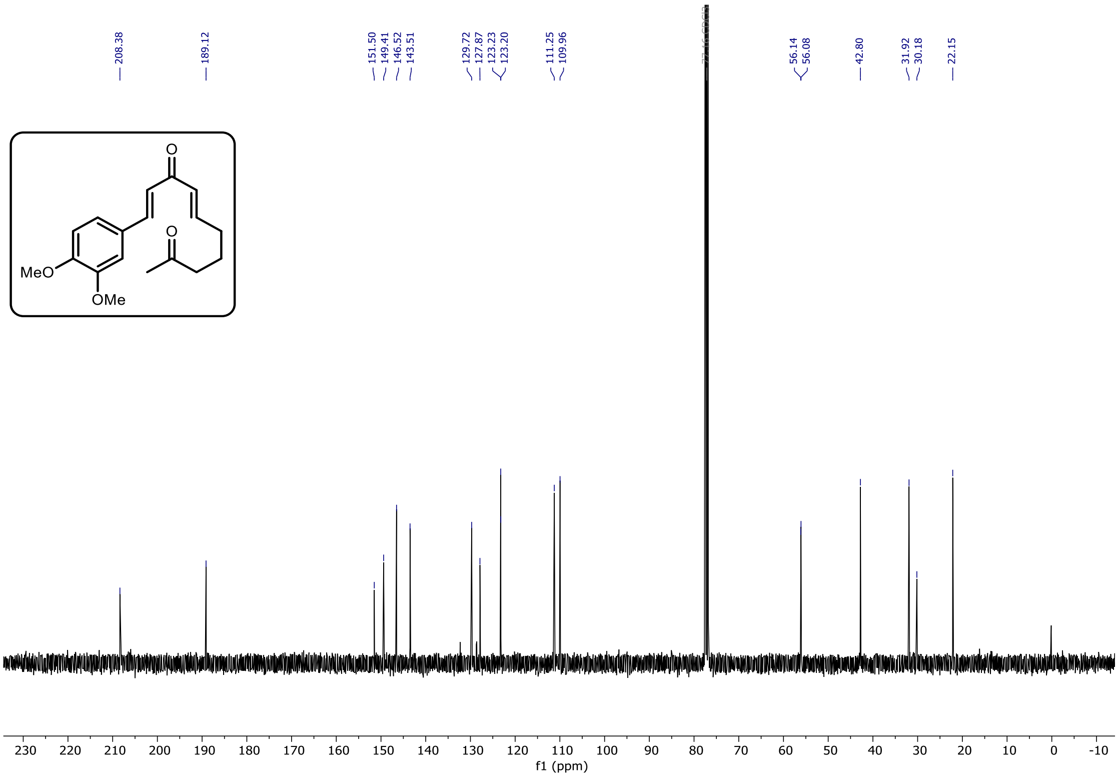

A34: *^{13}C NMR (CDCl$_3$) of compound* ***1e****.*

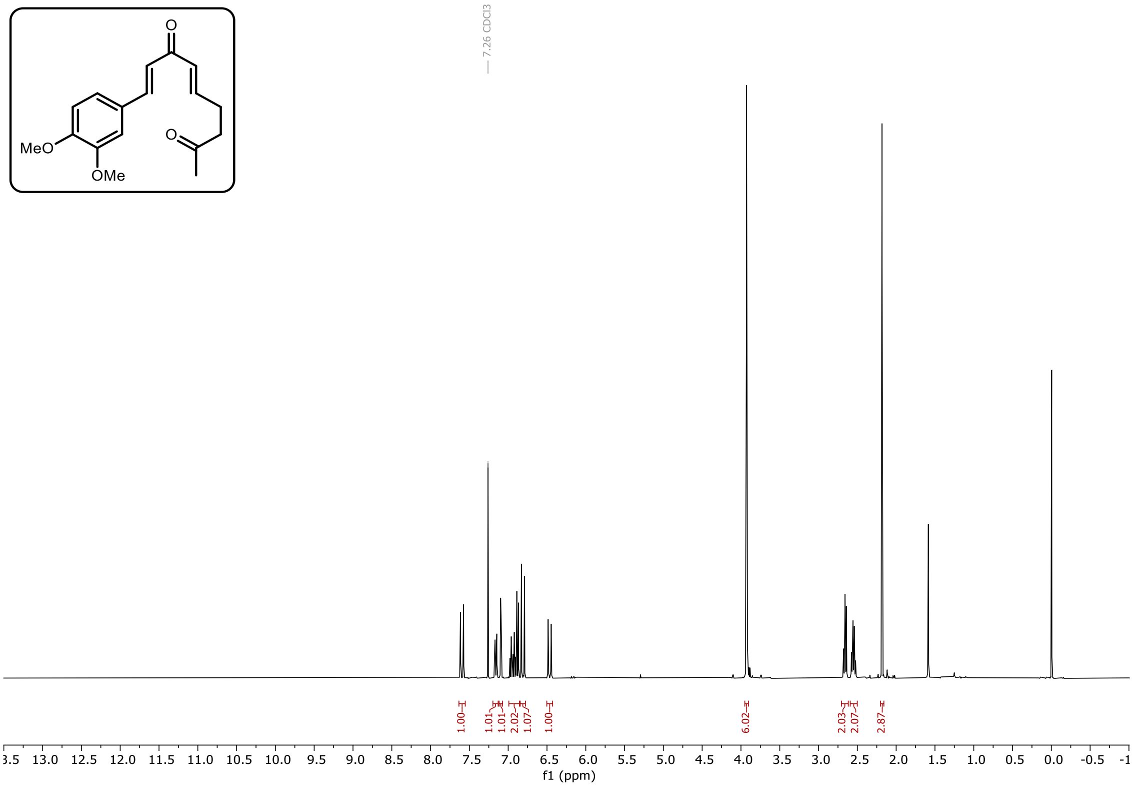

A35: *¹H NMR (CDCl$_3$) of compound* ***1f*****.**

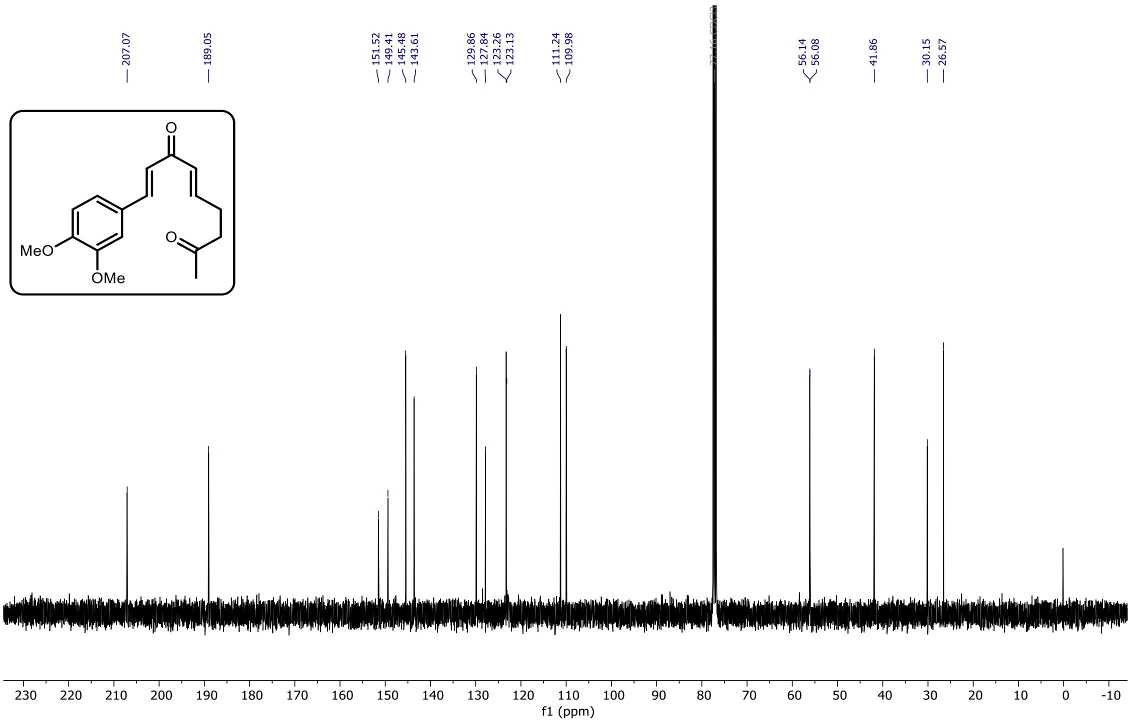

A36: *¹³C NMR (CDCl$_3$) of compound* ***1f*****.**

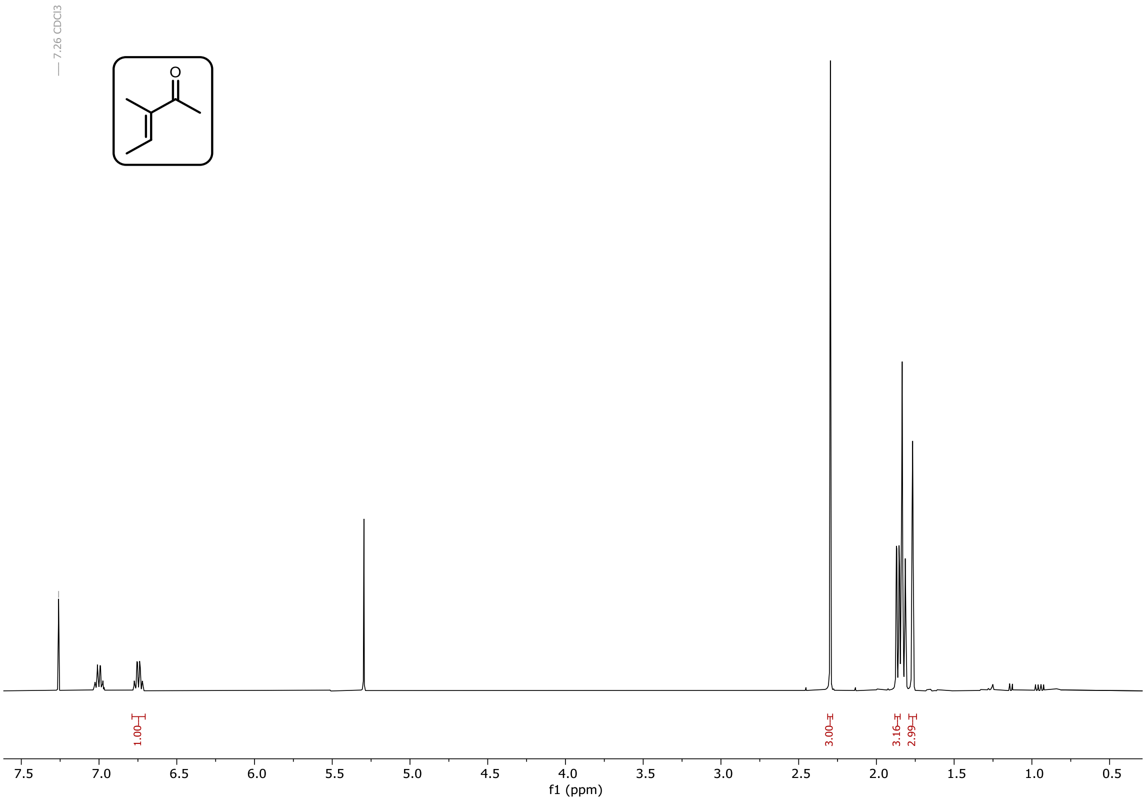

A37: *^{1}H NMR ($CDCl_3$) of compound* ***S13***.

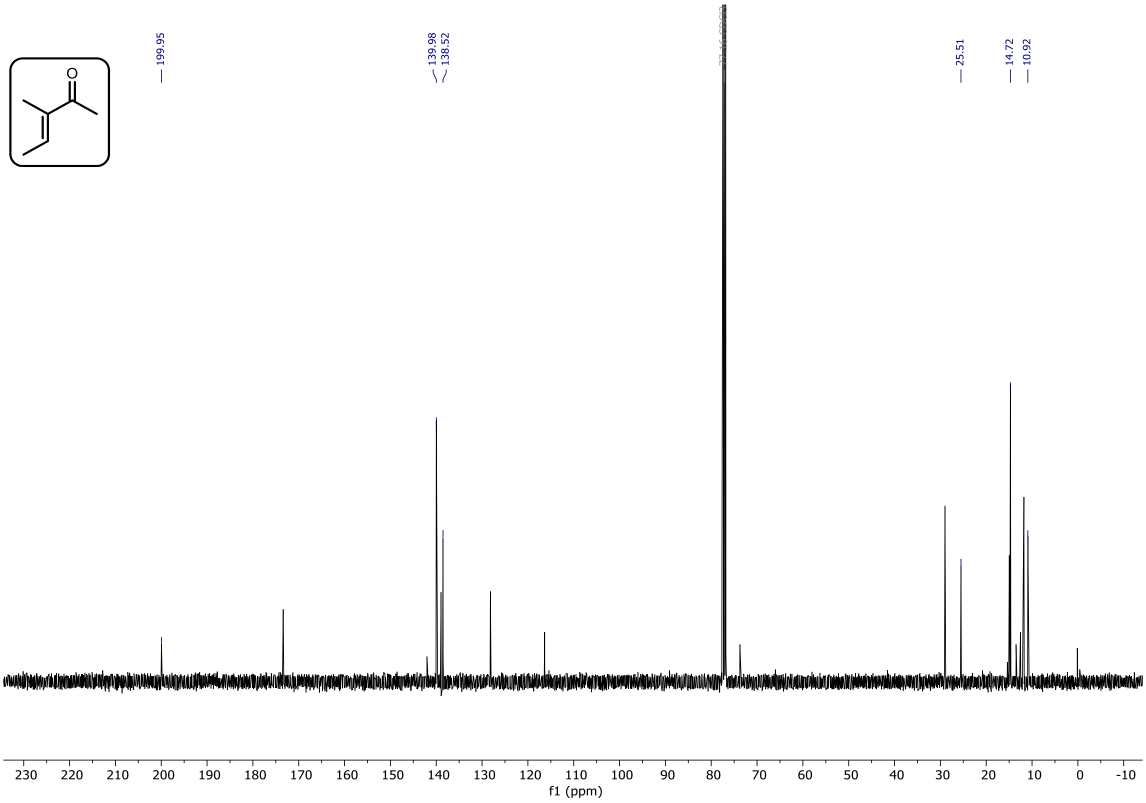

A38: *^{13}C NMR ($CDCl_3$) of compound* ***S13***.

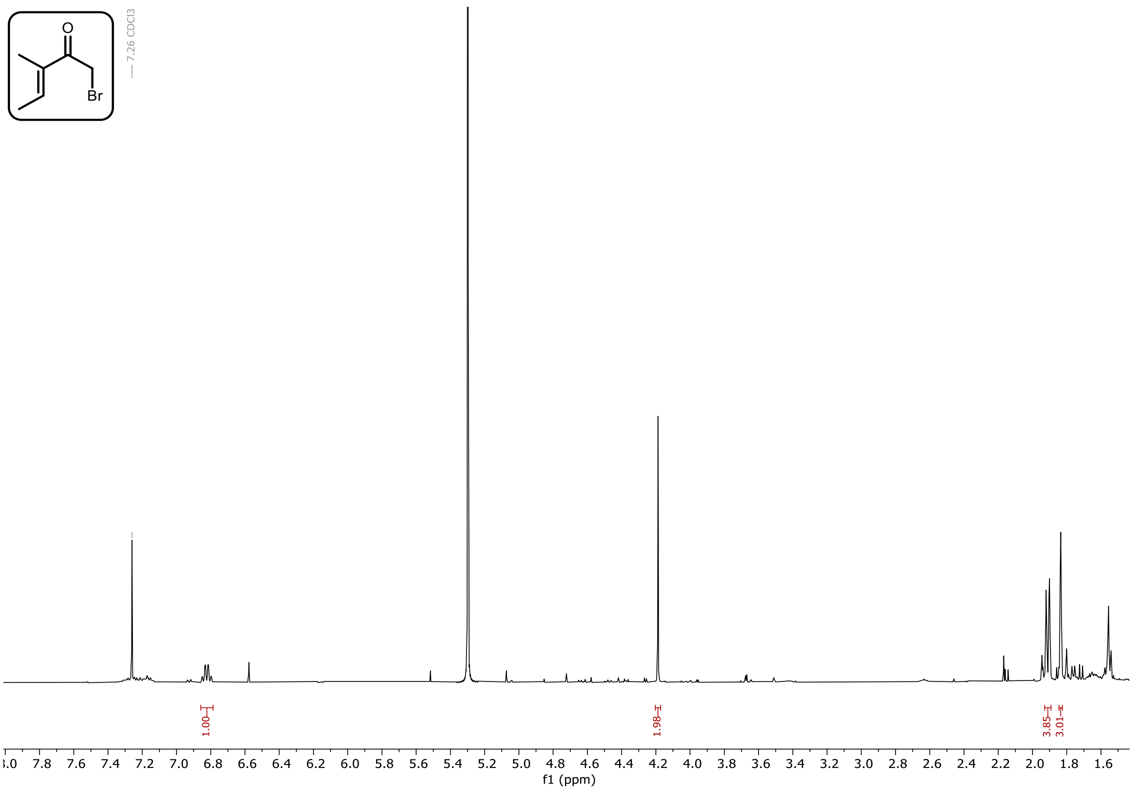

A39: *^{1}H NMR ($CDCl_3$) of compound* ***S14****.*

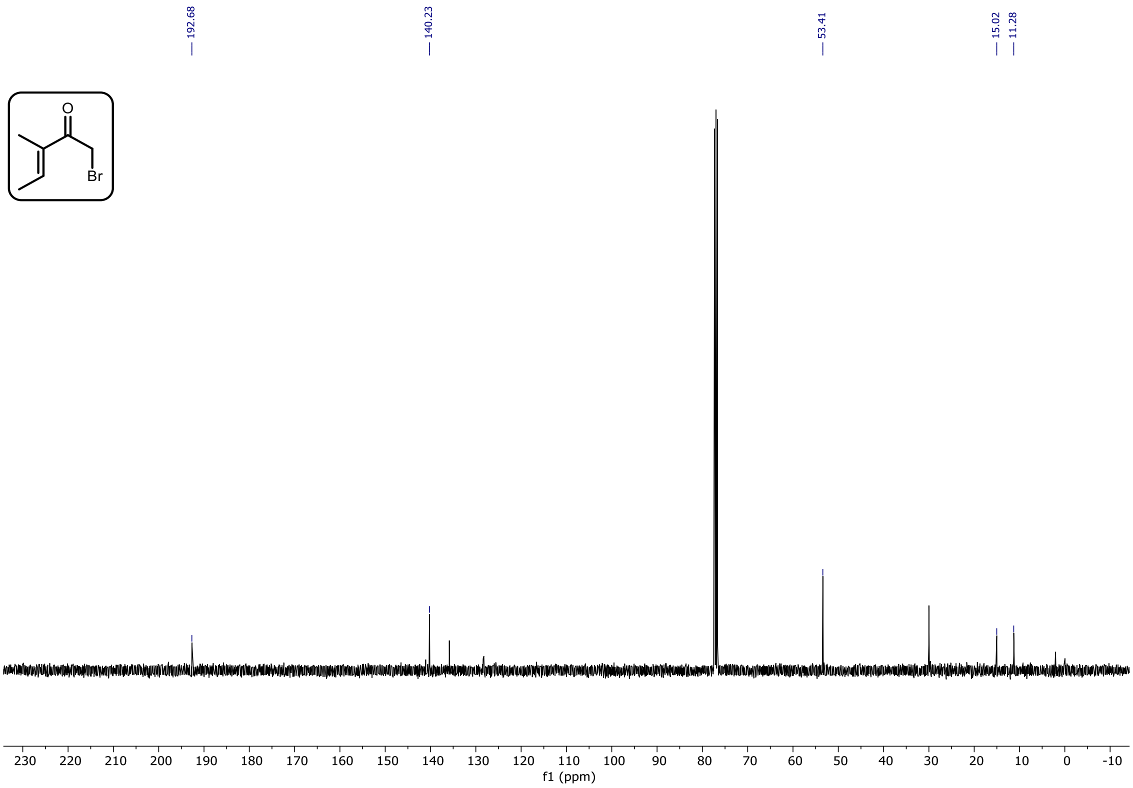

A40: *^{13}C NMR ($CDCl_3$) of compound* ***S14****.*

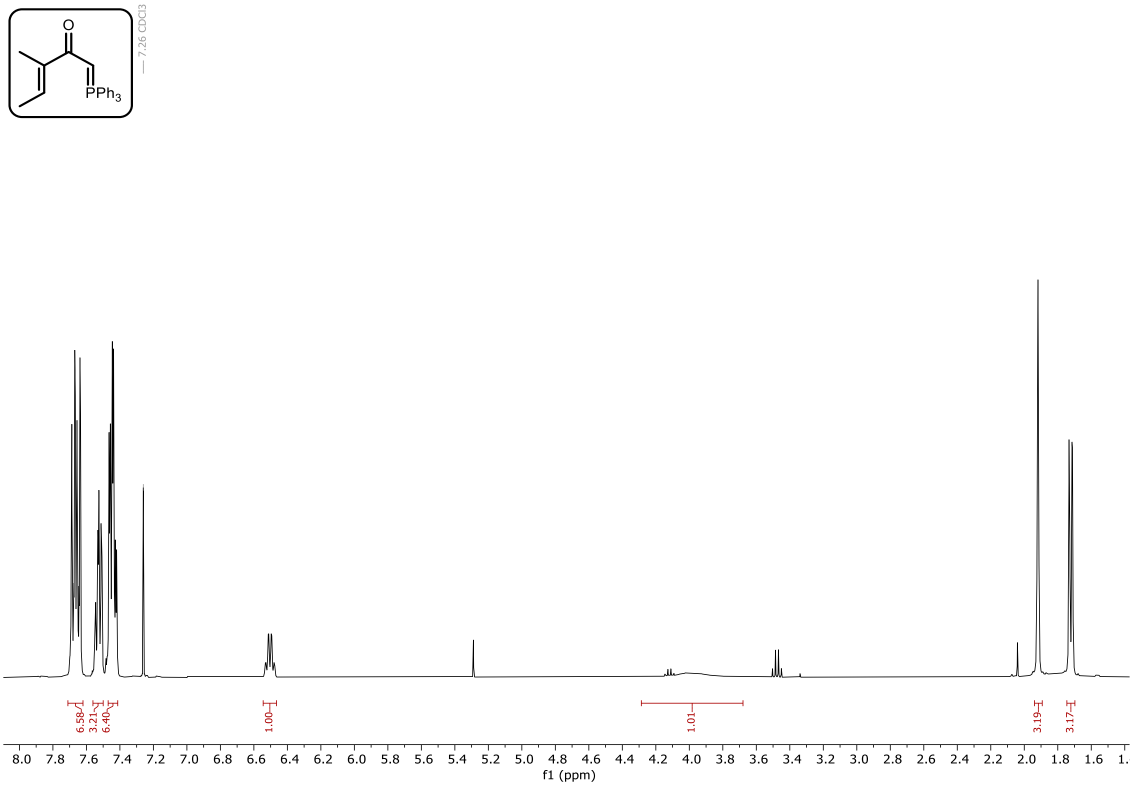

A41: *^{1}H NMR ($CDCl_3$) of compound **S15**.*

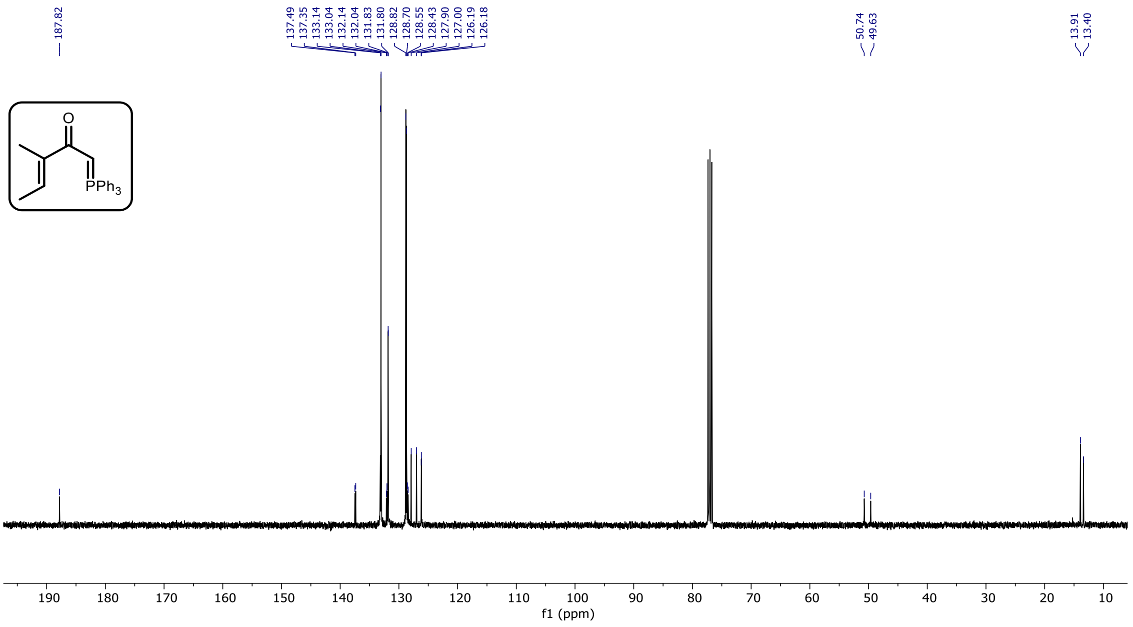

A42: *^{13}C NMR ($CDCl_3$) of compound **S15**.*

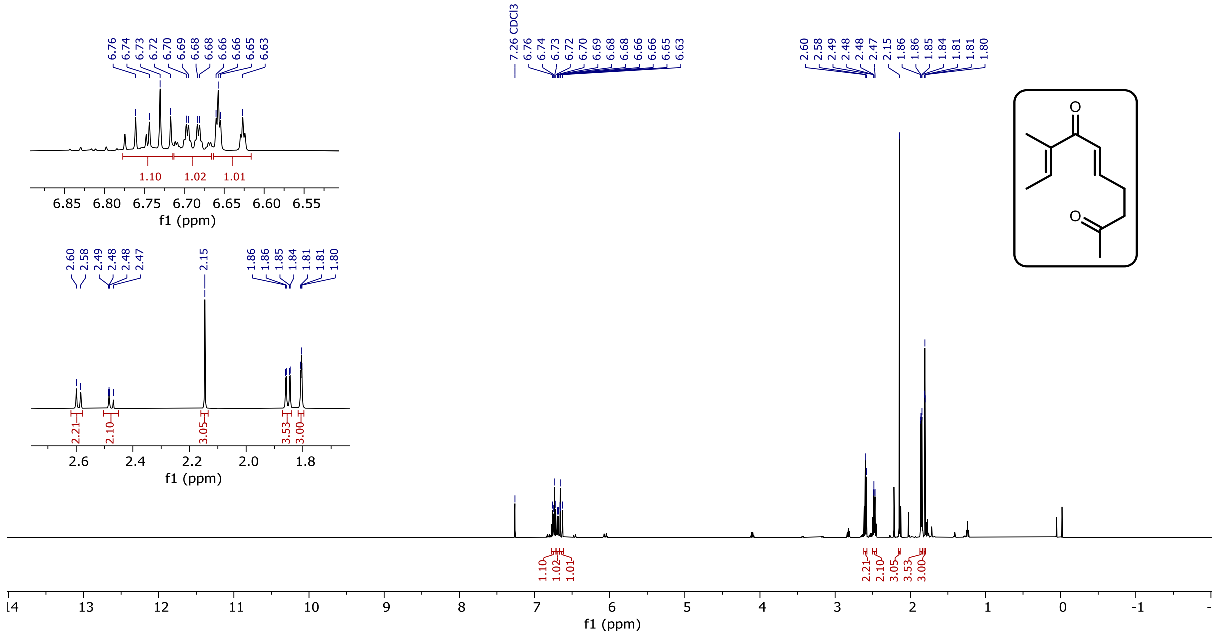

A43: *¹H NMR (CDCl$_3$) of compound* ***1g***.

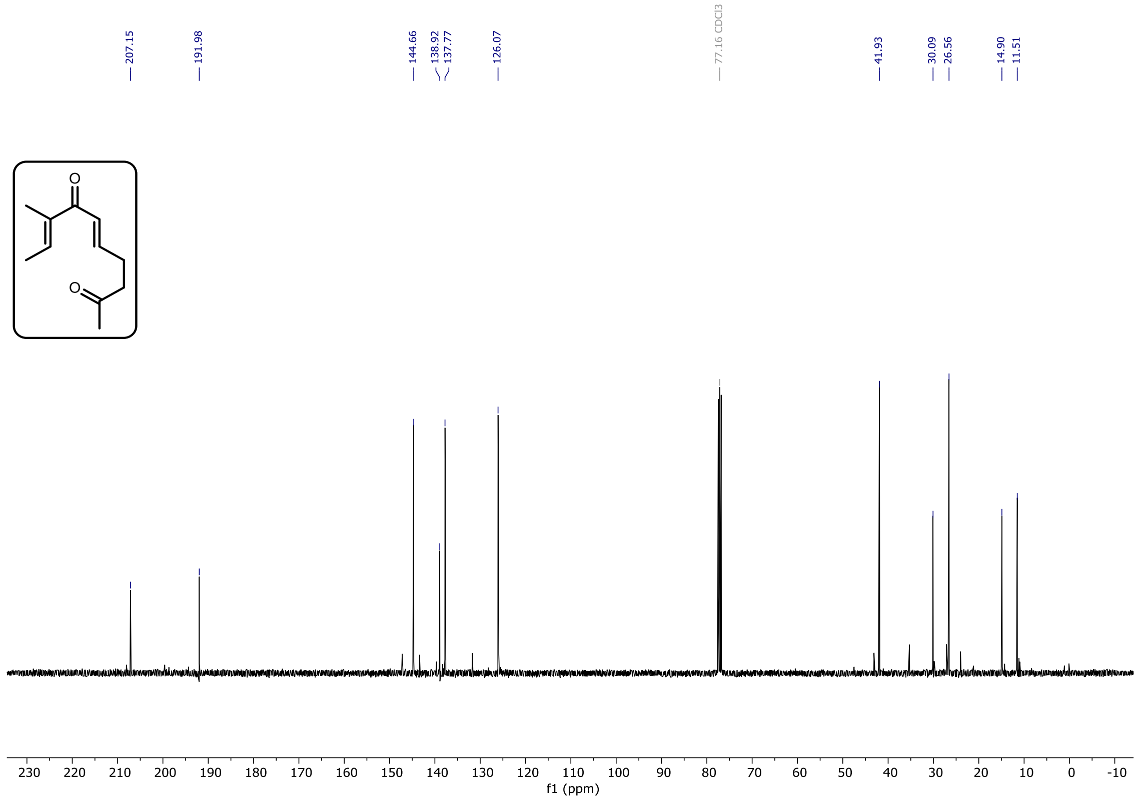

A44: *¹³C NMR (CDCl$_3$) of compound* ***1g***.

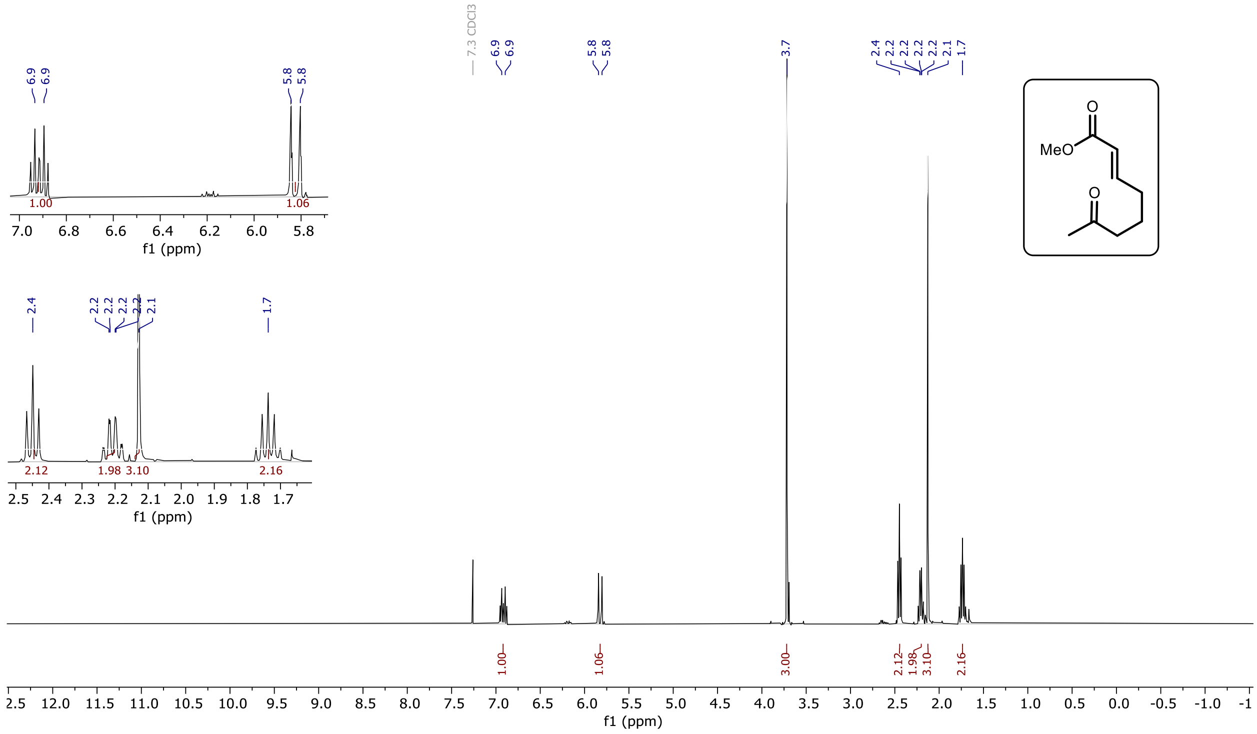

A45: *^{1}H NMR ($CDCl_3$) of compound* ***S16.***

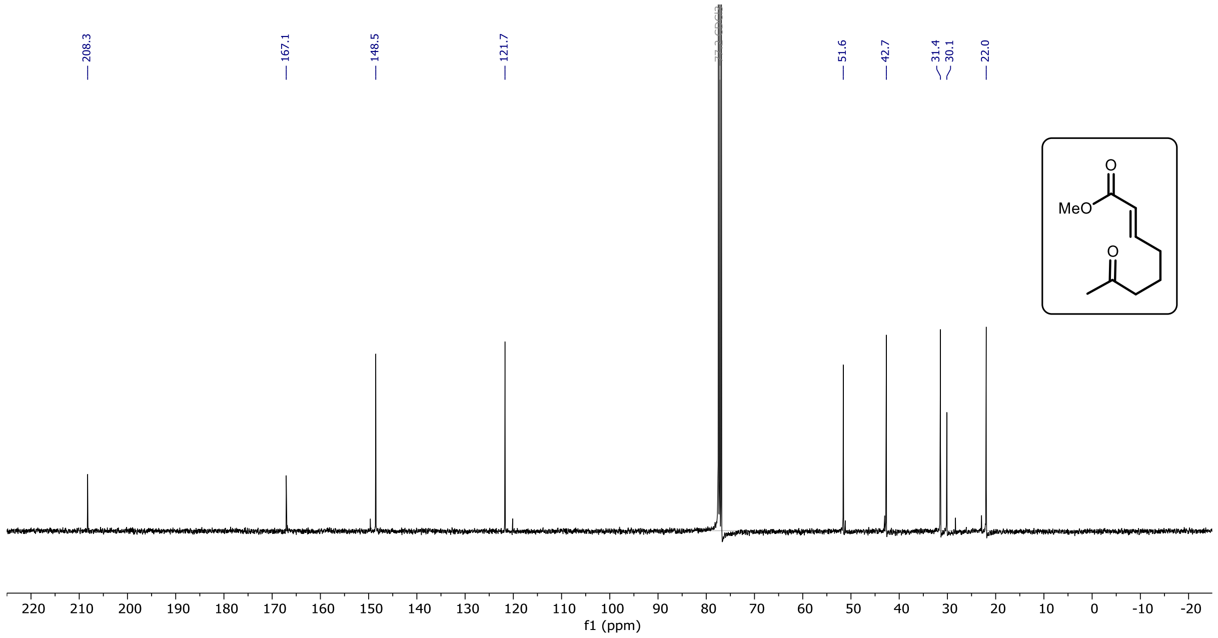

A46: *^{13}C NMR ($CDCl_3$) of compound* ***S16***.

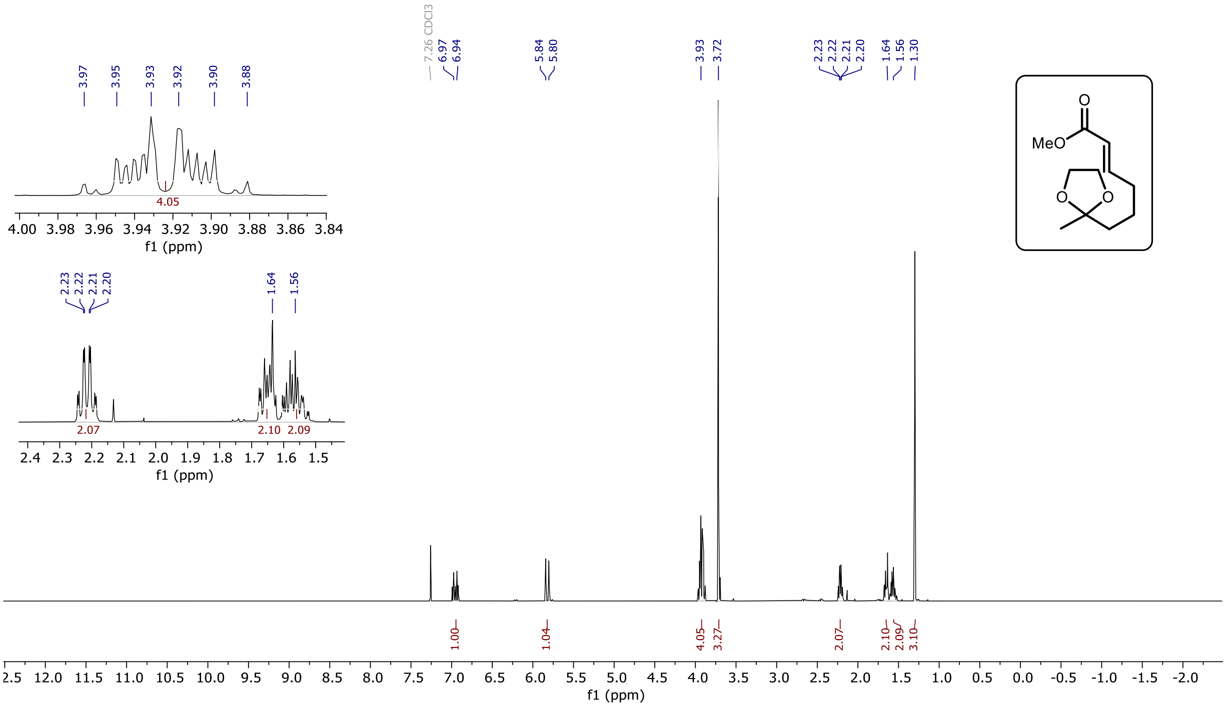

A47: *^{1}H NMR (CDCl$_3$) of compound* ***S17.***

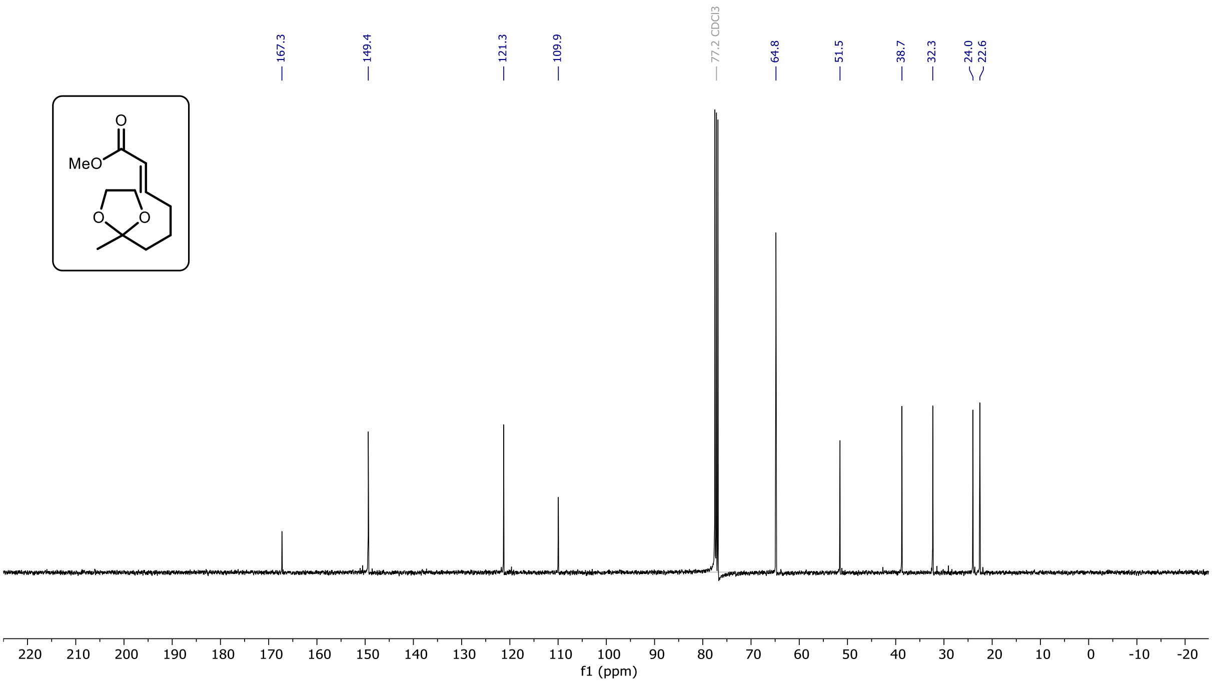

A48: ^{13}C NMR (CDCl$_3$) of compound **S17**.

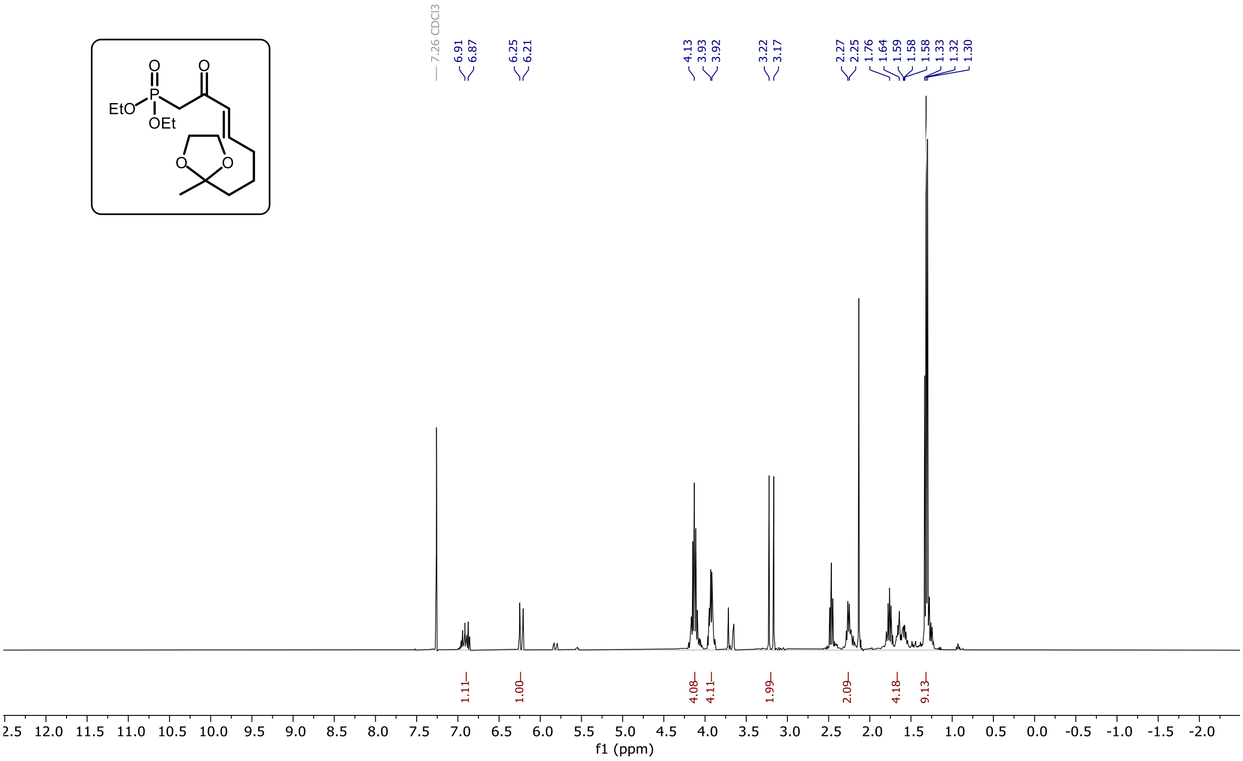

A49: *^{1}H NMR ($CDCl_3$) of compound* ***S18.***

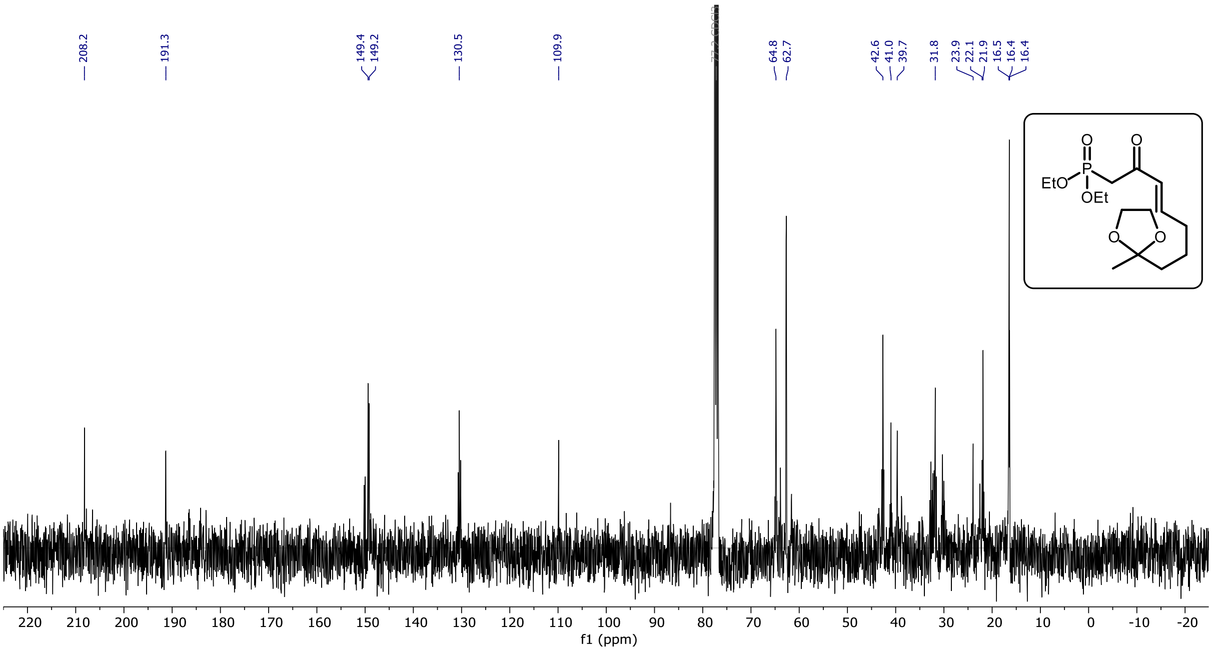

A50: ^{13}C NMR ($CDCl_3$) of compound **S18**.

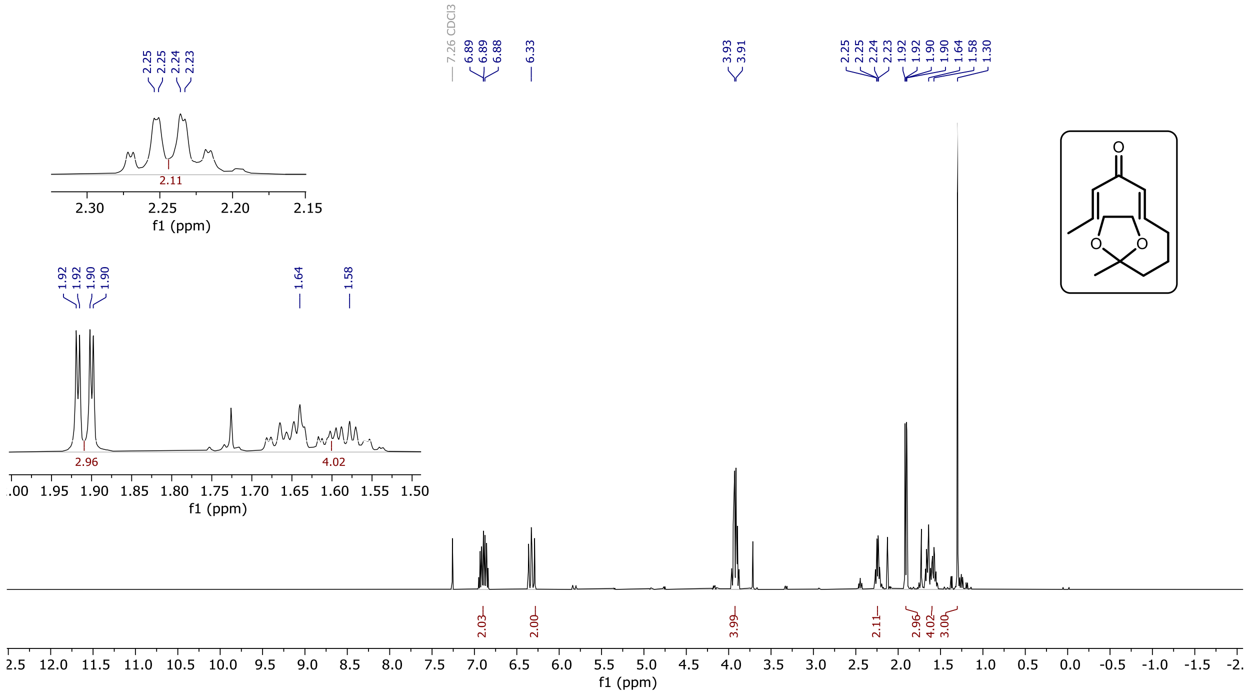

A51:** ^{1}H NMR (CDCl$_3$) of compound **S19.

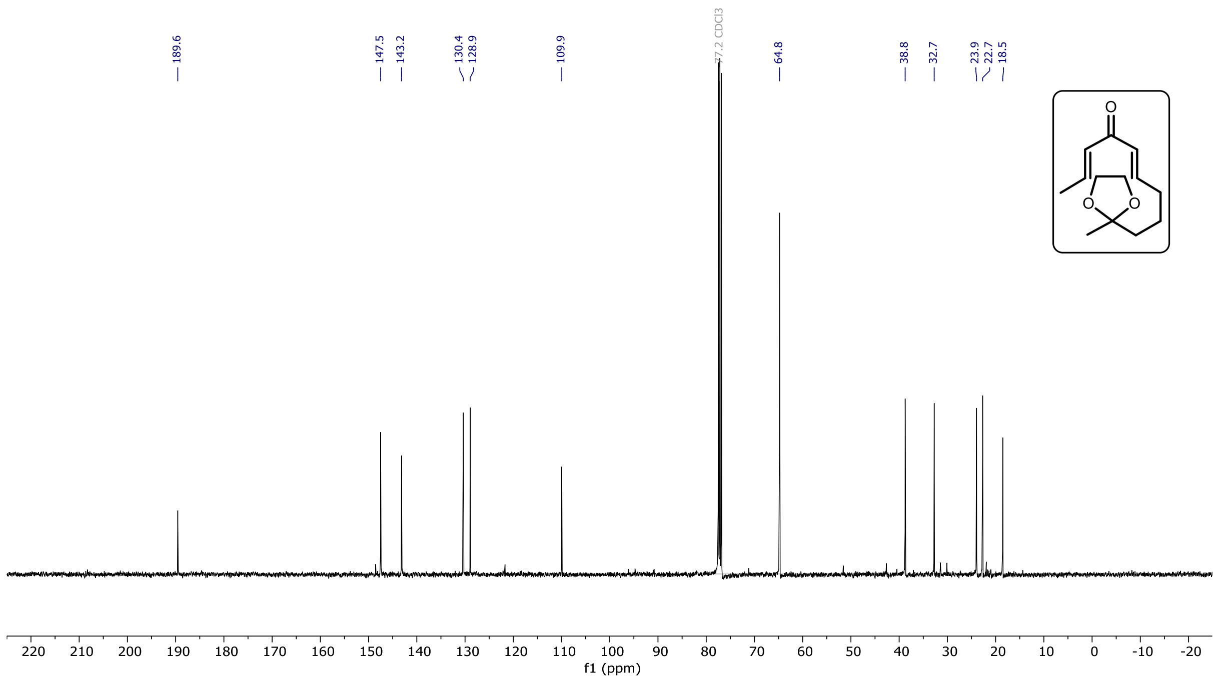

A52: ^{13}C NMR (CDCl$_3$) of compound **S19**.

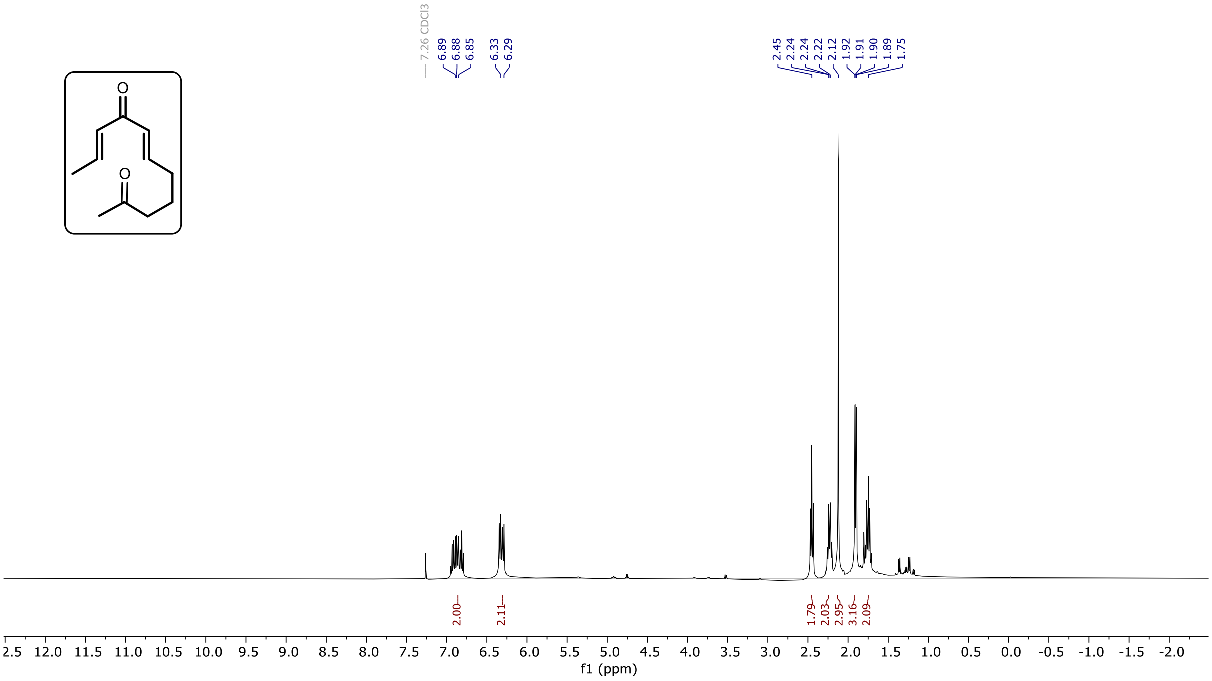

A53: *^{1}H NMR ($CDCl_3$) of compound* ***1h.***

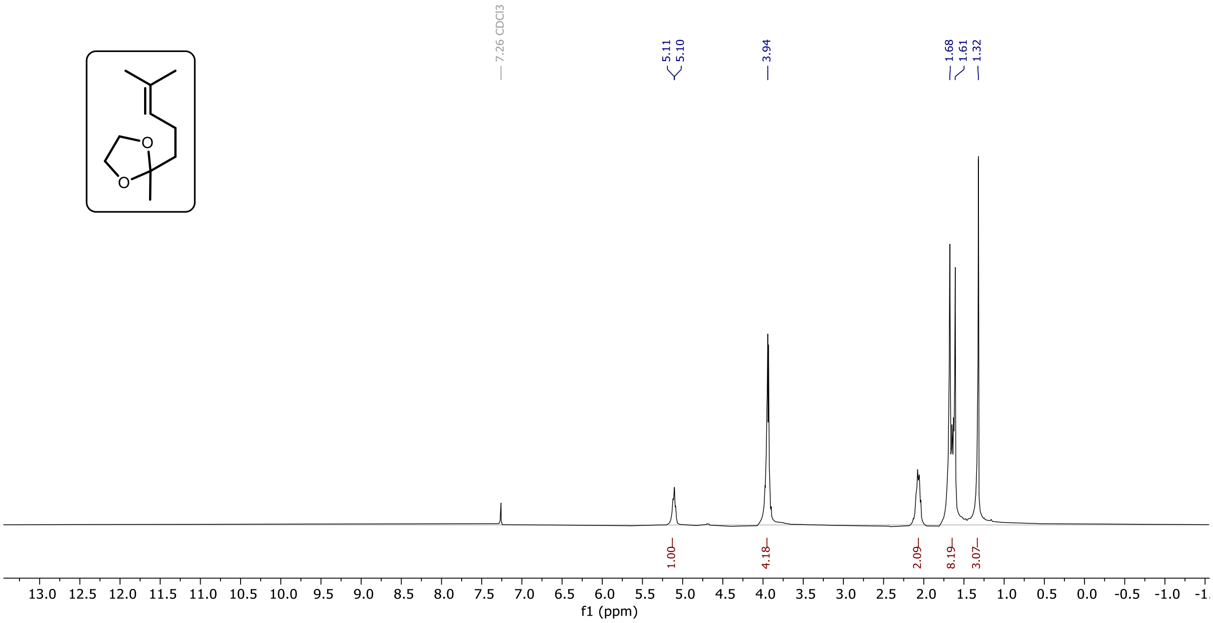

A54: *^{1}H NMR ($CDCl_3$) of compound* ***S20.***

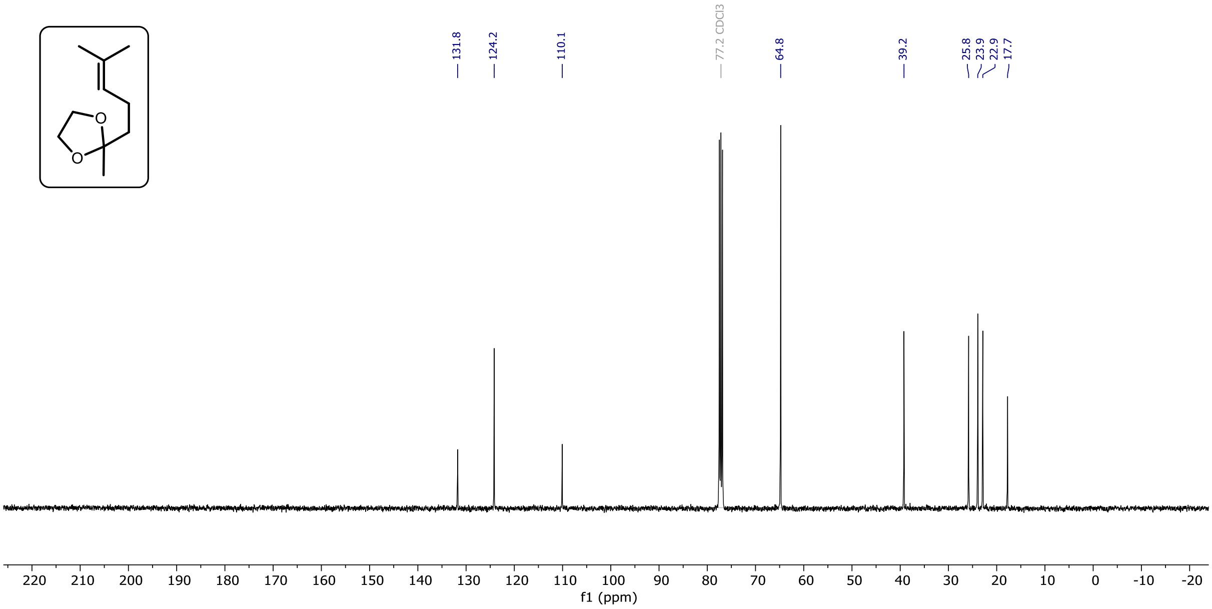

A55: ^{13}C NMR ($CDCl_3$) of compound **S20**.

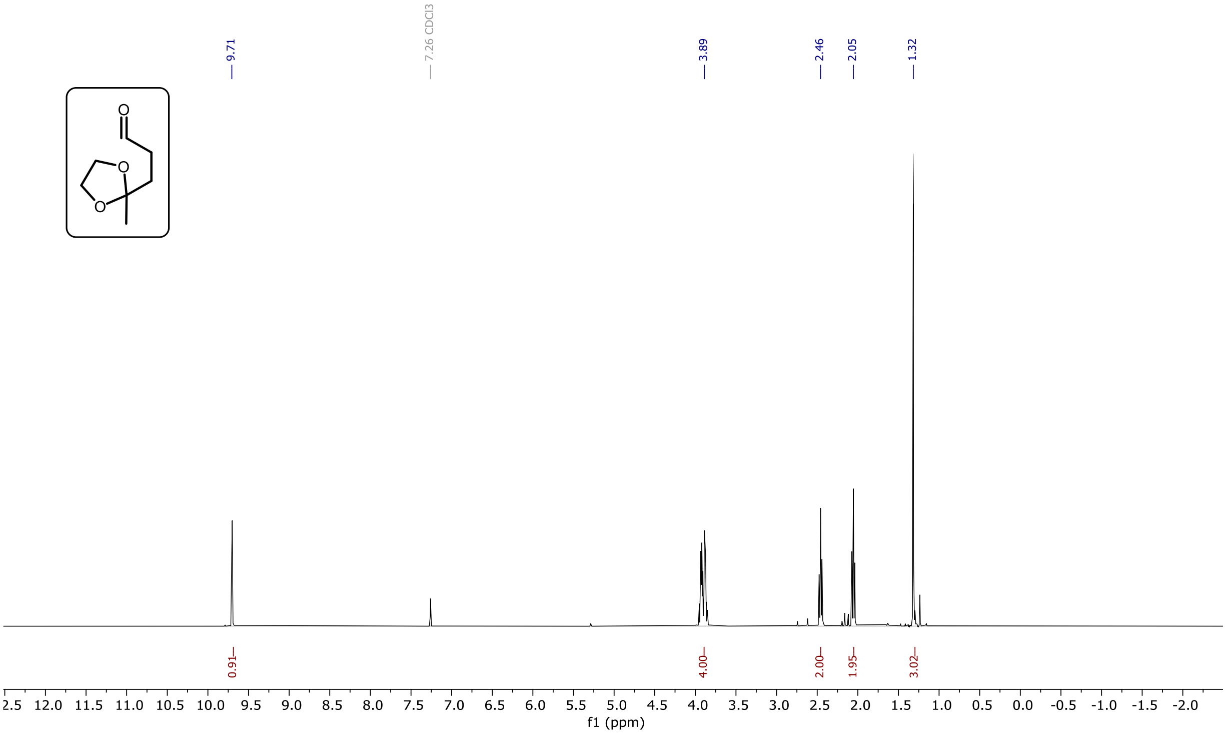

A56: *^{1}H NMR ($CDCl_3$) of compound* ***S21.***

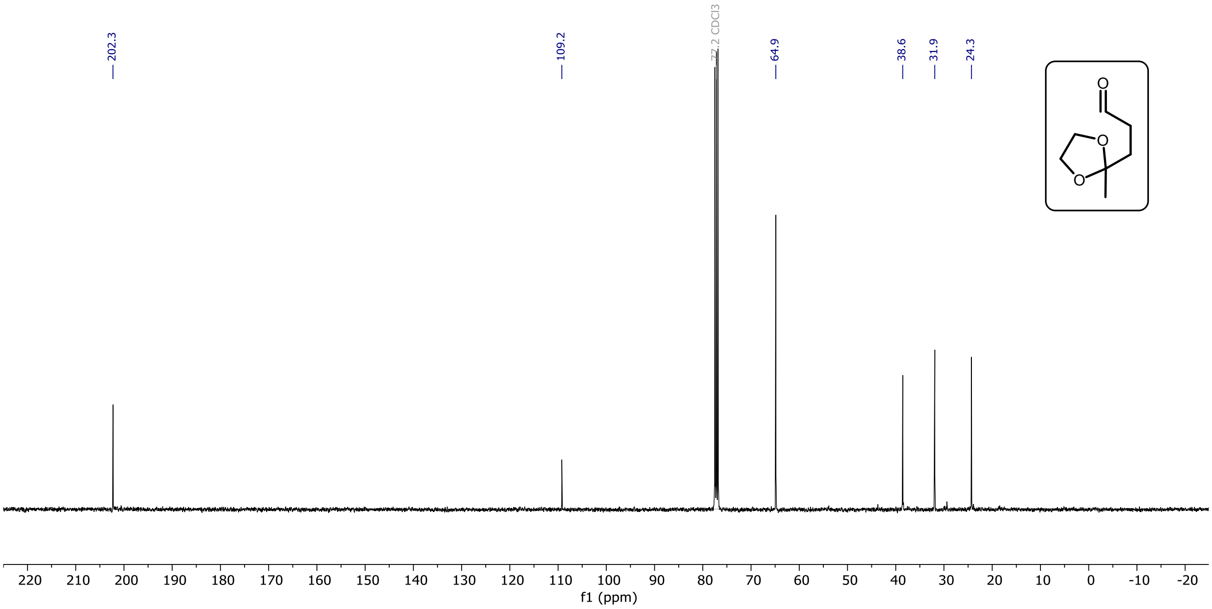

A57: ^{13}C NMR ($CDCl_3$) of compound **S21**.

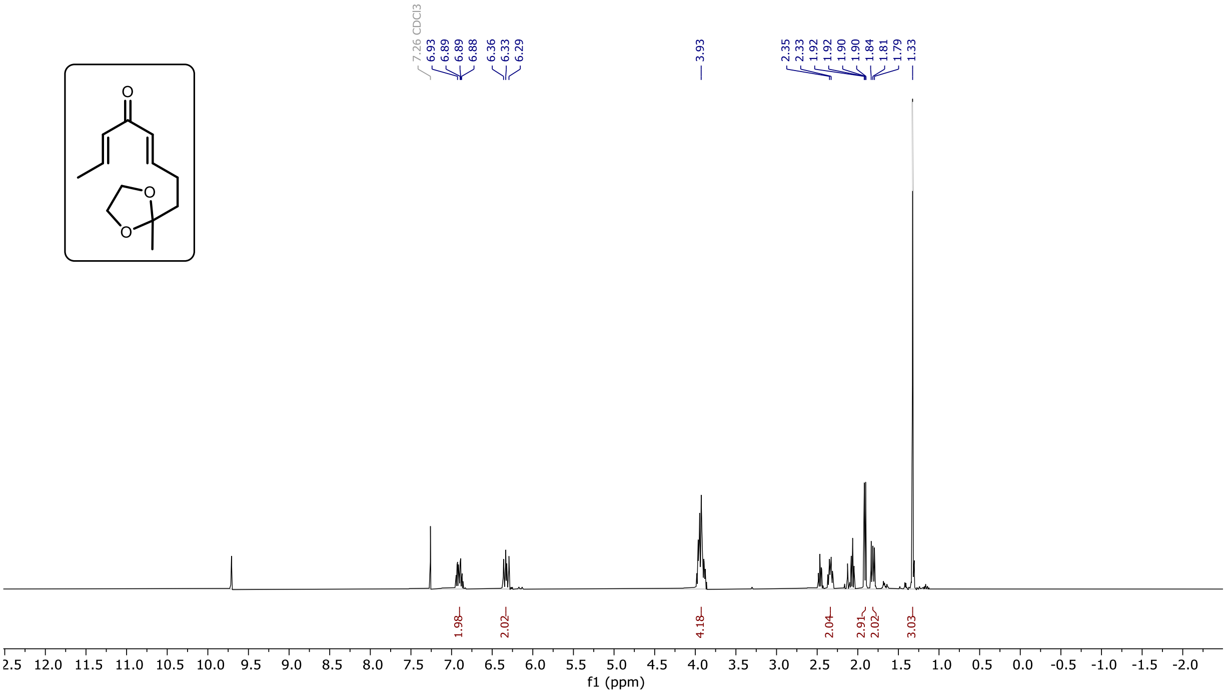

A58: *^{1}H NMR ($CDCl_3$) of compound* ***S22.***

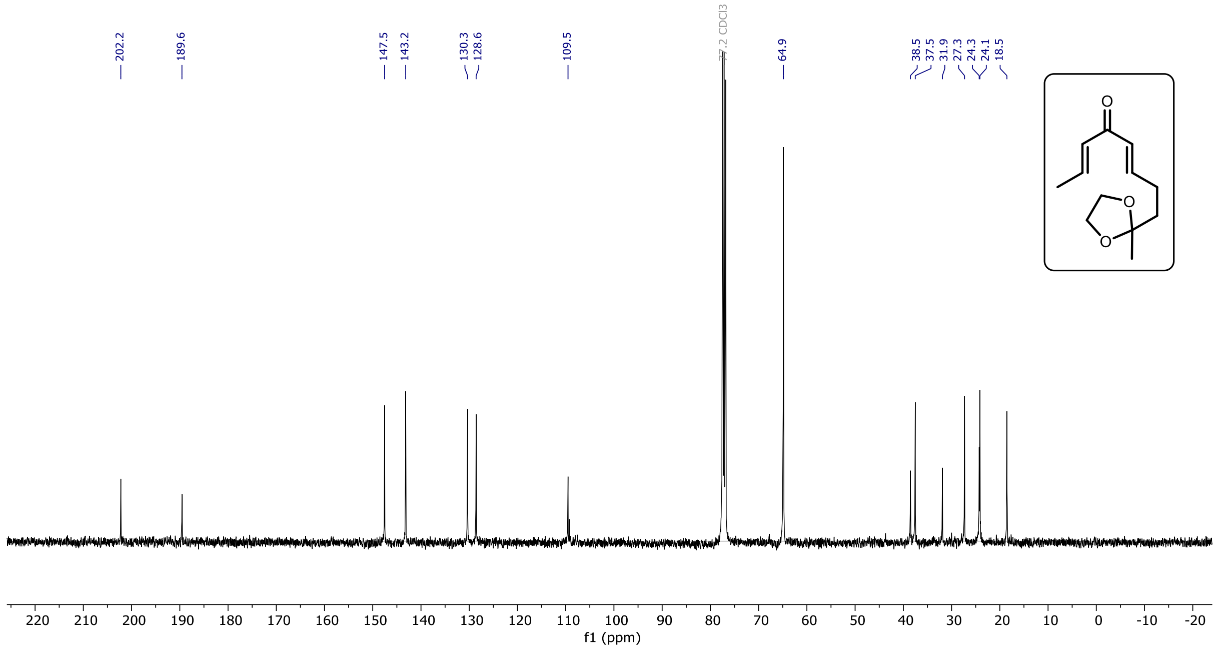

A59: ^{13}C NMR ($CDCl_3$) of compound **S22**.

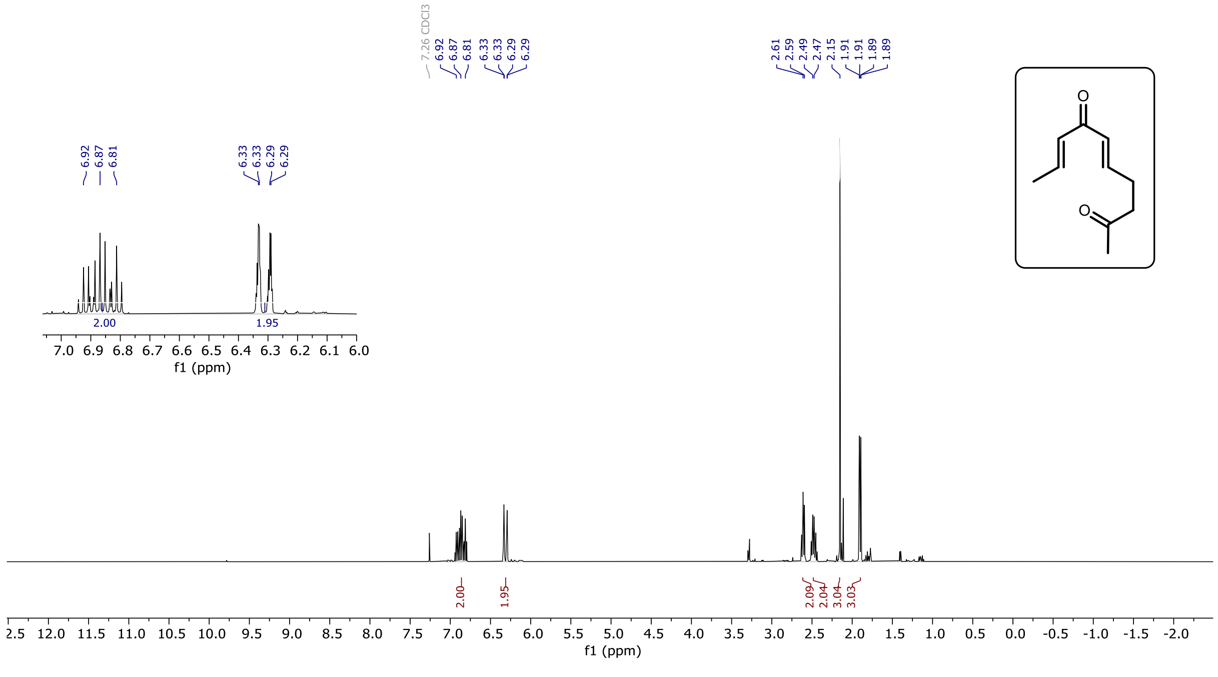

***A60:** ^{1}H NMR ($CDCl_3$) of compound **1i**.*

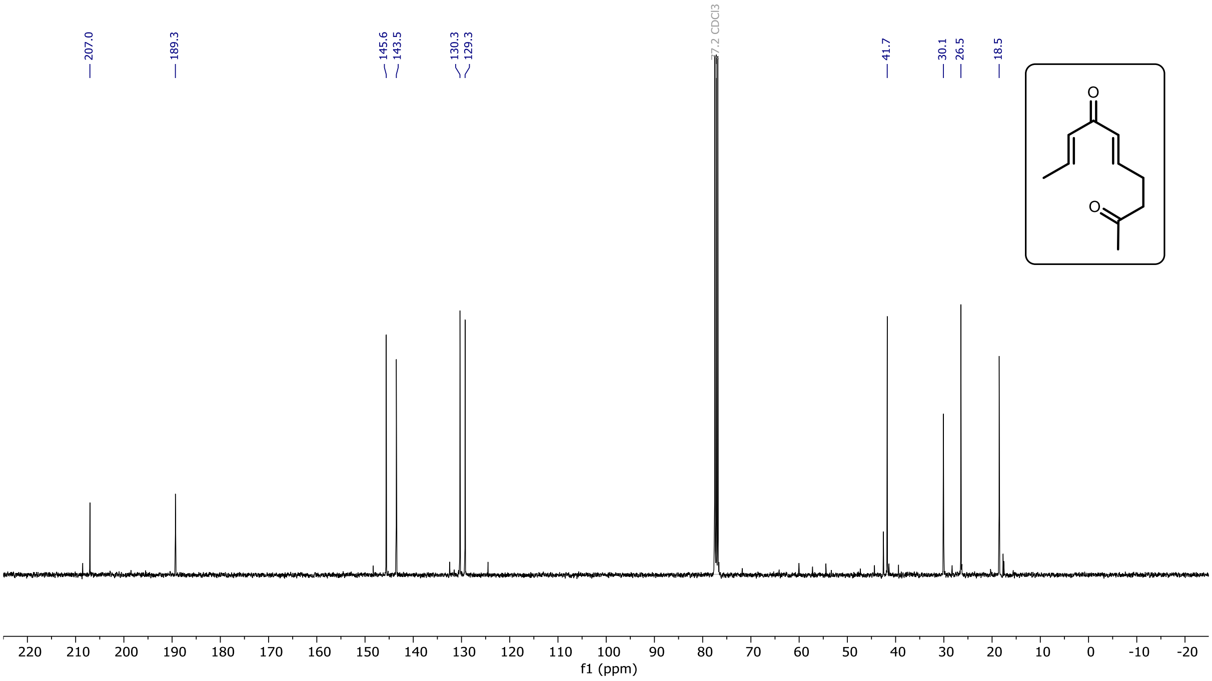

A61: ^{13}C NMR ($CDCl_3$) of compound **1i**.

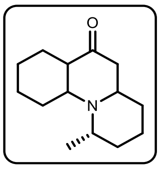

Crude reaction mixture: 4 stereoisomers

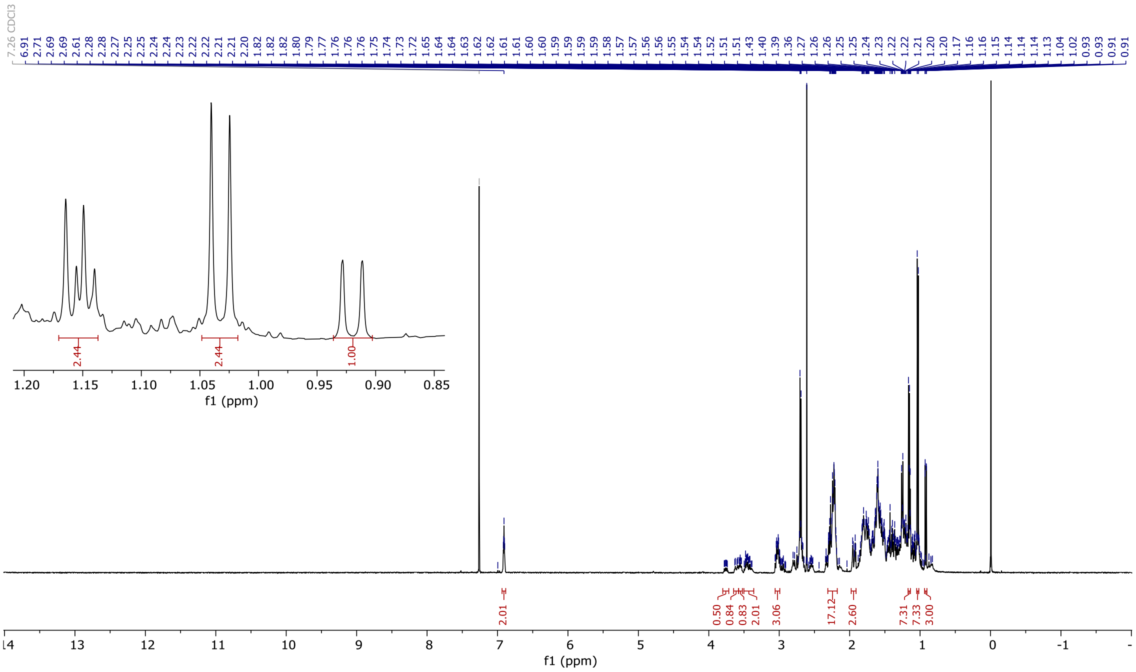

***A62:** ^{1}H NMR ($CDCl_3$) of compounds (S,S)-**3a** and (1S)-**4a**.*

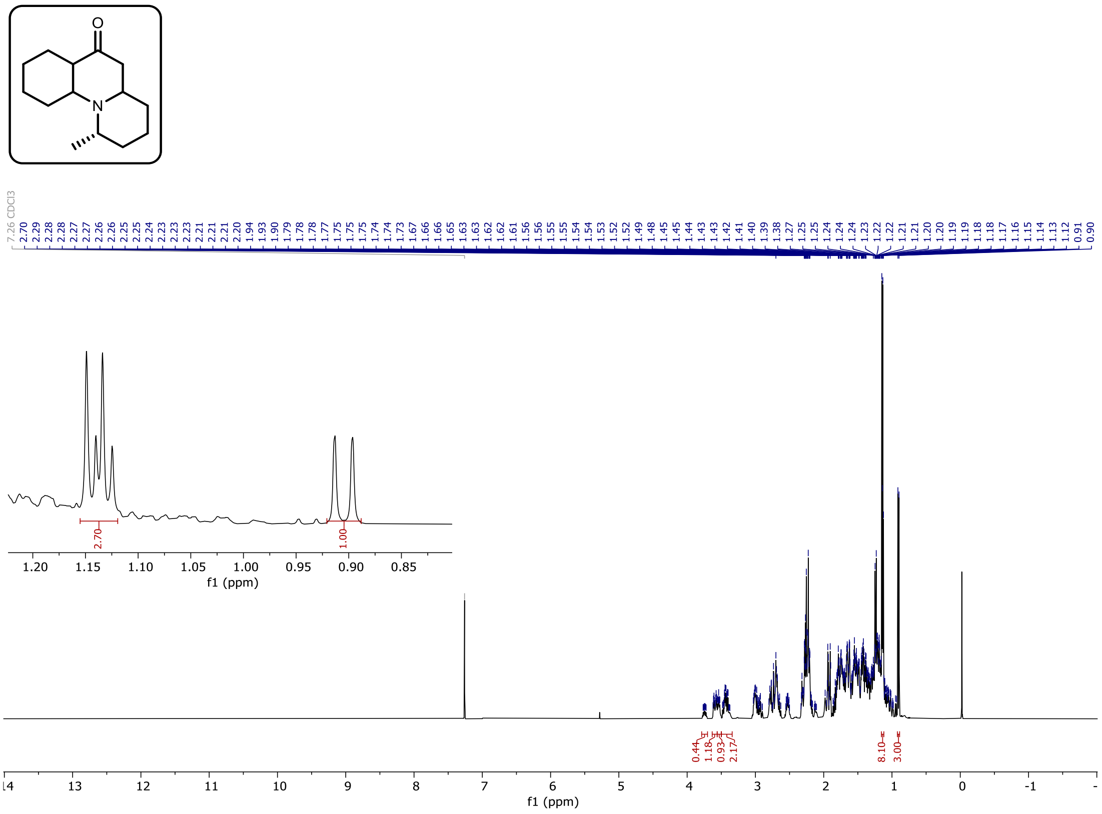

A63: *^{1}H NMR ($CDCl_3$) of compounds* ***(1S)-4a*** *(single IMAMR product removed).*

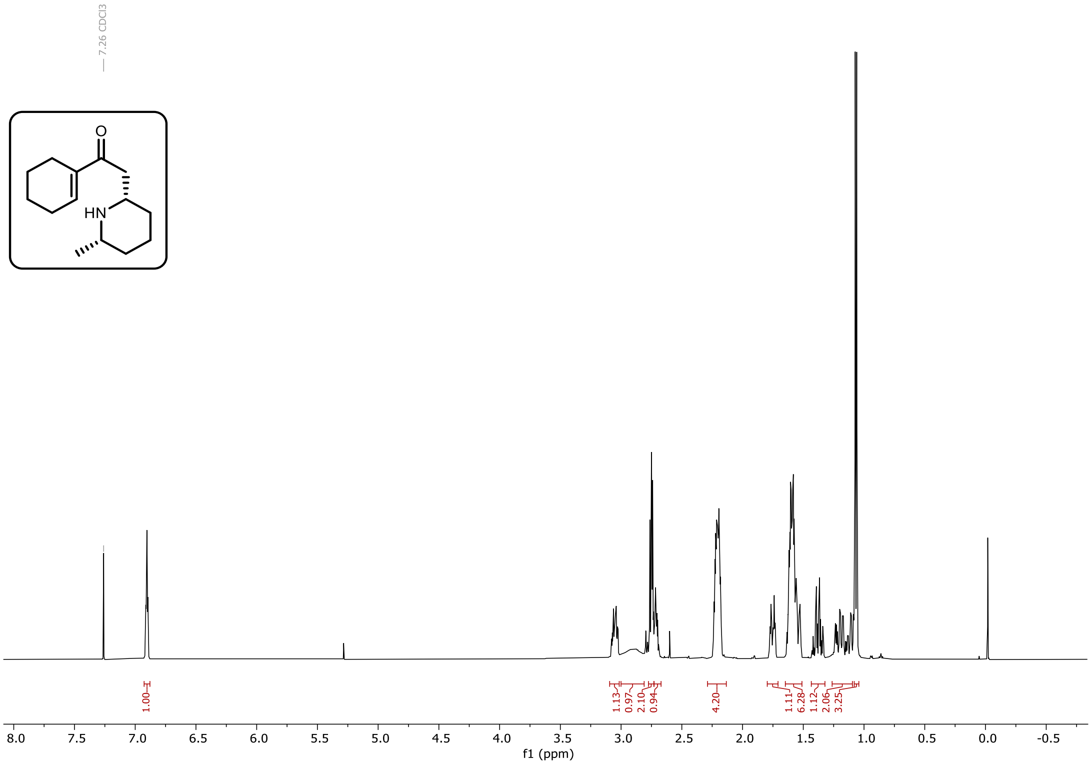

A64: *^{1}H NMR ($CDCl_3$) of compound* **(*S*,*S*)-3a**.

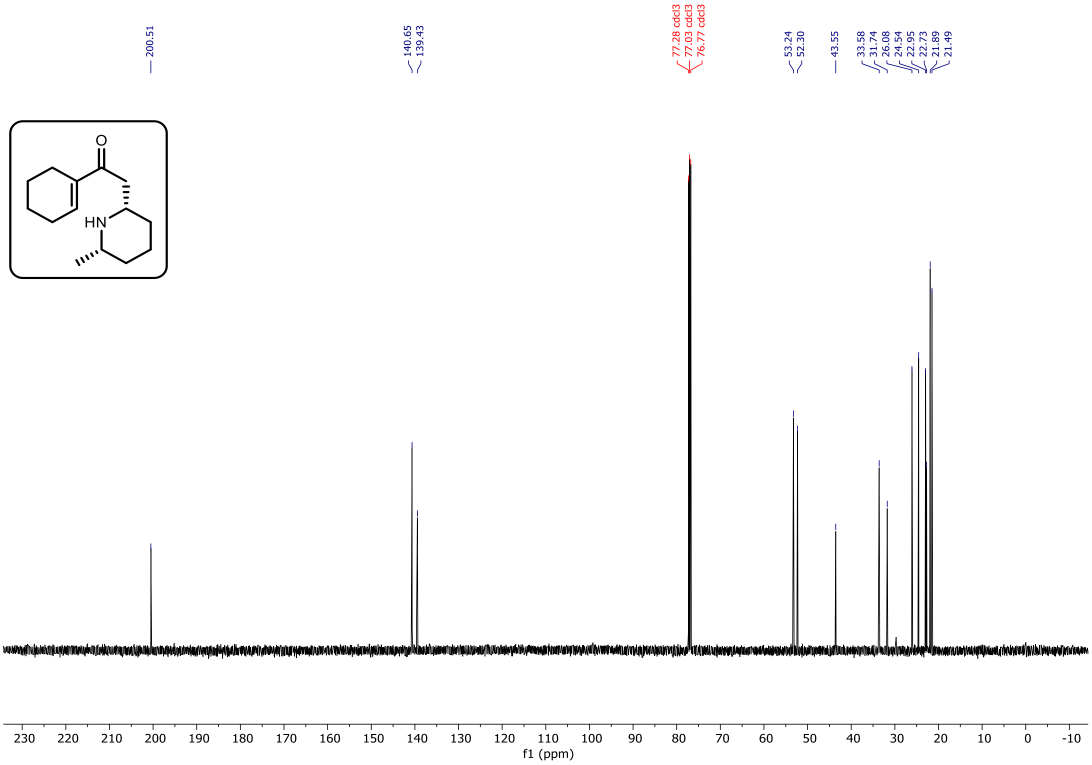

***A65:** ^{13}C NMR (CDCl$_3$) of compound* ***(S,S)*-3a**.

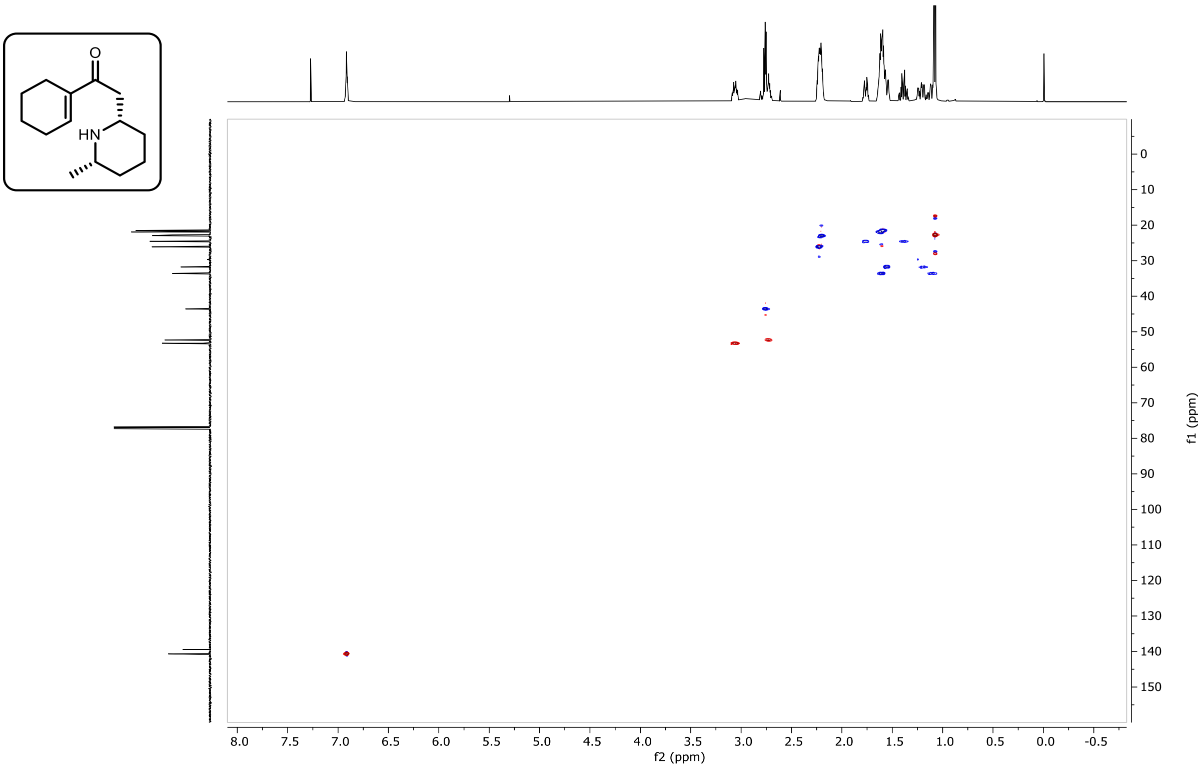

***A66:** HSQC (CDCl$_3$) of compound* ***(S,S)*-3a**.

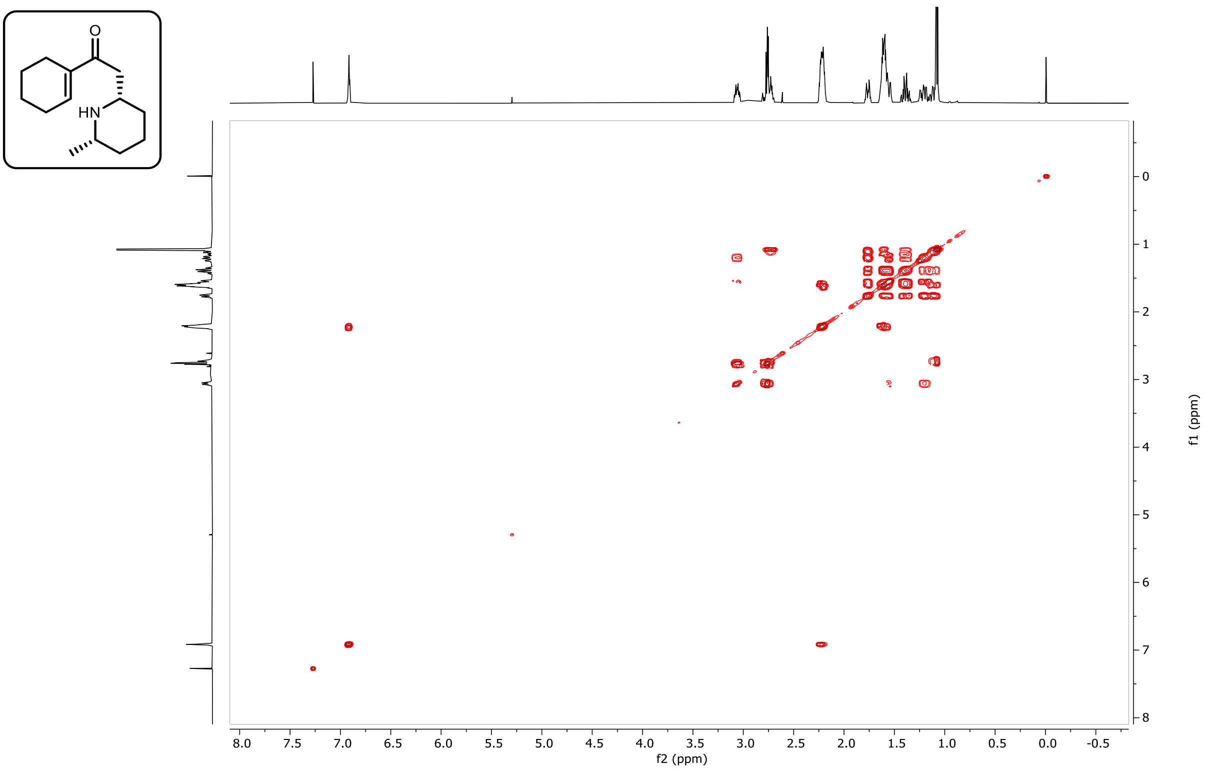

A67: *COSY (CDCl*$_3$*) of compound* ***(S,S)*-3a**.

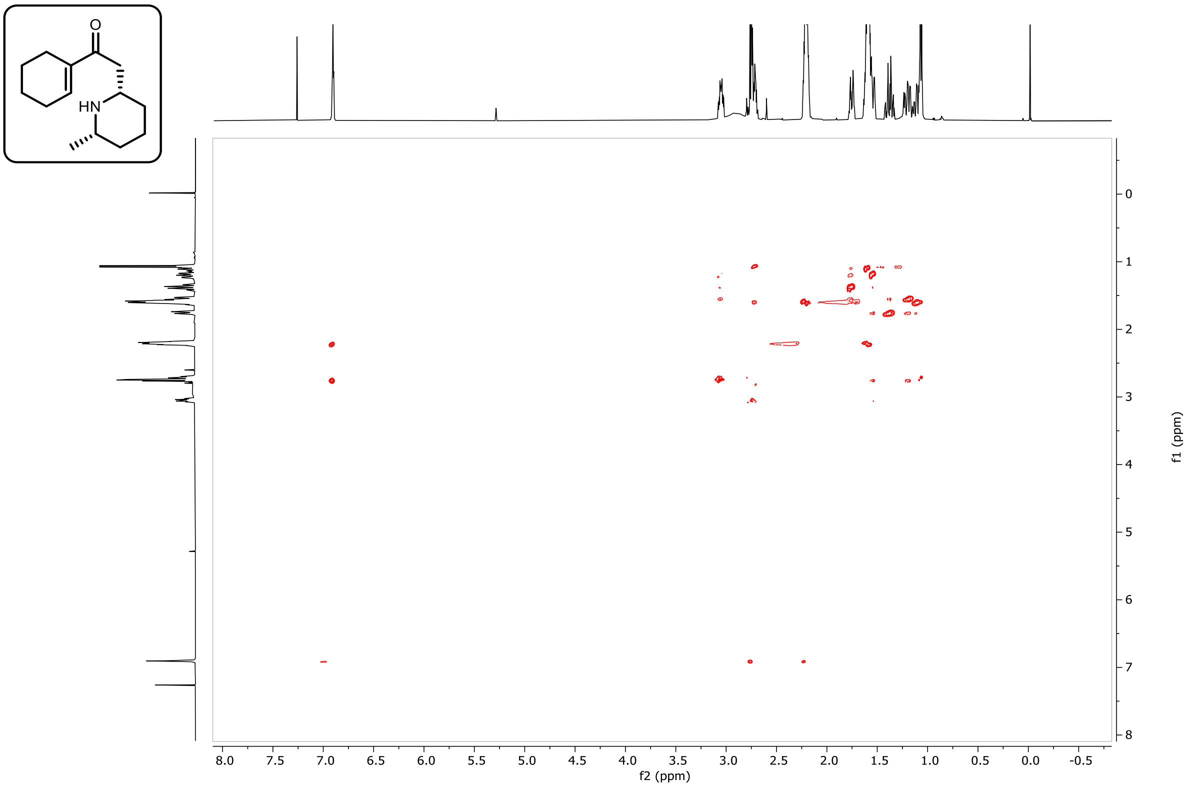

A68: *NOESY (CDCl*$_3$*) of compound* ***(S,S)*-3a**.

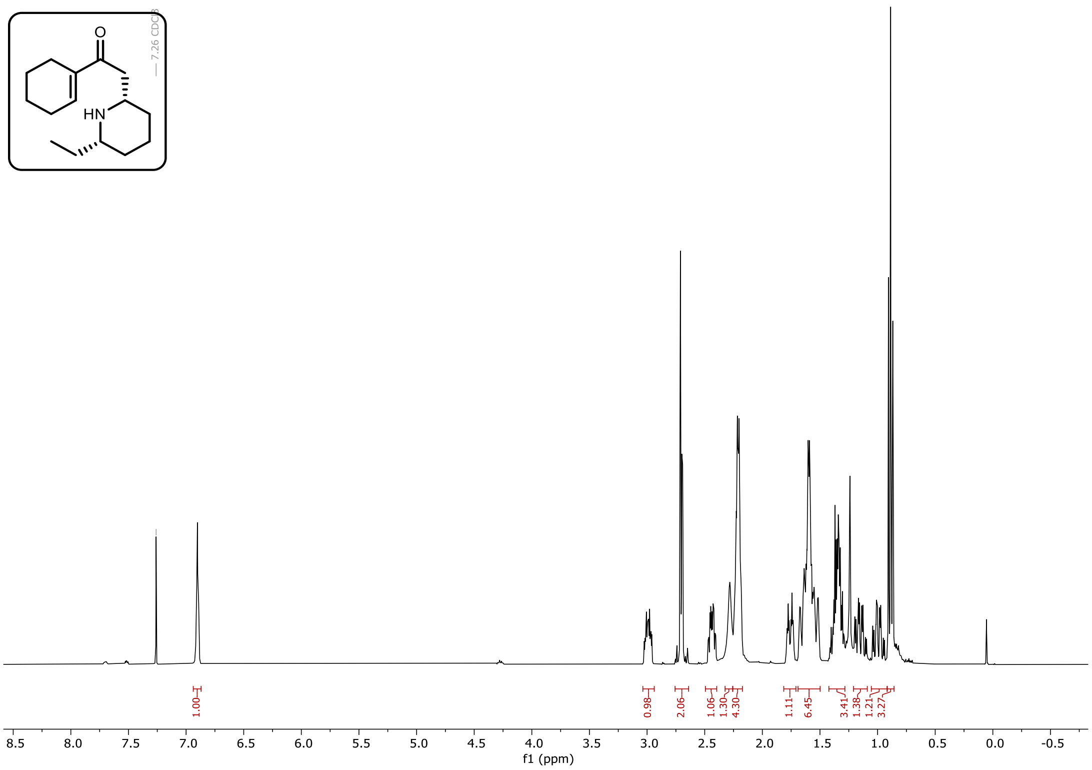

A69: *^{1}H NMR ($CDCl_3$) of compound* **(S,S)-3b**.

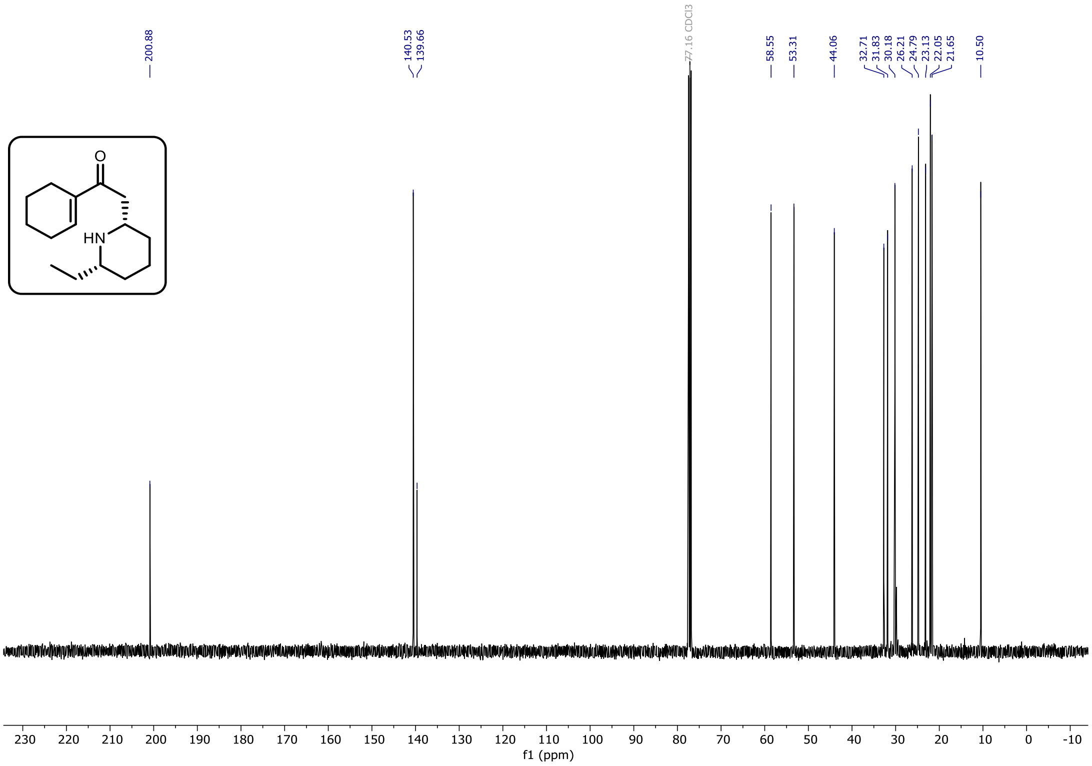

A70: *^{13}C NMR ($CDCl_3$) of compound* **(S,S)-3b**.

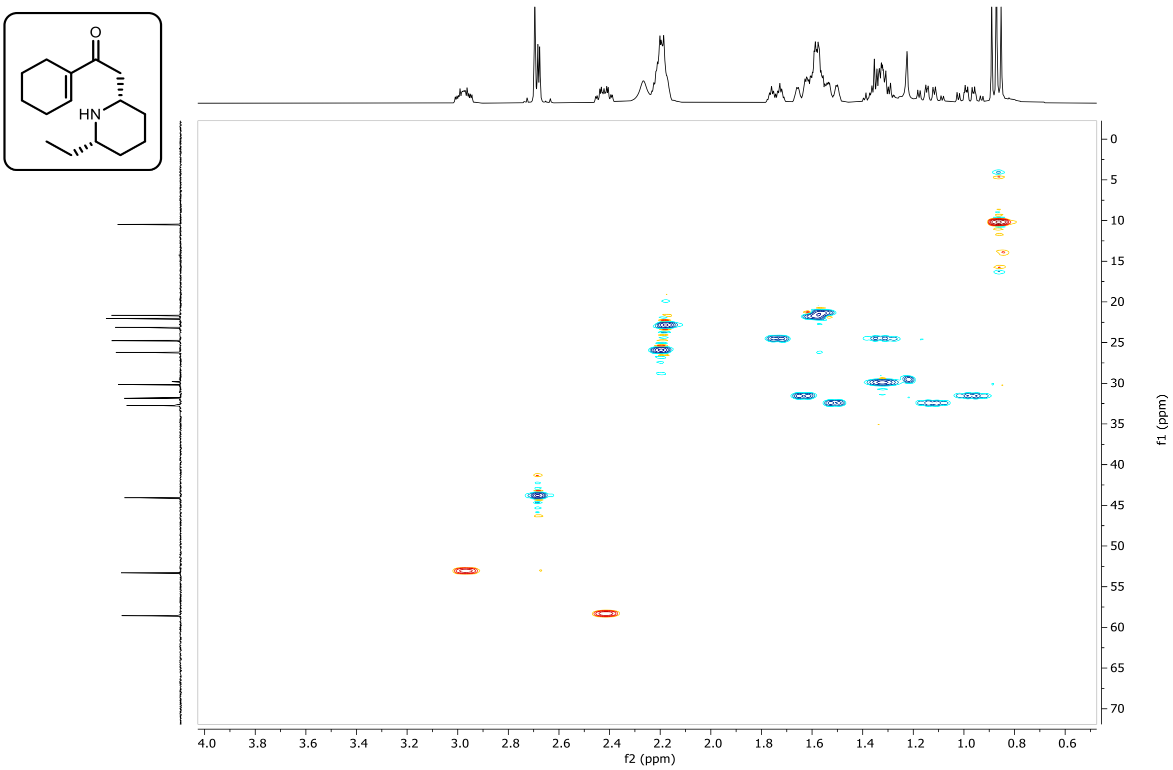

***A71:** H91SQC (CDCl$_3$) of compound* **(S,S)-3b**.

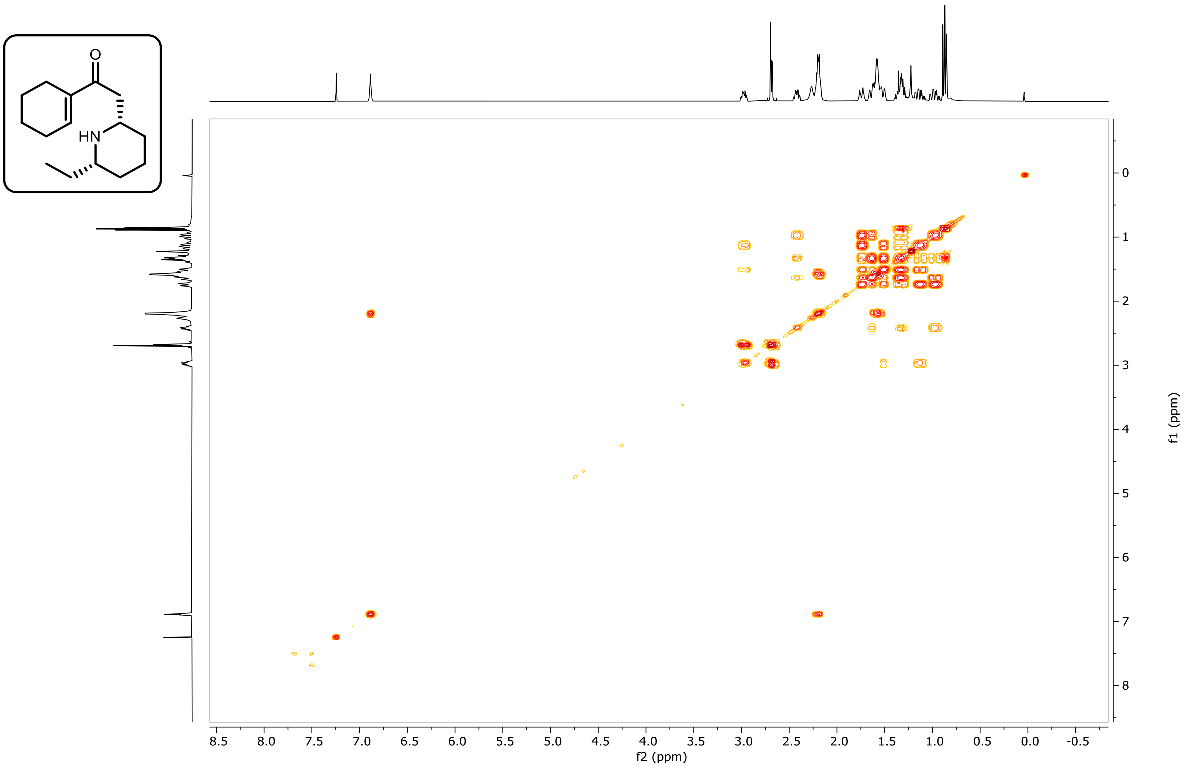

***A72:** COSY (CDCl$_3$) of compound* **(S,S)-3b**.

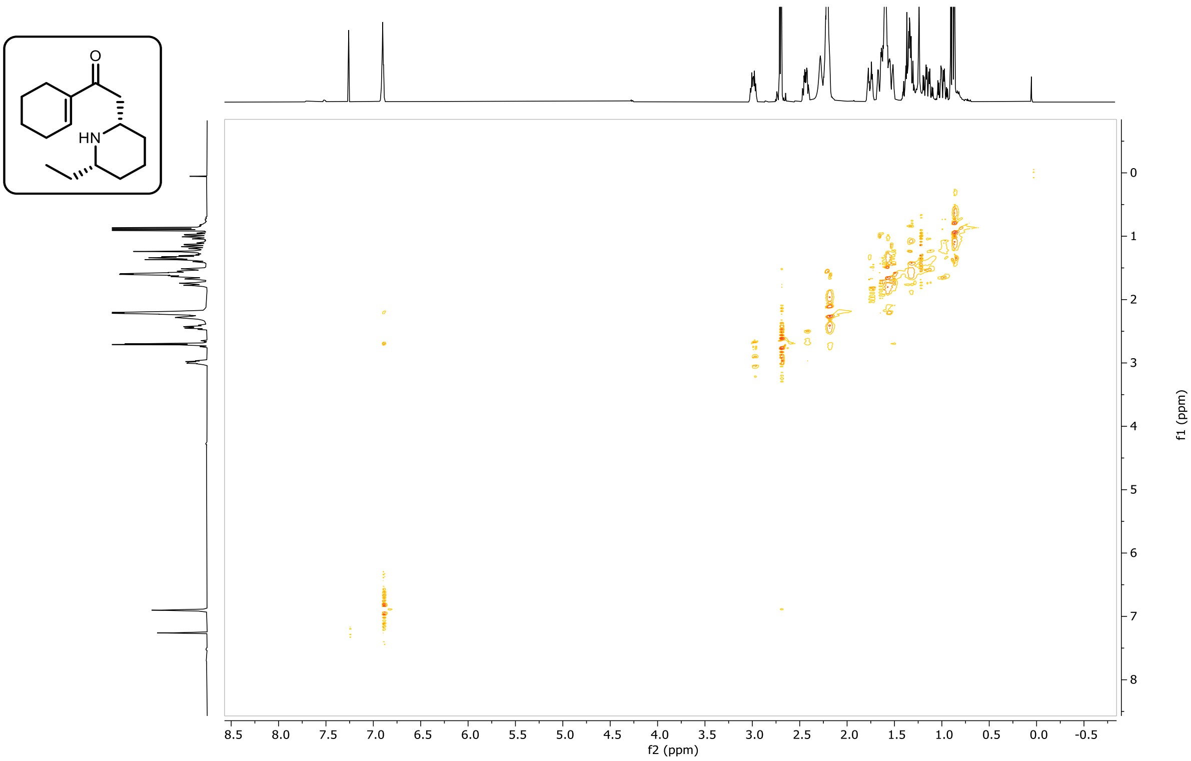

***A73:** NOESY (CDCl$_3$) of compound* **(S,S)-3b**.

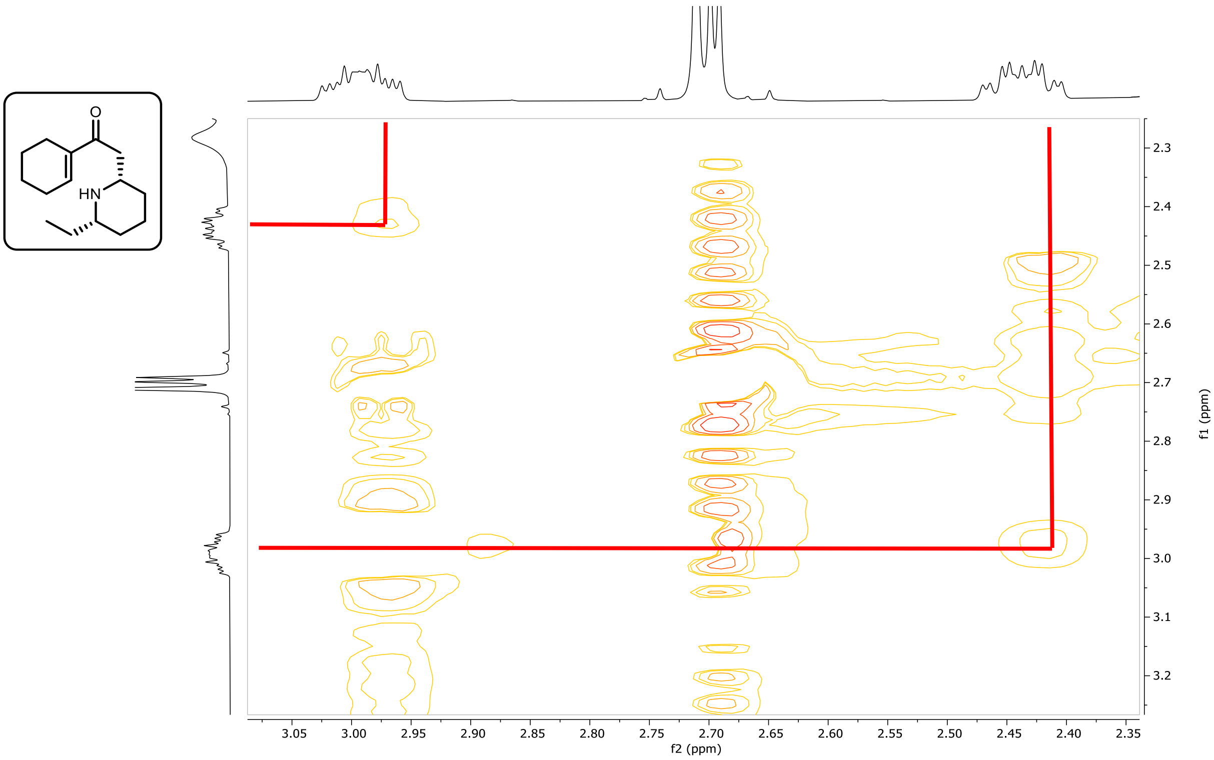

***A74:** NOESY zoomed (CDCl$_3$) of compound* **(S,S)-3b**.

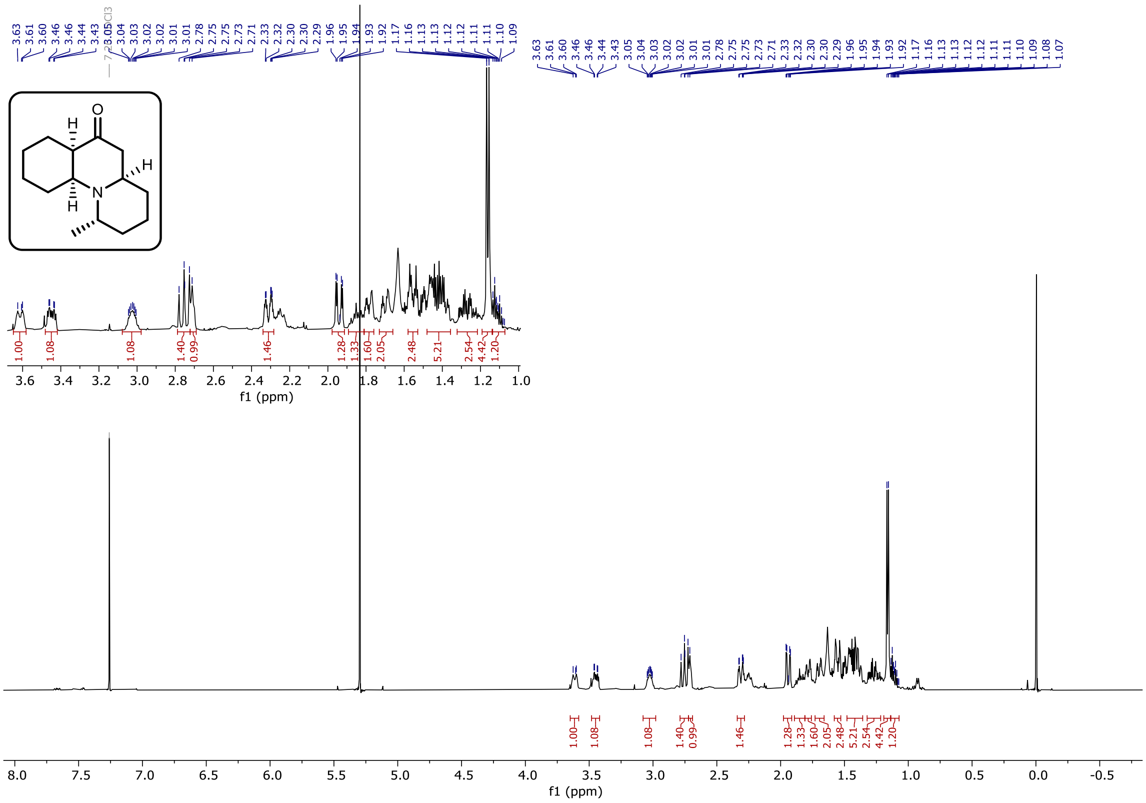

A75: *^{1}H NMR ($CDCl_3$) of compound 1S,4aR,6aR,10aS-**4a**.*

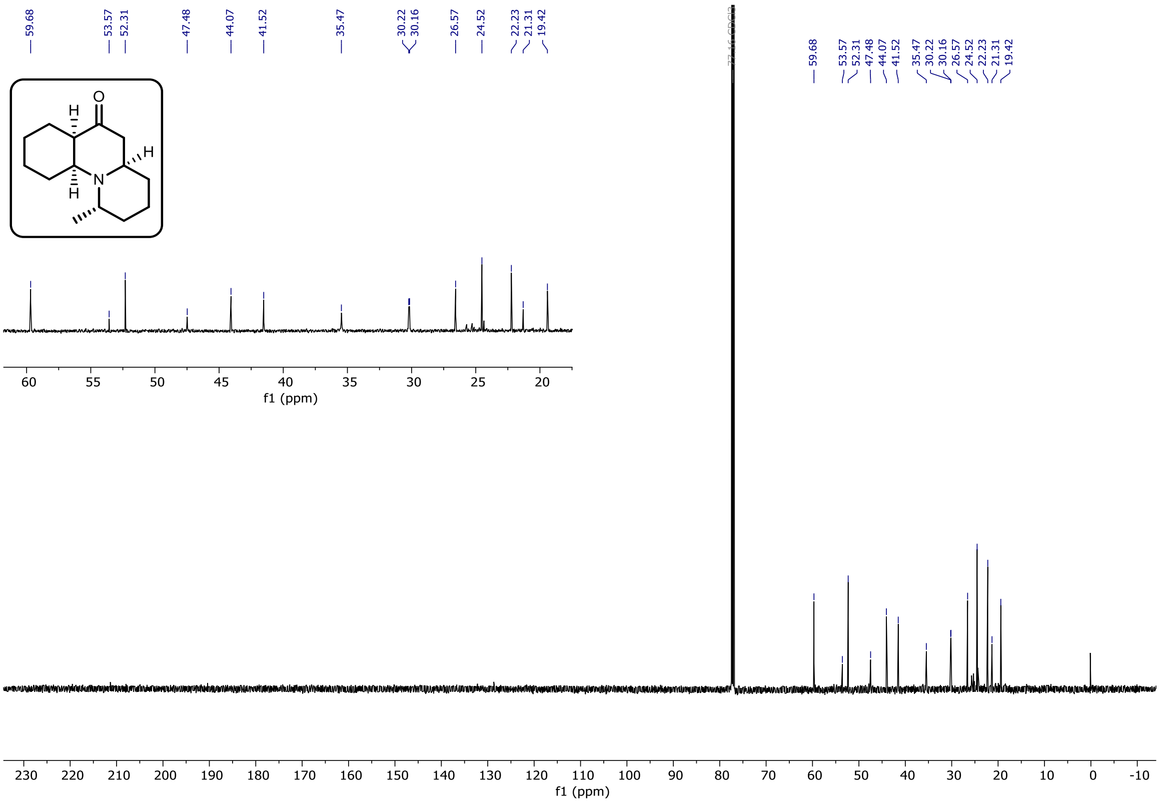

A76: *^{13}C NMR ($CDCl_3$) of compound 1S,4aR,6aR,10aS-**4a**.*

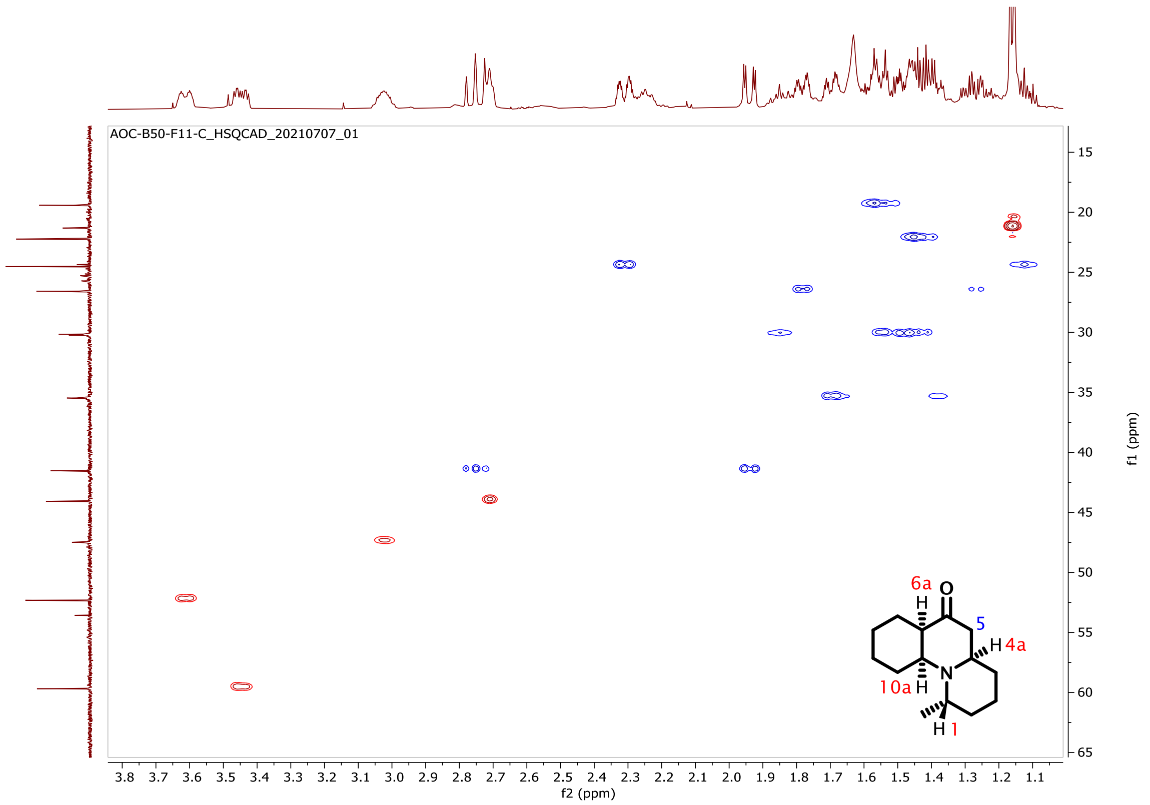

A77: *HSQC ($CDCl_3$) of compound 1S,4aR,6aR,10aS-**4a**.*

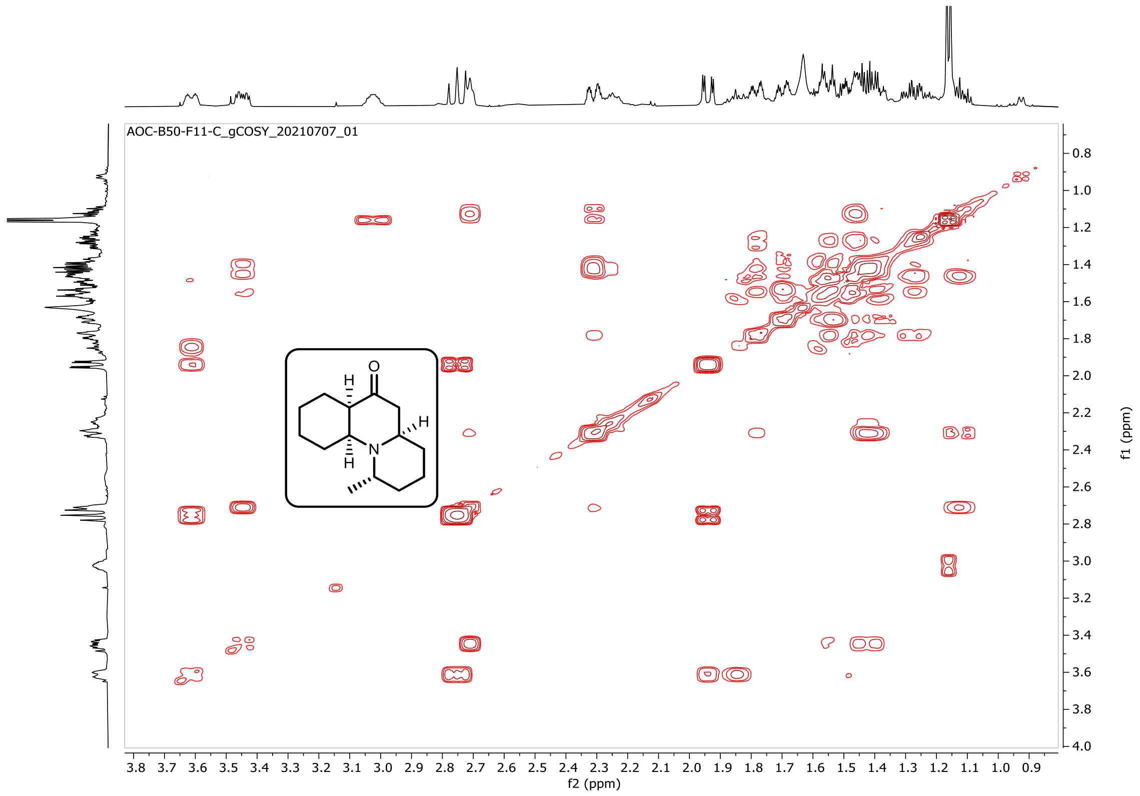

A78: *COSY NMR ($CDCl_3$) of compound 1S,4aR,6aR,10aS-**4a**.*

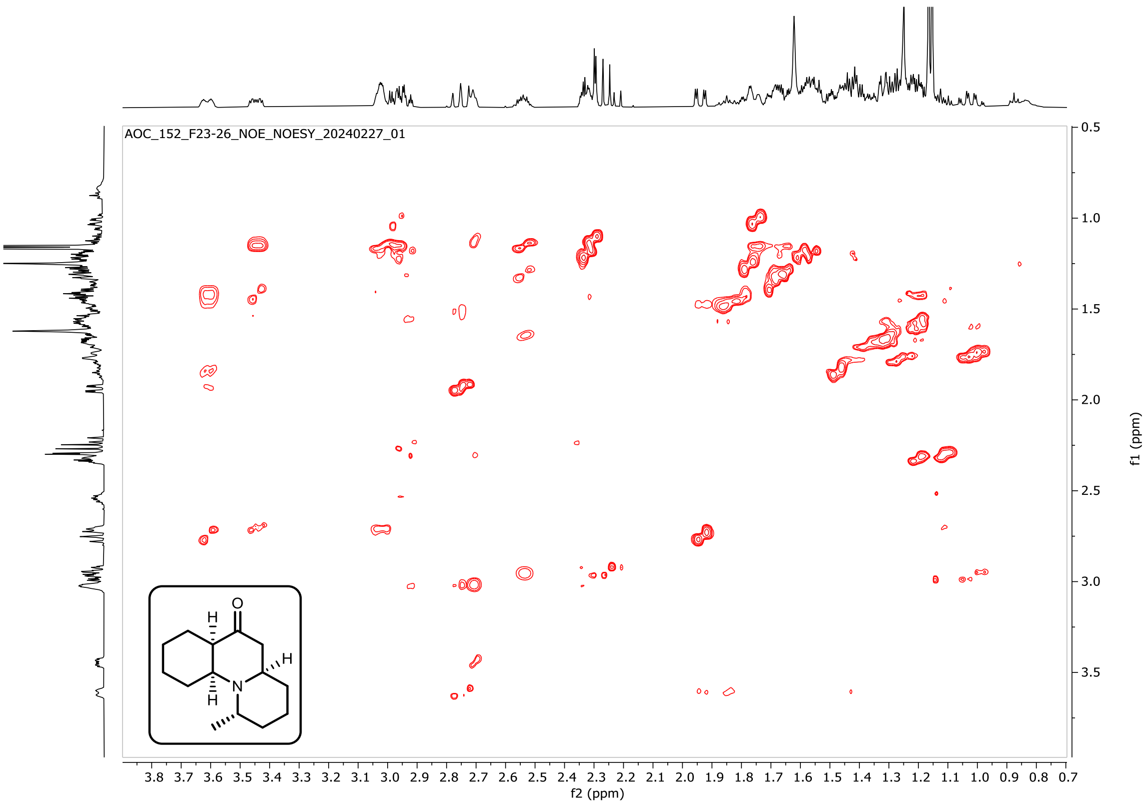

***A79:** NOESY ($CDCl_3$) of compound 1S,4aR,6aR,10aS-**4a**.*

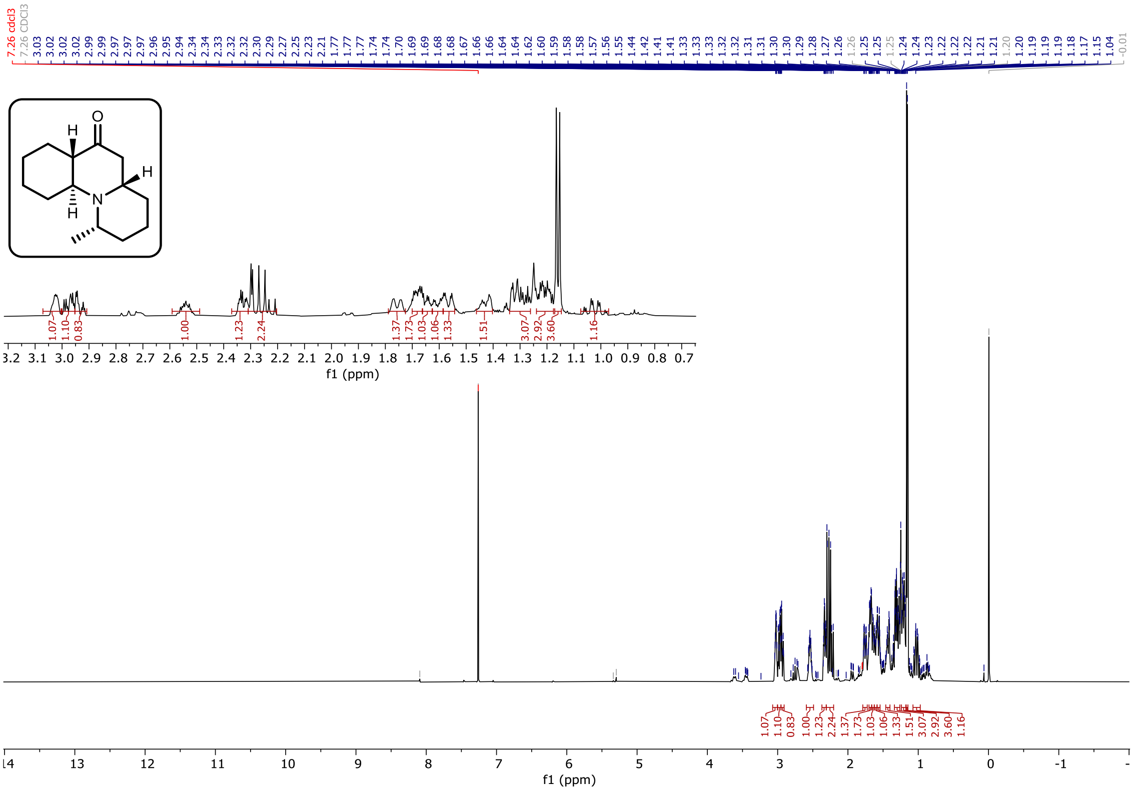

***A80:** ^{1}H NMR ($CDCl_3$) of compound 1S,4aS,6aS,10aS -**4a**.*

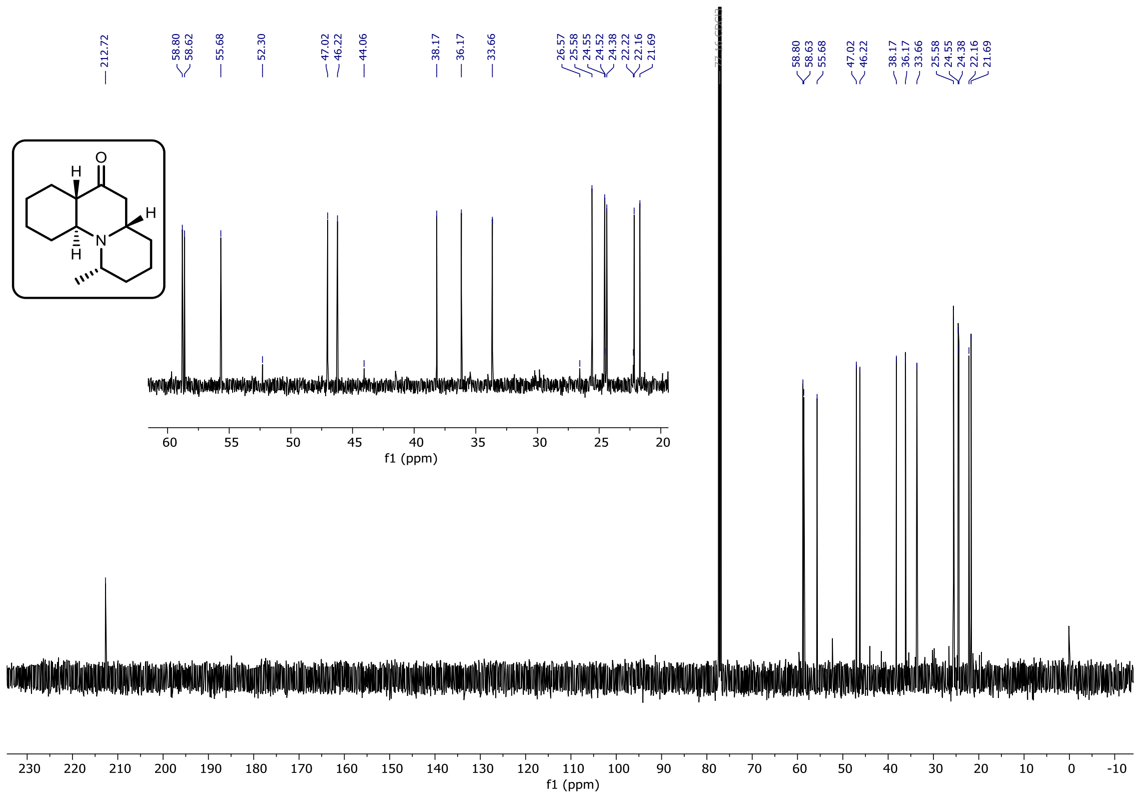

A81: ^{13}C NMR (CDCl$_3$) of compound 1S,4aS,6aS,10aS -**4a**.

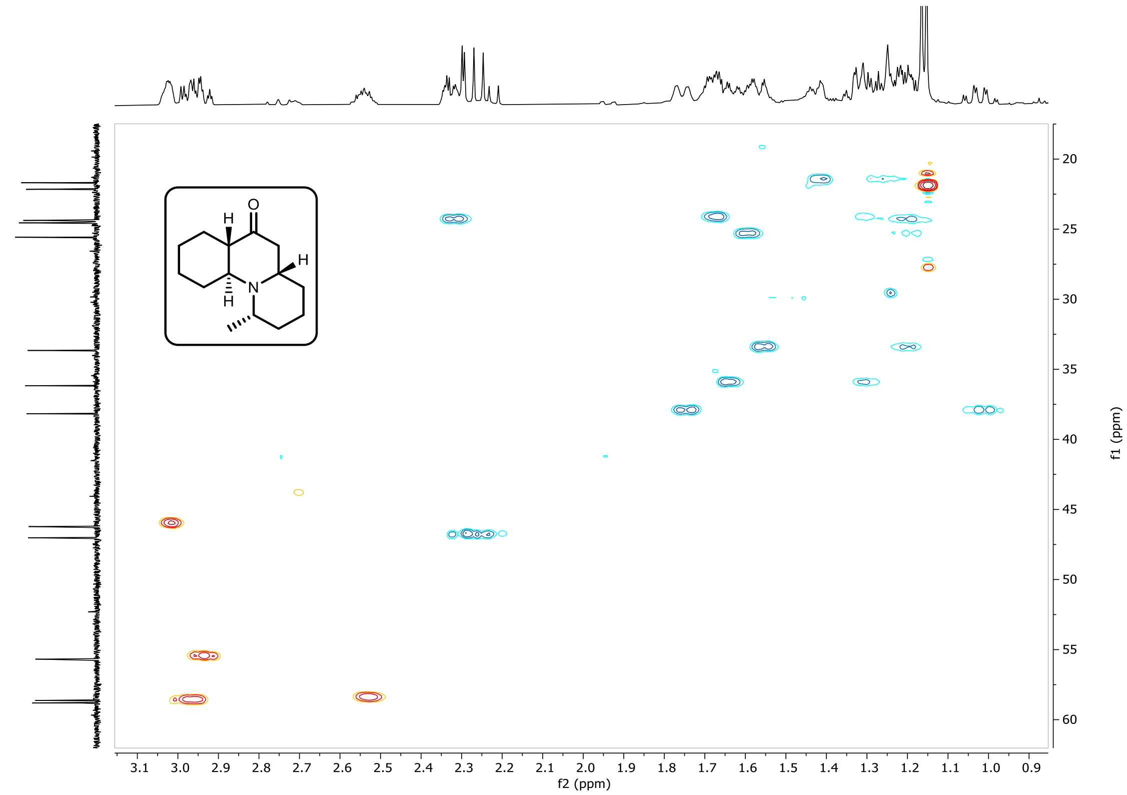

A82: HSQC (CDCl$_3$) of compound 1S,4aS,6aS,10aS -**4a**.

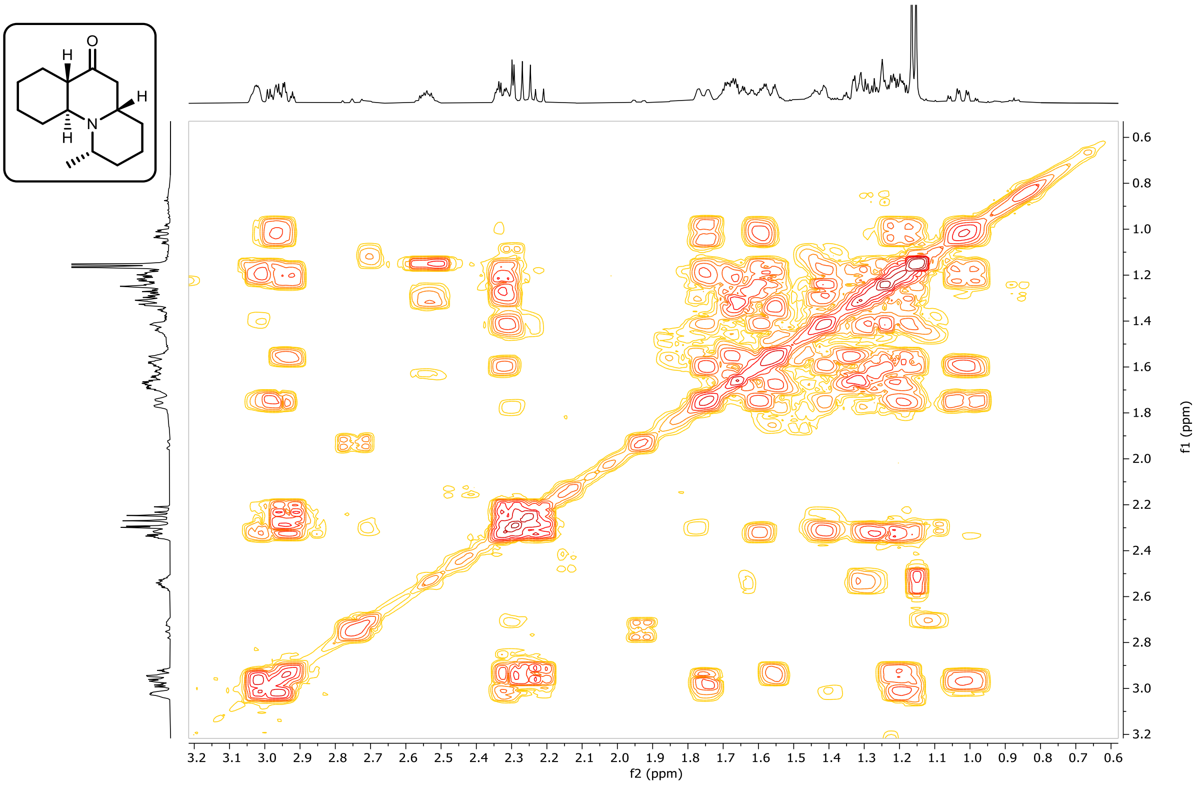

A83: *COSY NMR (CDCl₃) of compound 1S,4aS,6aS,10aS -**4a**.*

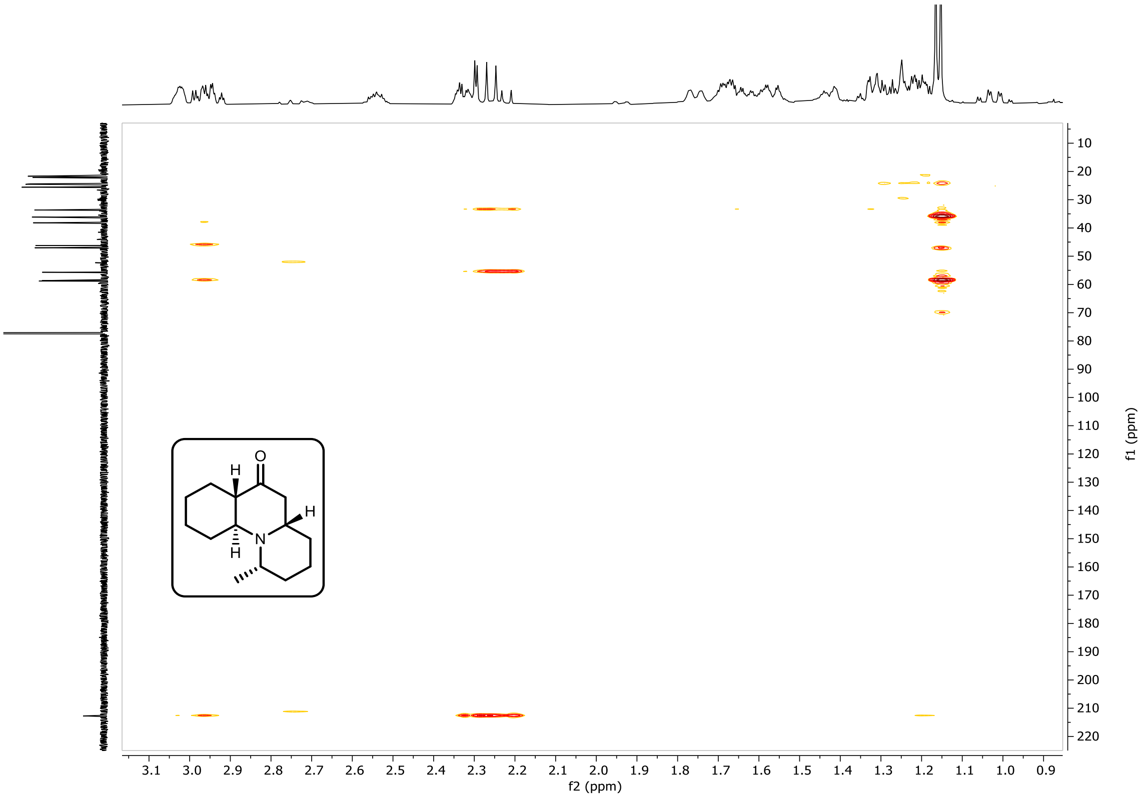

A84: *HMBC (CDCl₃) of compound 1S,4aS,6aS,10aS -**4a**.*

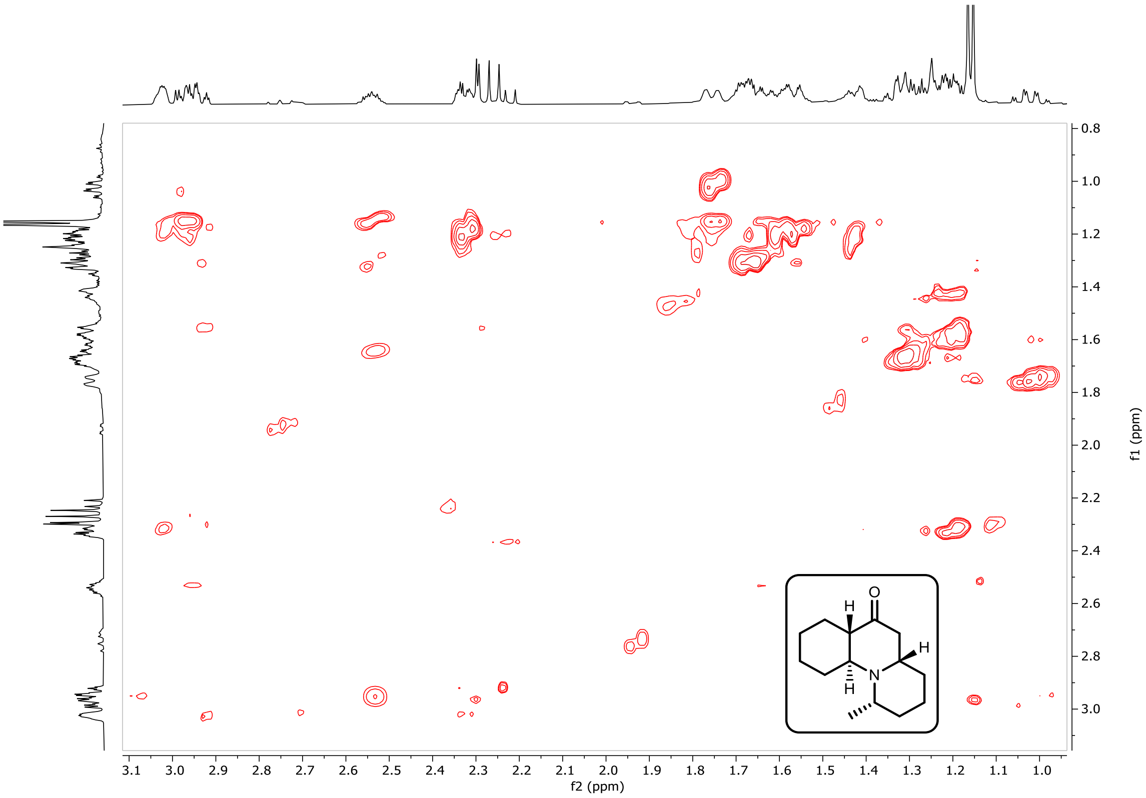

A85: *NOESY ($CDCl_3$) of compound 1S,4aS,6aS,10aS -**4a**.*

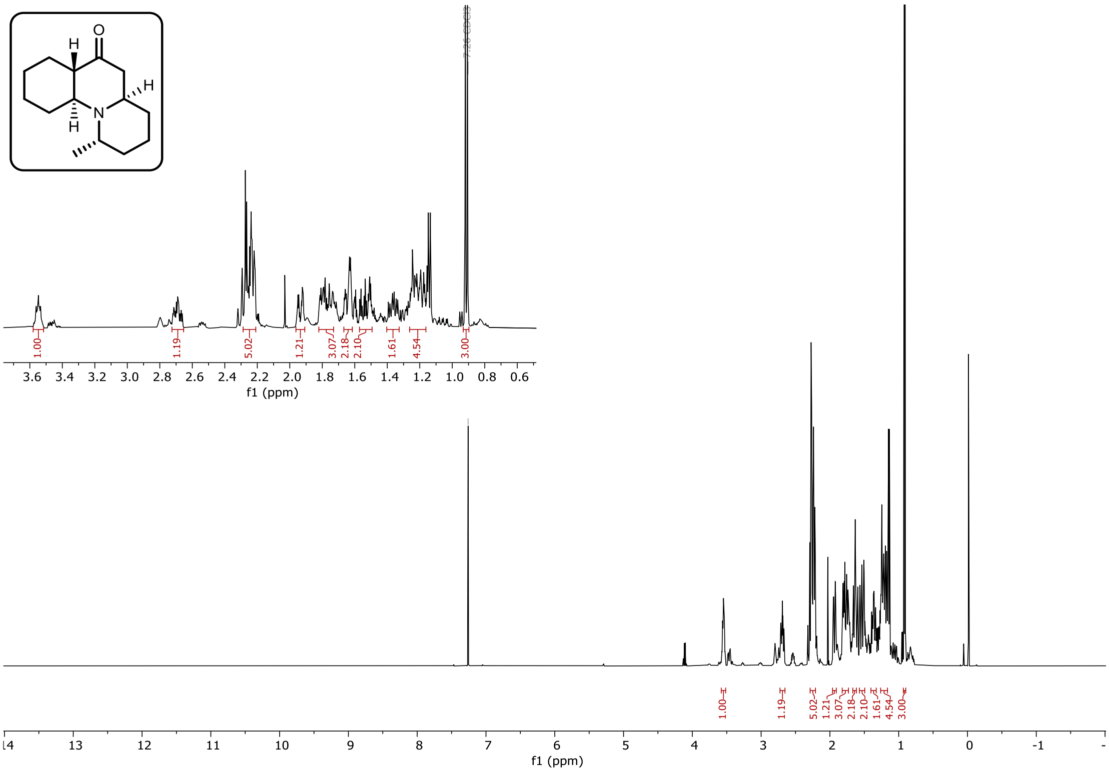

A86: *^{1}H NMR ($CDCl_3$) of compound 1S,4aR,6aS,10aS-**4a**.*

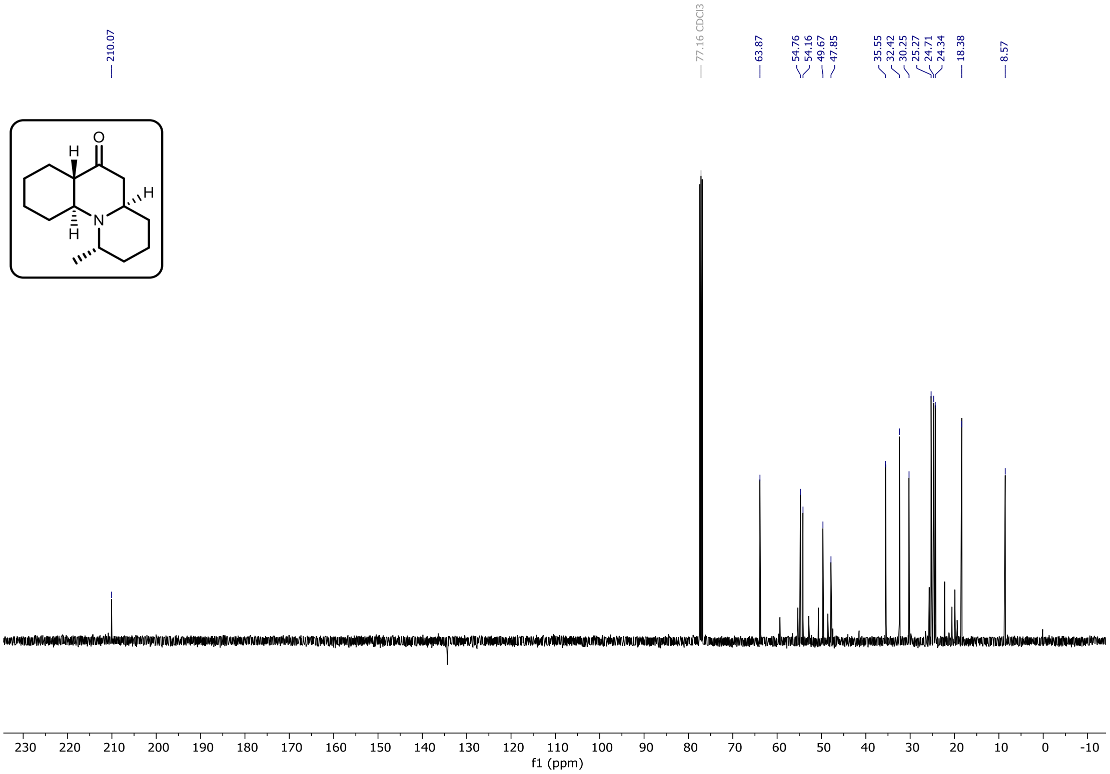

A87: *^{13}C NMR ($CDCl_3$) of compound 1S,4aR,6aS,10aS-**4a**.*

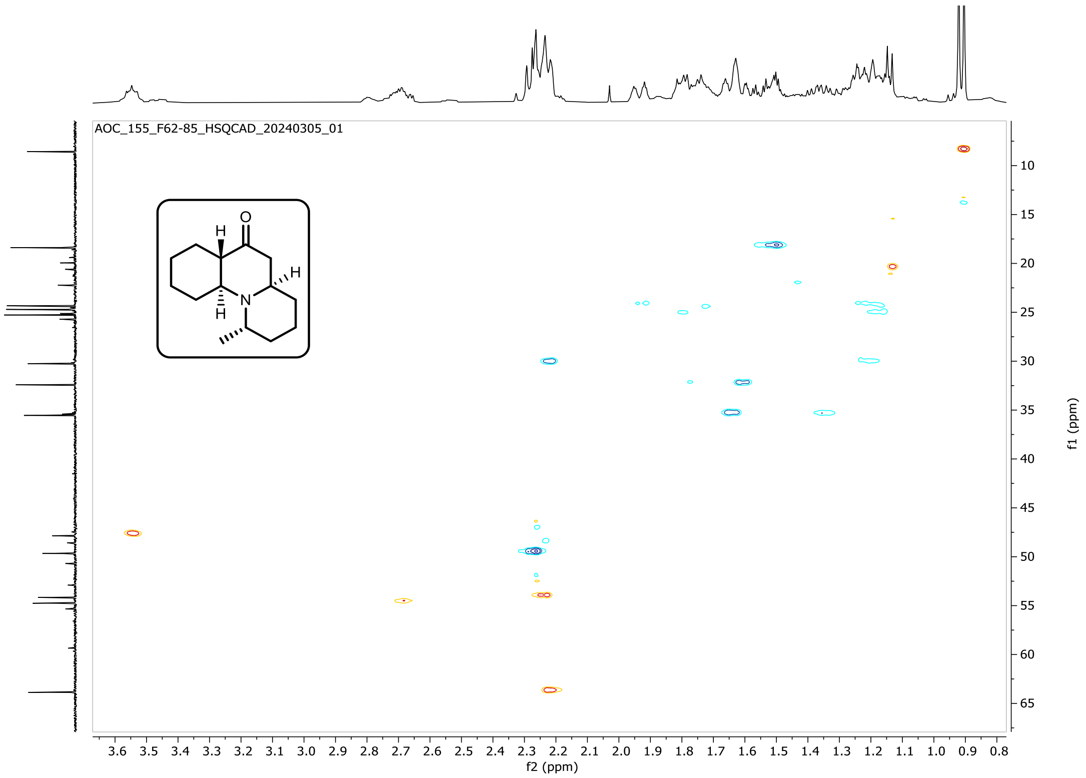

A88: *HSQC ($CDCl_3$) of compound 1S,4aR,6aS,10aS-**4a**.*

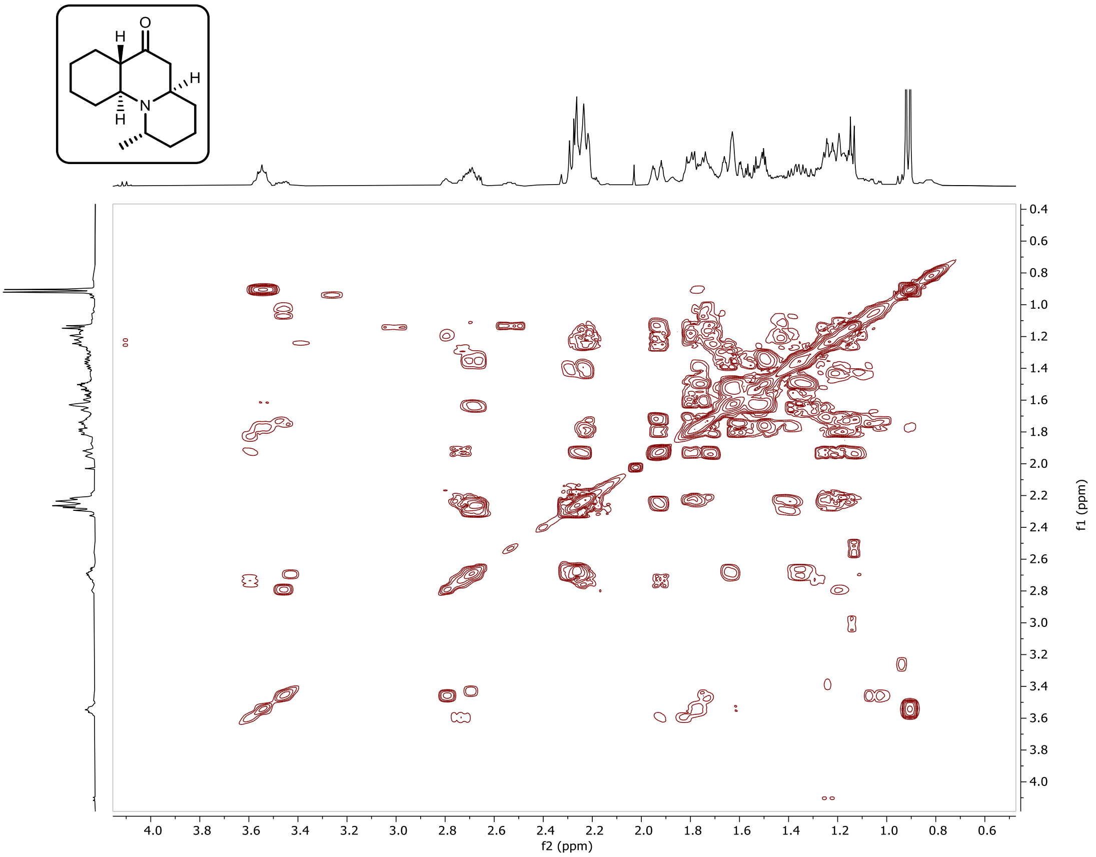

A89: *COSY NMR (CDCl$_3$) of compound 1S,4aR,6aS,10aS-**4a**.*

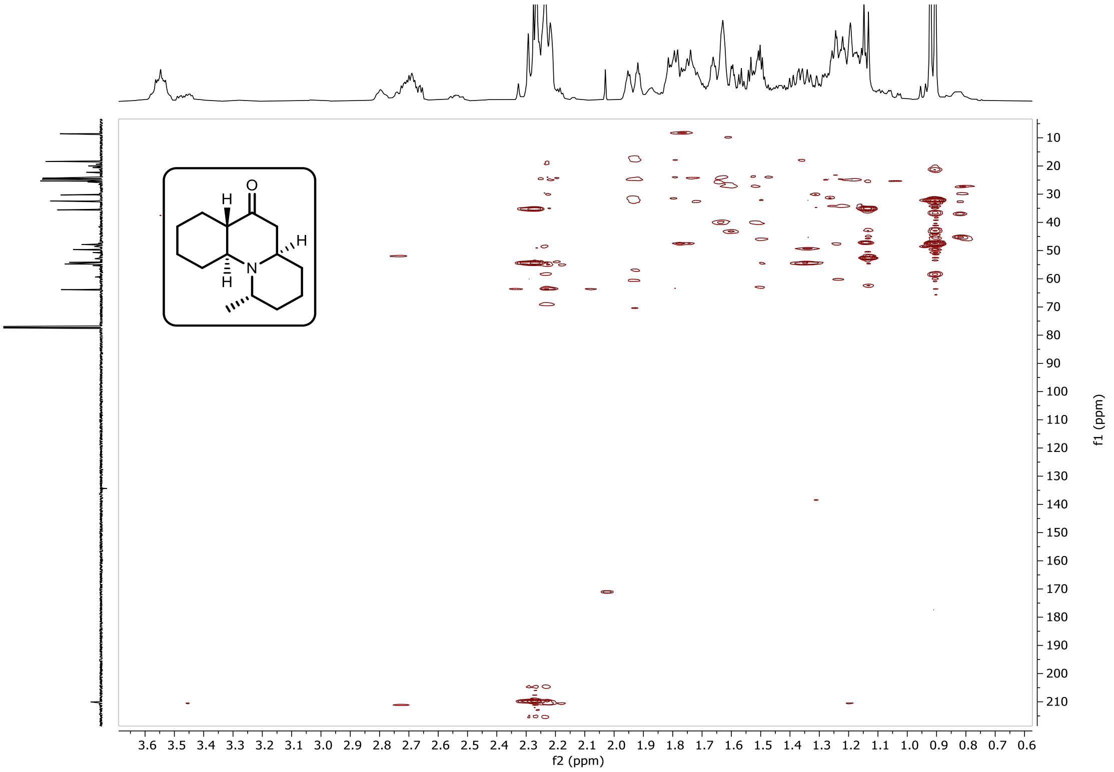

A90: *HMBC (CDCl$_3$) of compound 1S,4aR,6aS,10aS-**4a**.*

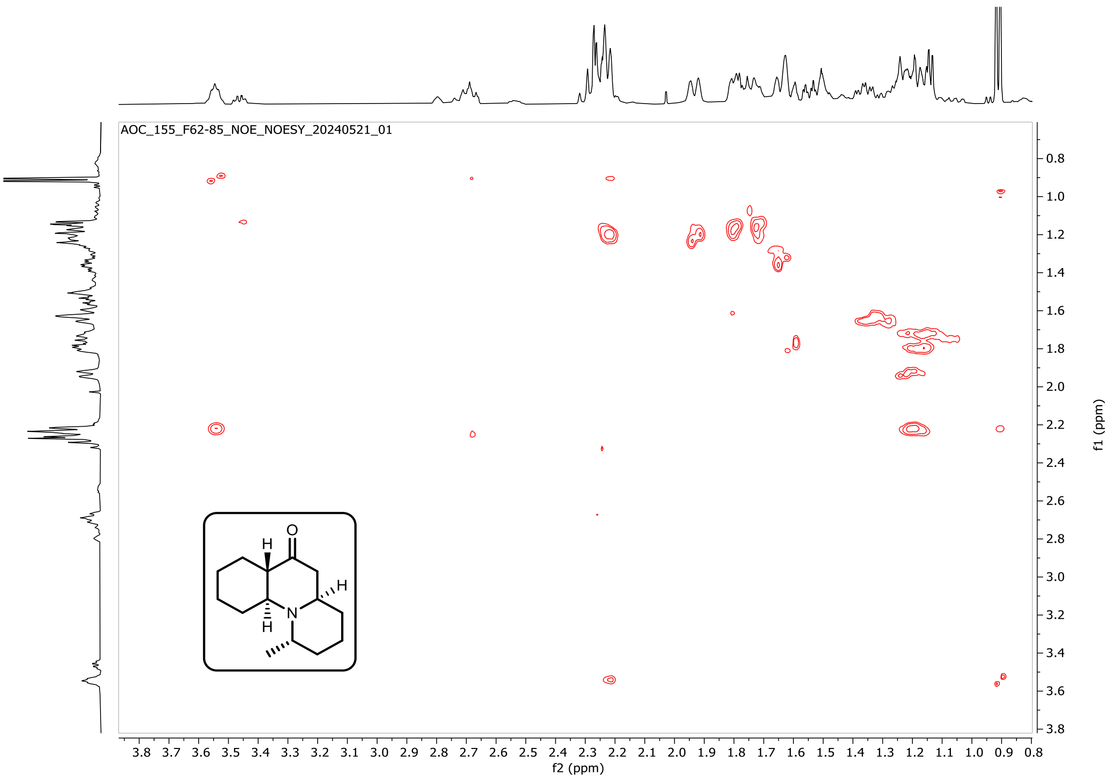

A91: *NOESY ($CDCl_3$) of compound 1S,4aR,6aS,10aS-**4a**.*

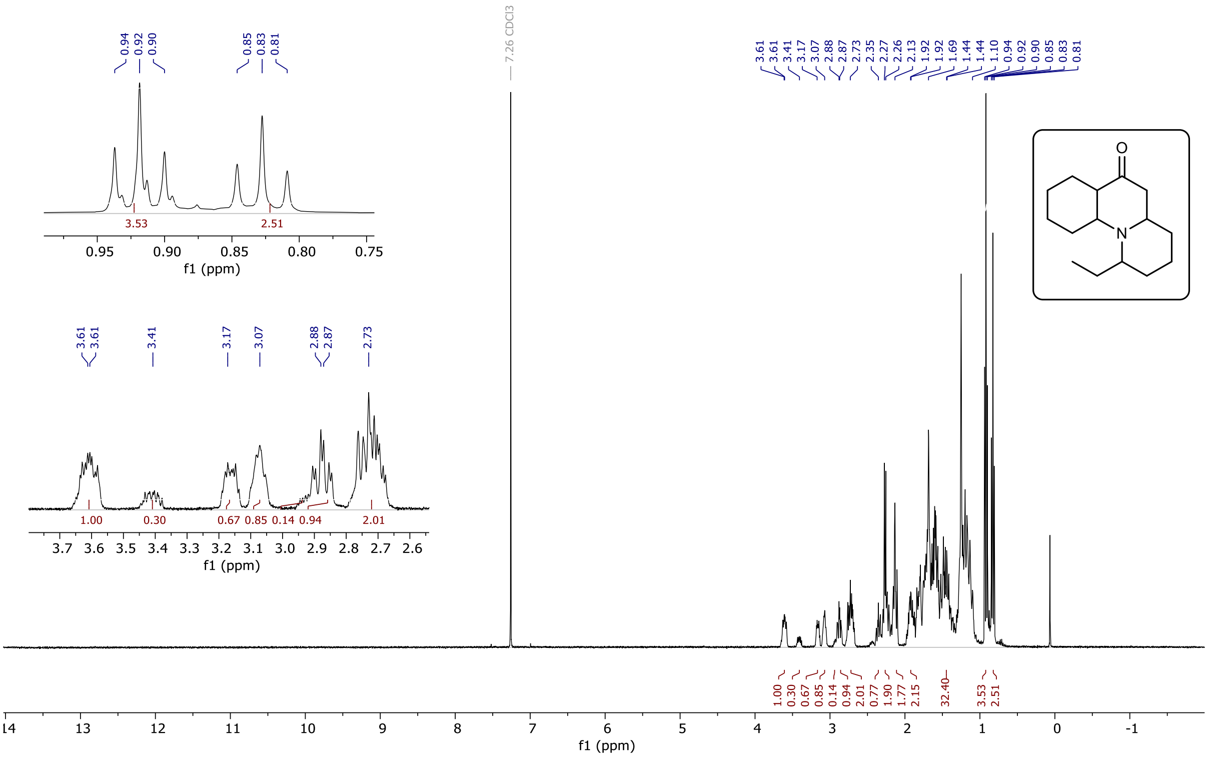

A92: *^{1}H NMR ($CDCl_3$) of compounds **4b**.*

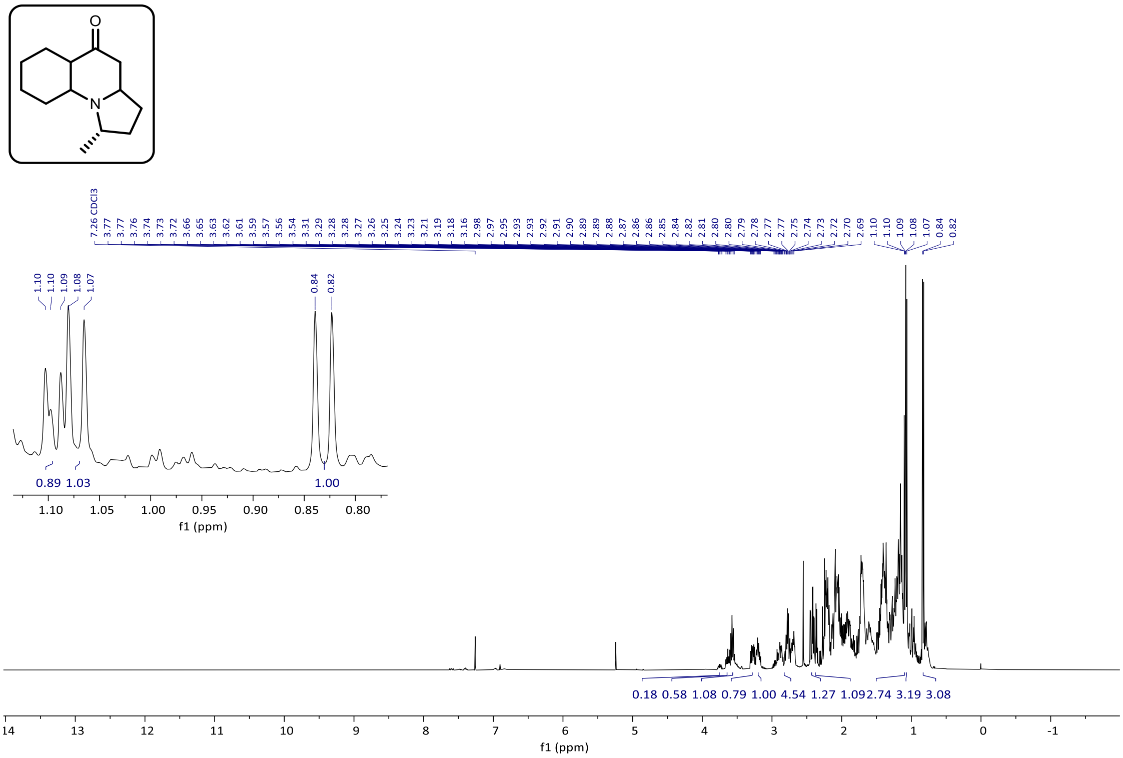

A93: *^{1}H NMR (CDCl$_3$) of compounds* **(1*S*)-4c**.

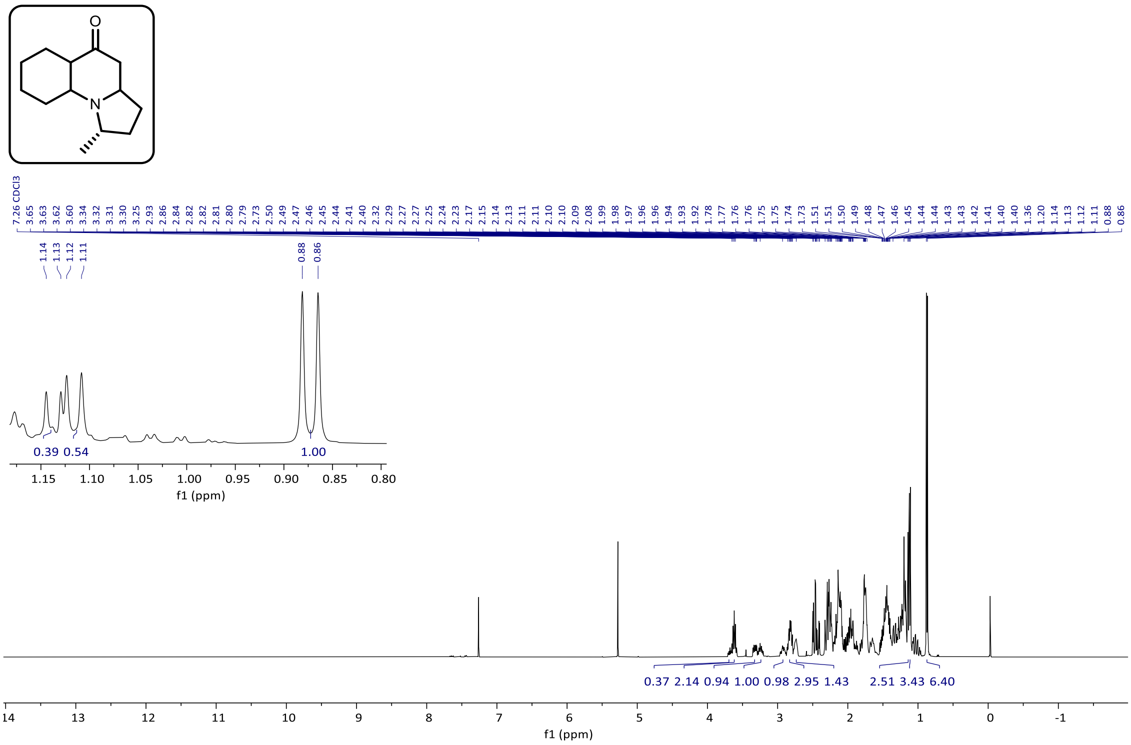

A94: *^{1}H NMR (CDCl$_3$) of compounds* **(1*S*)-4c** *after silica plug.*

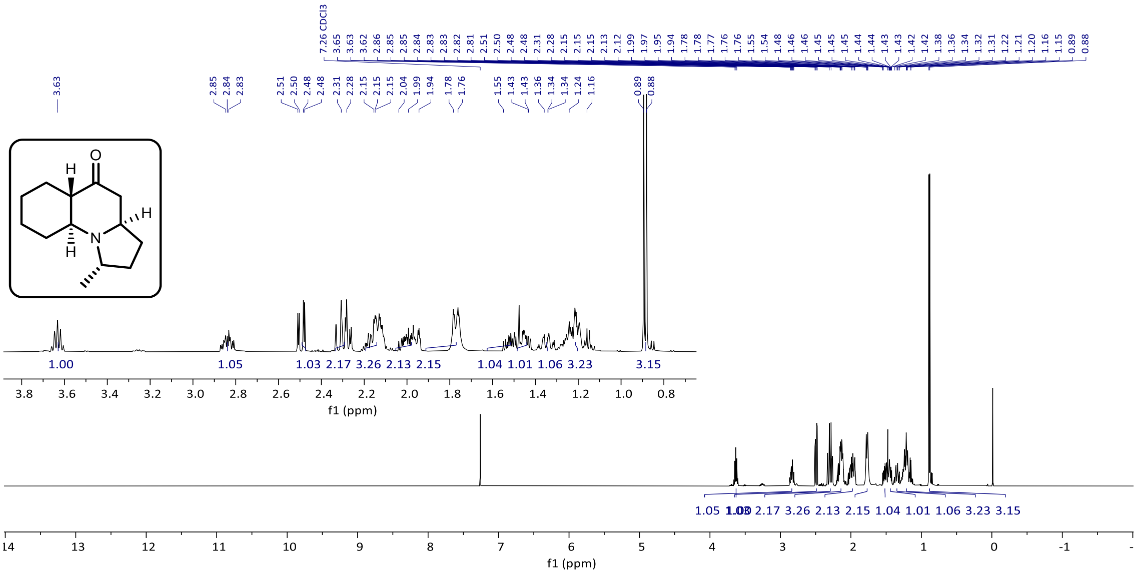

A95: *^{1}H NMR ($CDCl_3$) of compound 1S,3aR,5aS,9aS-**4c**.*

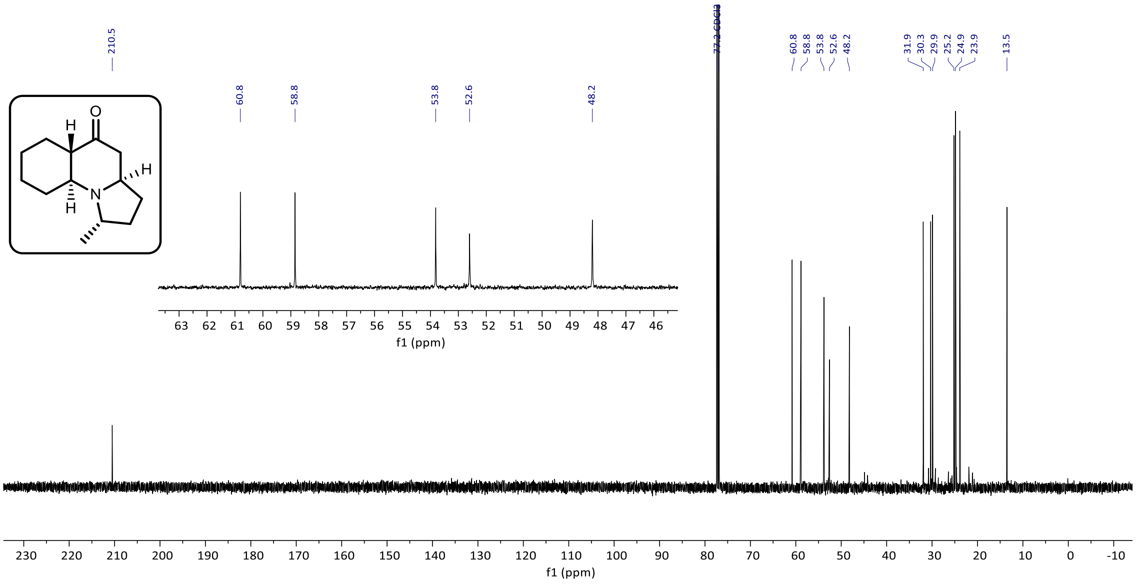

A96: *^{13}C NMR ($CDCl_3$) of compound 1S,3aR,5aS,9aS-**4c**.*

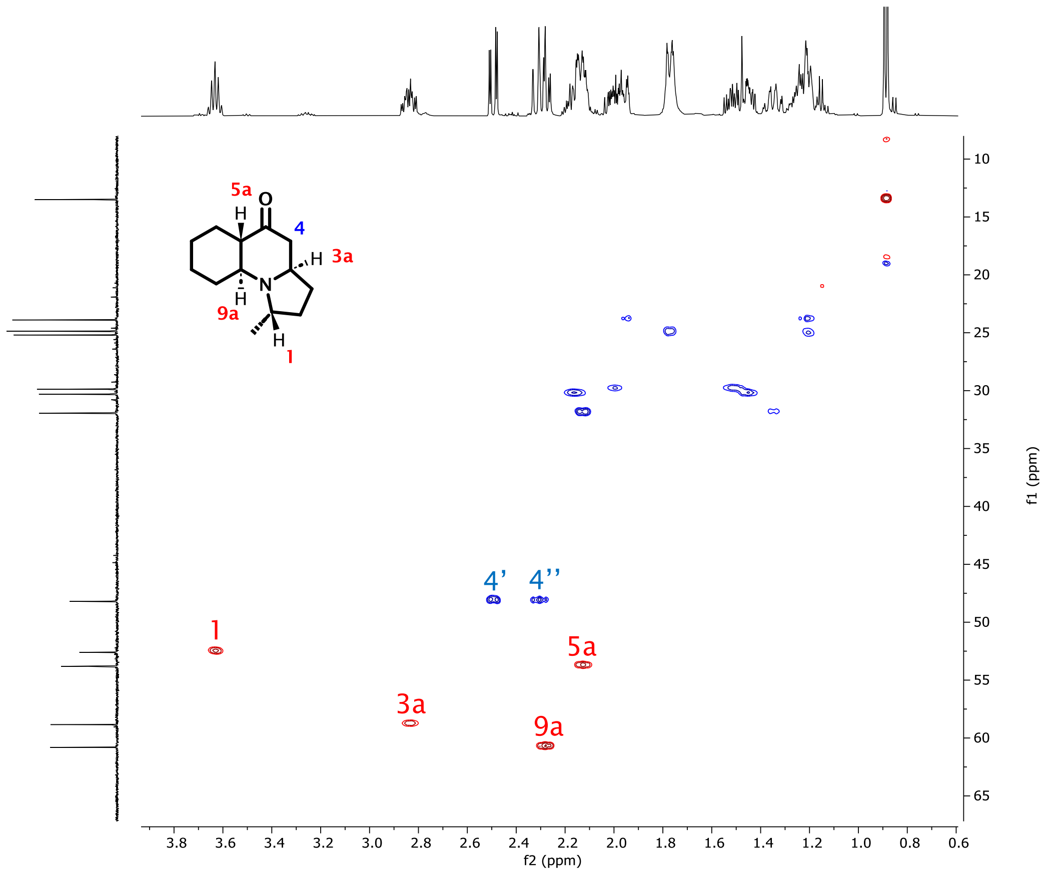

***A97:** HSQC ($CDCl_3$) of compound 1S,3aR,5aS,9aS-**4c**.*

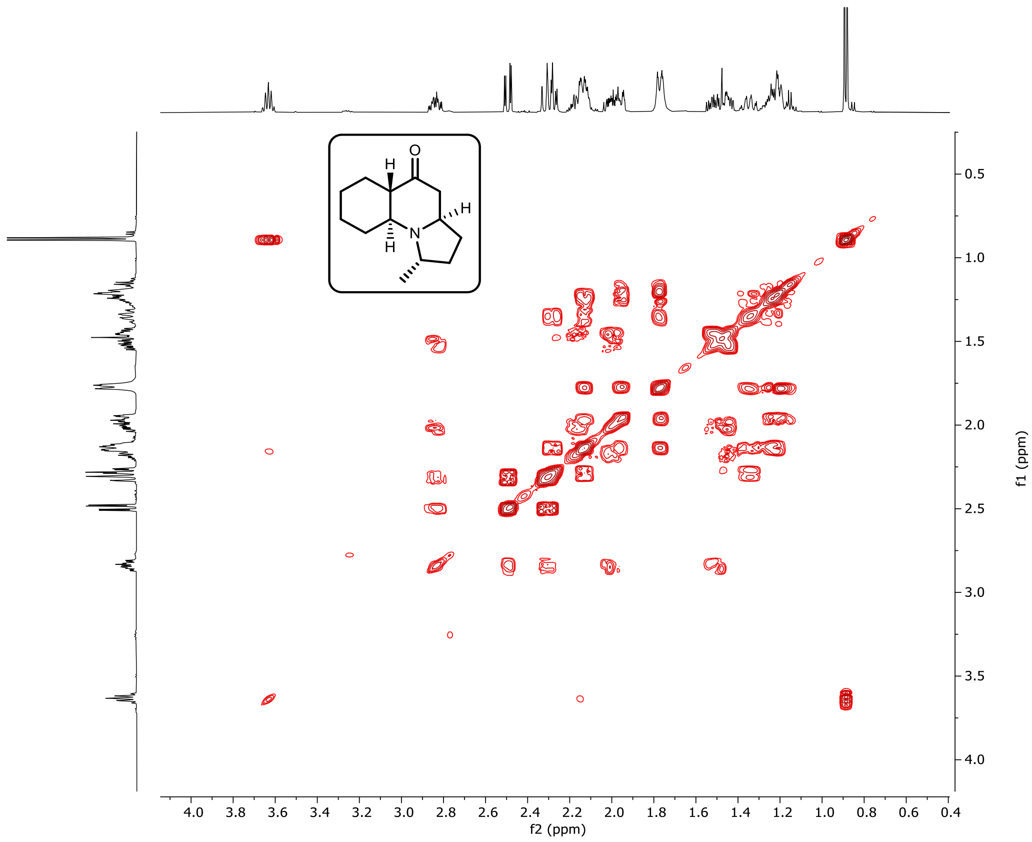

***A98:** COSY NMR ($CDCl_3$) of compound 1S,3aR,5aS,9aS-**4c**.*

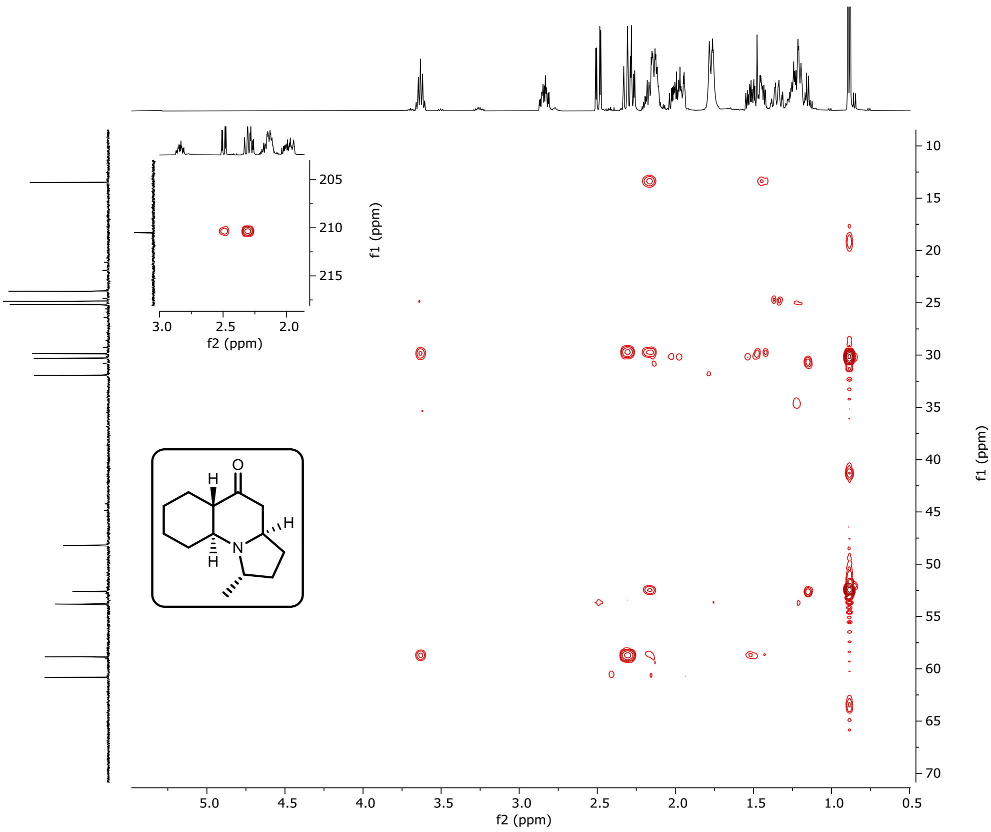

A99: *HMBC (CDCl₃) of compound 1S,3aR,5aS,9aS-**4c**.*

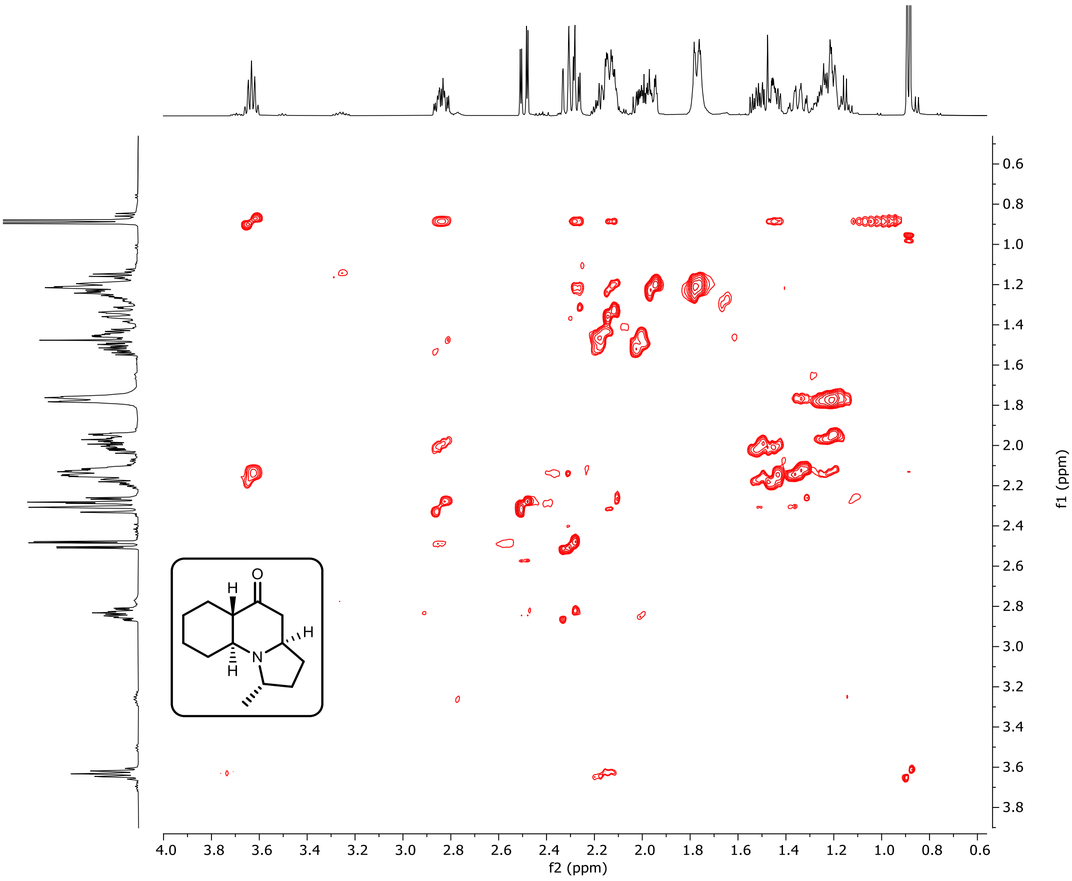

A100: *NOESY (CDCl₃) of compound 1S,3aR,5aS,9aS-**4c**.*

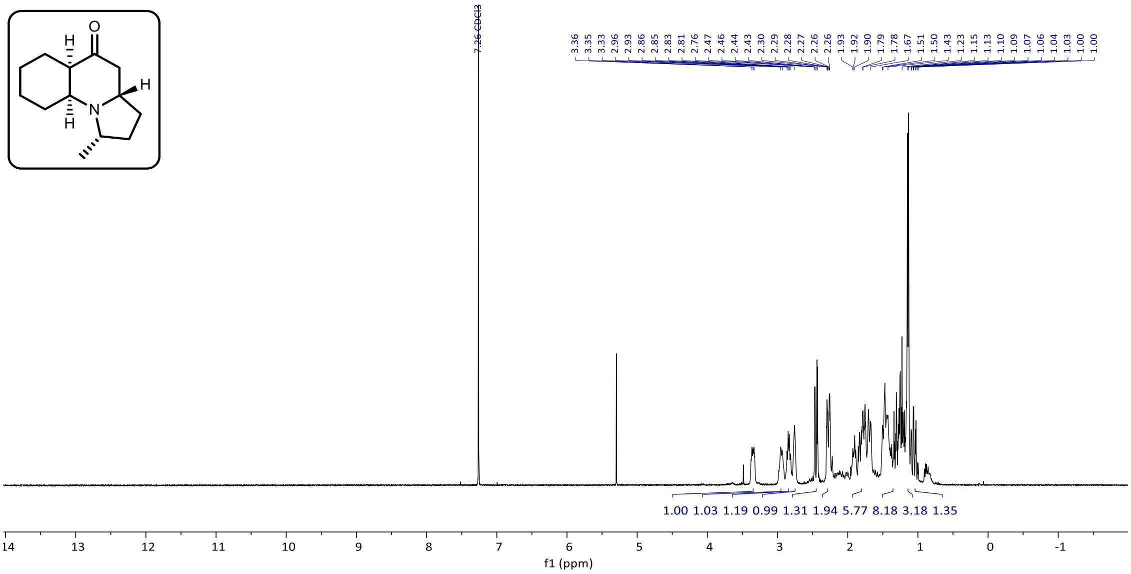

A101: *^{1}H NMR ($CDCl_3$) of compound 1S,3aS,5aR,9aS-**4c**.*

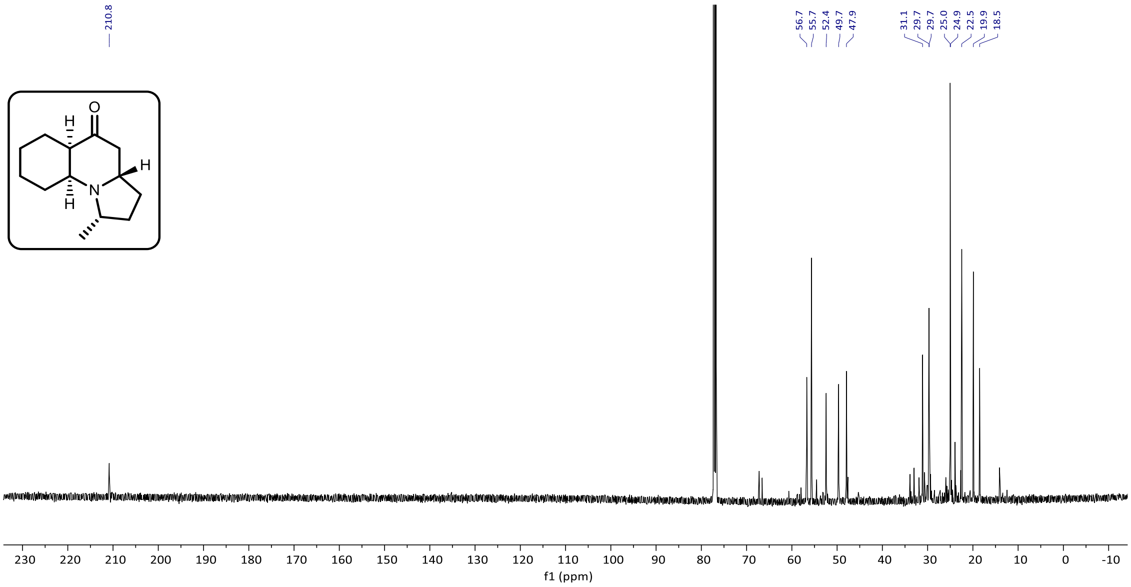

A102: *^{13}C NMR ($CDCl_3$) of compound 1S,3aS,5aR,9aS-**4c**.*

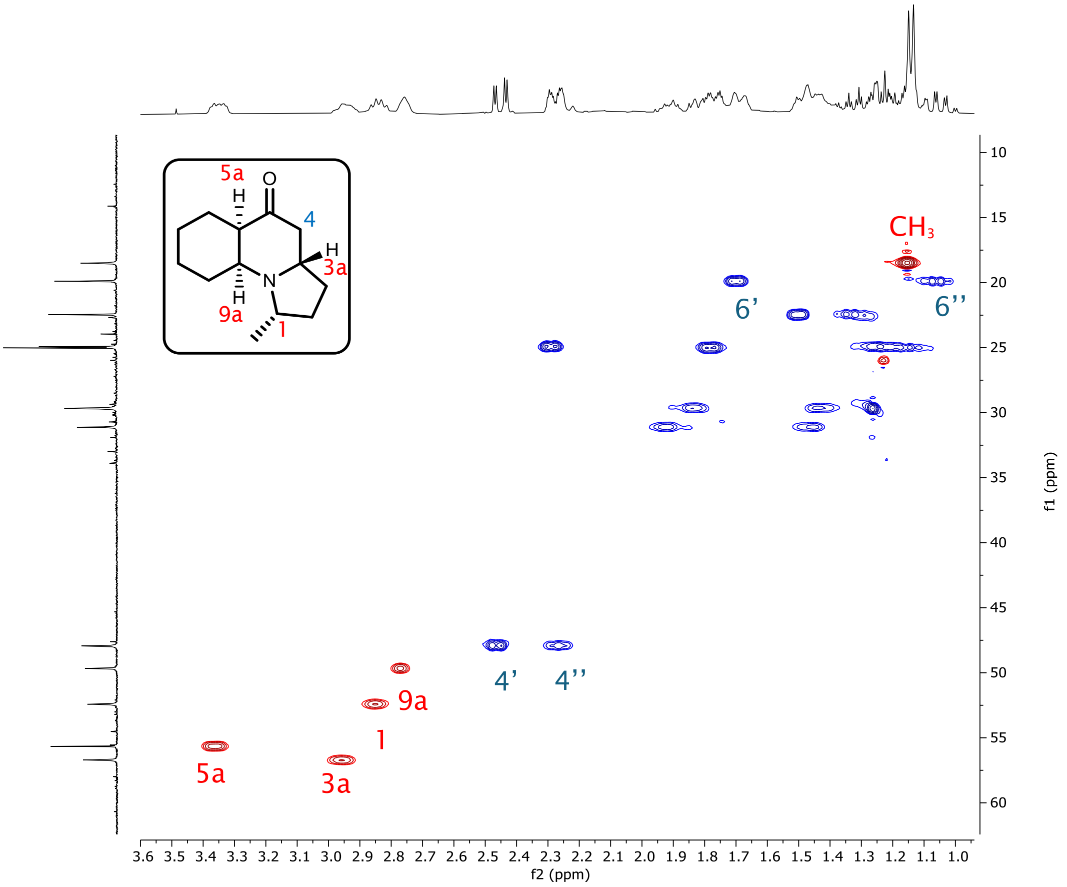

***A103:** HSQC ($CDCl_3$) of compound 1S,3aS,5aR,9aS-**4c**.*

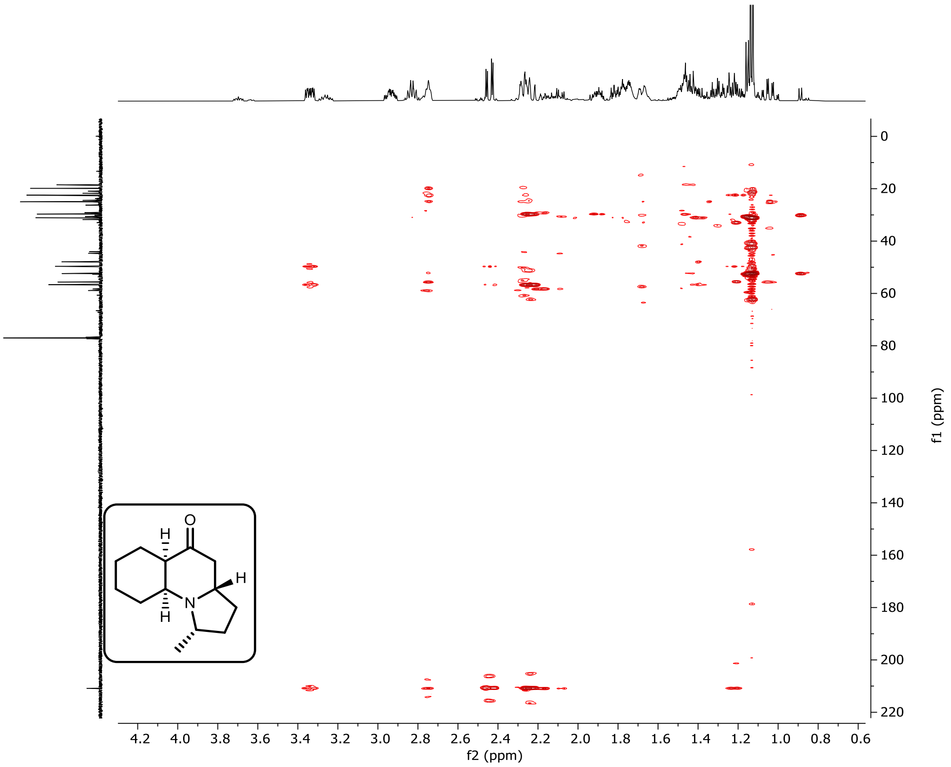

***A104:** HMBC ($CDCl_3$) of compound 1S,3aS,5aR,9aS-**4c**.*

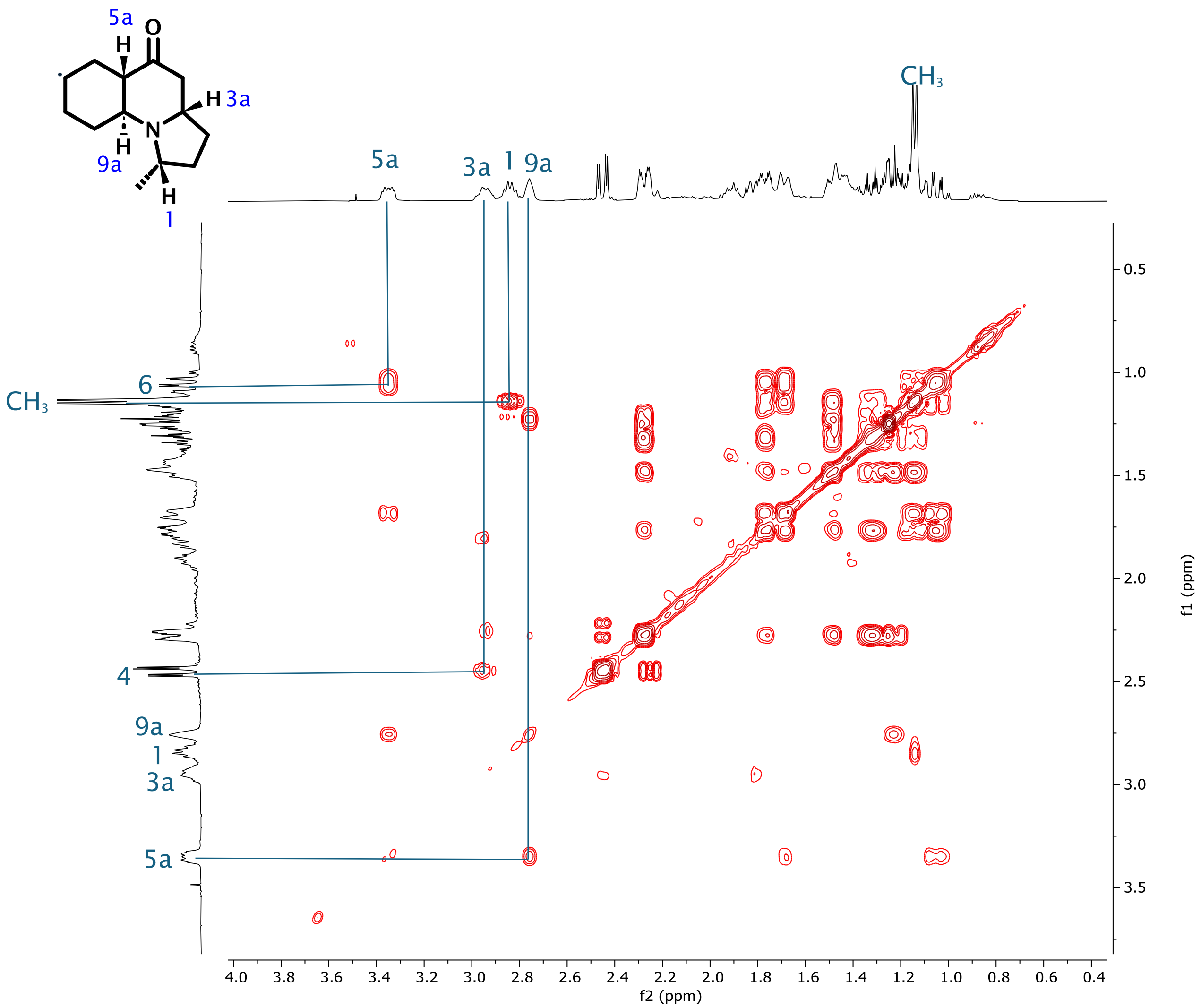

***A105:** COSY ($CDCl_3$) of compound 1S,3aS,5aR,9aS-**4c**.*

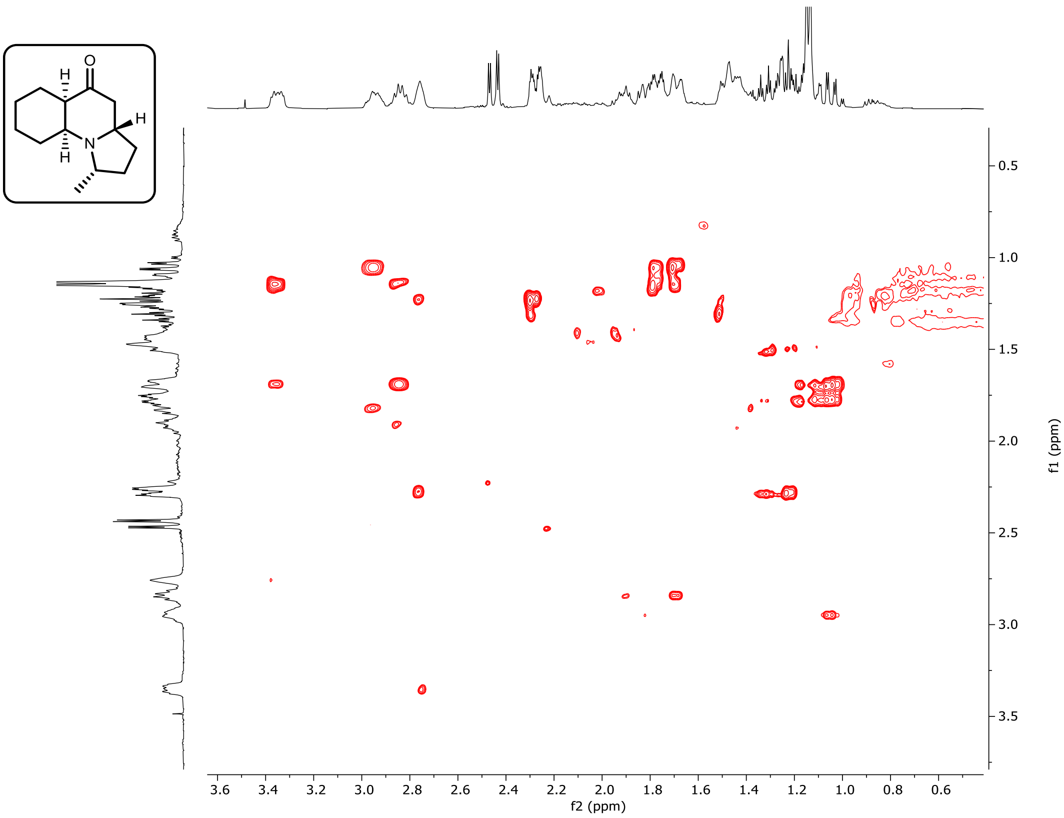

A106: *NOESY ($CDCl_3$) of compound 1S,3aS,5aR,9aS-**4c**.*

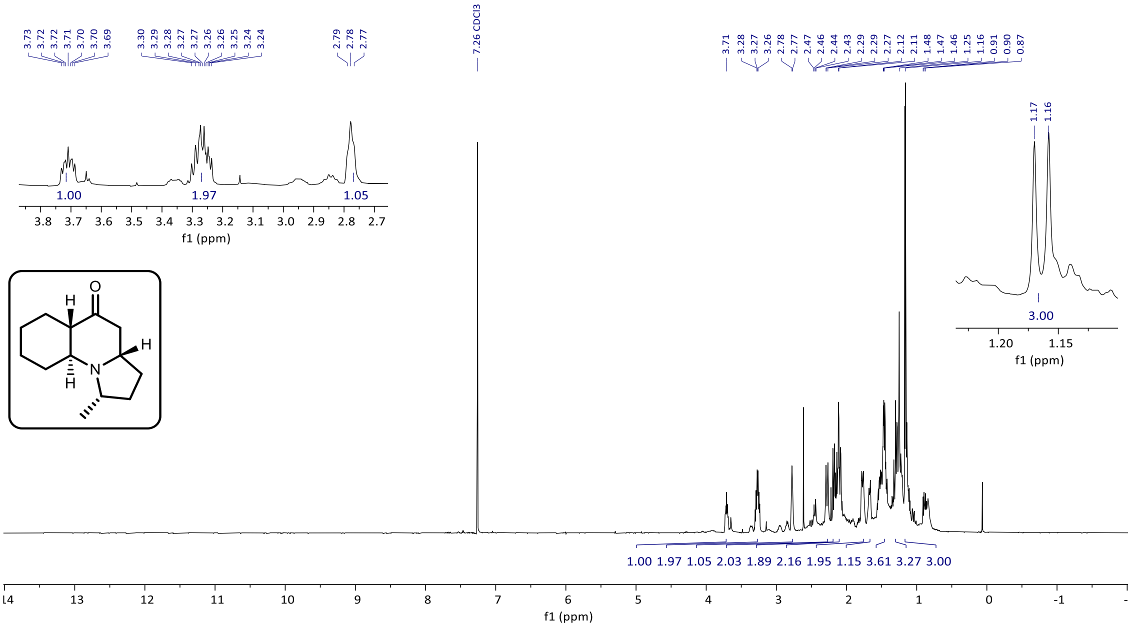

A107: *^{1}H NMR ($CDCl_3$) of compound 1S,3aS,5aS,9aS-**4c**.*

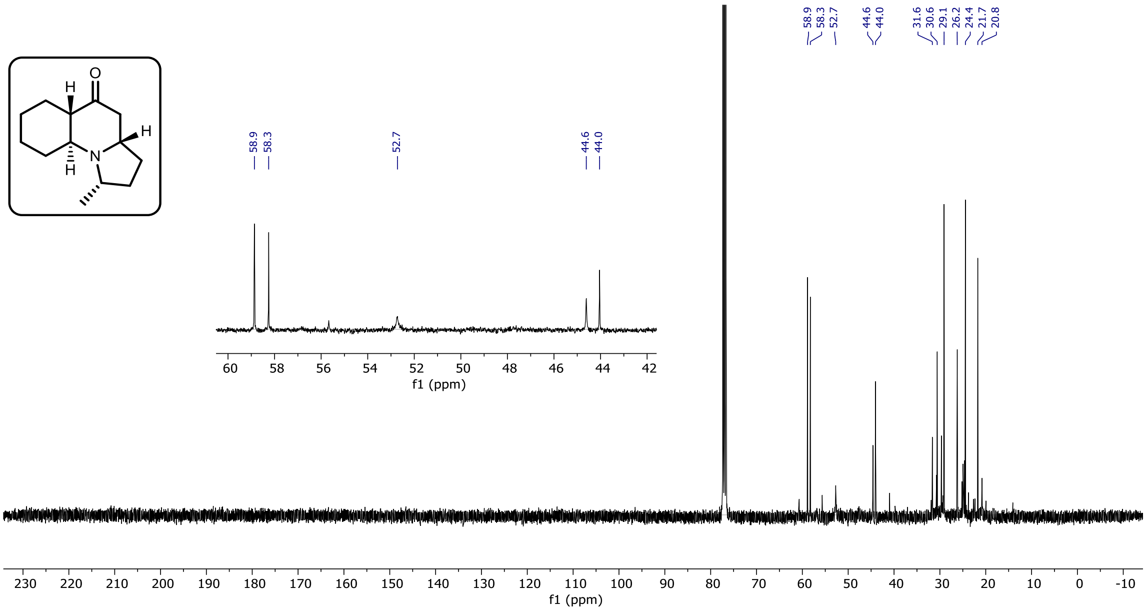

A108: *^{13}C NMR ($CDCl_3$) of compound 1S,3aS,5aS,9aS-**4c**.*

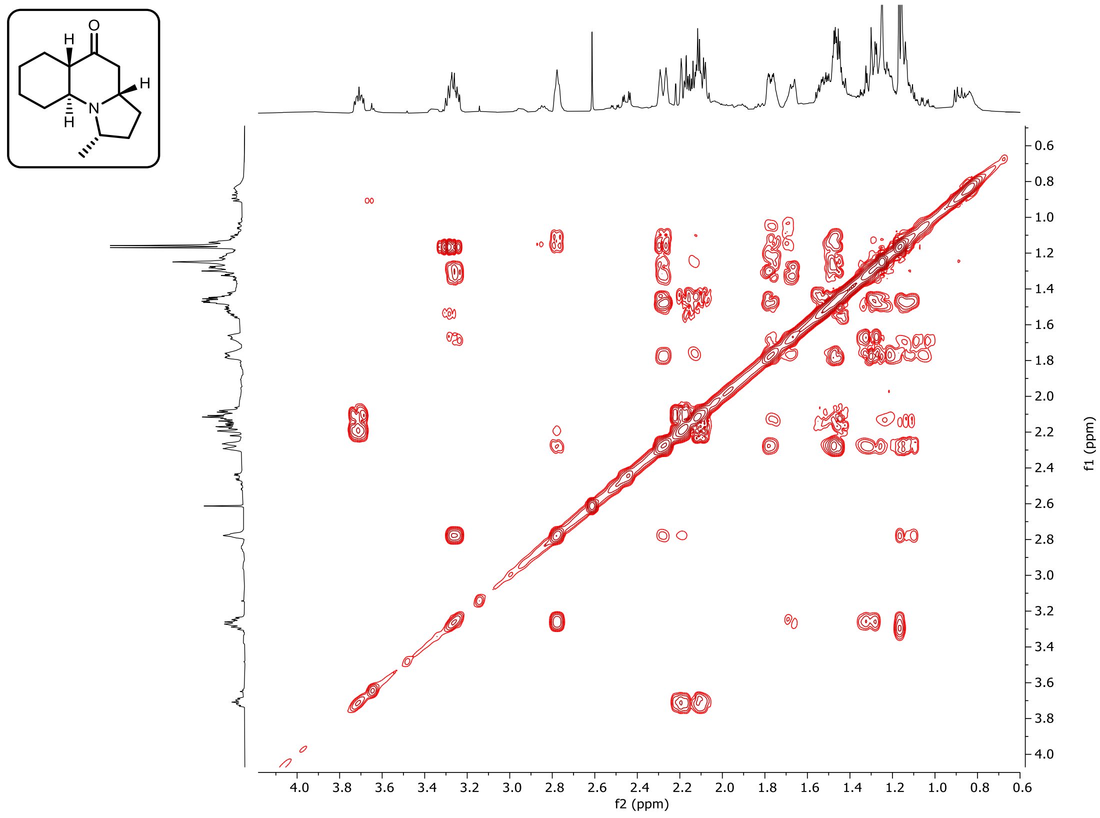

A109: *COSY ($CDCl_3$) of compound 1S,3aS,5aS,9aS-**4c**.*

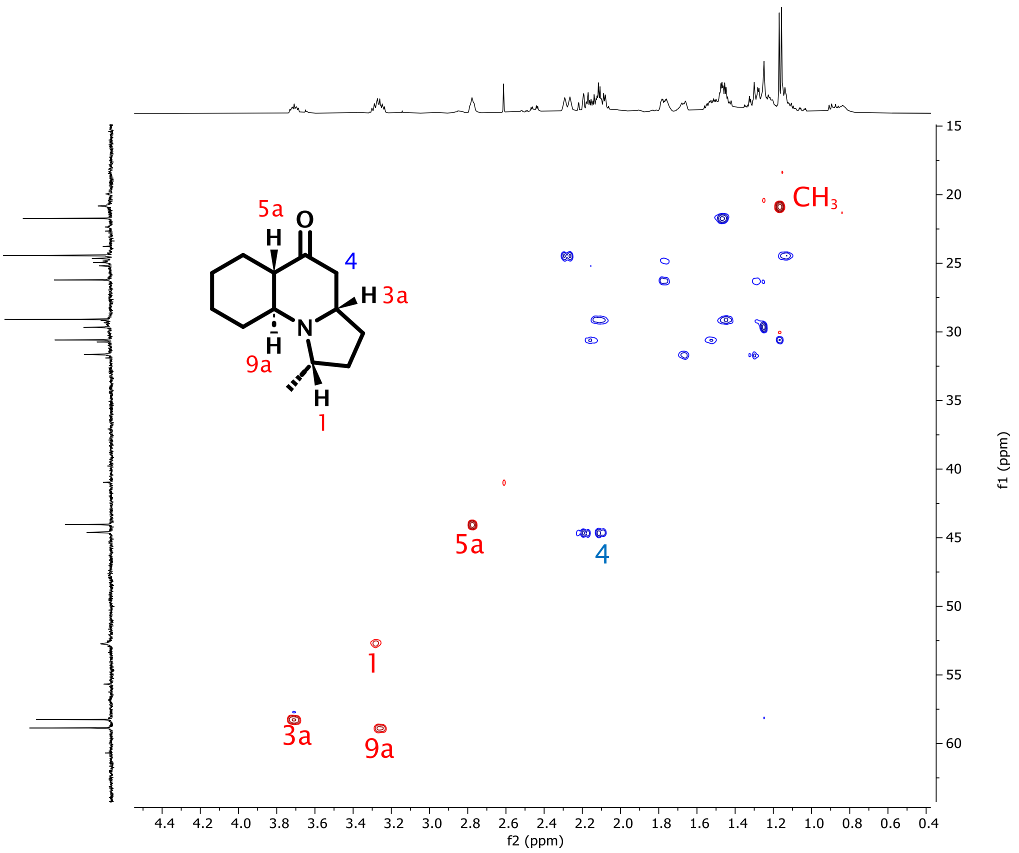

A110: *HSQC ($CDCl_3$) of compound 1S,3aS,5aS,9aS-**4c**.*

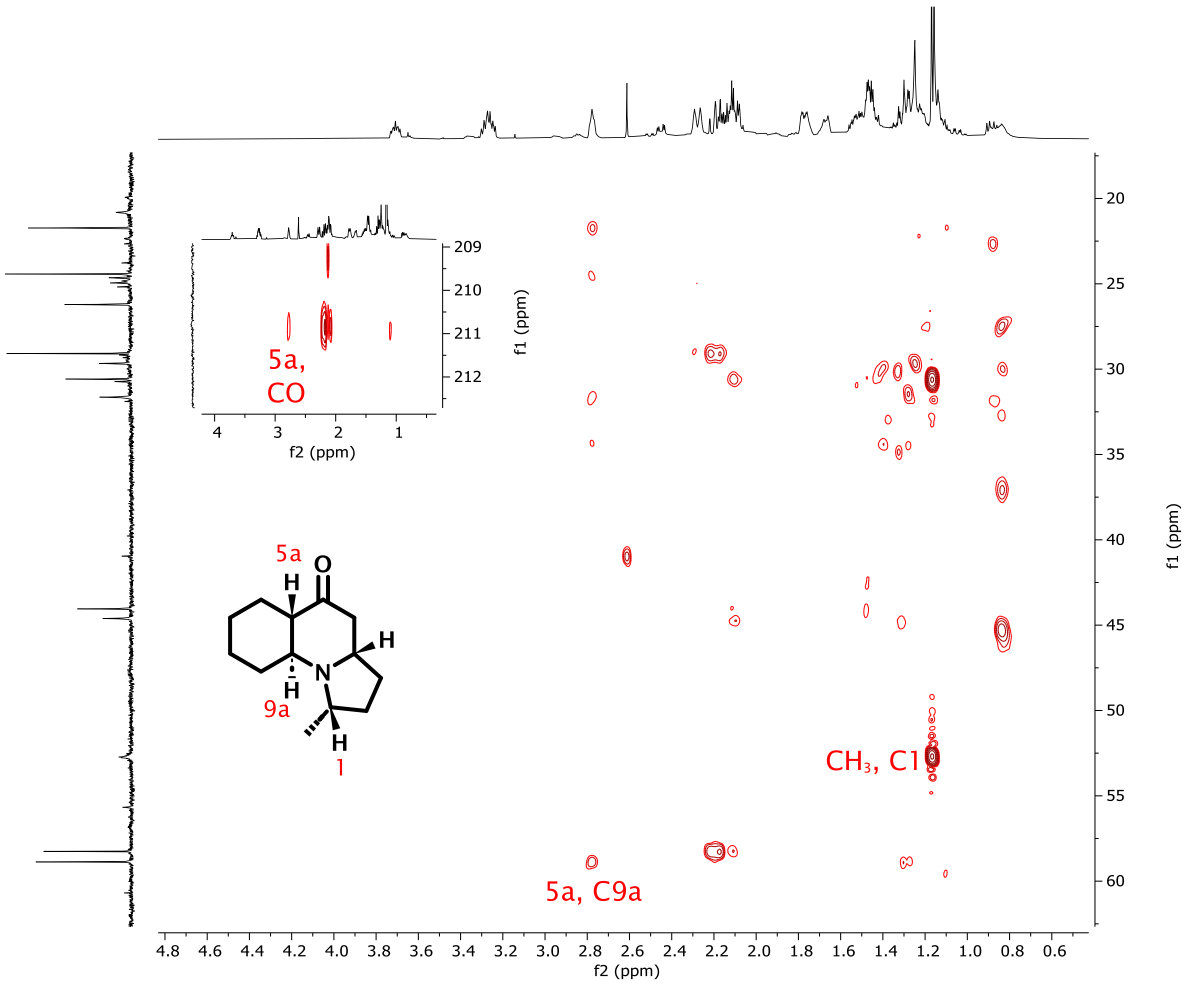

A111: *HMBC ($CDCl_3$) of compound 1S,3aS,5aS,9aS-**4c**.*

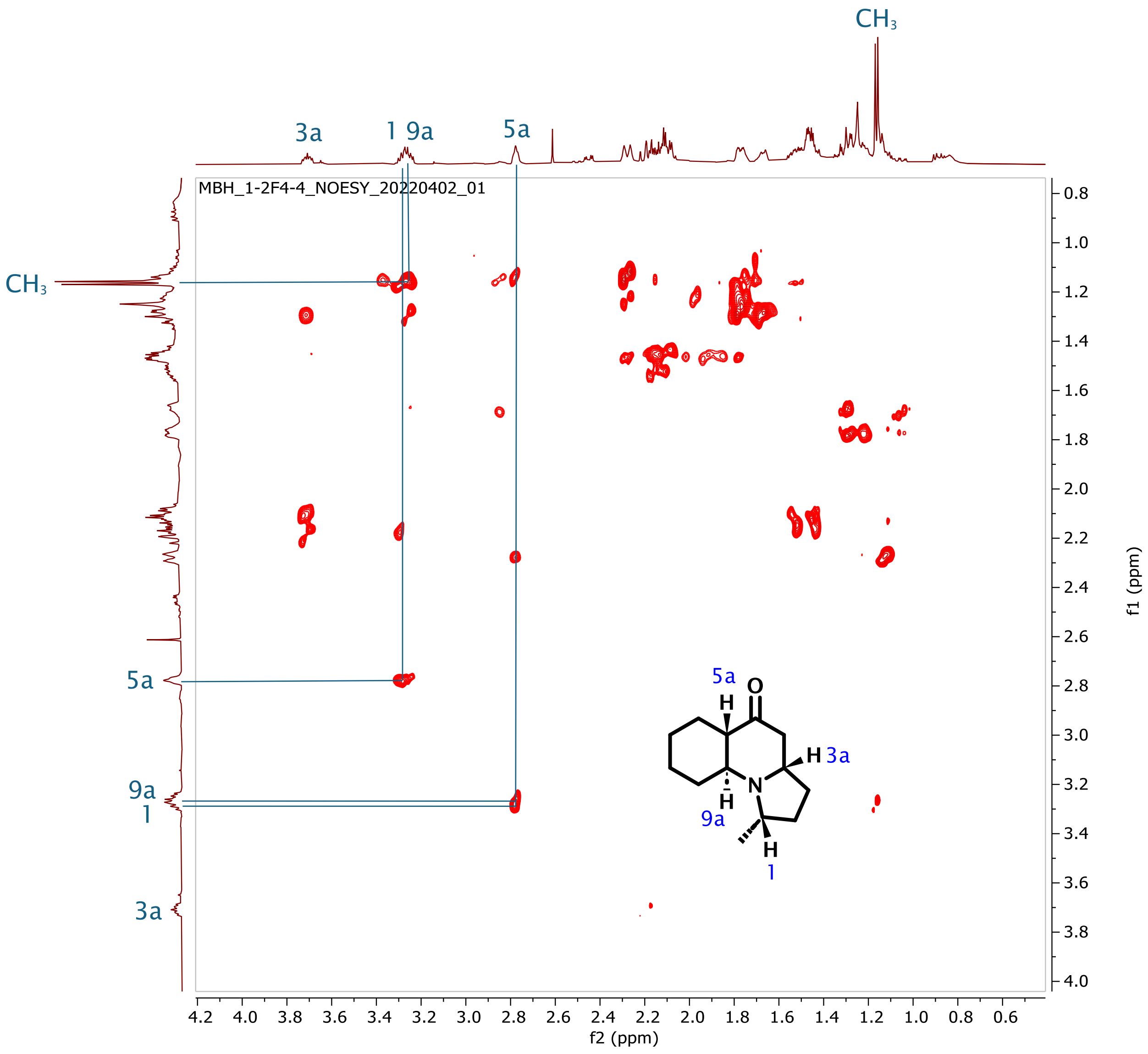

A112: *NOESY ($CDCl_3$) of compound (1S,3aS,5aS,9aS)-**4c**.*

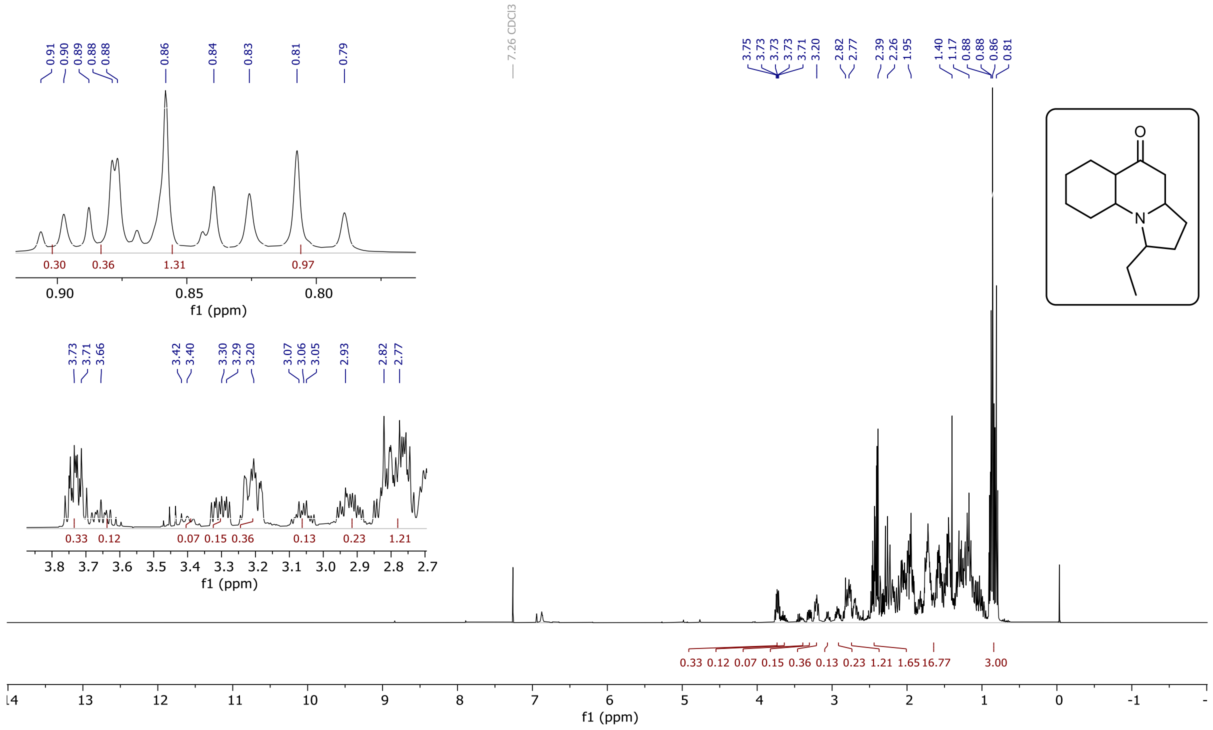

A113: ^{1}H NMR ($CDCl_3$) of compound **4d.**

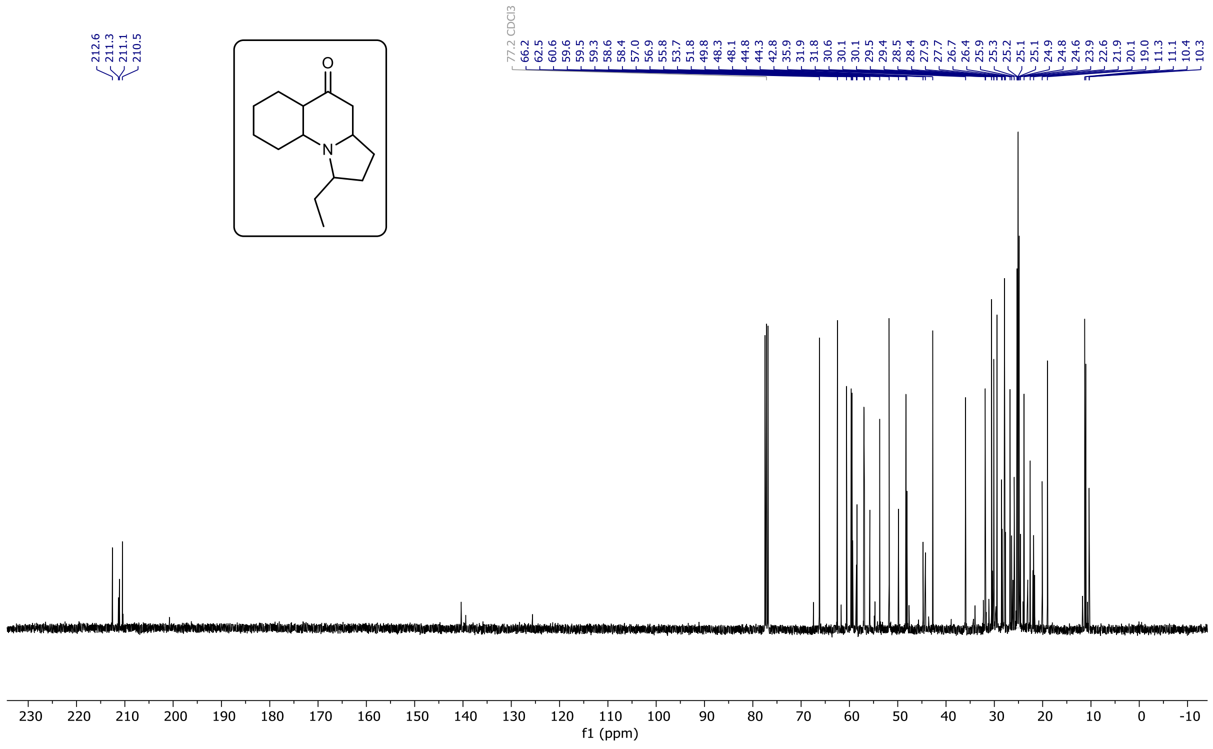

A114: ^{13}C NMR ($CDCl_3$) of compound **4d.**

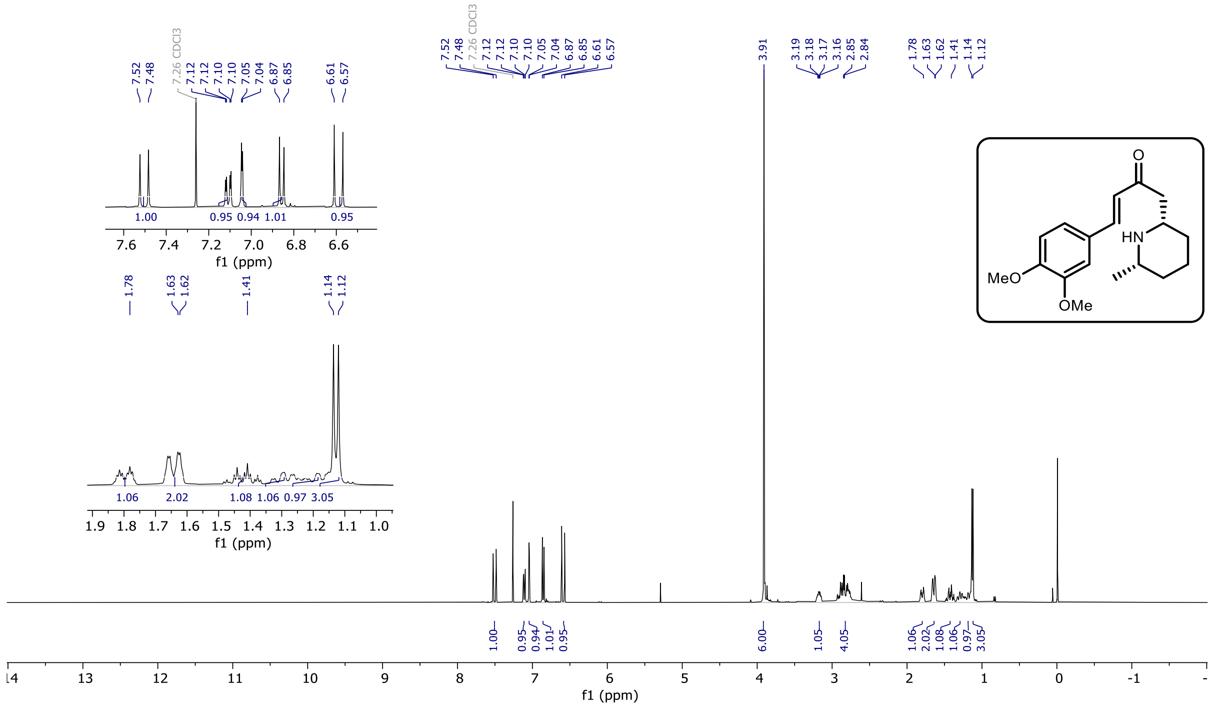

A115: *^{1}H NMR (CDCl$_3$) of compound (S,S)-**3e**.*

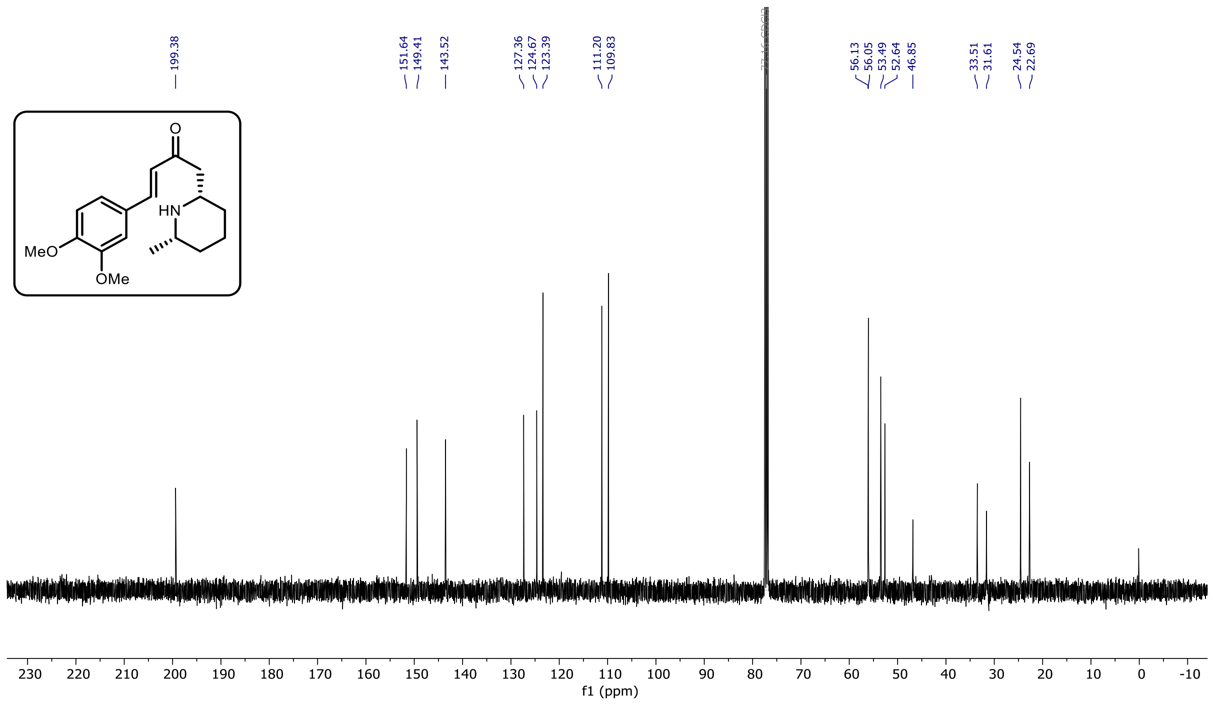

A116: *^{13}C NMR (CDCl$_3$) of compound (S,S)-**3e**.*

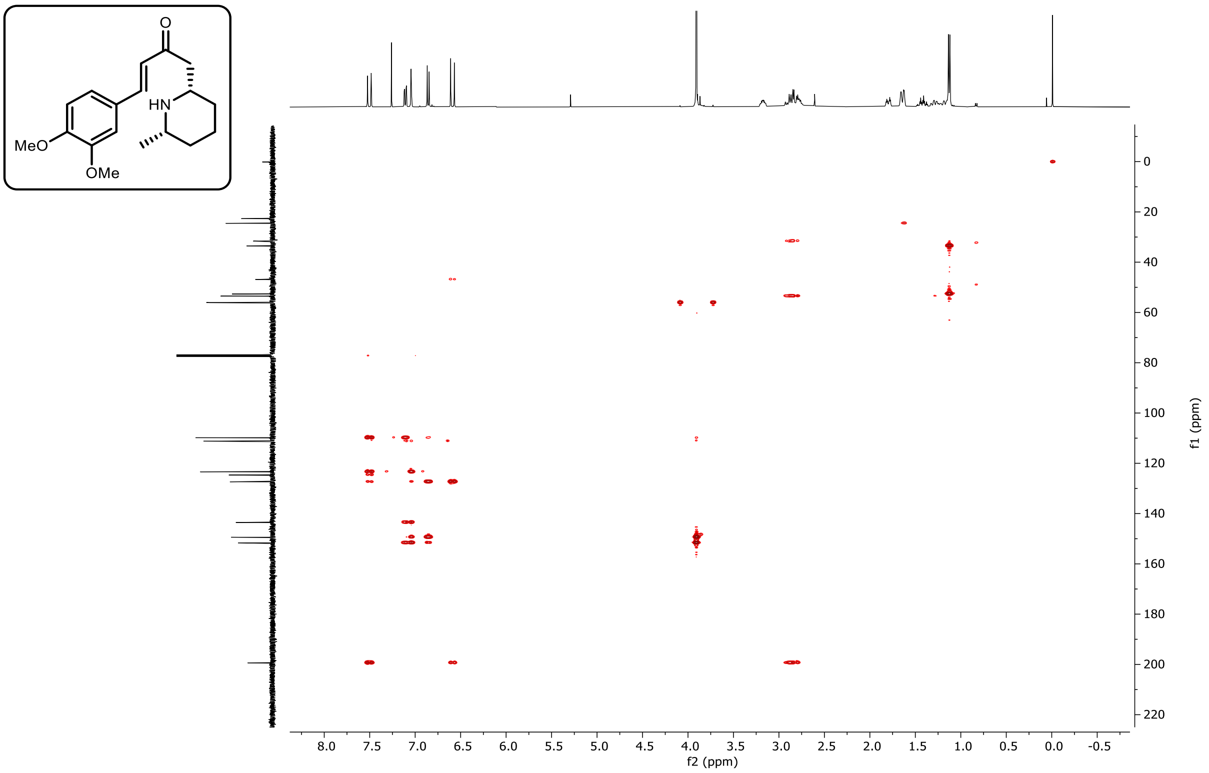

***A117:** HMBC ($CDCl_3$) of compound (S,S)-**3e**.*

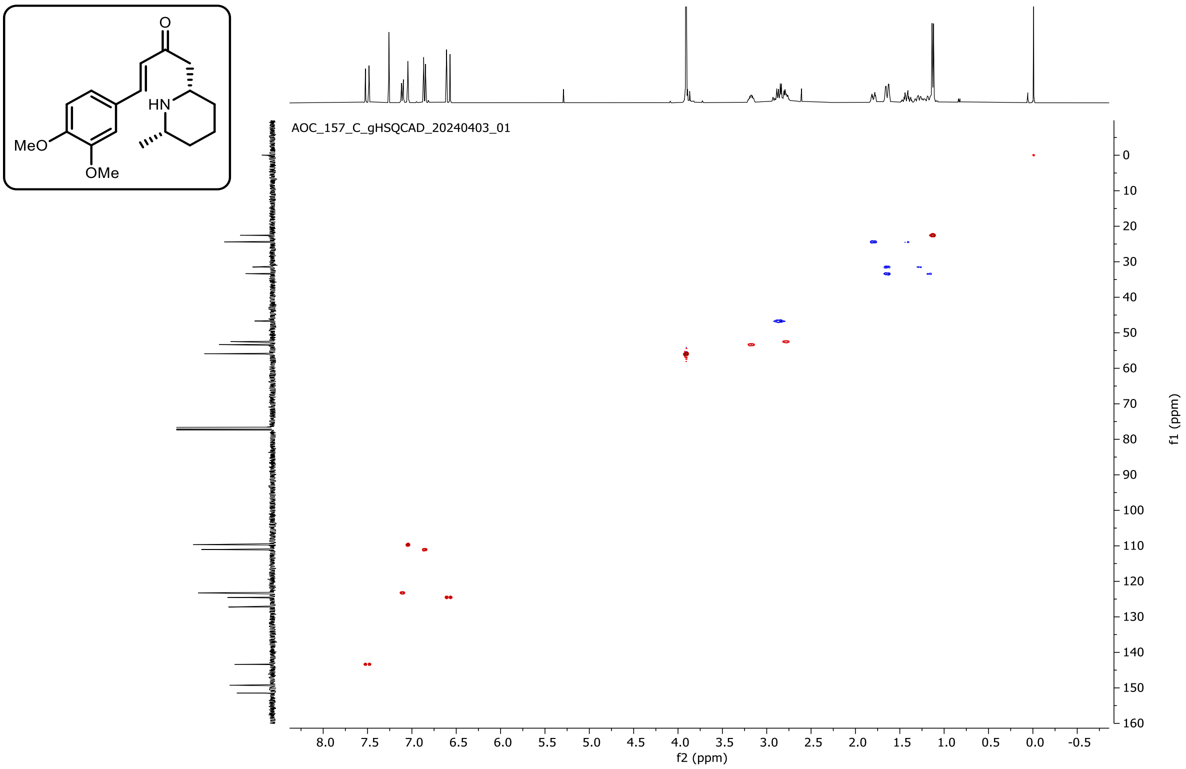

***A118:** HSQC ($CDCl_3$) of compound (S,S)-**3e**.*

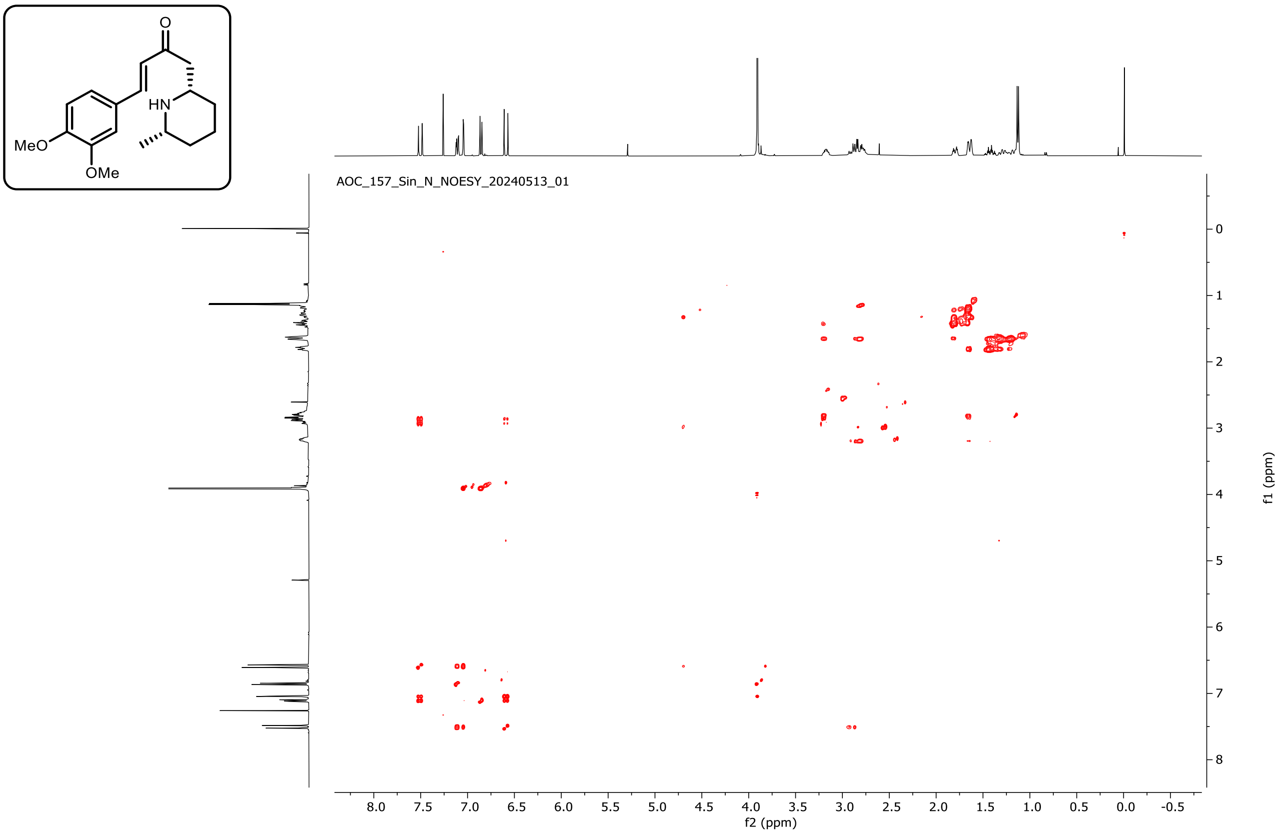

A119: *NOESY (CDCl$_3$) of compound (S,S)-**3e**.*

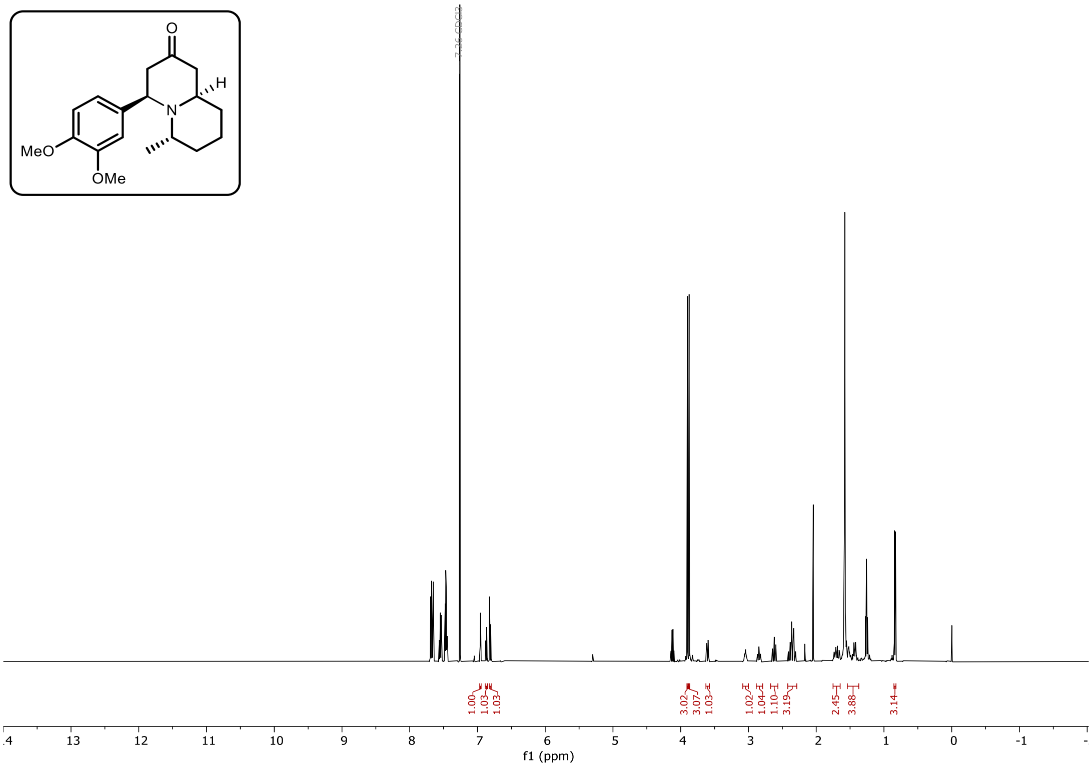

A120: *^{1}H NMR (CDCl$_3$) of compound (4R,6S,9aR)-**4e**.*

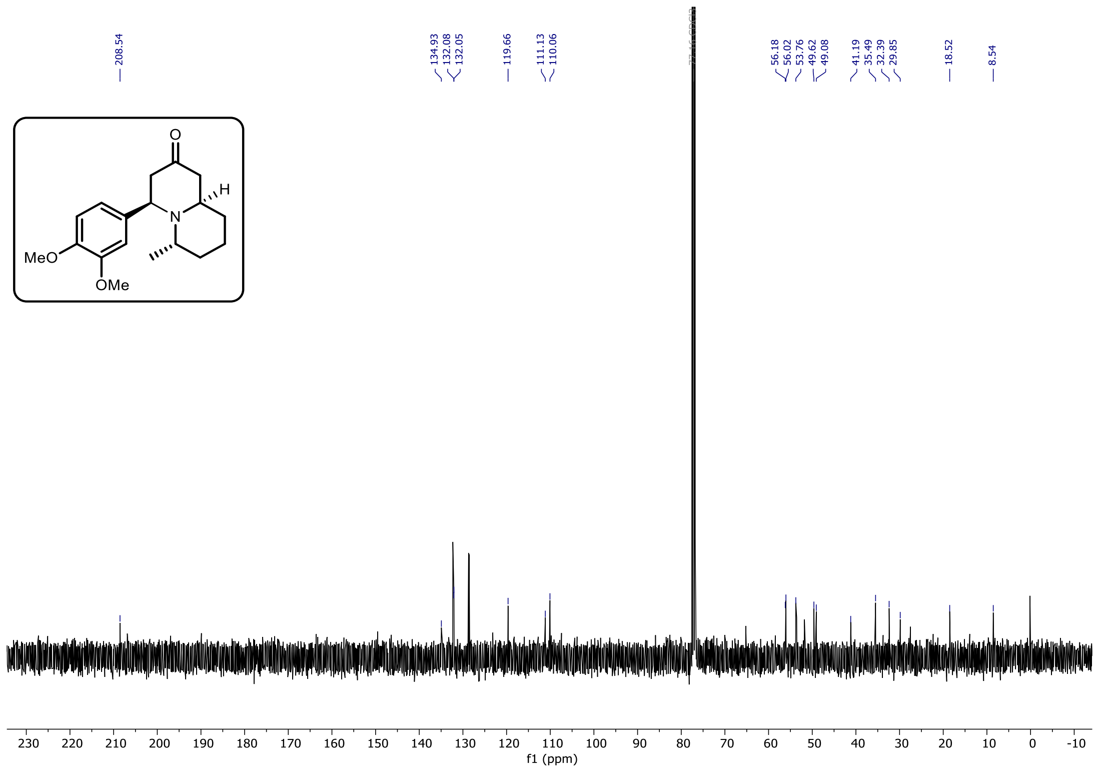

A121: *^{13}C NMR (CDCl$_3$) of compound* 4*R*,6*S*,9a*R*-**4e**.

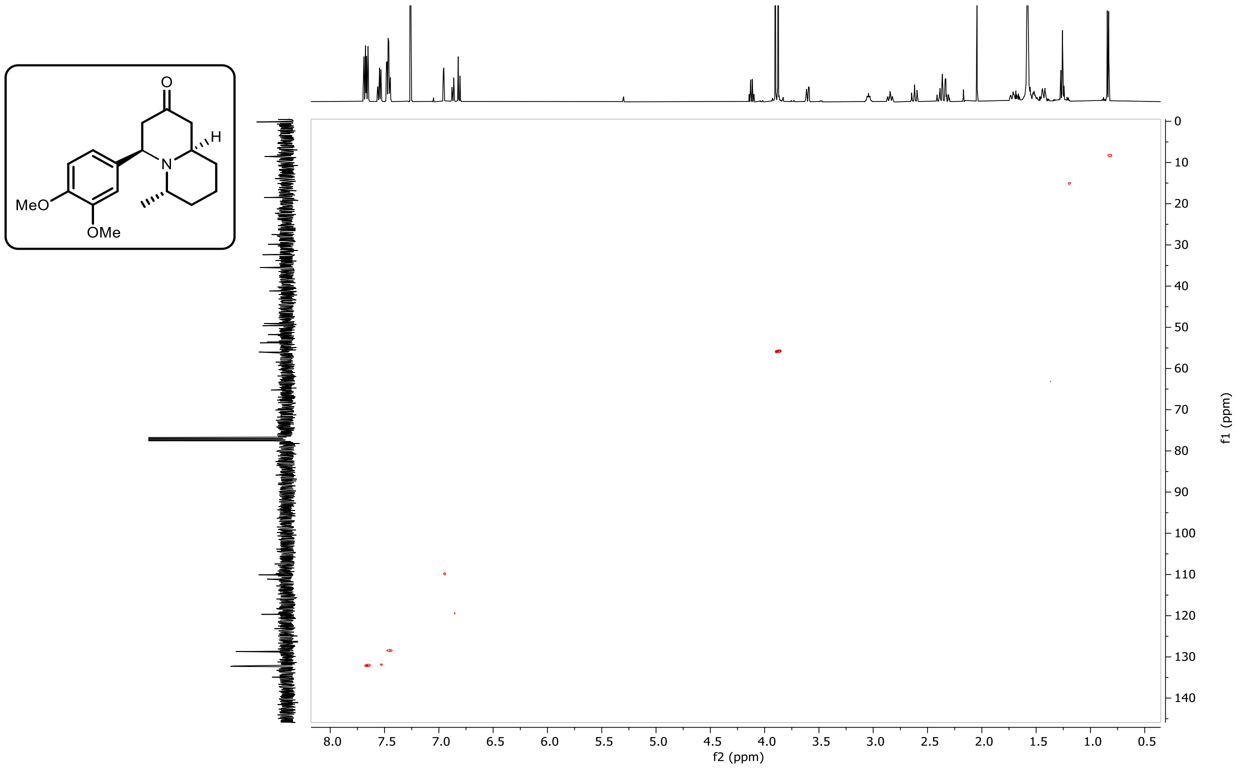

A122: *HSQC (CDCl$_3$) of compound* 4*R*,6*S*,9a*R*-**4e**.

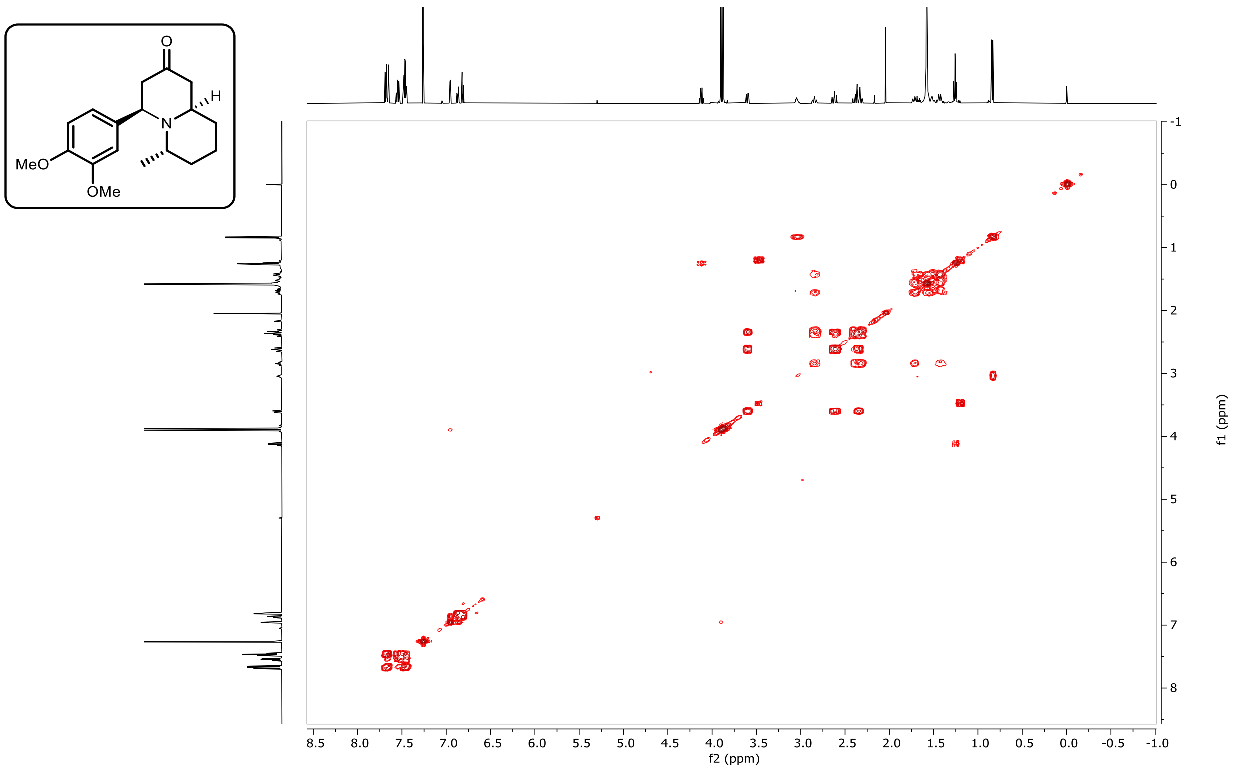

A123: *COSY NMR (CDCl$_3$) of compound* 4*R*,6*S*,9a*R*-**4e**.

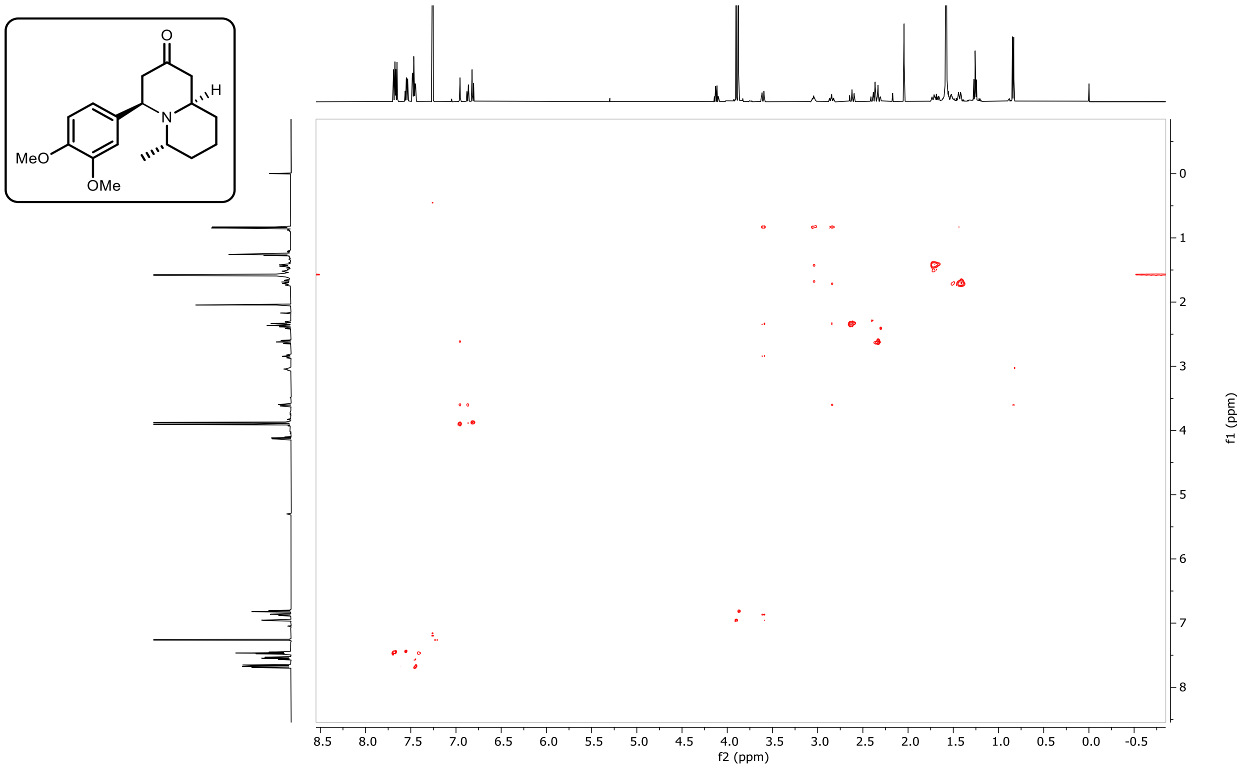

A124: *NOESY (CDCl$_3$) of compound* 4*R*,6*S*,9a*R*-**4e**.

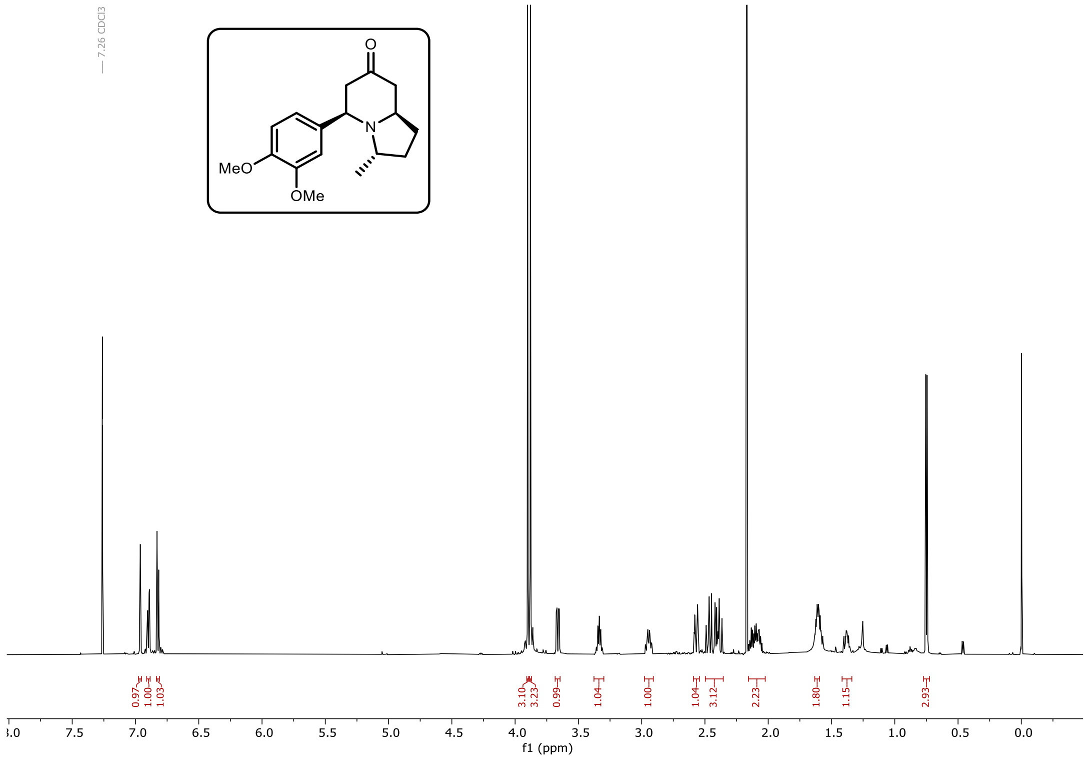

A125: *^{1}H NMR (CDCl$_3$) of compound 3S,5R,8aR-**4f**.*

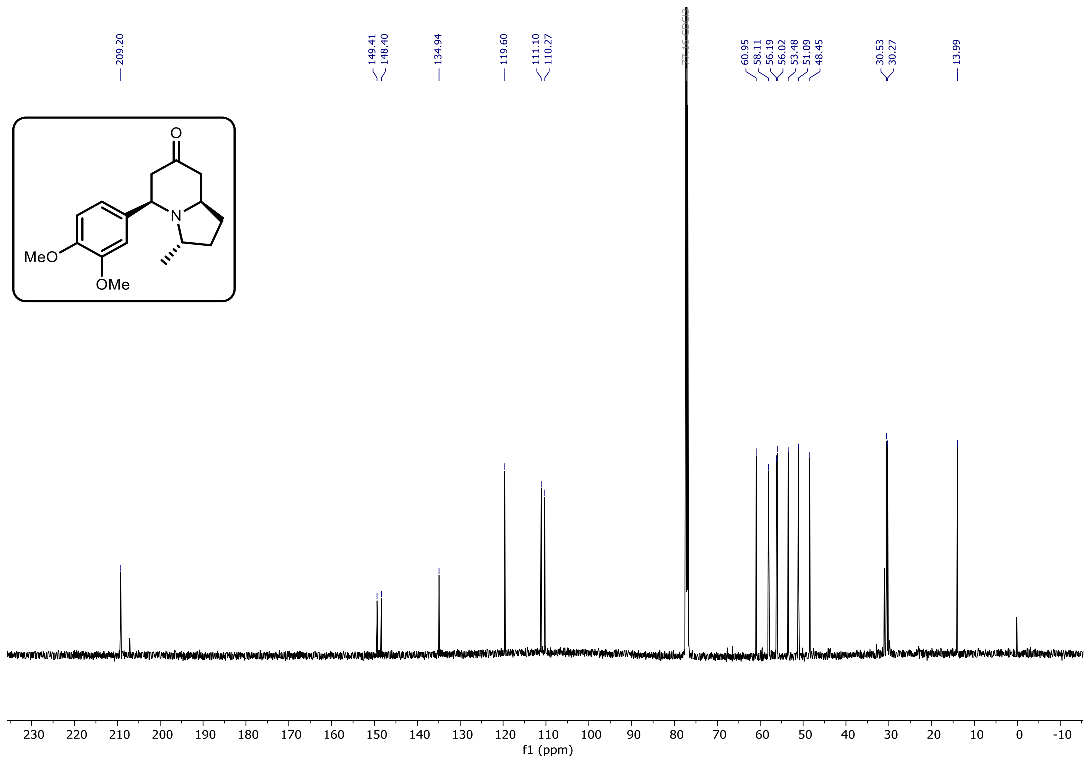

A126: *^{13}C NMR (CDCl$_3$) of compound 3S,5R,8aR-**4f**.*

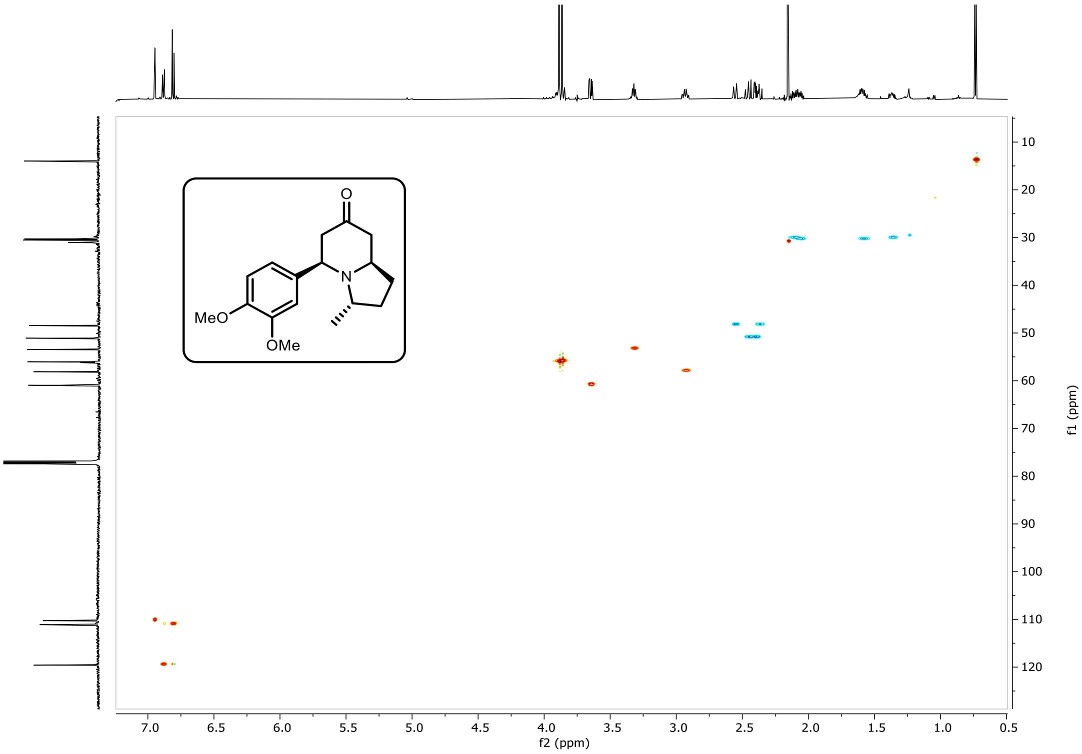

A127: *HSQC (CDCl$_3$) of compound 3S,5R,8aR-**4f**.*

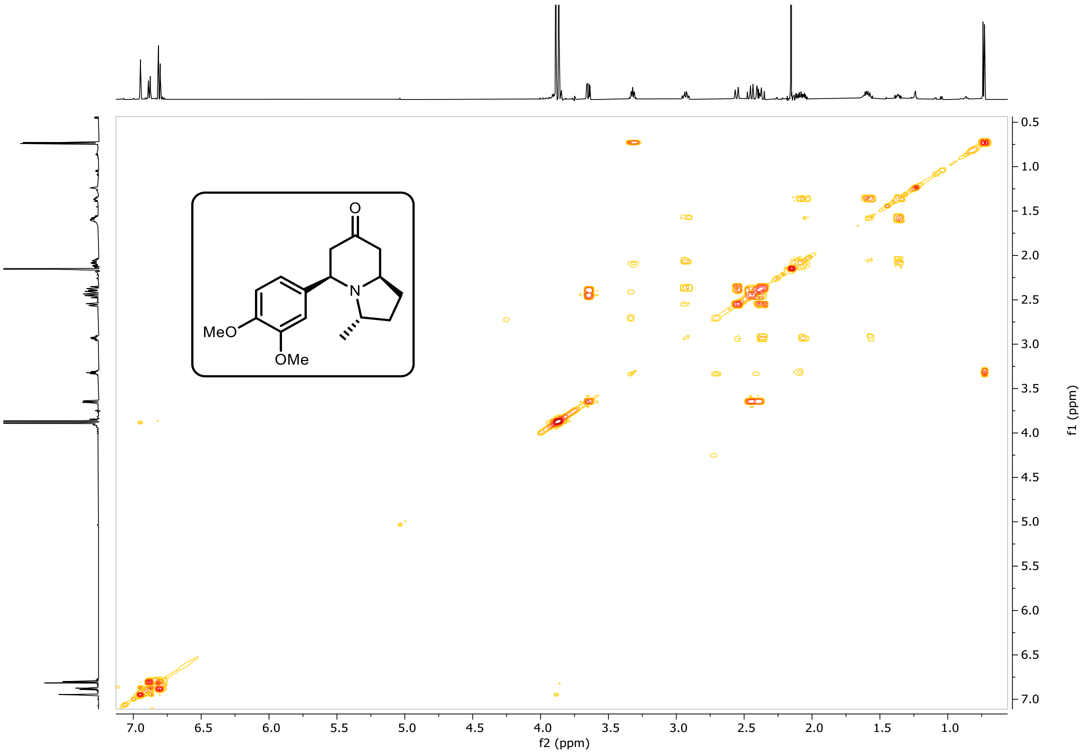

A128: *COSY NMR (CDCl$_3$) of compound 3S,5R,8aR-**4f**.*

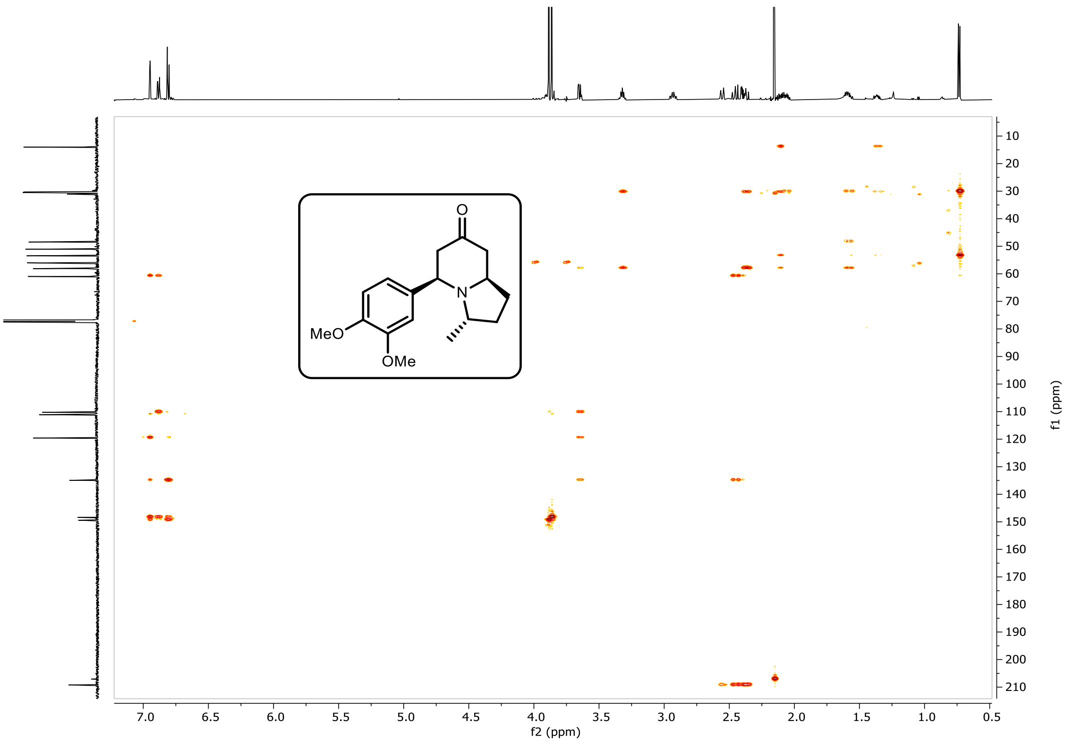

A129: *HMBC ($CDCl_3$) of compound 3S,5R,8aR-**4f**.*

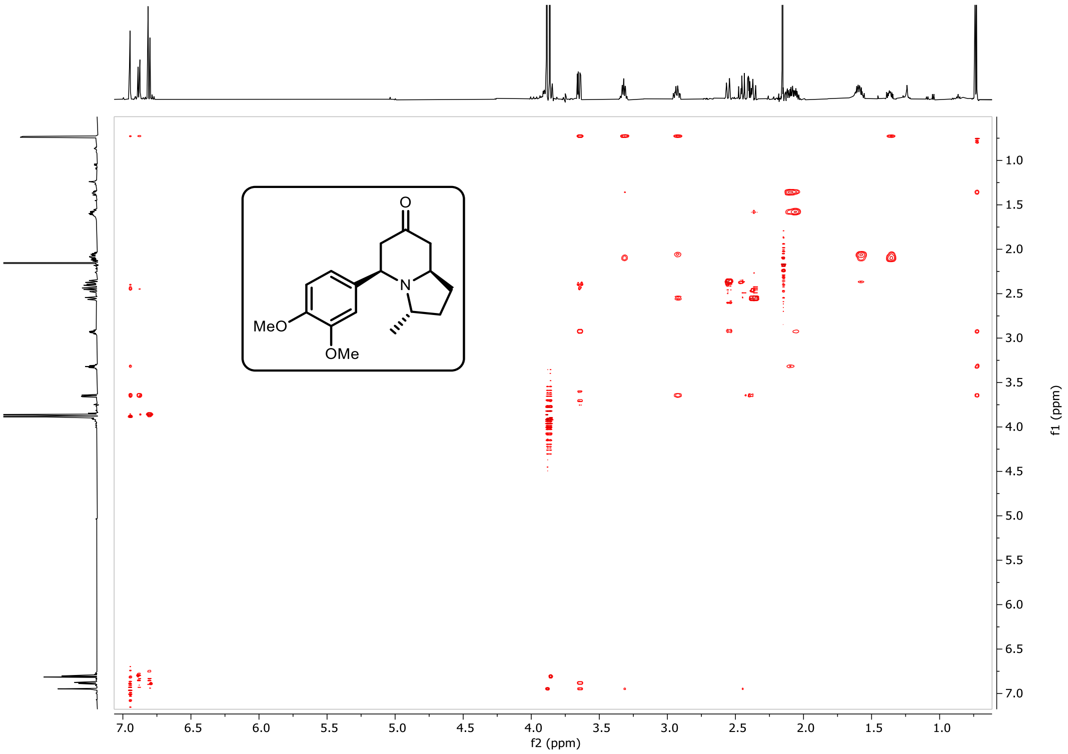

A130: *NOESY ($CDCl_3$) of compound 3S,5R,8aR-**4f**.*

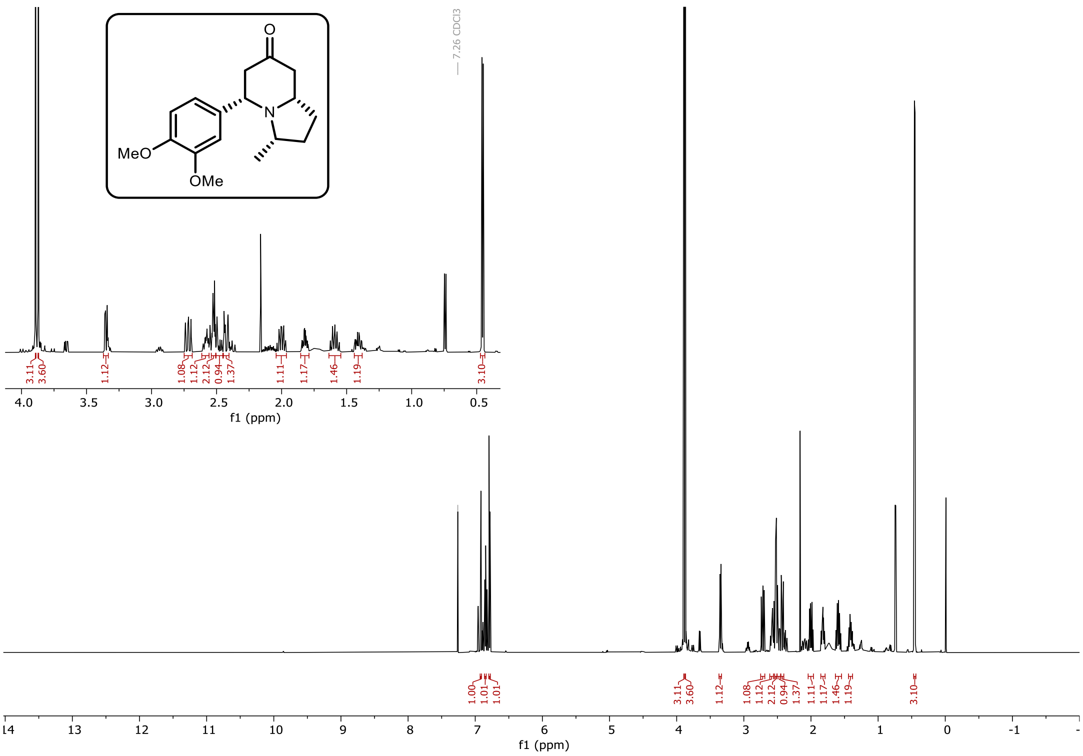

A131: *^{1}H NMR ($CDCl_3$) of compound 3S,5S,8aS-**4f**.*

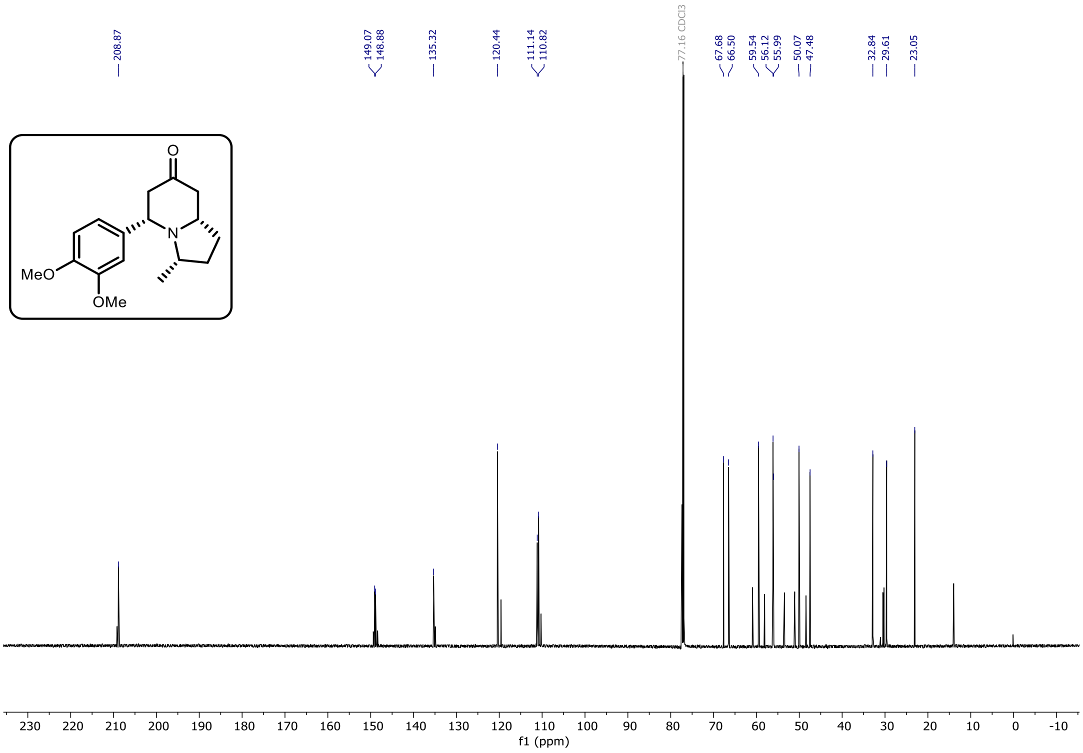

A132: *^{13}C NMR ($CDCl_3$) of compound 3S,5S,8aS-**4f**.*

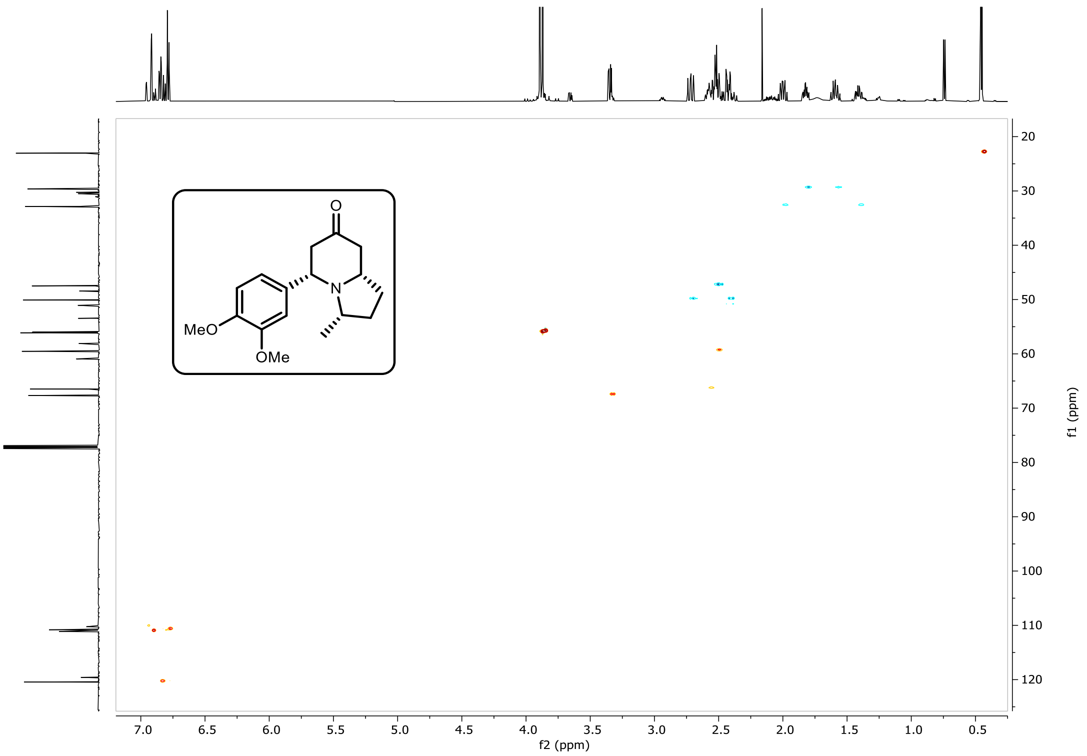

***A133:** HSQC (CDCl$_3$) of compound 3S,5S,8aS-**4f**.*

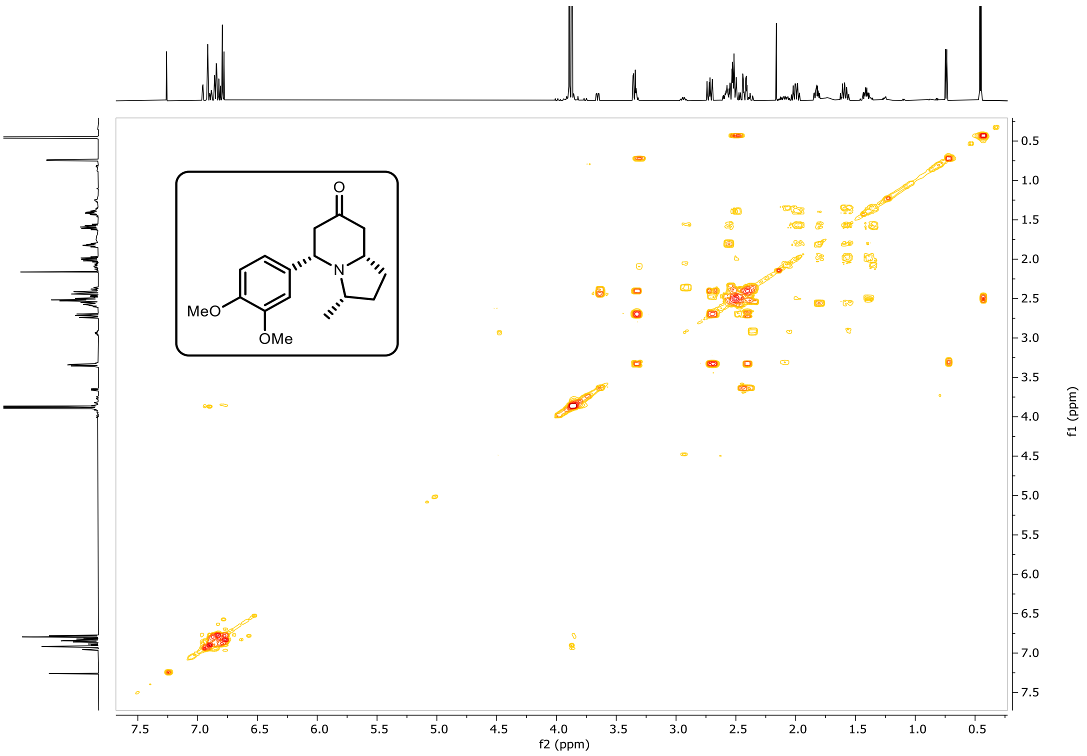

***A134:** COSY NMR (CDCl$_3$) of compound 3S,5S,8aS-**4f**.*

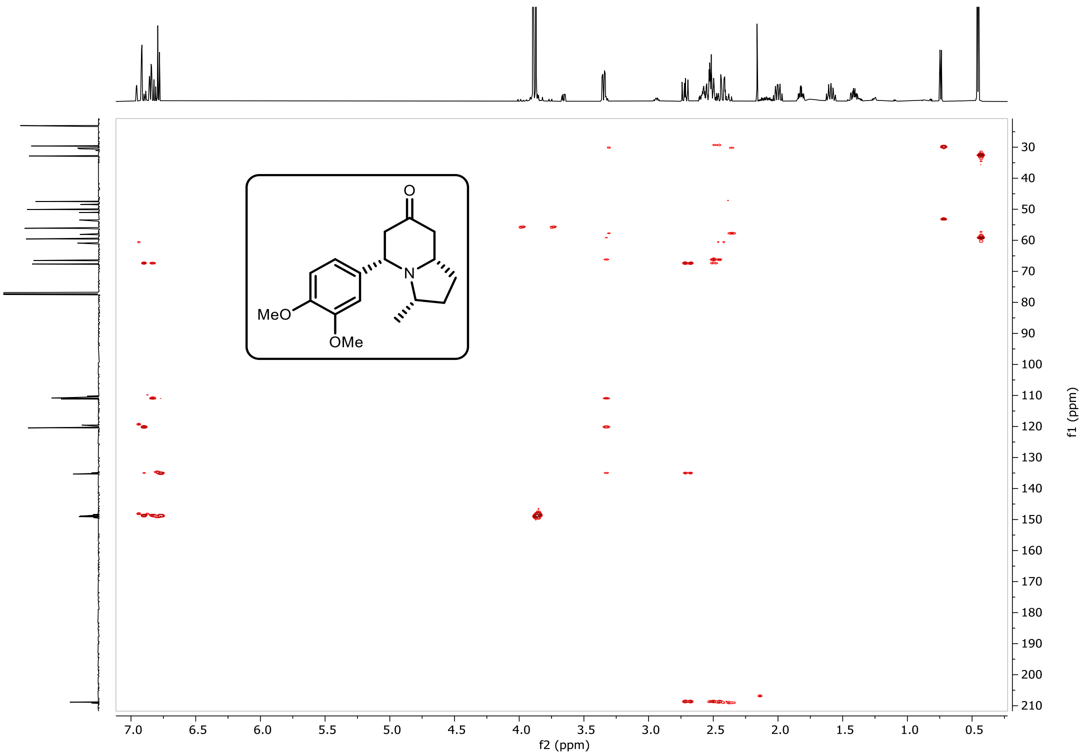

***A135:** HMBC (CDCl$_3$) of compound 3S,5S,8aS-**4f**.*

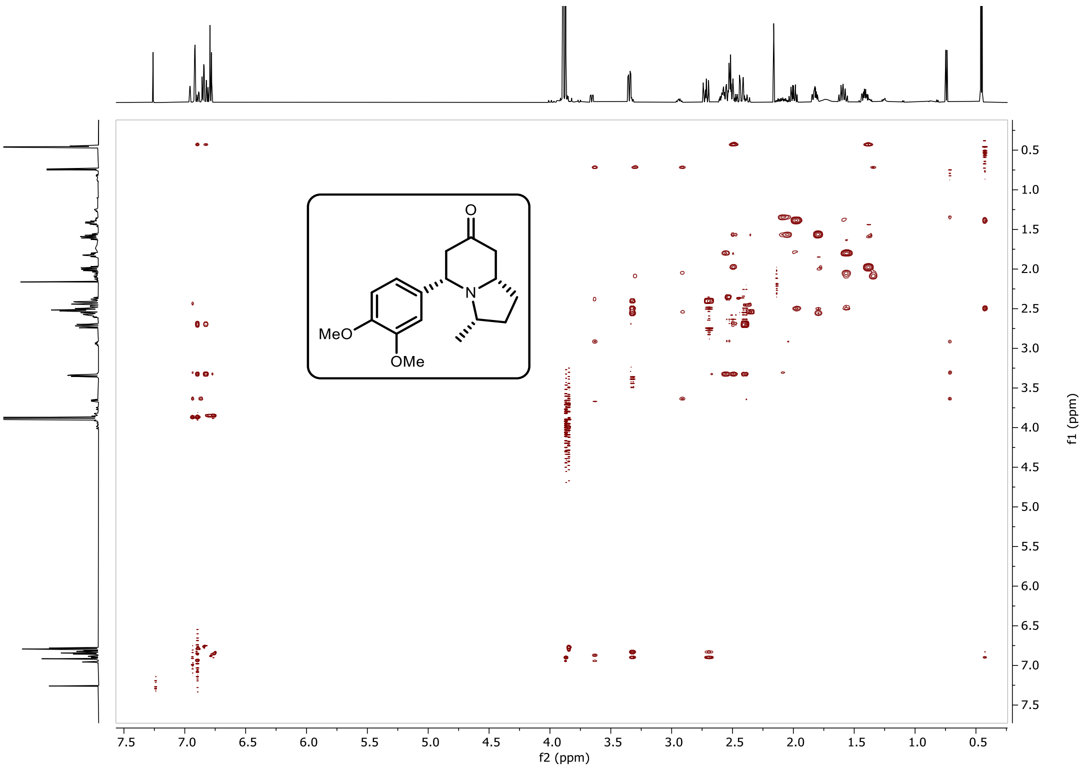

***A136:** NOESY (CDCl$_3$) of compound 3S,5S,8aS-**4f**.*

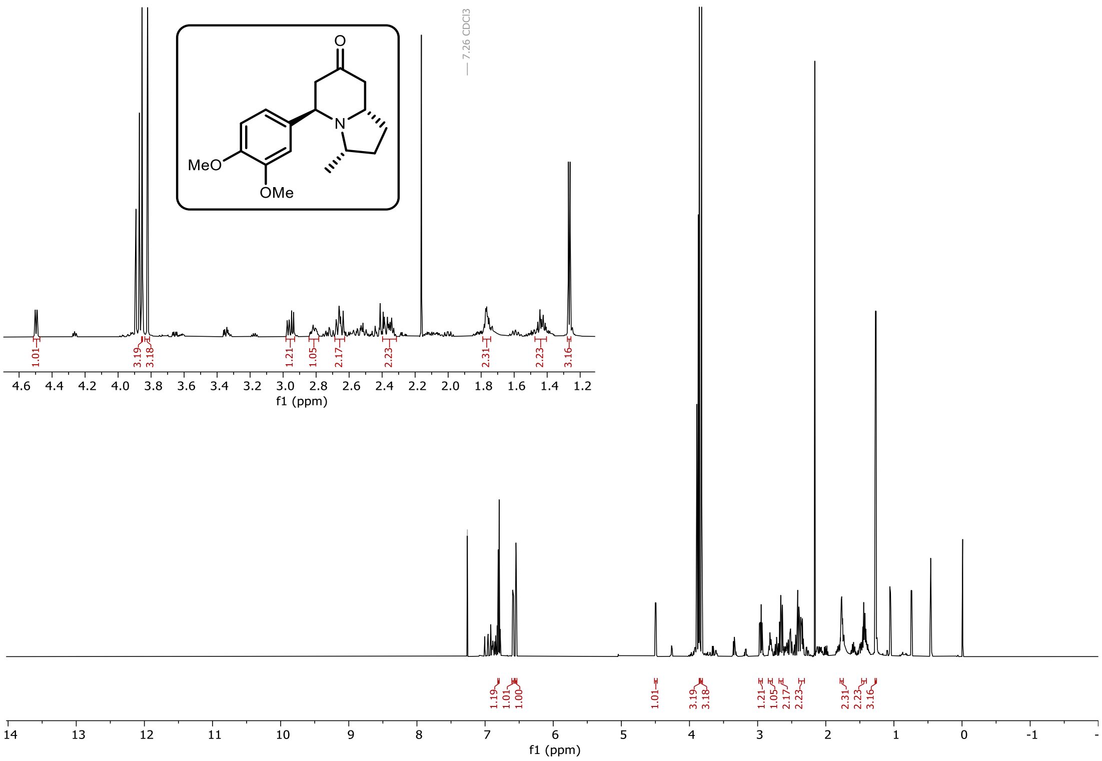

A137: *^{1}H NMR (CDCl$_3$) of compound 3S,5R,8aS-**4f** (enriched).*

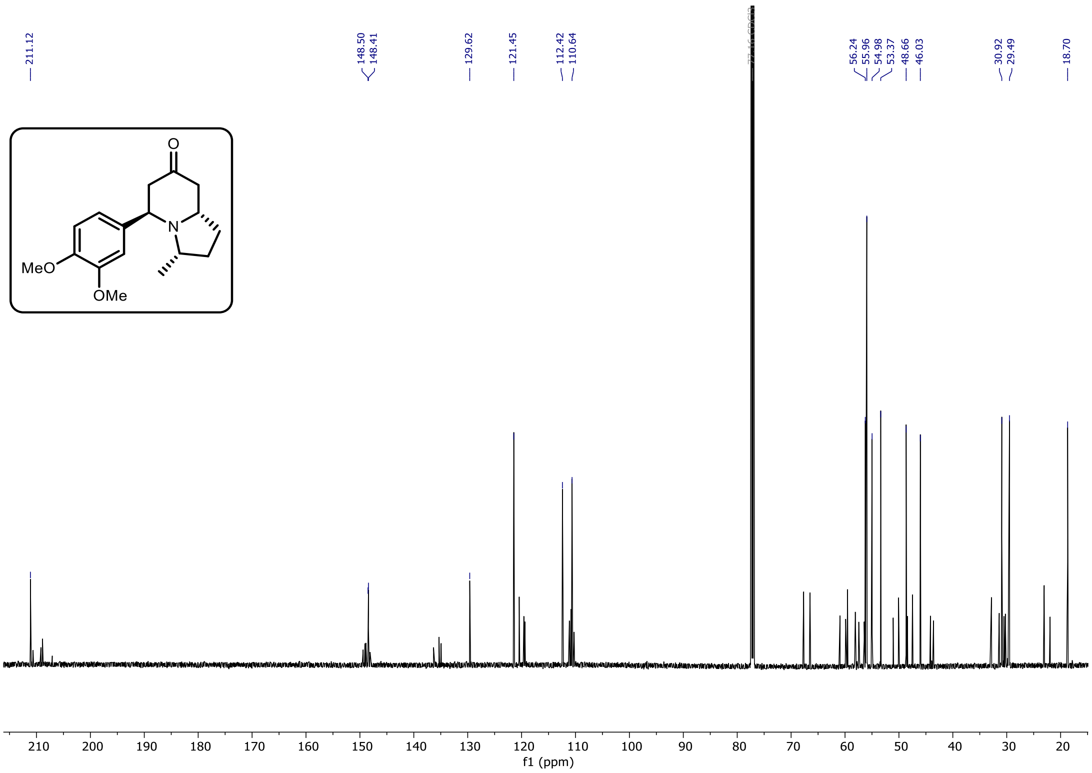

A138: *^{13}C NMR (CDCl$_3$) of compound 3S,5R,8aS-**4f** (enriched).*

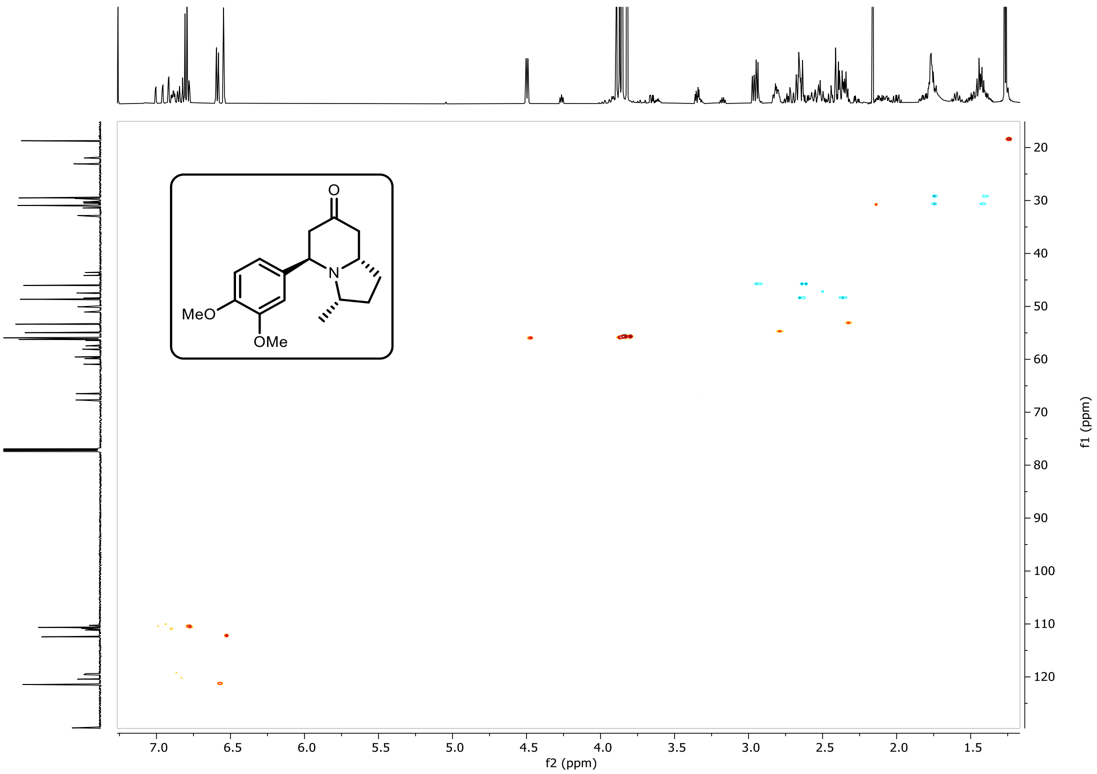

***A139:** HSQC ($CDCl_3$) of compound 3S,5R,8aS-**4f** (enriched).*

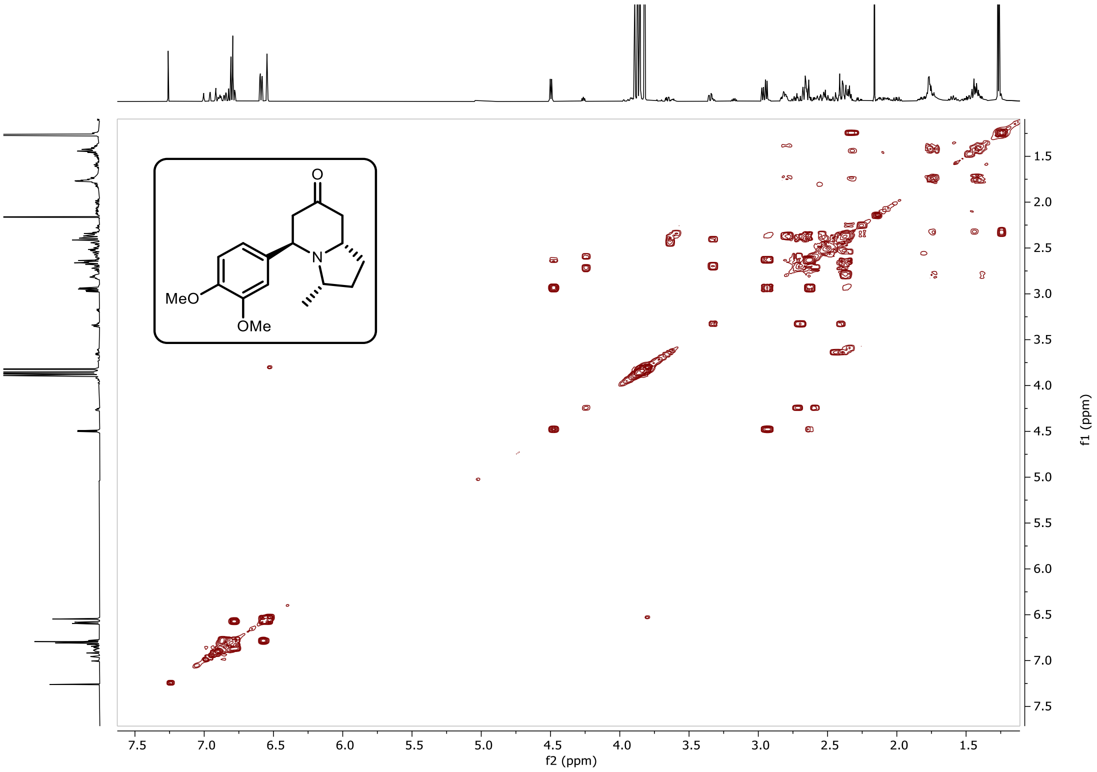

***A140:** COSY NMR ($CDCl_3$) of compound 3S,5R,8aS-**4f** (enriched).*

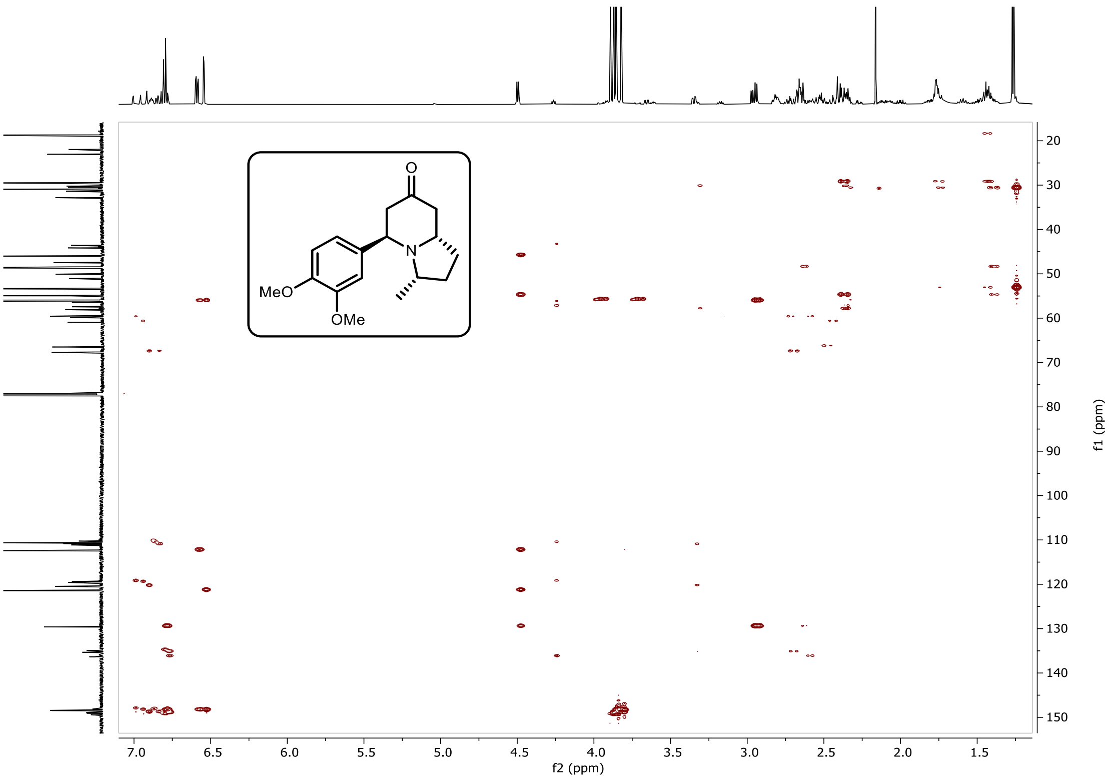

A141: *HMBC (CDCl$_3$) of compound 3S,5R,8aS-**4f** (enriched).*

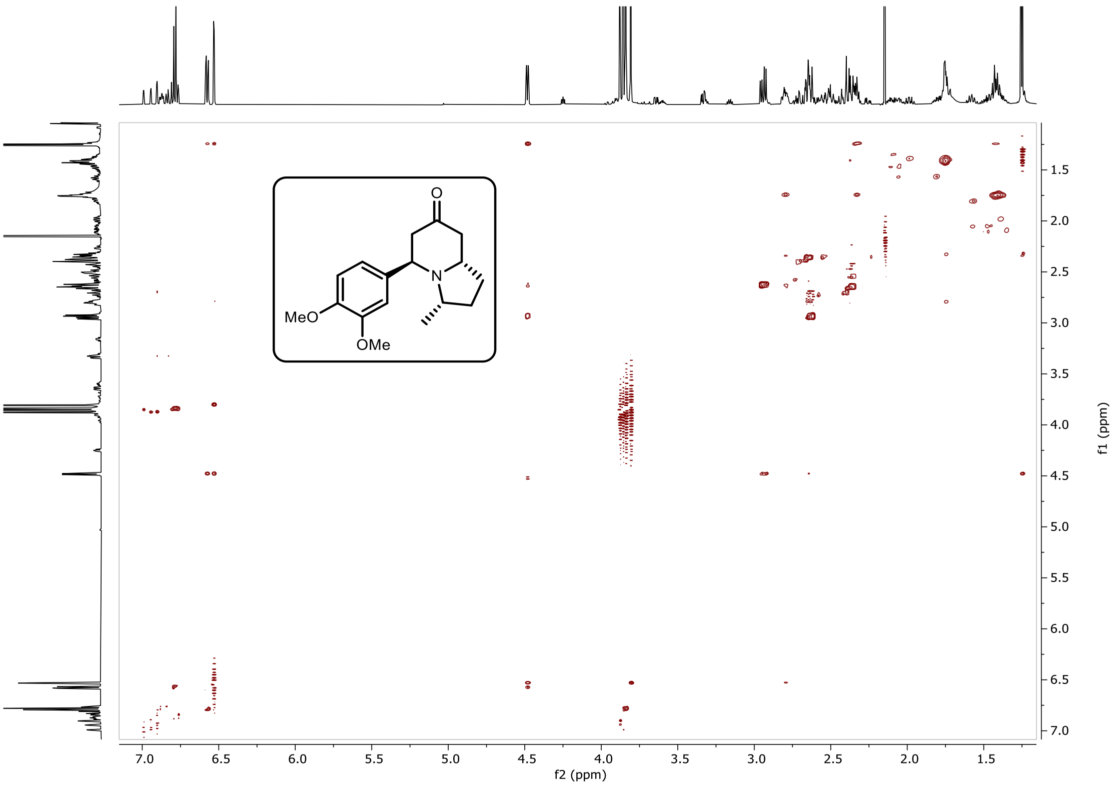

A142: *NOESY (CDCl$_3$) of compound 3S,5R,8aS-**4f** (enriched).*

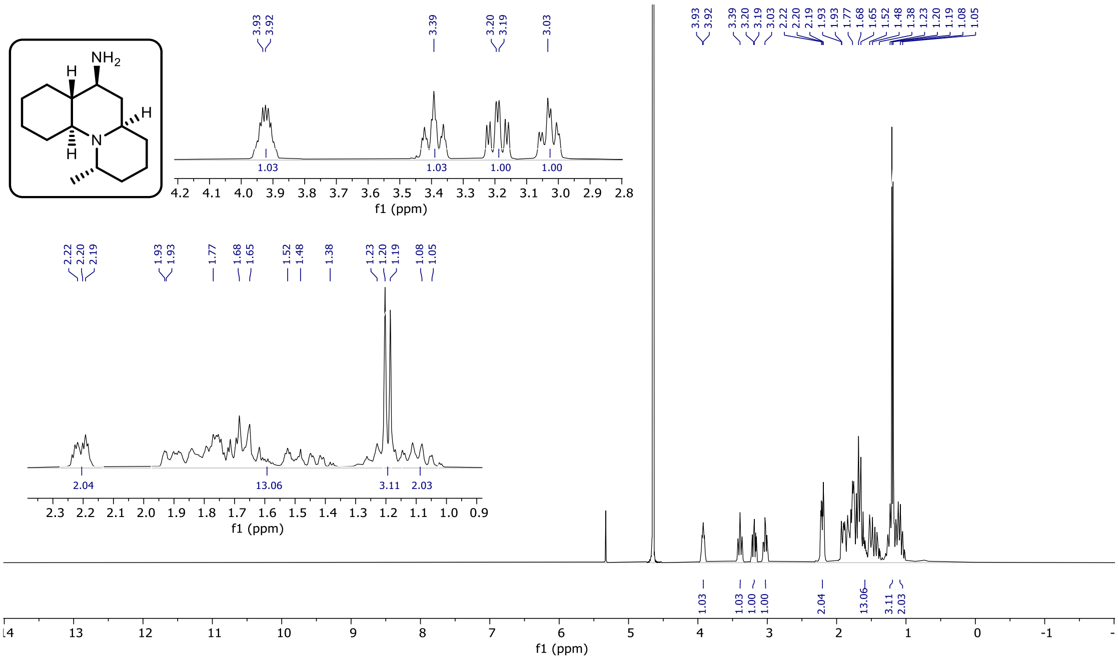

A143: *^{1}H NMR (D_2O) of compound* ***5a****.*

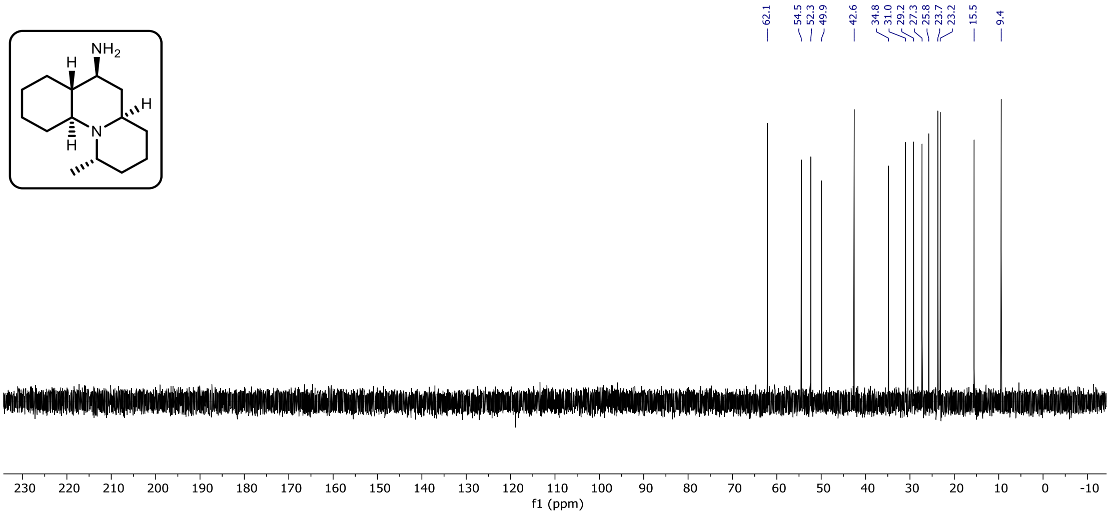

A144: *^{13}C NMR (D_2O) of compound* ***5a****.*

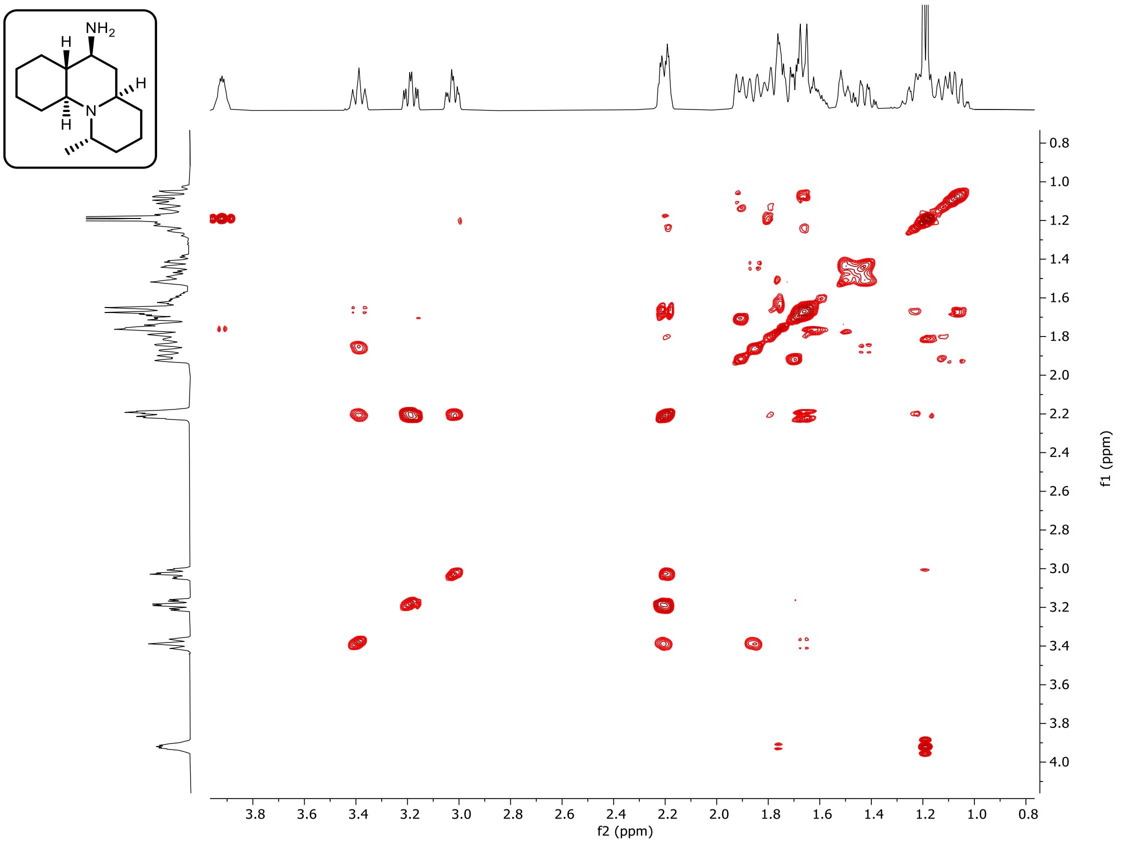

A145: *COSY NMR (D_2O) of compound* ***5a***.

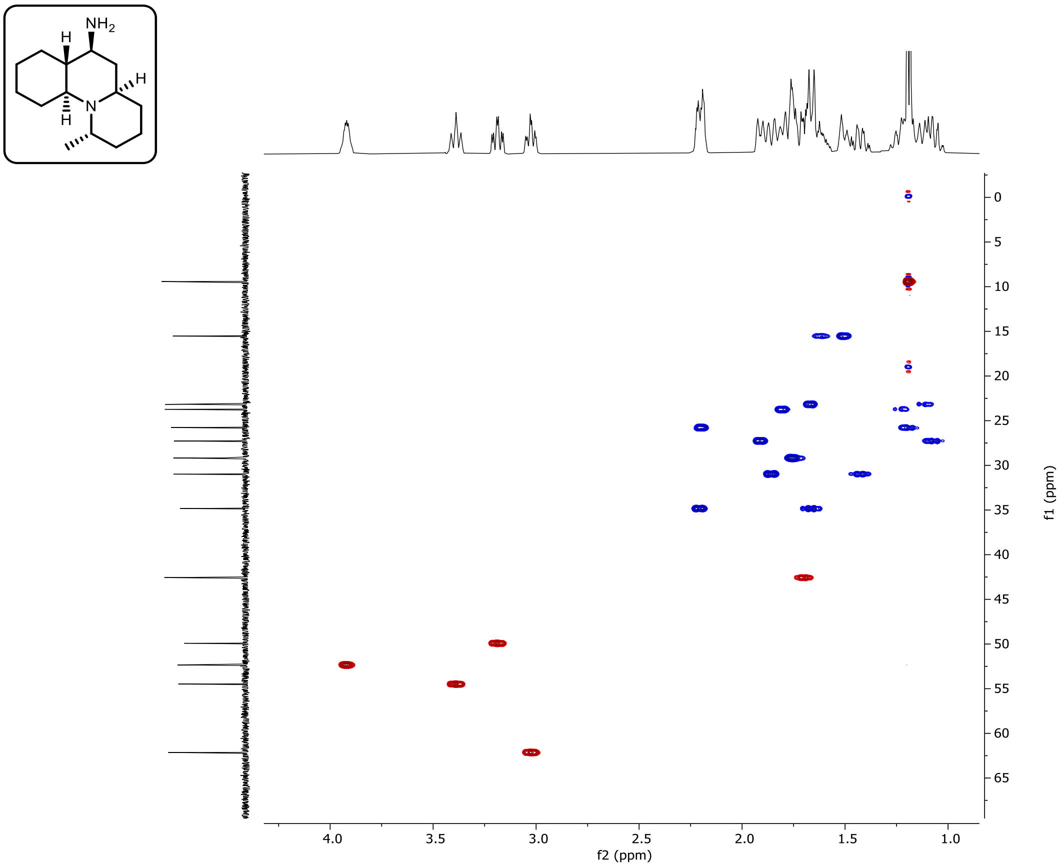

A146: *HSQC (D_2O) of compound* ***5a***.

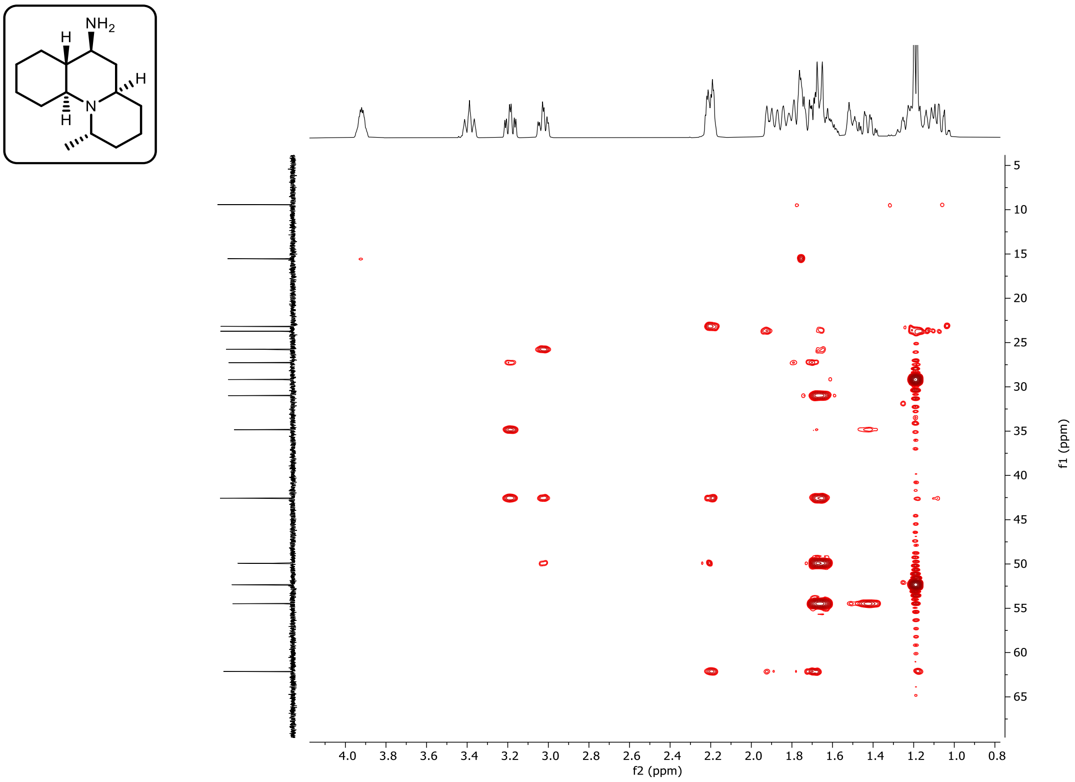

A147: *HMBC (D_2O) of compound* ***5a***.

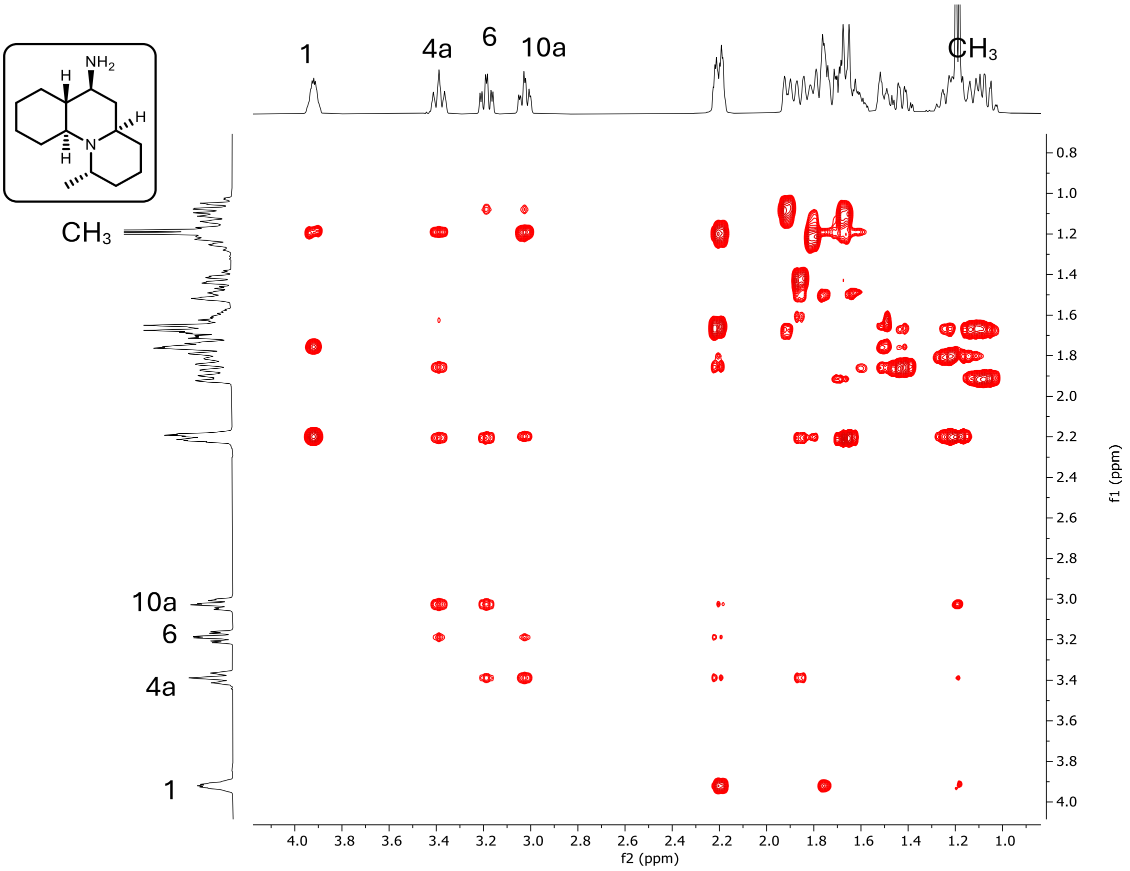

A148: *NOESY (D_2O) of compound* ***5a***.

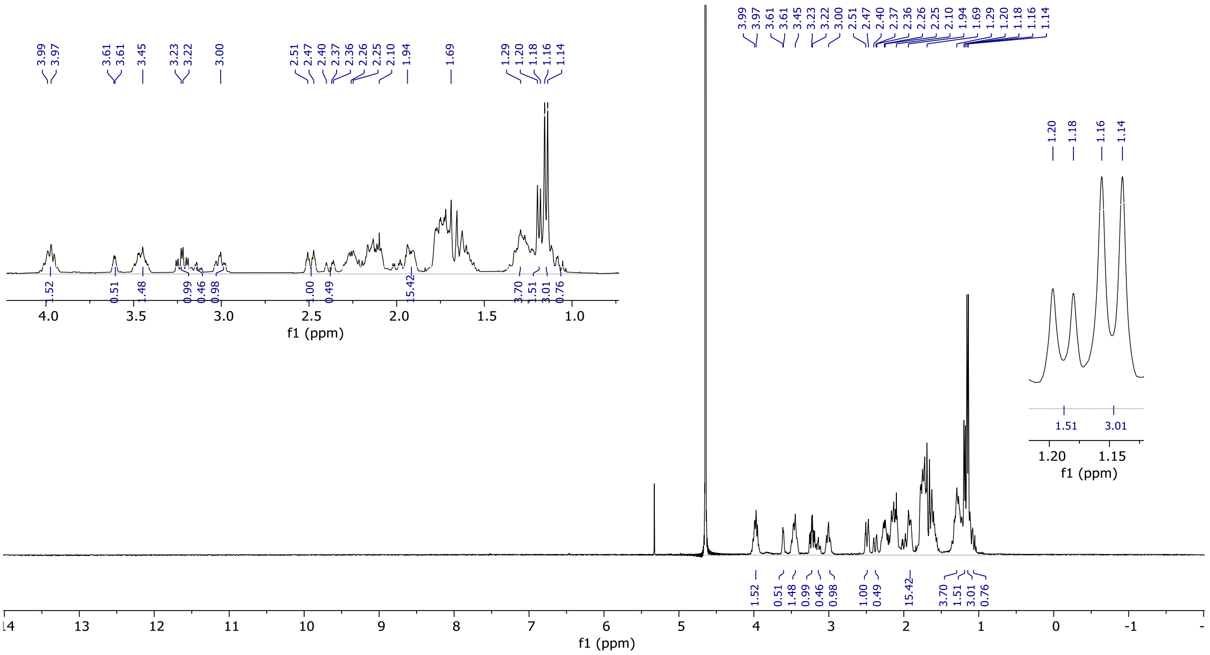

A149: ^{1}H NMR (D_2O) of compound (5*S*/5*R*)-**5c** in a 2:1 ratio from the transaminase catalysed biotransformation of **4c**.

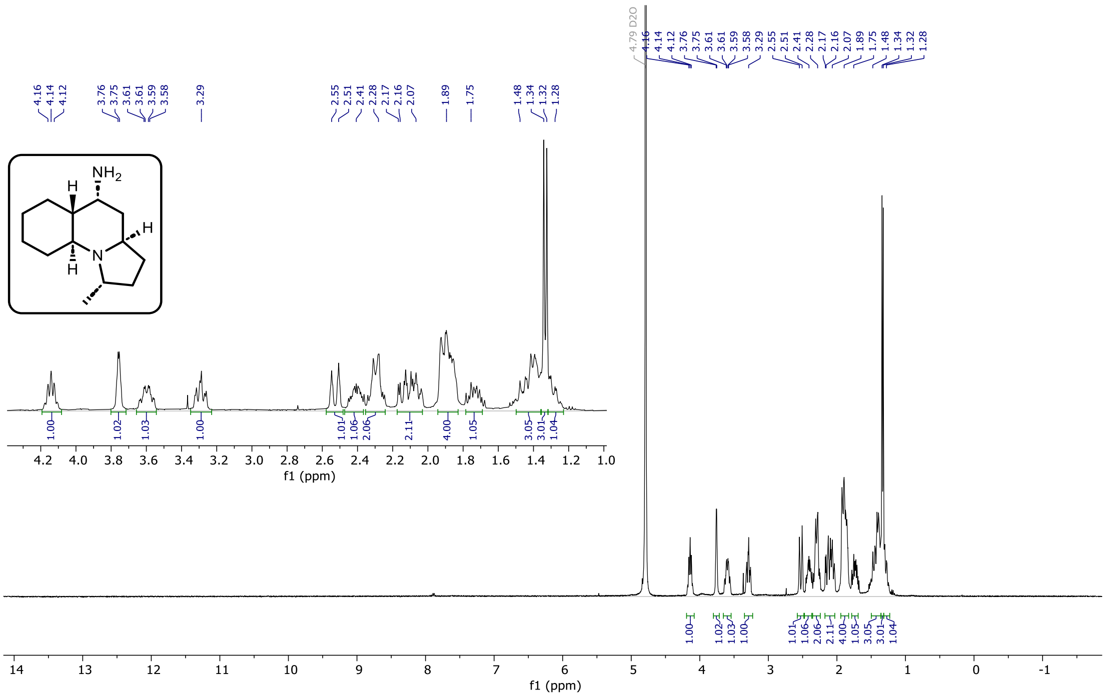

A150: *^{1}H NMR (D_2O) of compound 5R-**5c**.*

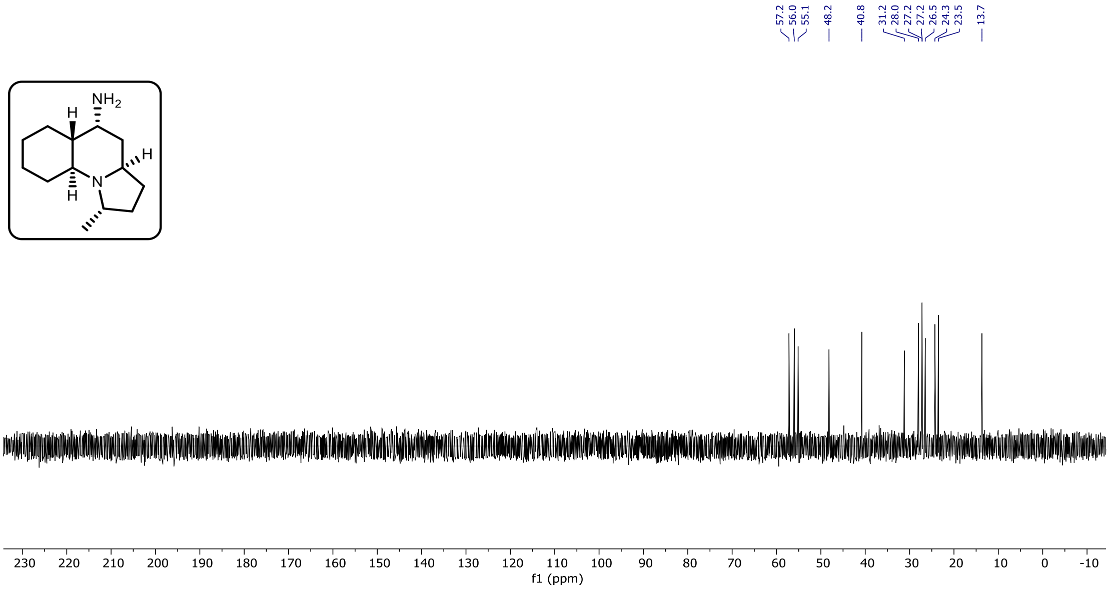

A151: *^{13}C NMR (D_2O) of compound 5R-**5c**.*

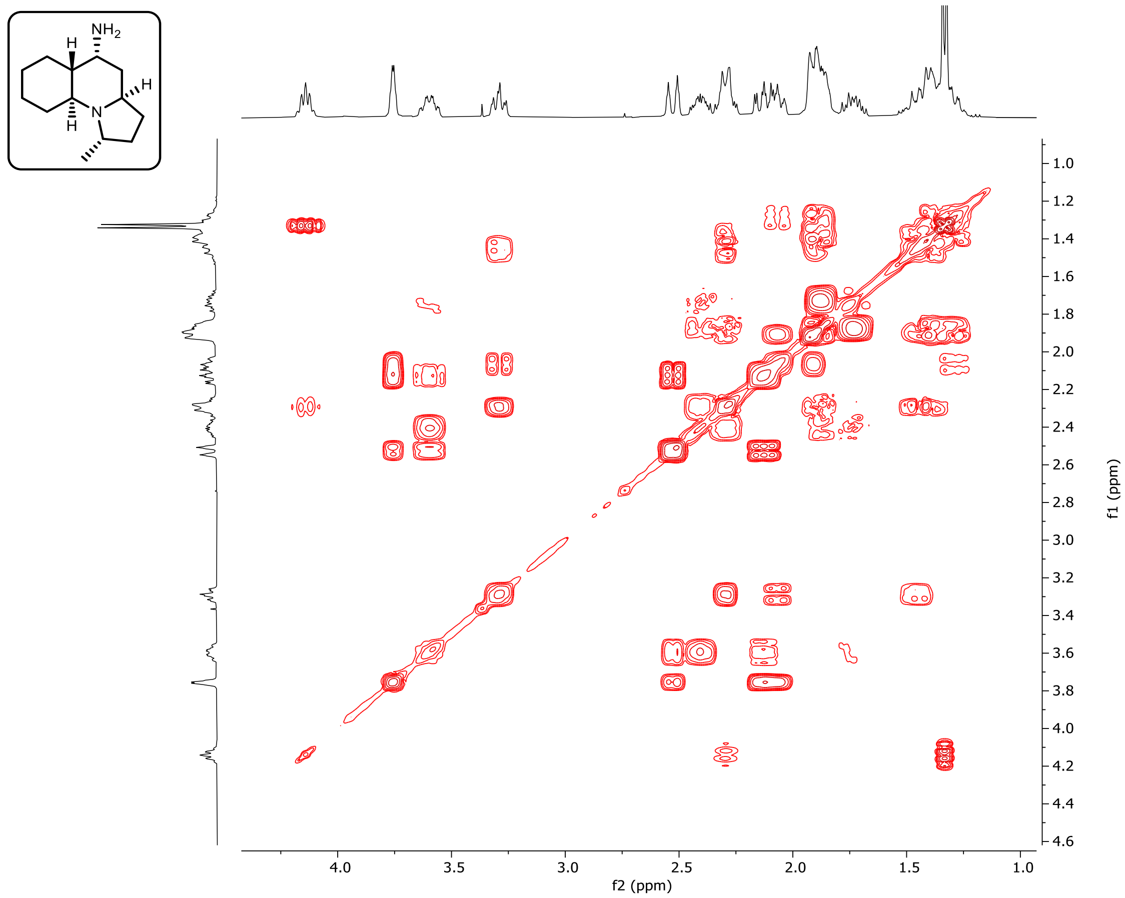

A152: *COSY NMR (D_2O) of compound 5R-**5c**.*

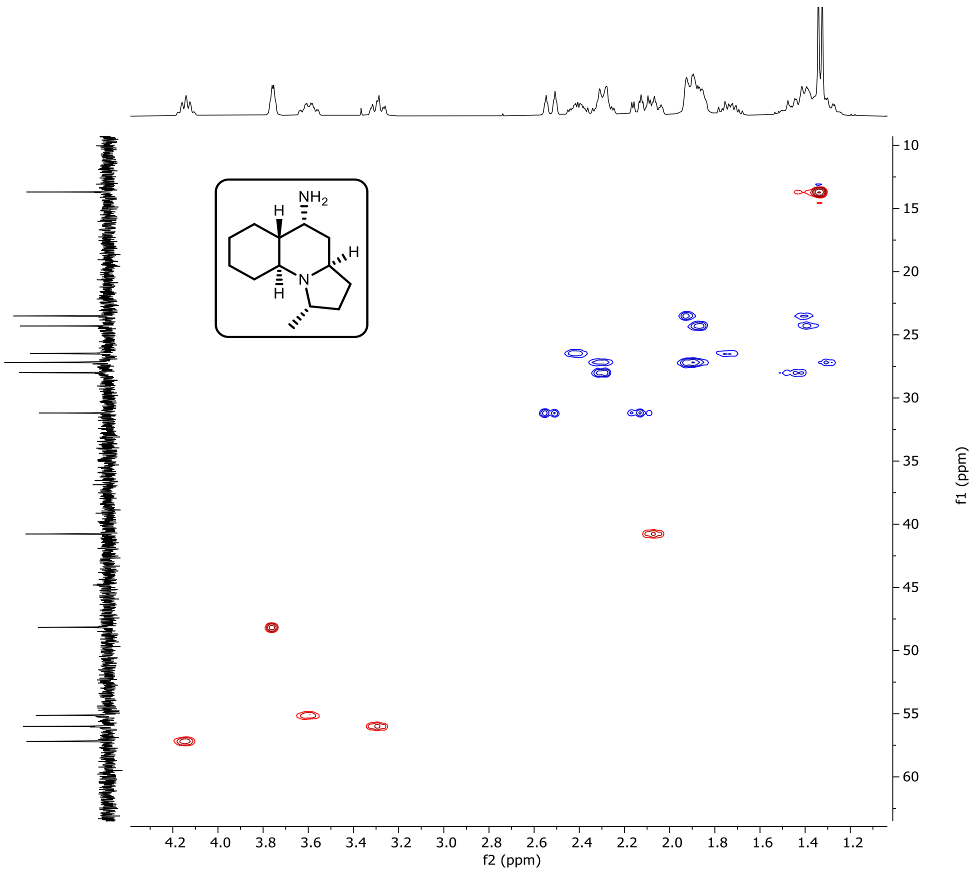

A153: *HSQC (D_2O) of compound 5R-**5c***

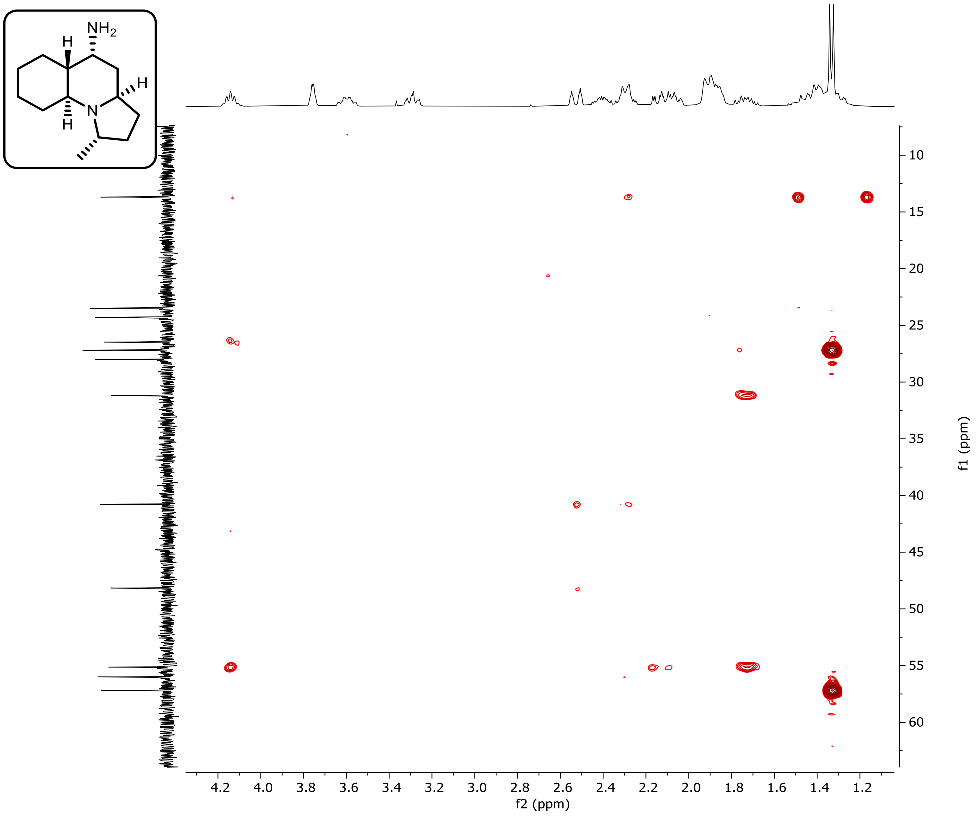

A154: *HMBC (D_2O) of compound 5R-**5c**.*

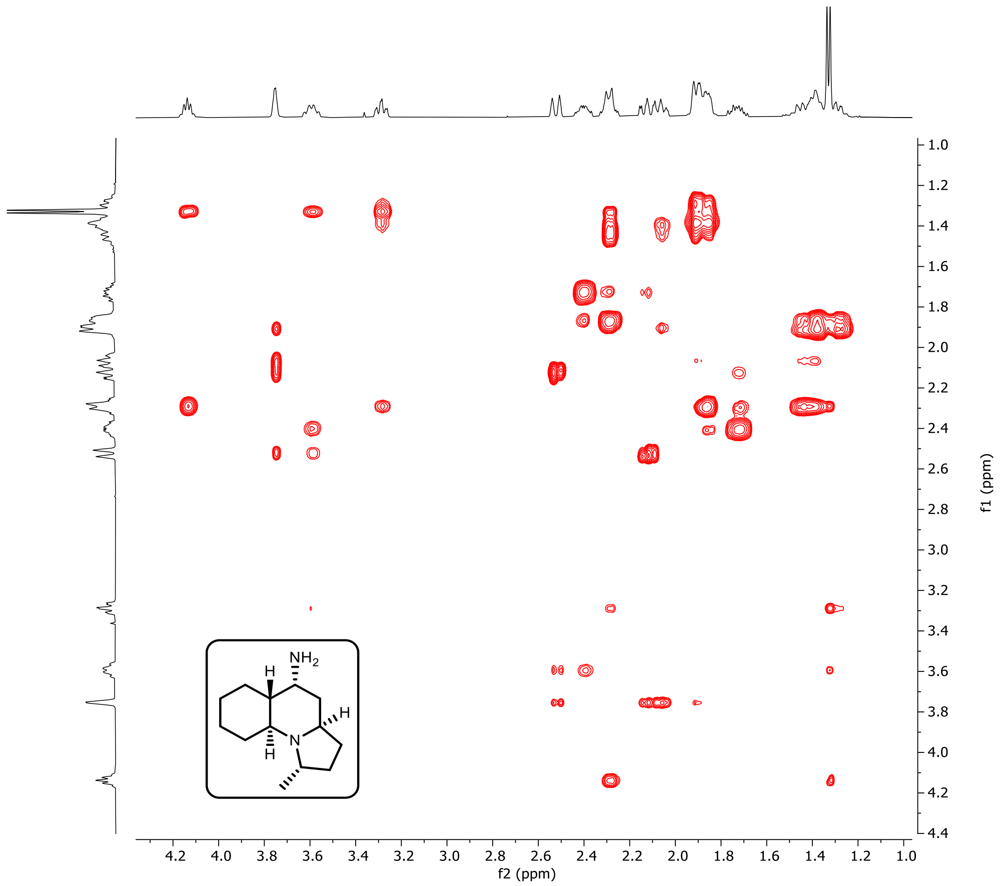

***A155:** NOESY (D_2O) of compound 5R-**5c**.*

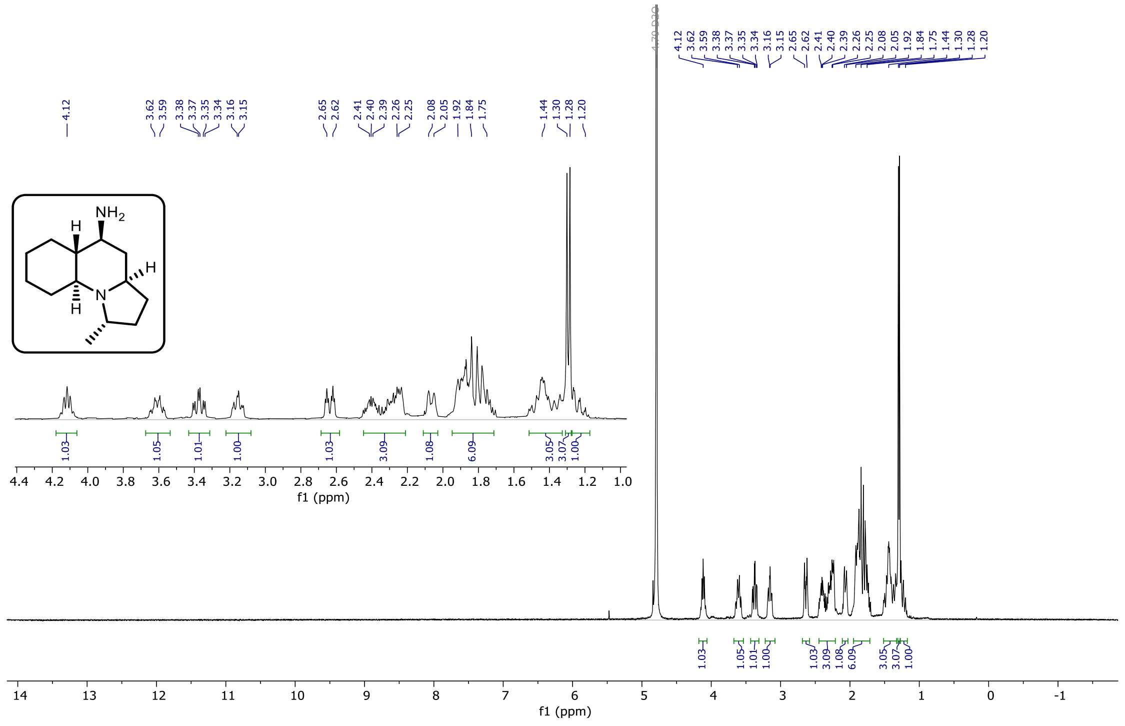

***A156:** ^1H NMR (D_2O) of compound 5S-**5c**.*

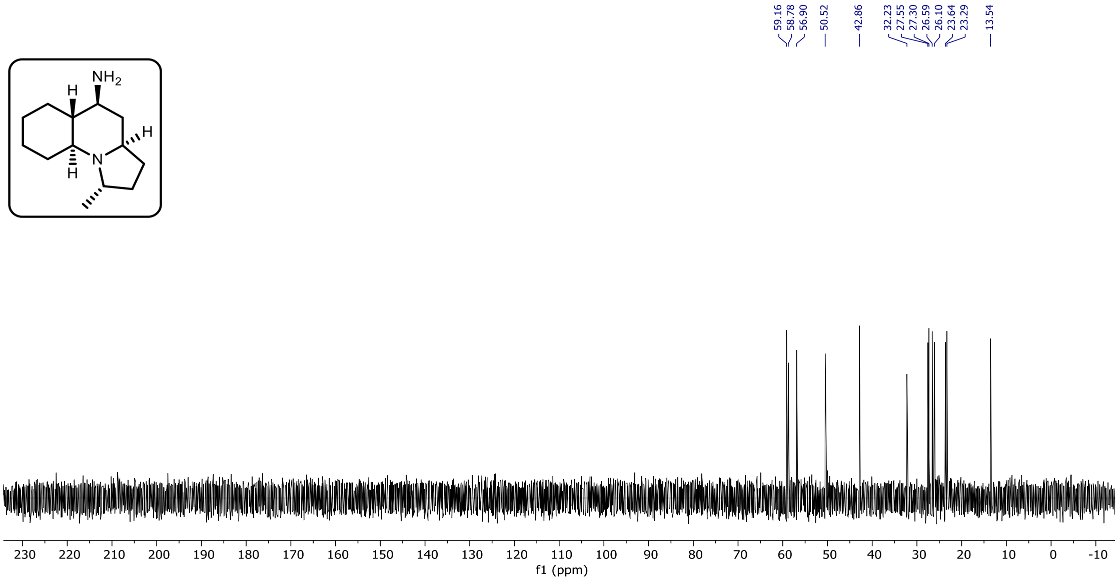

A157: *^{13}C NMR (D_2O) of compound 5S-**5c**.*

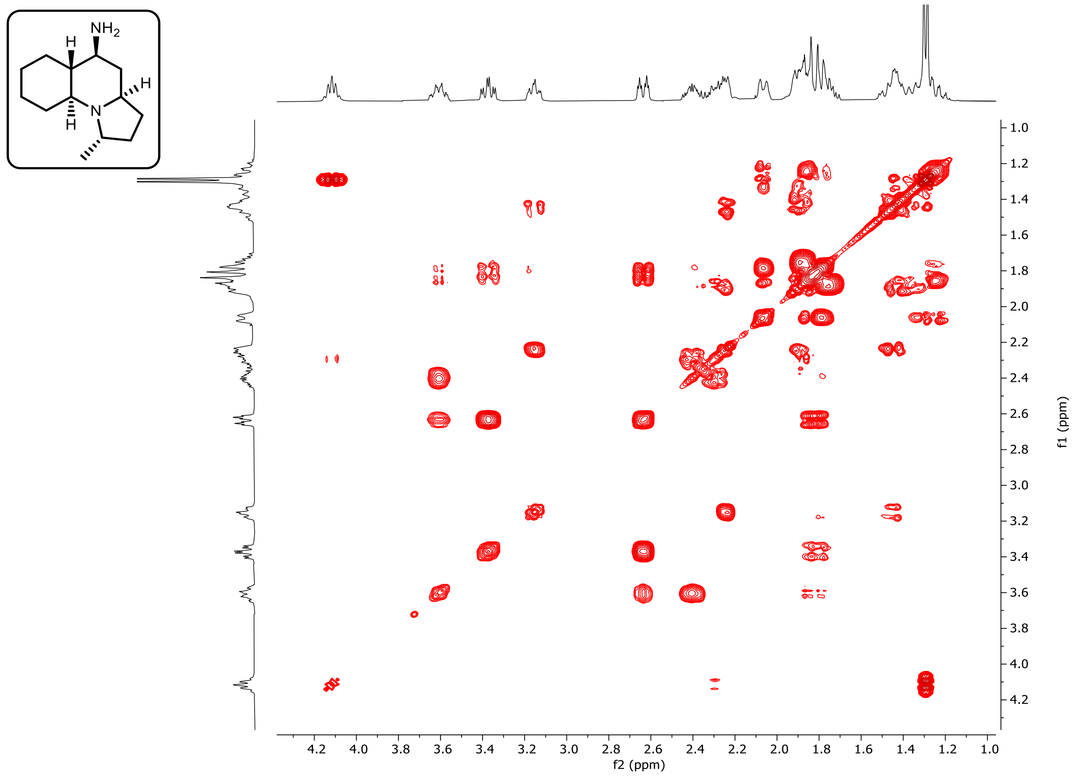

A158: *COSY NMR (D_2O) of compound 5S-**5c**.*

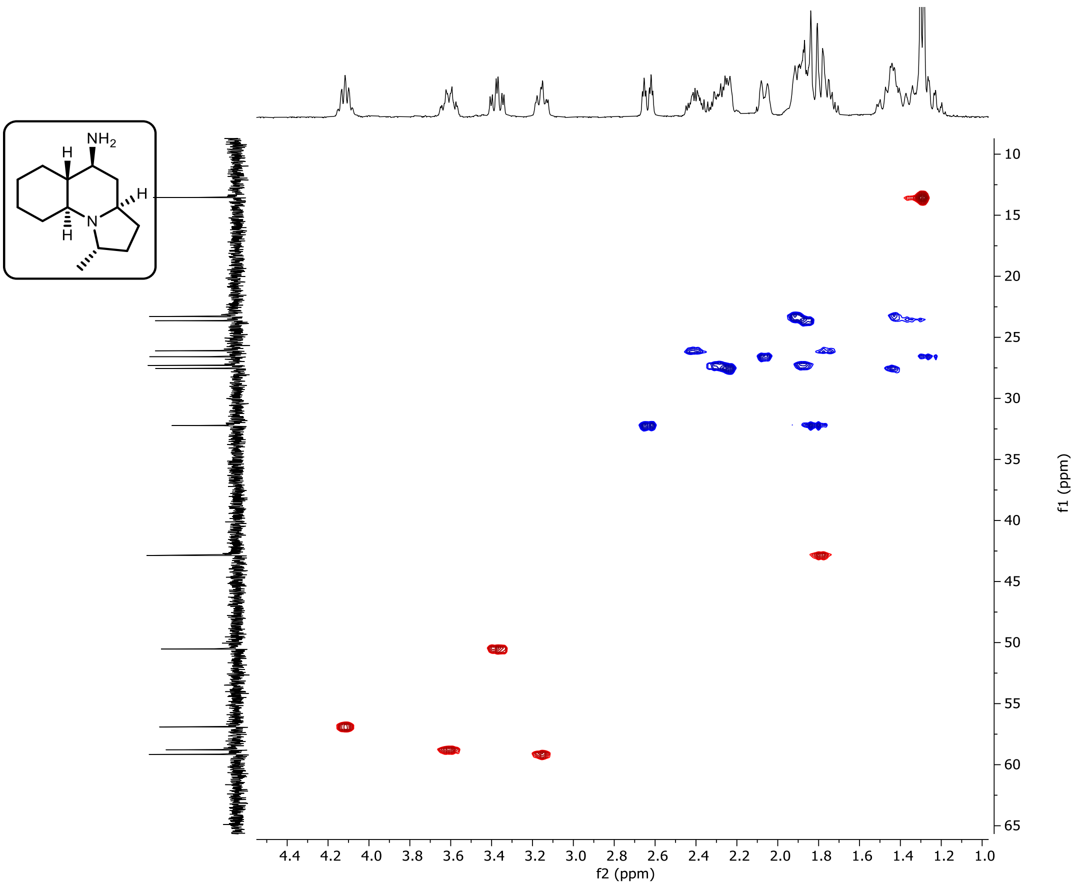

***A159:** HSQC (D_2O) of compound 5S-**5c**.*

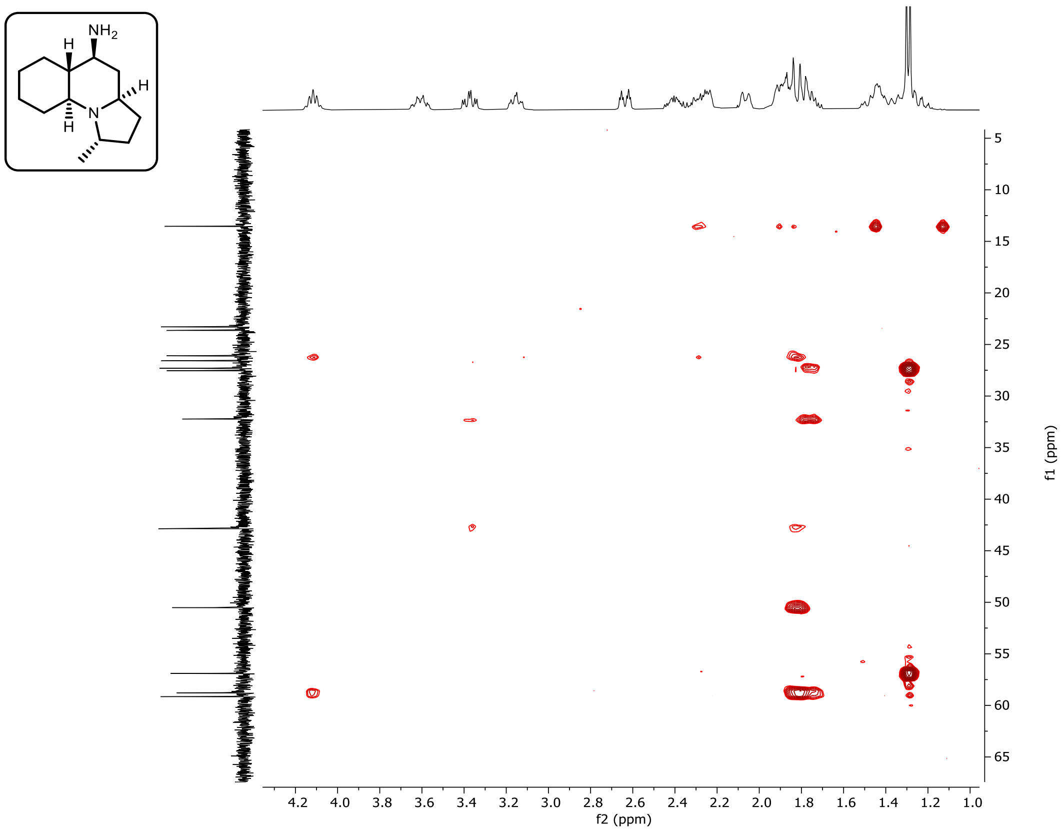

***A160:** HMBC (D_2O) of compound 5S-**5c**.*

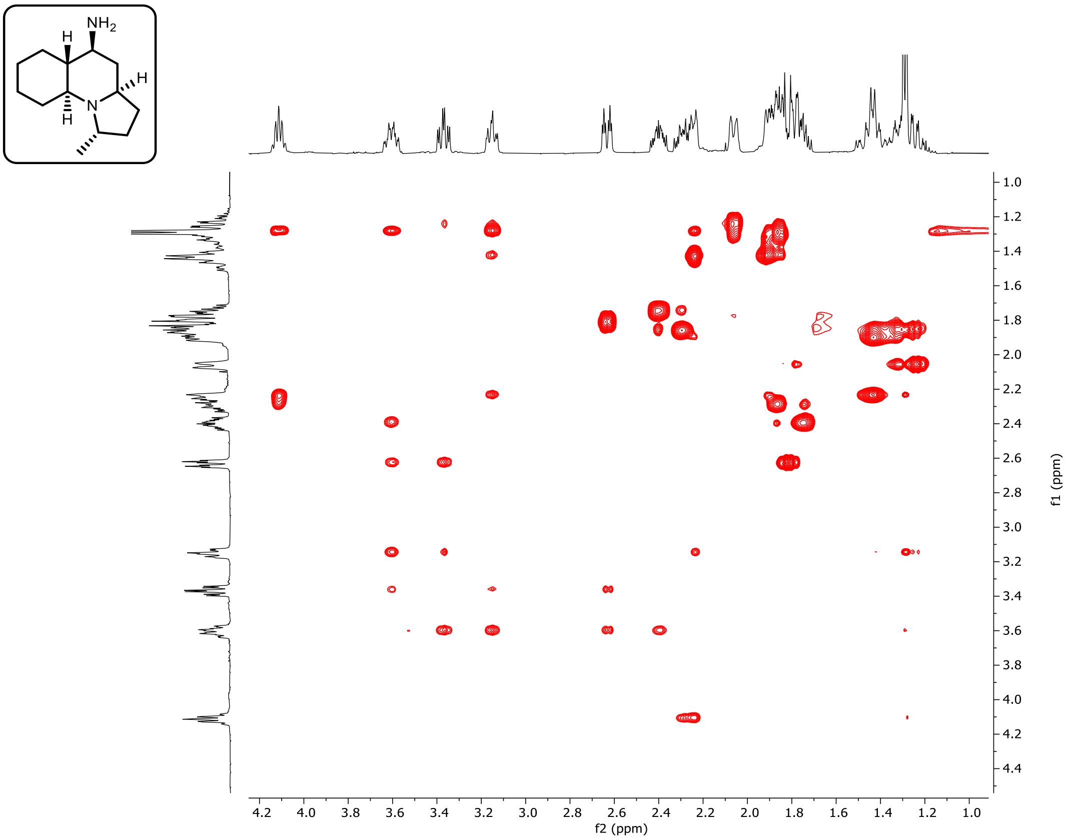

A161: *NOESY (D_2O) of compound 5S-**5c**.*

8. Chiral GC-MS Chromatograms

e.e. determination of compounds **3a** was carried out in an Agilent 5977B MSD combined to an Agilent 7890B GC system equipped with a Cyclosil-β chiral column (30 m long, 0.25 mm diam. narrowbore, 0.25 μm film).

Isothermic method, 130 °C, 10 psi. Helium used as carrier gas.

Cyclosil-β, 30 m long, 0.25 mm diam. narrowbore, 0.25 μm film

Isothermic 120 °C, 10 psi

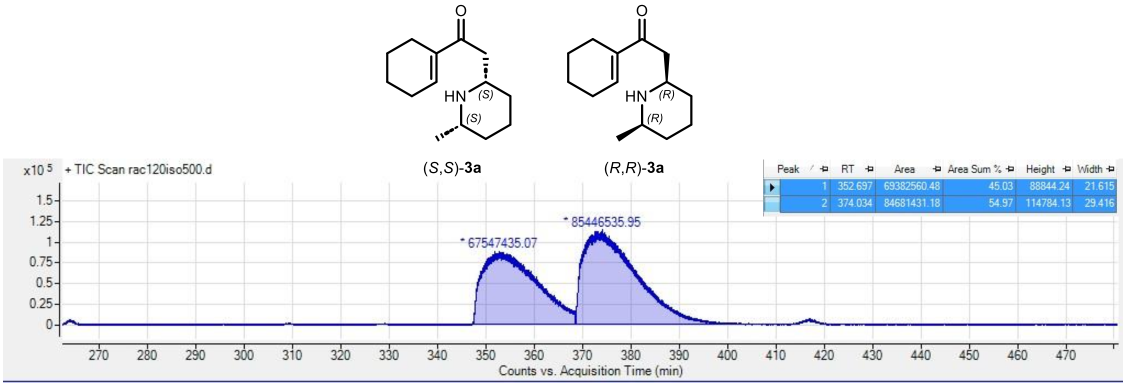

Figure S5: *Racemic sample – prepared by combined equal amounts of samples* (*S*,*S*)-***3a*** *and* (*R*,*R*)-***3a***.

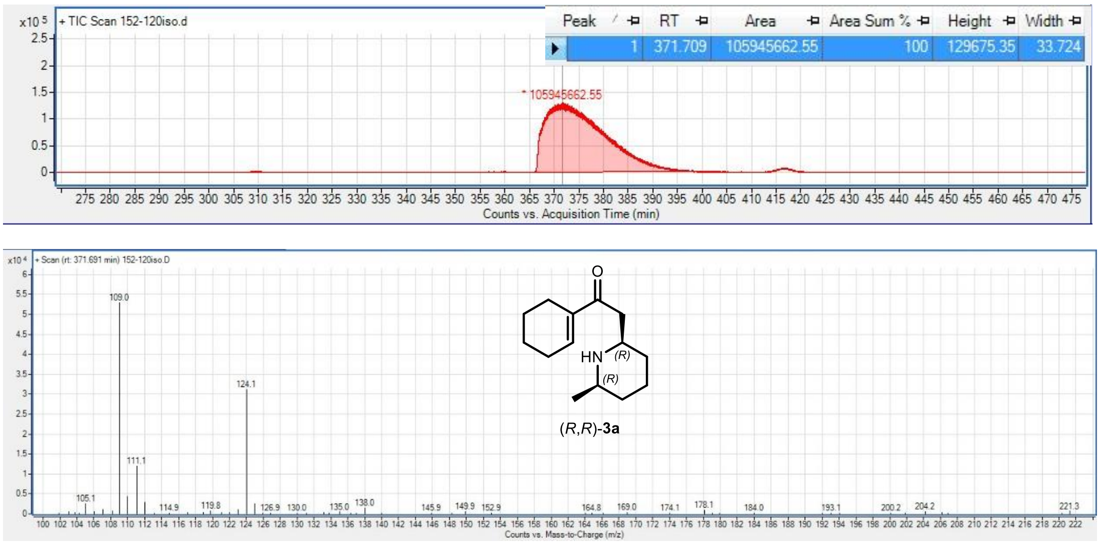

Figure S6: *GC-MS chromatograms of* (*R*,*R*)-***3a***.

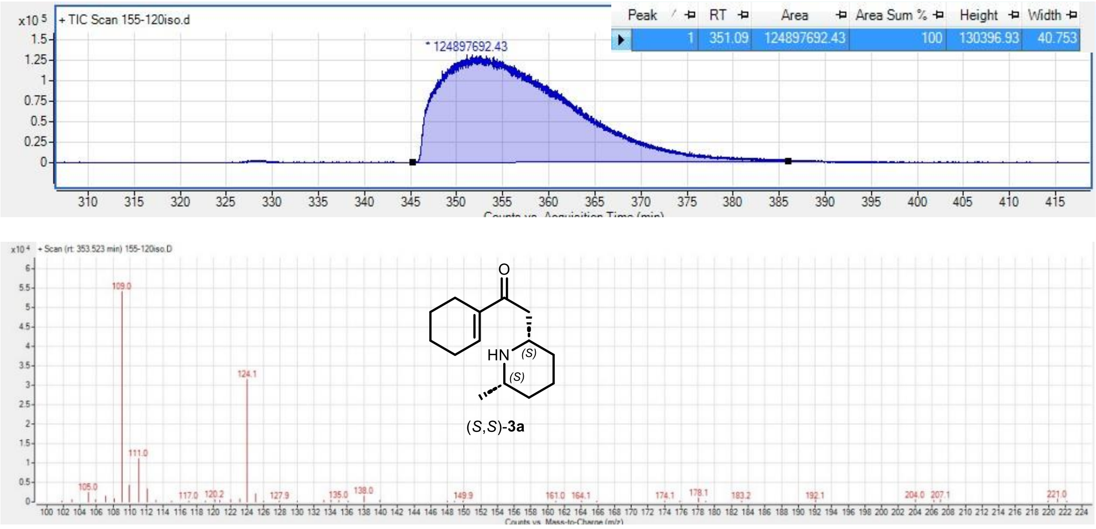

Figure S7: *GC-MS chromatograms of (S,S)-**3a**.*

9. Chiral HPLC Chromatograms

Chiral HPLC analysis of compound **3e** was performed using an Agilent Technologies 1200 series equipped with a binary pump (G1312A) and automated liquid sampler (G1329A); OJ-H, 1.0 mL/min, 95:5 Heptane/isopropanol.

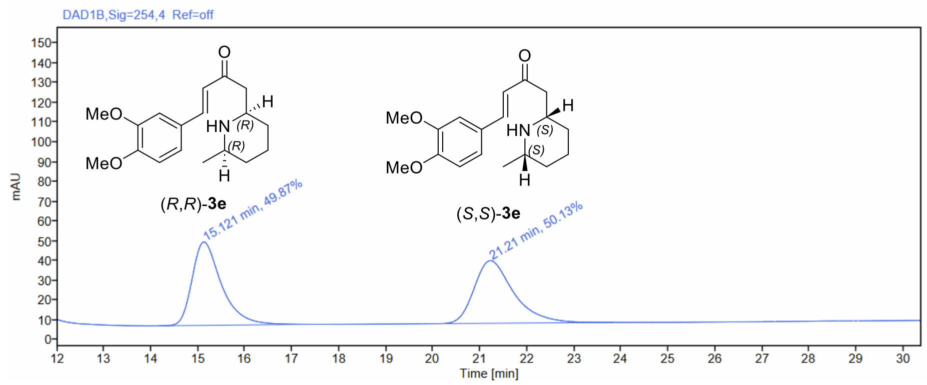

Peak Results (Area Percent at least 1%)

RT (min)	Signal Description	Width (min)	Area	Height	Area%
15.121	DAD1B,Sig=254,4 Ref=off	3.097	1840.8	42.2	49.87
21.210	DAD1B,Sig=254,4 Ref=off	3.708	1850.2	31.7	50.13
		**Sum DAD1B**	3691.0		

Figure S8: *Racemic sample – prepared by combined equal amounts of samples (S,S)-**3e** and (R,R)-**3e**.*

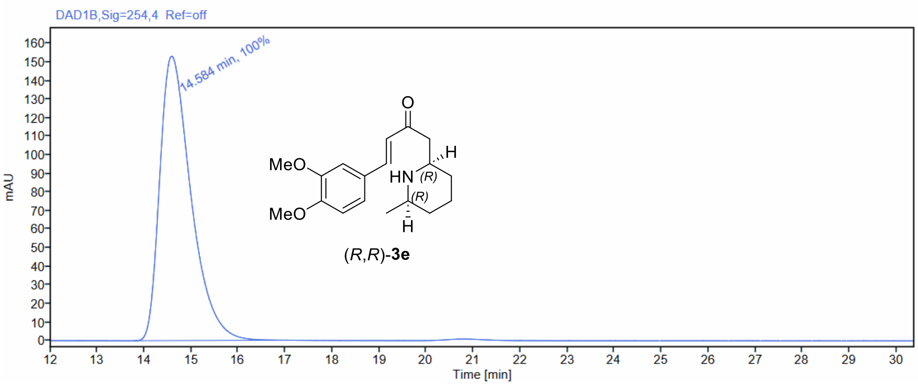

Figure S9:** HPLC chromatograms of* (*R*,*R*)-3e***.

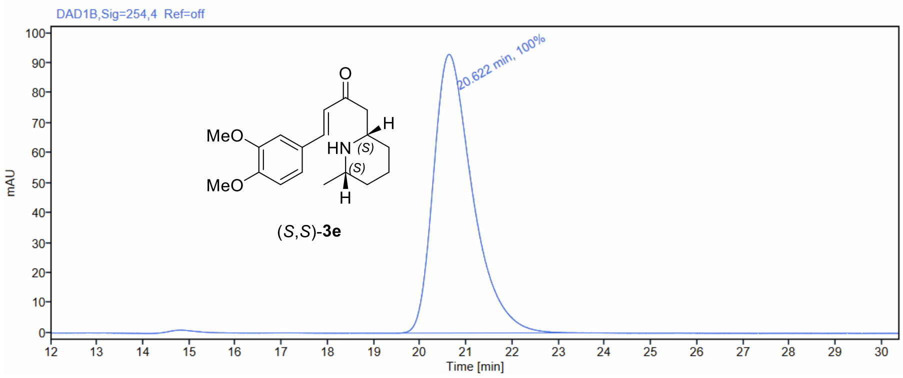

Figure S10:** HPLC chromatograms of* (*S*,*S*)-3e***.

10. Energy calculations

All structures were constructed in ChemDraw and geometry-optimized (HF/6-31G*) in Gaussian16.[13] Hydrogen-hydrogen distances were calculated from the optimized structures using a TCL script (available upon request).

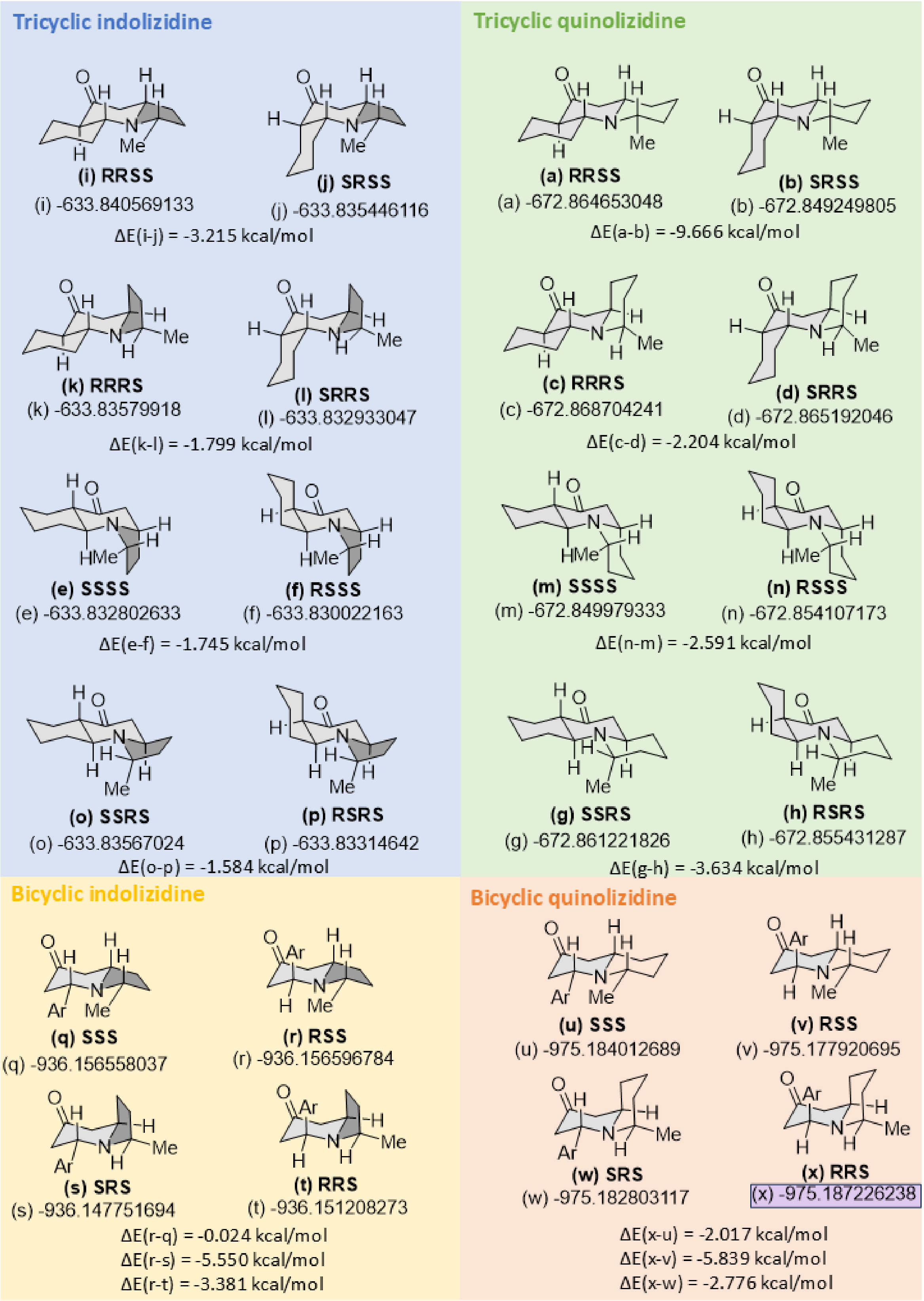

Figure S11. *Energy calculation for the quinolizidines **4a** and **4e**, and indolizidines **4c** and **4f**. Absolute energies are given in Hartree. ΔE is given in kcal/mol. 1 Hartree = 627.503 kcal/mol*

11. H-H proximity calculation

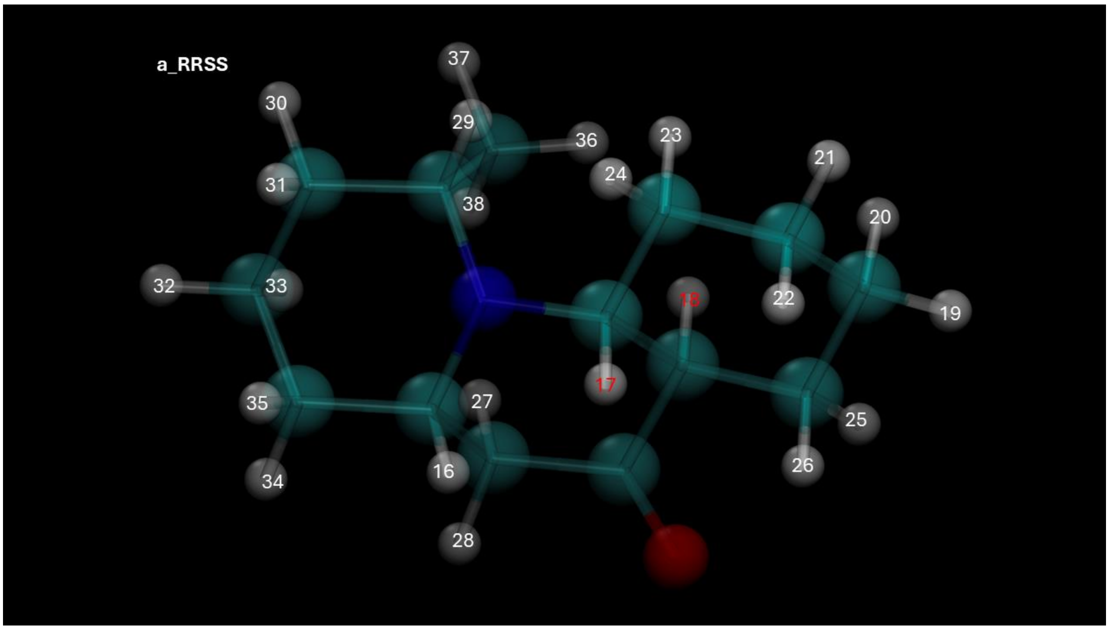

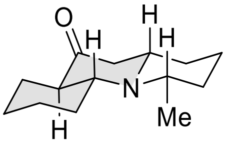

(a) RRSS

```
#H-H distances (a)
#H16
H16 H17 2.204862117767334
H16 H26 4.044322490692139
H16 H27 3.0331459045410156
H16 H28 2.447922468185425
H16 H34 2.528081178665161
H16 H35 2.2844884395599365
H16 H32 4.220254421234131
H16 H33 3.8774986267089844
#H17
H17 H18 3.0224008560180664
H17 H21 3.6803741455078125
H17 H22 2.506410837173462
H17 H23 3.0262832641601562
H17 H24 2.442352771759033
H17 H25 3.7103166580200195
H17 H26 2.544203519821167
H17 H27 3.965827703475952
H17 H28 3.961921453475952
#H18
H18 H17 3.0224008560180664
H18 H19 3.772749662399292
H18 H20 2.624556541442871
H18 H21 4.138729572296143
H18 H22 4.004448413848877
H18 H23 2.616095781326294
H18 H24 3.7641355991363525
H18 H25 2.46162748336792
H18 H26 3.0372633934020996
```

```
#H29
H29 H16 4.024765491485596
H29 H27 4.190476417541504
H29 H28 5.272850036621094
H29 H30 2.4830644130706787
H29 H31 2.34018611907959
H29 H32 4.245436191558838
H29 H33 3.8170976638793945
H29 H36 2.465608596801758
H29 H37 2.4173264503479004
H29 H38 3.0478222370147705
#H27
H27 H17 3.965827703475952
H27 H18 2.8265371322631836
H27 H28 1.753517985343933
H27 H32 4.072012901306152
H27 H33 2.593975305557251
H27 H34 2.981219530105591
H27 H35 3.885967969894409
H27 H36 3.247580051422119
H27 H37 3.867764949798584
H27 H38 2.195845603942871
#H28
H28 H17 3.961921453475952
H28 H18 3.8480024337768555
H28 H27 1.753517985343933
H28 H32 4.5517706871032715
H28 H33 3.5484085083007812
H28 H34 2.4821202754974365
H28 H35 3.6593267917633057
H28 H36 4.79719877243042
H28 H37 5.478423118591309
H28 H38 3.8953347206115723
#H36
H36 H16 4.6556196212768555
H36 H17 4.106292247772217
```

H36 H23 2.3945274353027344
H36 H24 3.7743353843688965
H36 H29 2.465608596801758
H36 H30 3.649007797241211
H36 H31 4.2798991203308105
H36 H37 1.7427763938903809
H36 H38 1.7505019903182983
#H37
H37 H16 5.294671535491943
H37 H17 5.203505039215088
H37 H23 3.5448663234710693
H37 H24 4.557003021240234
H37 H29 2.4173264503479004
H37 H30 2.3889260292053223
H37 H31 3.619703769683838
H37 H36 1.7427763938903809
H37 H38 1.7480370998382568
#H38
H38 H16 4.296773910522461
H38 H17 4.61707067489624
H38 H23 3.87646484375
H38 H24 4.848628520965576
H38 H29 3.047822370147705
H38 H30 3.000932455062866
H38 H31 3.86969256401062
H38 H37 1.7480370998382568
H38 H36 1.750501990318298

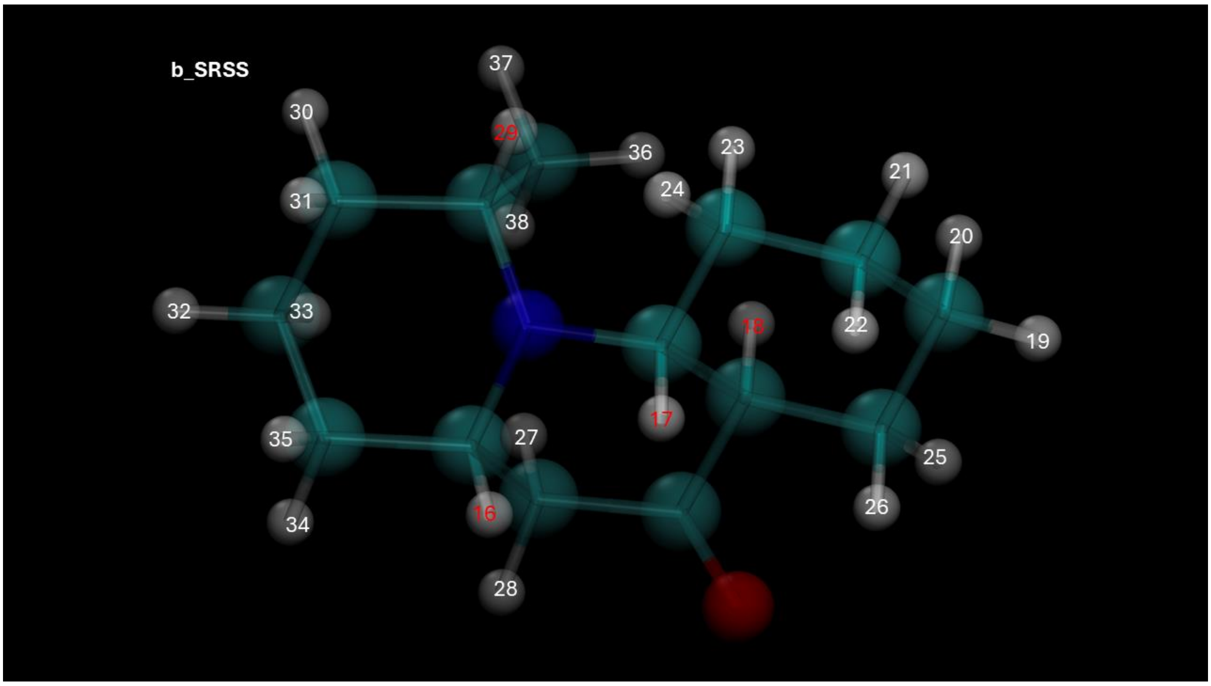

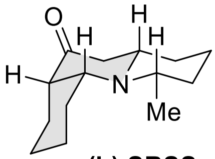

(b) SRSS

#H-H distances (b)
#H16
H16 H17 2.113414764404297
H16 H26 5.037173748016357
H16 H27 3.0289065837860107
H16 H28 2.465876579284668
H16 H34 2.5480399131774902
H16 H35 2.24284291267395
H16 H32 4.193188667297363
H16 H33 3.915217161178589
#H17
H17 H18 2.2464566230773926
H17 H21 3.9381511211395264
H17 H22 4.197274208068848
H17 H23 2.52380108833313
H17 H24 2.222257614135742
H17 H25 3.9684197902679443
H17 H26 4.195682525634766
H17 H27 4.00944185256958
H17 H28 4.00829553604126
#H18
H18 H17 2.2464566230773926
H18 H19 2.6407618522644043
H18 H20 3.775934934616089
H18 H21 4.080803394317627
H18 H22 4.208032131195068
H18 H23 3.859882354736328
H18 H24 2.761197090148926
H18 H25 3.0253798961639404
H18 H26 2.359145164489746
#H29
H29 H16 4.0272393226623535

H29 H27 4.150750160217285
H29 H28 5.259464740753174
H29 H30 2.4790472984313965
H29 H31 2.3724746704101562
H29 H32 4.255843639373779
H29 H33 3.8041200637817383
H29 H36 2.485165596008301
H29 H37 2.4150872230529785
H29 H38 3.049739122390747
#H27
H27 H17 4.00944185256958
H27 H18 3.999547004699707
H27 H28 1.749277114868164
H27 H32 4.225047588348389
H27 H33 2.7946739196777344
H27 H34 3.00775408744812
H27 H35 3.9198715686798096
H27 H36 3.081749200820923
H27 H37 3.8539116382598877
H27 H38 2.2033491134643555
#H28
H28 H17 4.00829553604126
H28 H18 4.062001705169678
H28 H27 1.749277114868164
H28 H32 4.637738227844238
H28 H33 3.6740617752075195
H28 H34 2.4986963272094727
H28 H35 3.648306369781494
H28 H36 4.689176082611084
H28 H37 5.452237129211426
H28 H38 3.862734794616699
#H36
H36 H16 4.646442890167236
H36 H17 4.317303657531738
H36 H23 3.8429312705993652
H36 H24 4.501542568206787
H36 H29 2.485165596008301
H36 H30 3.65400767326355
H36 H31 4.287403583526611
H36 H37 1.7415080070495605
H36 H38 1.7453685998916626
#H37
H37 H16 5.281245231628418
H37 H17 5.290686130523682
H37 H23 4.462106704711914
H37 H24 5.606960773468018
H37 H29 2.4150872230529785
H37 H30 2.3953540325164795
H37 H31 3.630852699279785
H37 H36 1.7415080070495605
H37 H38 1.7465217113494873
#H38
H38 H16 4.2786030769348145
H38 H17 4.762928485870361
H38 H23 4.830212116241455
H38 H24 5.630415439605713
H38 H29 3.049739122390747
H38 H30 2.9887568950653076
H38 H31 3.8330514430999756
H38 H37 1.7465217113494873
H38 H36 1.7453685998916626

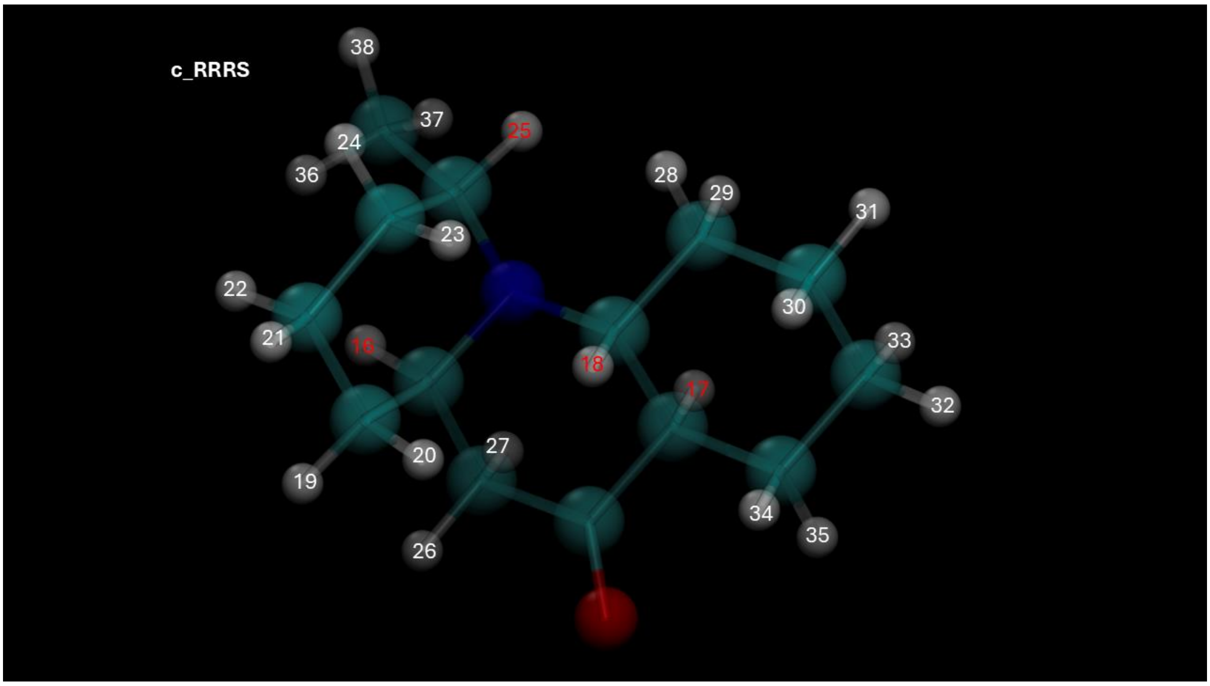

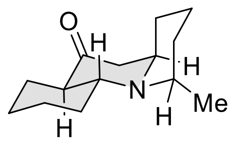

(c) RRRS

```
#H-H distances (c)
#H16
H16 H17 3.9094929695129395
H16 H26 2.520646333694458
H16 H27 2.329335927963257
H16 H28 4.80732536315918
H16 H34 5.594220161437988
H16 H35 5.853340148925781
H16 H32 7.168247699737549
H16 H33 6.278567790985107
#H17
H17 H18 3.0164482593536377
H17 H21 6.225434303283691
H17 H22 5.910215854644775
H17 H23 5.004868984222412
H17 H24 5.994495868682861
H17 H25 4.118030071258545
H17 H26 3.719942808151245
H17 H27 2.5548183917999268
H17 H28 3.719787359237671
#H18
H18 H17 3.0164482593536377
H18 H19 3.808887004852295
H18 H20 2.281122922897339
H18 H21 3.91585111618042
H18 H22 4.3472208976745605
H18 H23 2.433688163757324
H18 H24 3.9584569931030273
H18 H25 3.0269076824188232
H18 H26 4.086523056030273
#H25
H25 H18 3.0269076824188232
H25 H23 2.384373903274536
H25 H24 2.500727891921997
H25 H24 1.9309768676757812
H25 H24 2.5847253799438477
H25 H36 3.0264182090759277
H25 H37 2.4554905891418457
H25 H38 2.34881854057312
#H29
H29 H16 4.618402004241943
H29 H27 4.436560153961182
H29 H28 1.745507001876831
H29 H30 3.052079677581787
H29 H31 2.464594602584839
H29 H32 3.8306808471679688
H29 H33 2.6958446502685547
H29 H36 4.932004451751709
H29 H37 3.769806146621704
H29 H38 4.594394683837891
#H27
H27 H17 2.5548183917999268
H27 H18 4.002045631408691
H27 H28 5.270539283752441
H27 H32 6.165356636047363
H27 H33 5.138639450073242
H27 H34 4.715691089630127
H27 H35 4.326382160186768
H27 H36 4.201470375061035
H27 H37 4.061139106750488
H27 H38 5.405788421630859
#H28
H28 H17 3.719787359237671
H28 H18 2.461106777191162
H28 H27 5.270539283752441
H28 H32 4.265326023101807
H28 H33 3.79636287689209
H28 H34 4.143850326538086
H28 H35 4.946738243103027
H28 H36 4.846646308898926
H28 H37 4.116210460662842
H28 H38 4.261039733886719
#H36
H36 H16 2.130427360534668
H36 H17 5.236322402954102
H36 H23 3.8594000339508057
H36 H24 2.9947166442871094
H36 H25 3.0264182090759277
H36 H29 4.932004451751709
H36 H30 6.818620204925537
H36 H31 7.085949420928955
H36 H37 1.754571557044983
H36 H38 1.7446495294570923
#H37
H37 H16 2.8237149715423584
H37 H17 4.4578328132629395
H37 H23 4.285791397094727
H37 H24 3.7445871829986572
H37 H25 2.4554905891418457
H37 H29 3.769806146621704
H37 H30 6.2457356452941895
H37 H31 6.105340957641602
H37 H36 1.754571557044983
H37 H38 1.758793592453003
#H38
H38 H16 3.599996328353882
H38 H17 5.726466178894043
H38 H23 3.7253620624542236
H38 H24 2.573881149291992
H38 H25 2.34881854057312
H38 H28 4.261039733886719
H38 H29 4.594394683837891
H38 H30 6.552095890045166
H38 H31 6.563816547393799
H38 H37 1.758793592453003
H38 H36 1.7446495294570923
```

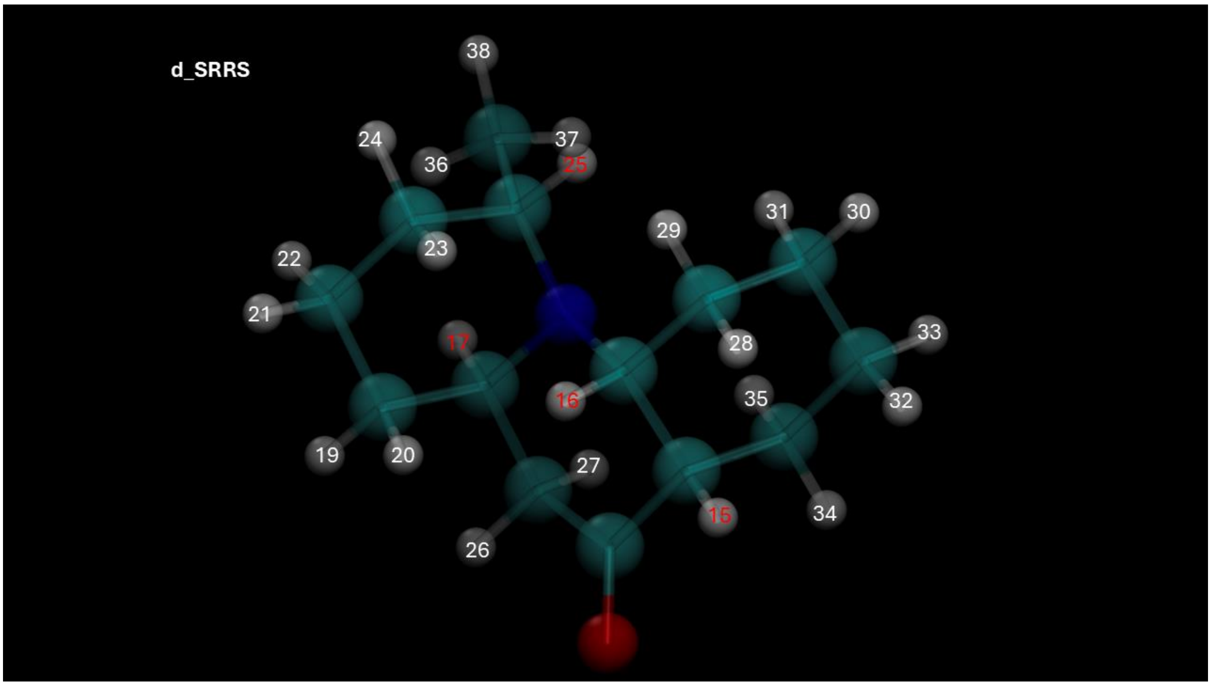

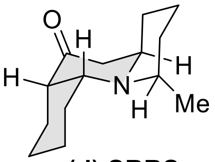

(d) SRRS

#H-H distances (d)
#H15
H15 H16 2.3796496391296387
H15 H26 4.187689304351807
H15 H27 3.8401198387145996
H15 H28 2.438356876373291
H15 H29 3.691164255142212
H15 H34 2.4593358039855957
H15 H35 3.0470058917999268
#H16
H16 H15 2.3796496391296387
H16 H17 3.7174596786499023
H16 H25 3.0498337745666504
H16 H26 3.997816562652588
H16 H27 4.008469104766846
H16 H28 2.344736099243164
H16 H29 2.480494260787964
#H17
H17 H18 2.587338924407959
H17 H21 3.7203502655029297
H17 H22 2.574742317199707
H17 H23 3.9005074501037598
H17 H24 3.933126926422119
H17 H25 3.581092357635498
H17 H26 2.5419044494628906
H17 H27 2.3100132942199707
H17 H28 5.431207656860352
#H18
H18 H17 2.587338924407959
H18 H19 4.441048622131348
H18 H20 4.486976623535156
H18 H21 4.198313236236572
H18 H22 2.9872381687164307
H18 H23 3.4712395668029785
H18 H24 2.7213799953460693
H18 H25 2.0896077156066895
H18 H26 5.0407867431640625
#H25
H25 H18 2.0896077156066895
H25 H23 2.384159803390503
H25 H24 2.5023841857910156
H25 H24 3.4704999923706055
H25 H24 1.9636330604553223
H25 H36 3.0284643173217773
H25 H37 2.457576036453247
H25 H38 2.354527473449707
#H29
H29 H16 2.480494260787964
H29 H27 5.364698886871338
H29 H28 1.7263227701187134
H29 H30 2.4654388427734375
H29 H31 2.524770975112915
H29 H32 3.757850408554077
H29 H33 4.3009352684021
H29 H36 4.879043102264404
H29 H37 4.179011344909668
H29 H38 4.301640510559082
#H27
H27 H17 2.3100132942199707
H27 H18 4.347268581390381
H27 H28 5.28061056137085
H27 H32 5.087078094482422
H27 H33 4.7950825691223145
H27 H34 3.246840476989746
H27 H35 2.359556198120117
H27 H36 4.204559326171875
H27 H37 4.098598957061768

H27 H38 5.43226957321167
#H28
H28 H17 5.431207656860352
H28 H18 5.292738437652588
H28 H27 5.28061056137085
H28 H32 2.575312852859497
H28 H33 3.743828773498535
H28 H34 4.03277063369751
H28 H35 3.9421305656433105
H28 H36 6.052840232849121
H28 H37 5.327759265899658
H28 H38 5.802742004394531
#H36
H36 H16 4.722336769104004
H36 H17 2.139509439468384
H36 H23 3.858893871307373
H36 H24 2.9959113597869873
H36 H25 3.0284643173217773
H36 H29 4.879043102264404
H36 H30 6.106171131134033
H36 H31 4.550611972808838
H36 H37 1.7539597749710083
H36 H38 1.7448527812957764
#H37
H37 H16 4.661746978759766
H37 H17 2.8368656635284424
H37 H23 4.286658763885498
H37 H24 3.743502140045166
H37 H25 2.457576036453247
H37 H29 4.179011344909668
H37 H30 4.772906303405762
H37 H31 3.1267457008361816
H37 H36 1.7539597749710083
H37 H38 1.7586594820022583
#H38
H38 H16 5.006158351898193
H38 H17 3.6111369132995605
H38 H23 3.7239768505096436
H38 H24 2.5703864097595215
H38 H25 2.354527473449707
H38 H28 5.802742004394531
H38 H29 4.301640510559082
H38 H30 5.481201171875
H38 H31 4.142645835876465
H38 H37 1.7586594820022583
H38 H36 1.7448527812957764

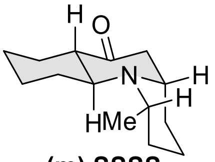

(m) SSSS

#H-H distances (m)
#H16
H16 H17 3.0083374977111816
H16 H18 3.7379627227783203
H16 H23 2.3686575889587402
H16 H24 3.1088271141052246
H16 H29 3.035841941833496
H16 H36 2.990018606185913
H16 H37 2.142939567565918
H16 H38 3.708496570587158
#H17
H17 H16 3.0083374977111816
H17 H18 2.968388795852661
H17 H23 3.720337390899658
H17 H24 2.7921721935272217
H17 H25 3.1125833988189697
H17 H26 2.355670213699341
H17 H27 3.8512699604034424
H17 H28 4.038520336151123
H17 H29 4.496638774871826
#H18
H18 H16 3.7379627227783203
H18 H17 2.968388795852661
H18 H27 3.0704305171966553
H18 H28 2.536369562149048
H18 H29 4.074857711791992
H18 H32 3.6930325031280518
H18 H33 2.383831739425659
H18 H34 3.087022066116333
H18 H35 2.367145538330078
H18 H36 4.101545333862305
H18 H37 4.728232383728027
H18 H38 5.159276008605957
#H29
H29 H16 3.035841941833496
H29 H17 4.496638774871826
H29 H18 4.074857711791992
H29 H23 2.3085873126983643
H29 H24 2.6996169090270996
H29 H25 5.636519908905029
H29 H30 2.623117446899414
H29 H31 2.3161489963531494
H29 H36 3.083657741546631
H29 H37 2.4352598190307617
H29 H38 2.442859411239624

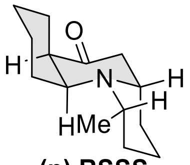

(n) RSSS

#H-H distances (n)
#H16
H16 H17 2.410846710205078
H16 H18 4.049373149871826
H16 H19 2.8937673568725586
H16 H20 3.923755645751953
H16 H21 3.84234356880188
H16 H34 3.876873254776001
H16 H35 4.382880687713623
#H17
H17 H16 2.410846710205078
H17 H18 3.7947001457214355
H17 H19 4.16529655456543
H17 H20 4.975197792053223
H17 H21 2.3642148971557617
H17 H23 4.29379940032959
H17 H34 2.2190282344818115
H17 H35 2.661545991897583
H17 H36 2.465543270111084
H17 H37 3.779594898223877
H17 H38 3.68558931350708
#H18
H18 H16 4.049373149871826
H18 H17 3.7947001457214355
H18 H19 3.0748443603515625
H18 H20 2.4219725131988525
H18 H21 4.1768412590026855
H18 H26 2.5001330375671387
H18 H27 2.3393492698669434
#H21
H21 H16 3.84234356880188
H21 H17 2.3642148971557617
H21 H18 4.1768412590026855
H21 H19 4.1768412590026855
H21 H20 4.2479071617126465
H21 H22 5.340999603271484
H21 H23 2.384725570678711
H21 H24 4.278432369232178
H21 H25 3.846802234649658

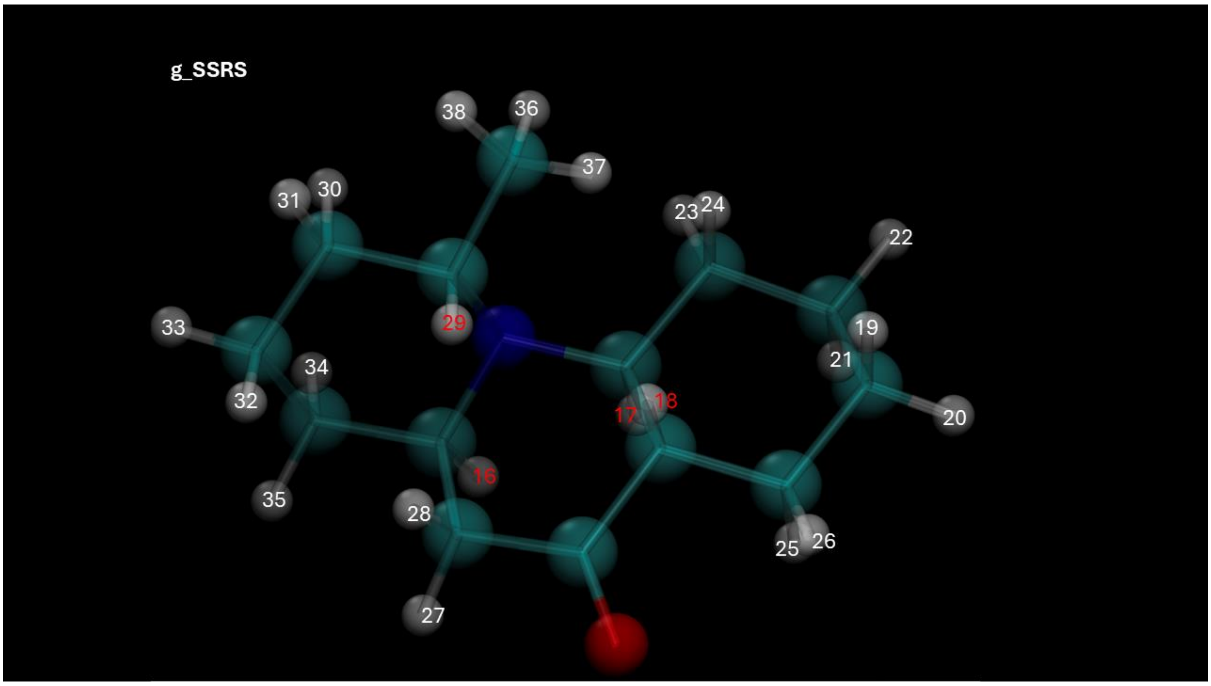

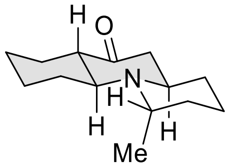

(g) SSRS

#H-H distances (g)
#H16
H16 H17 2.0152759552001953
H16 H18 3.941641092300415
H16 H27 2.4418652057647705
H16 H28 3.0411219596862793
H16 H29 3.7156567573547363
H16 H30 3.9745357036590576
H16 H31 4.835440158843994
H16 H34 2.32846736907959
H16 H35 2.4687793254852295
H16 H36 4.6831159591674805
H16 H37 4.9798102378845215
H16 H38 5.425002574920654
#H17

H17 H16 2.0152759552001953
H17 H18 3.020108461380005
H17 H23 2.3959810733795166
H17 H24 3.008091926574707
H17 H25 2.633697509765625
H17 H26 3.7554614543914795
H17 H27 3.7225165367126465
H17 H28 3.8765671253204346
H17 H29 3.8155646324157715
#H18
H18 H16 3.941641092300415
H18 H17 3.020108461380005
H18 H23 3.8283536434173584
H18 H24 2.7457363605499268
H18 H25 3.0372090339660645
H18 H26 2.436363458633423
H18 H27 3.950187921524048
H18 H28 3.0290799140930176
H18 H29 2.475264310836792
#H29
H29 H16 3.7156567573547363
H29 H17 3.8155646324157715
H29 H18 2.475264310836792
H29 H27 3.7548623085021973
H29 H28 2.234041929244995
H29 H30 3.024094820022583
H29 H31 2.4340503215789795
H29 H36 3.034071922302246
H29 H37 2.4089198112487793
H29 H38 2.5002710819244385
#H30
H30 H29 3.024094820022583
H30 H31 1.7497085332870483
H30 H18 5.017143726348877
H30 H19 6.799642086029053
H30 H23 4.285325527191162
H30 H24 4.560378074645996
H30 H36 2.6227376461029053
H30 H37 3.7365031242370605
H30 H38 2.8135030269622803
#H31
H31 H17 5.408580780029297
H31 H18 4.849887371063232
H31 H29 2.4340503215789795
H31 H30 1.7497085332870483
H31 H32 2.4459807872772217
H31 H33 2.5091631412506104
H31 H36 3.1588633060455322
H31 H37 3.557534694671631
H31 H38 2.25711989402771
#H34
H34 H15 4.559057712554932
H34 H17 3.8901147842407227
H34 H18 5.374241352081299
H34 H21 6.170171737670898
H34 H22 7.034179210662842
H34 H31 3.7662551403045654
H34 H33 2.432619333267212
H34 H35 1.7482794523239136
#H35
H35 H15 5.374346733093262
H35 H17 4.393375873565674
H35 H18 5.378992557525635
H35 H21 6.841216087341309
H35 H22 7.864568710327148
H35 H31 4.278370380401611
H35 H33 2.539233684539795
H35 H34 1.7482794523239136

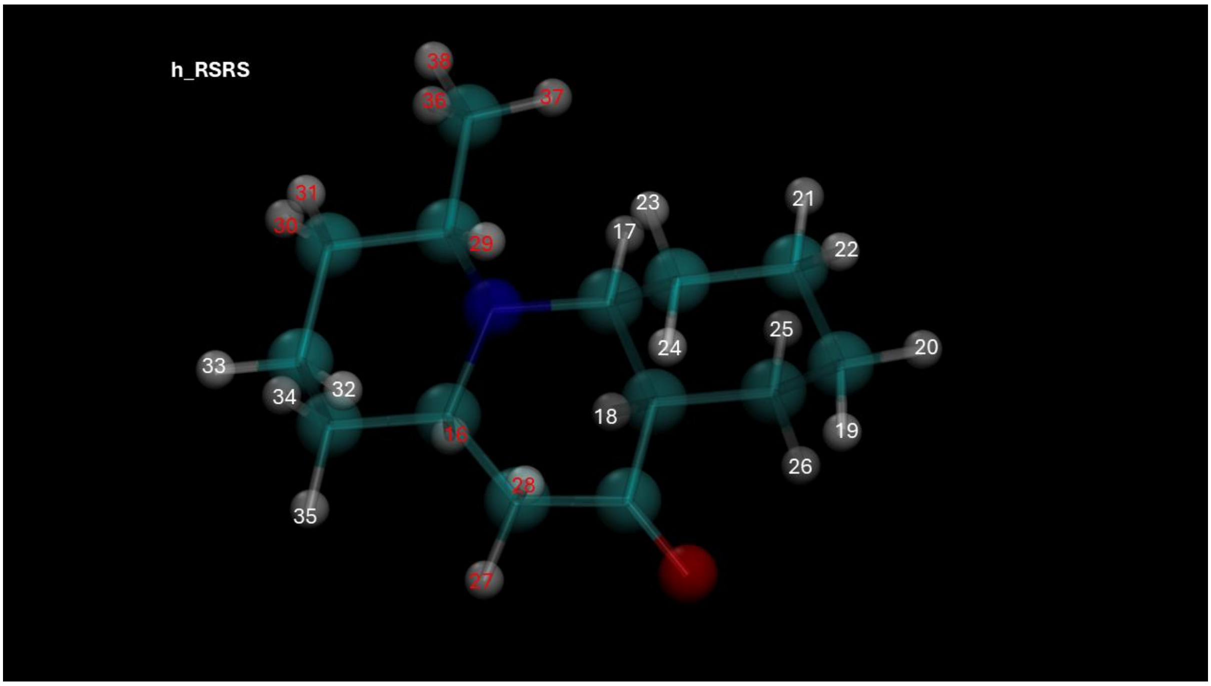

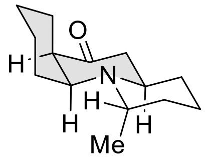

(h) RSRS

```
#H-H distances (h)
#H16
H16 H17 3.350390672683716
H16 H26 4.430899620056152
H16 H27 2.481499195098877
H16 H28 3.02990460395813
H16 H29 3.9941322803497314
H16 H30 3.741410255432129
H16 H31 4.771738052368164
H16 H32 3.8388314247131348
H16 H33 4.204478740692139
H16 H34 2.2537310123443604
H16 H35 2.5174591541290283
H16 H36 4.204206943511963
H16 H37 4.699637413024902
H16 H38 5.267868995666504
#H17
H17 H18 2.4282784461975098
H17 H21 2.4975643157958984
H17 H22 3.6885666847229004
H17 H23 2.5021302700042725
H17 H24 3.040989398956299
H17 H25 2.555913209915161
H17 H26 3.7217040061950684
H17 H27 4.84902811050415
H17 H28 4.185085296630859
#H18
H18 H17 2.4282784461975098
H18 H19 3.8419253826141357
H18 H20 4.263055324554443
H18 H21 4.112231731414795
H18 H22 4.93388032913208
H18 H23 4.289885520935059
H18 H24 3.770322799682617
H18 H25 2.382896661758423
H18 H26 2.4691481590270996
#H19
H19 H18 3.8419253826141357
H19 H20 1.7430168390274048
H19 H21 3.0451788902282715
H19 H22 2.457620859146118
H19 H23 3.789581537246704
H19 H24 2.6678597927093506
H19 H25 3.039156198501587
H19 H26 2.4396767616271973
#H29
H29 H16 3.9941322803497314
H29 H17 3.2968966960906982
H29 H27 4.498043060302734
H29 H28 3.1058058738708496
H29 H30 3.0078704357147217
H29 H31 2.330488681793213
H29 H36 3.042652130126953
H29 H37 2.5143191814422607
H29 H38 2.473994016647339

#H30
H30 H16 3.741410255432129
H30 H29 3.0078704357147217
H30 H31 1.7470238208770752
H30 H32 3.0455105304718018
H30 H33 2.4895401000976562
H30 H36 2.465907096862793
H30 H37 3.6824421882629395
H30 H38 2.8523879051208496

#H31
H31 H16 4.771738052368164
H31 H29 2.330488681793213
H31 H30 1.7470238208770752
H31 H32 2.521040439605713
H31 H33 2.494533061981201
H31 H36 3.1670281887054443
H31 H37 3.6934494972229004
H31 H38 2.460721254348755

#H36
H36 H16 4.204206943511963
H36 H17 2.9347081184387207
H36 H18 4.650691032409668
H36 H29 3.042652130126953

#H37
H37 H16 4.699637413024902
H37 H17 2.311356782913208
H37 H18 4.582381725311279
H37 H29 2.5143191814422607

#H38
H38 H16 5.267868995666504
H38 H17 3.82857084274292
H38 H18 5.779983043670654
H38 H29 2.473994016647339
```

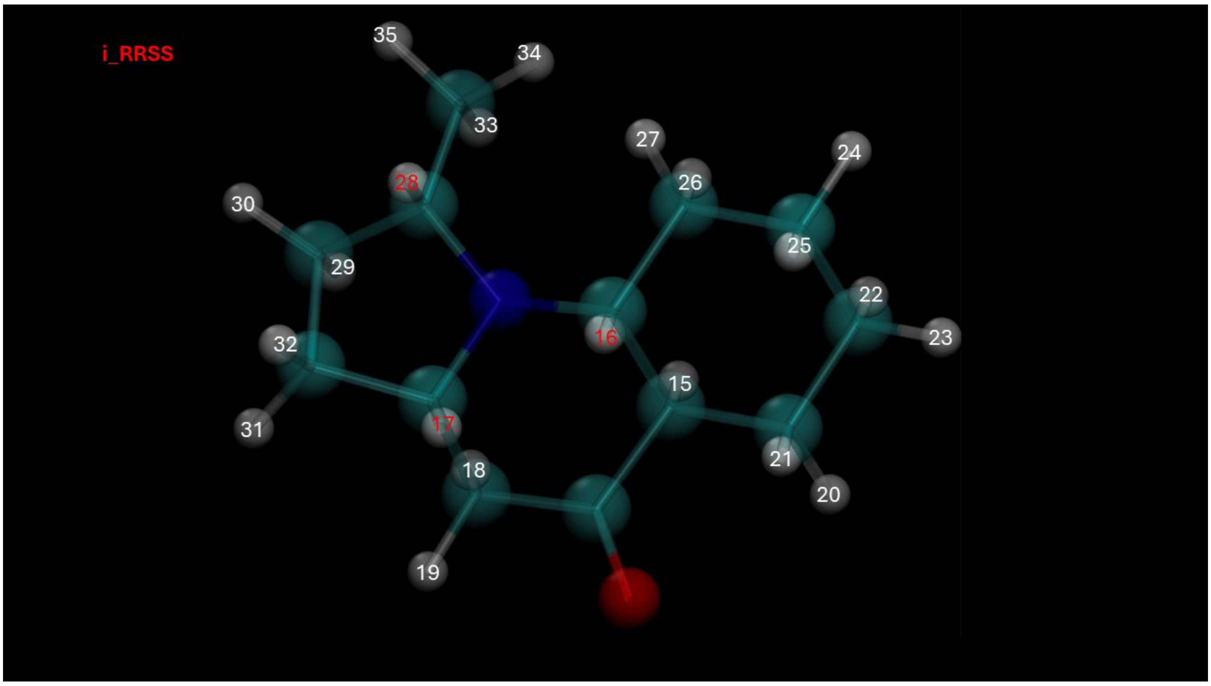

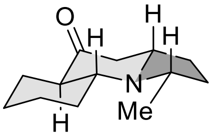

(i) RRSS

#H-H distances (i)
#H16
H16 H15 3.1122488975524902
H16 H17 2.284801959991455
H16 H18 4.041592597961426
H16 H19 4.1367974281311035
H16 H26 3.083815336227417
H16 H27 2.4589176177978516
H16 H28 3.033634901046753
H16 H33 4.527142524719238
H16 H34 3.925123929977417
H16 H35 4.977095127105713
#H17
H17 H15 3.9123947620391846
H17 H16 2.284801959991455
H17 H18 3.0781548023223877
H17 H19 2.494049072265625
H17 H29 3.7662999629974365
H17 H30 4.054847717285156
H17 H31 2.8022119998931885
H17 H32 2.2085933685302734
#H18
H18 H15 2.749983310699463
H18 H16 4.041592597961426
H18 H17 3.0781548023223877
H18 H19 1.8120825290679932
H18 H31 2.982492446899414
H18 H32 3.9380390644073486
#H28
H28 H16 3.033634901046753
H28 H17 2.989964723587036
H28 H29 3.104753017425537
H28 H30 2.5715372562408447
H28 H33 3.120378017425537
H28 H34 2.552609443664551
H28 H35 2.5420279502868652
#H33
H33 H26 2.917091131210327
H33 H27 3.5501701831817627
H33 H28 3.120378017425537
H33 H29 2.447922945022583
H33 H30 3.2515313625335693
H33 H34 1.7993377447128296
H33 H35 1.805186152458191
#H34
H34 H26 2.360694408416748
H34 H27 2.180738925933838
H34 H28 2.552609443664551
H34 H29 3.7174317836761475
H34 H30 3.8920042514801025
H34 H33 1.7993377447128296
H34 H35 1.7698321342468262
#H35
H35 H26 3.958726406097412
H35 H27 3.759351968765259
H35 H28 2.5420279502868652
H35 H29 3.0004727840423584
H35 H30 2.6653265953063965
H35 H33 1.805186152458191
H35 H34 1.7698321342468262

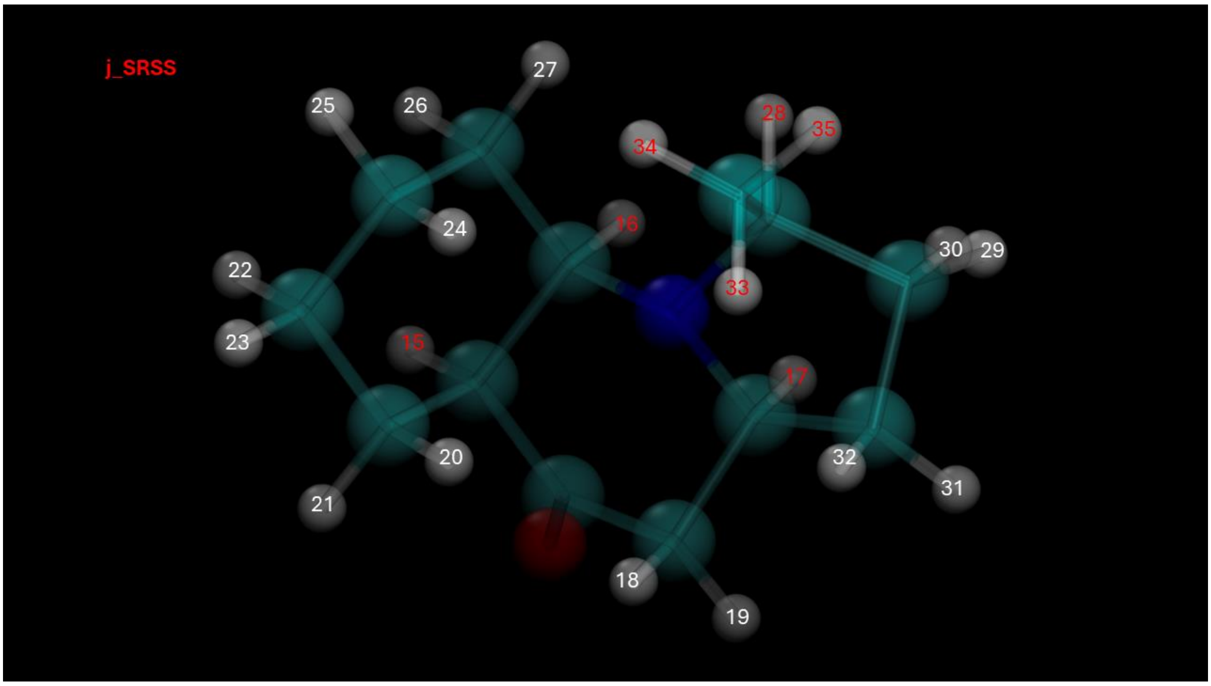

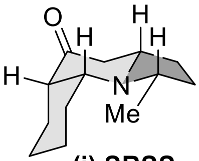

(j) SRSS

```
#H-H distances (j)
#H15
H15 H16 2.495083808898926
H15 H17 3.896345853805542
H15 H18 3.935655355453491
H15 H19 4.321197032928467
H15 H20 3.097569704055786
H15 H21 2.4777820110321045
H15 H21 2.4777820110321045
H15 H22 3.756240129470825
H15 H23 2.530355215072632
H15 H24 4.022121906280518
H15 H25 4.166080474853516
H15 H26 2.6069321632385254
H15 H27 3.8363447189331055
#H16
H16 H15 2.495083808898926
H16 H17 2.342231512069702
H16 H18 4.075924873352051
H16 H19 4.162993431091309
H16 H24 3.8745460510253906
H16 H25 4.322678565979004
H16 H26 2.4304158687591553
H16 H27 2.5368871688842773
H16 H28 2.650919198989868
H16 H33 4.6613688468933105
H16 H34 4.133923053741455
H16 H35 4.876774787902832
#H17
H17 H16 2.342231512069702
H17 H18 3.077601671218872
H17 H19 2.4791226387023926
H17 H28 3.1384966373443604
H17 H29 3.6087145805358887
H17 H30 2.2808046340942383
H17 H31 3.0709896087646484
H17 H32 2.4896132946014404
H17 H33 4.356574535369873
H17 H34 4.738417148590088
H17 H35 4.839174747467041
#H28
H28 H16 2.650919198989868
H28 H17 3.1384966373443604
H28 H29 2.776123285293579
H28 H30 2.3304359912872314
H28 H31 3.7946059703826904
H28 H32 4.190736293792725
H28 H33 3.0968177318573
H28 H34 2.4897682666778564
H28 H35 2.492147207260132
#H33
H33 H16 4.6613688468933105
H33 H17 4.356574535369873
H33 H28 3.0968177318573
H33 H29 2.8791604042053223
H33 H30 3.849177122116089
H33 H31 2.45281982421875
H33 H32 4.025505542755127
H33 H34 1.8063668012619019
H33 H35 1.8062231540679932
#H34
H34 H16 4.133923053741455
H34 H17 4.738417148590088
H34 H28 2.4897682666778564
H34 H29 3.734097480773926
H34 H30 4.271815776824951
H34 H31 3.9282071590423584
```

H34 H32 5.226253986358643
H34 H33 1.8063668012619019
H34 H35 1.779442548751831
#H35
H35 H16 4.876774787902832
H35 H17 4.839174747467041
H35 H28 2.492147207260132
H35 H29 2.4771957397460938
H35 H30 3.5719871520996094
H35 H31 3.6213347911834717
H35 H32 4.6798200607299805
H35 H33 1.8062231540679932
H35 H34 1.779442548751831

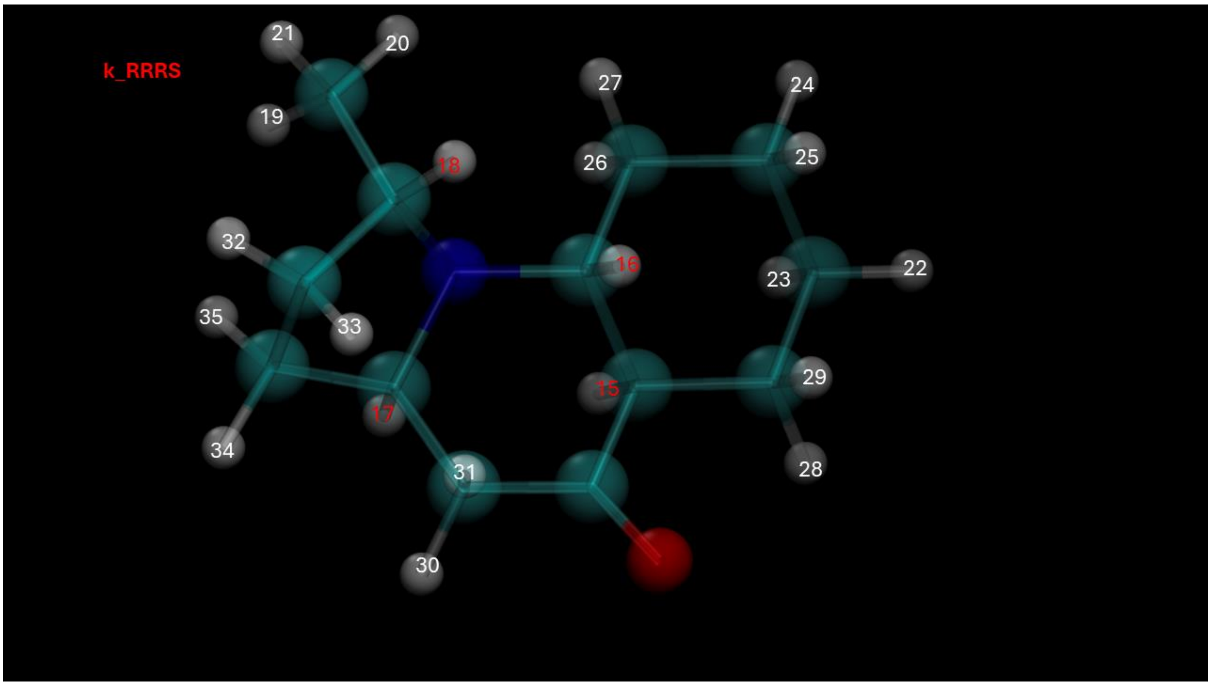

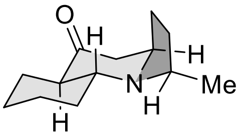

(k) RRRS

#H-H distances (k)
#H15
H15 H16 3.085693836212158
H15 H17 2.487208604812622
H15 H18 4.622262001037598
H15 H28 2.503389596939087
H15 H29 3.1006827354431152
H15 H30 3.526456356048584
H15 H31 3.8381845951080322
#H16
H16 H15 3.085693836212158
H16 H17 3.8341176509857178
H16 H19 4.527358055114746
H16 H20 3.8066647052764893
H16 H21 4.573228359222412
H16 H18 2.318532943725586
H16 H22 4.2001051902771
H16 H23 4.0356268882751465
H16 H24 3.8044886589050293
H16 H25 2.594539165496826
H16 H26 3.0972535610198975
H16 H27 2.50309419631958
H16 H28 3.8173515796661377
H16 H29 2.6132278442382812
#H17
H17 H15 2.487208604812622
H17 H16 3.8341176509857178
H17 H18 4.170382499694824
H17 H19 3.5988948345184326
H17 H20 4.401975631713867
H17 H21 4.905324935913086
H17 H30 2.4023351669311523
H17 H31 3.0921688079833984
H17 H32 4.2598676681518555
H17 H33 3.7365002632141113
H17 H34 2.7131595611572266
H17 H35 2.3970746994018555
#H18
H18 H16 2.318532943725586
H18 H17 4.170382499694824
H18 H19 3.0946826934814453
H18 H20 2.481955051422119
H18 H21 2.472580671310425
H18 H26 3.913487195968628
H18 H27 2.7869133949279785
H18 H32 2.72515869140625
H18 H33 2.373492956161499
H18 H34 4.256747722625732
H18 H35 3.756169319152832
#H19
H19 H18 3.0946826934814453

H19 H20 1.7930903434753418
H19 H21 1.8040953874588013
H19 H26 3.9446728229522705
H19 H27 3.788252830505371
H19 H32 2.9685420989990234
H19 H33 3.87245774269104
H19 H34 3.9036874771118164
H19 H35 2.2627968788146973
#H20
H20 H18 2.481955051422119
H20 H19 1.7930903434753418
H20 H21 1.7959100008010864
H20 H26 3.118790626525879
H20 H27 2.3467459678649902
H20 H32 3.783447027206421
H20 H33 4.297637939453125
H20 H34 5.144302845001221
H20 H35 3.7709269523620605
#H21
H21 H18 2.472580671310425
H21 H19 1.8040953874588013
H21 H20 1.7959100008010864
H21 H26 4.757336139678955
H21 H27 3.863662004470825
H21 H32 2.5485825538635254
H21 H33 3.665628671646118
H21 H34 4.63405704498291
H21 H35 3.427595853805542

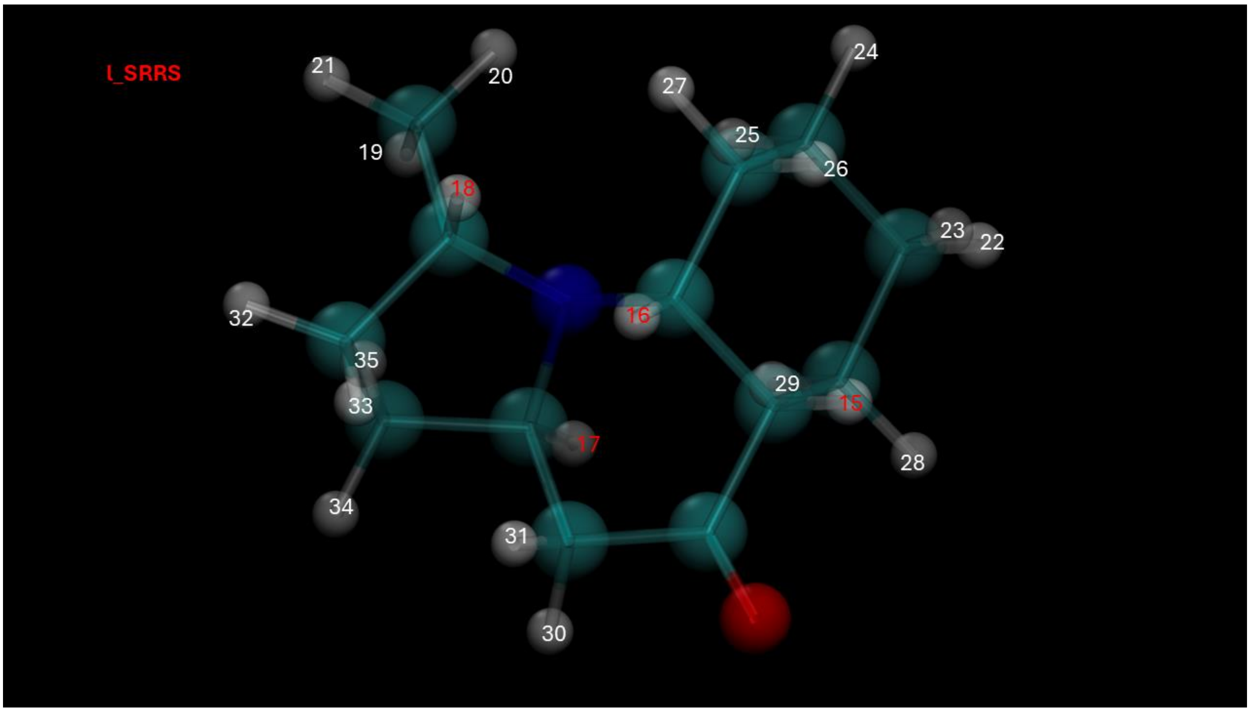

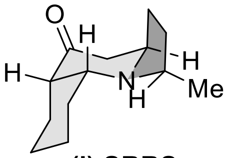

(l) SRRS

#H-H distances (l)
#H15
H15 H16 2.4585139751434326
H15 H17 4.171119689941406
H15 H18 4.629546165466309
H15 H28 3.095231533050537
H15 H29 2.477414608001709
H15 H30 4.2763991355896
H15 H31 3.81459379196167
#H16
H16 H15 2.4585139751434326
H16 H17 3.8265459537506104
H16 H18 2.30694317817688
H16 H22 4.138825416564941
H16 H23 5.011595249176025
H16 H24 3.8325140476226807
H16 H25 4.3307623863220215
H16 H26 2.5412354469299316
H16 H27 2.4663708209991455
H16 H28 3.8752453327178955
H16 H29 4.33194637298584
#H17
H17 H15 4.171119689941406
H17 H16 3.8265459537506104
H17 H18 4.173085689544678
H17 H19 3.493023633956909
H17 H20 4.333494186401367
H17 H21 4.8263678550720215
H17 H30 2.3852808475494385
H17 H31 3.0869362354278564
H17 H32 4.229710102081299
H17 H33 3.717257022857666

H17 H34 2.3629543781280518
H17 H35 2.6942803859710693
#H18
H18 H16 2.30694317817688
H18 H17 4.173085689544678
H18 H19 3.0901830196380615
H18 H20 2.4651987552642822
H18 H21 2.461366653442383
H18 H24 4.022594928741455
H18 H25 4.838090896606445
H18 H26 2.536687135696411
H18 H27 3.7707443237304688
H18 H32 2.699371814727783
H18 H33 2.383004903793335
H18 H34 3.7398605346679688
H18 H35 4.259425163269043
#H19
H19 H18 3.0901830196380615
H19 H20 1.7919995784759521
H19 H21 1.8045034408569336
H19 H26 3.901251792907715
H19 H27 5.368136405944824
H19 H32 3.0204923152923584
H19 H33 3.893623113632202
H19 H34 2.232694387435913
H19 H35 3.8847174644470215
#H20
H20 H18 2.4651987552642822
H20 H19 1.7919995784759521
H20 H21 1.7982237339019775
H20 H26 2.452756881713867
H20 H27 4.152398586273193
H20 H32 3.8007736206054688
H20 H33 4.301331996917725
H20 H34 3.747098684310913
H20 H35 5.125955581665039
#H21
H21 H18 2.461366653442383
H21 H19 1.8045034408569336
H21 H20 1.7982237339019775
H21 H26 3.8341431617736816
H21 H27 5.517261981964111
H21 H32 2.5709259510040283
H21 H33 3.6968696117401123
H21 H34 3.369710683822632
H21 H35 4.607275009155273

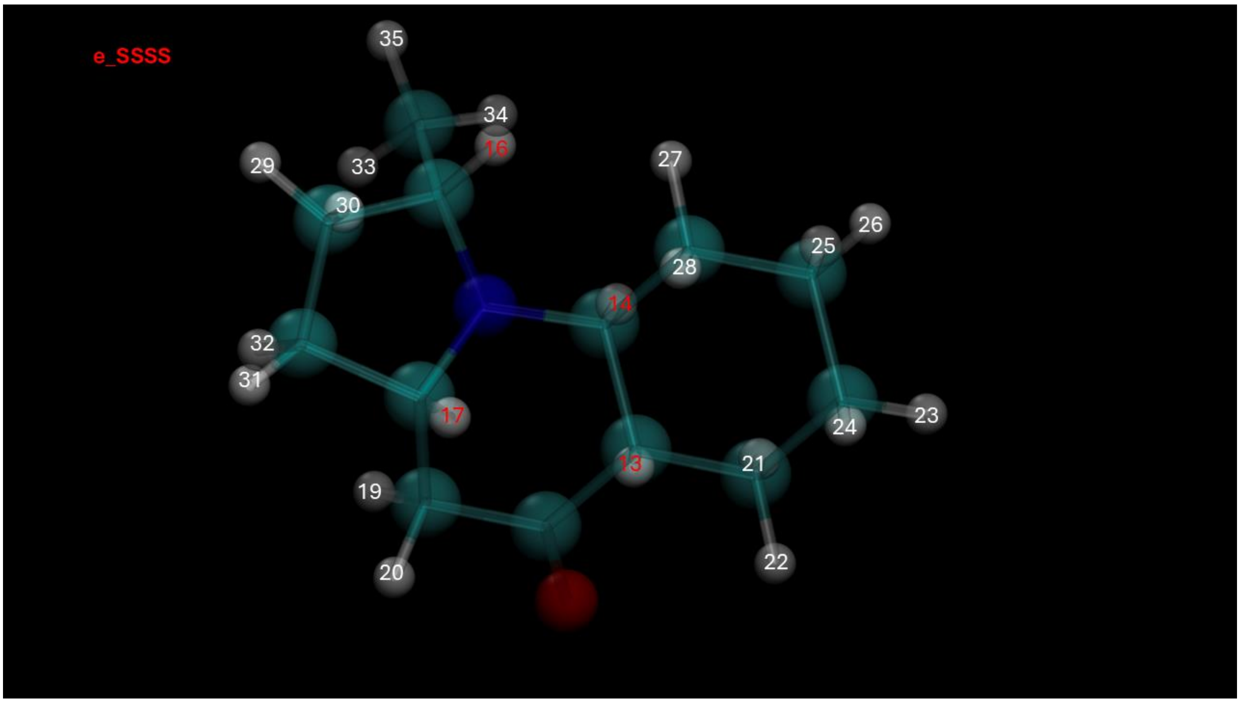

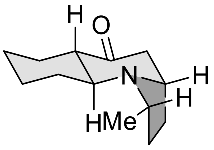

(e) SSSS

#H-H distances (e)
#H13
H13 H14 3.0170278549194336
H13 H17 2.3418188095092773
H13 H21 3.036106824874878
H13 H22 2.4799468517303467
H13 H27 3.678208827972412
H13 H28 2.4737913608551025
#H14
H14 H13 3.0170278549194336
H14 H16 3.2962803840637207
H14 H17 3.825310707092285

H14 H27 2.477142810821533
H14 H28 3.0314104557037354
H14 H33 3.4569246768951416
H14 H34 2.6364147663116455
H14 H35 4.158455848693848
#H16
H16 H14 3.2962803840637207
H16 H17 3.3161733150482178
H16 H27 2.1678738594055176
H16 H28 2.683210611343384
H16 H29 2.7782106399536133
H16 H30 2.2871384620666504
H16 H33 3.0405633449554443
H16 H34 2.507309675216675
H16 H35 2.443206787109375
#H17
H17 H13 2.3418188095092773
H17 H14 3.825310707092285
H17 H16 3.3161733150482178
H17 H19 3.0389602184295654
H17 H20 2.4148738384246826
H17 H29 3.8314990997314453
H17 H30 2.779426097869873
H17 H31 2.3972535133361816
H17 H32 3.021347761154175
#H19
H19 H13 3.7167749404907227
H19 H17 3.0389602184295654
H19 H20 1.7494990825653076
H19 H31 3.2642266750335693
H19 H32 2.3504457473754883
#H20
H20 H13 3.181819200515747
H20 H17 2.4148738384246826
H20 H19 1.7494990825653076
H20 H31 3.087502956390381
H20 H32 3.161036252975464
#H33
H33 H14 3.4569246768951416
H33 H16 3.0405633449554443
H33 H17 4.516113758087158
H33 H29 2.6942074298858643
H33 H30 3.8009583950042725
H33 H34 1.7537988424301147
H33 H35 1.7637031078338623
#H34
H34 H14 2.6364147663116455
H34 H16 2.507309675216675
H34 H17 4.796438217163086
H34 H29 3.6941585540771484
H34 H30 4.255570411682129
H34 H33 1.7537988424301147
H34 H35 1.7543723583221436
#H35
H35 H14 4.158455848693848
H35 H16 2.443206787109375
H35 H17 5.08101749420166
H35 H29 2.590613603591919
H35 H30 3.550642967224121
H35 H33 1.7637031078338623
H35 H34 1.7543723583221436

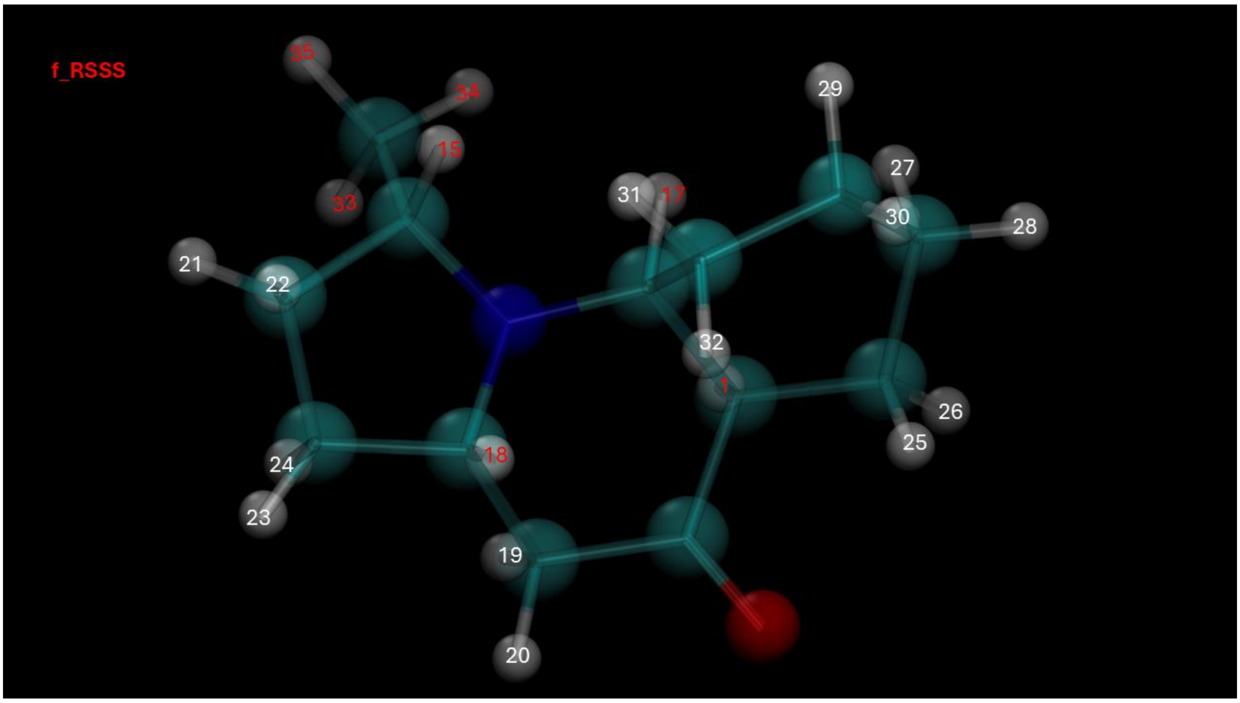

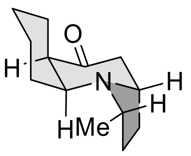

(f) RSSS

#H-H distances (f)
#H1
H1 H17 2.3129048347473145
H1 H18 4.020094871520996
H1 H19 2.7779102325439453
H1 H20 3.8382232189178467
H1 H25 2.991110324859619
H1 H26 2.3345465660095215
H1 H31 4.262908458709717
H1 H32 3.725621223449707
#H15
H15 H17 2.7013819217681885
H15 H18 3.1970043182373047
H15 H18 3.1970043182373047
H15 H21 2.8101143836975098
H15 H22 2.2970542907714844
H15 H23 4.0644426345825195
H15 H24 3.865967035293579
H15 H33 3.041111707687378
H15 H34 2.509404420852661
H15 H35 2.4456374645233154
#H17
H17 H1 2.3129048347473145
H17 H15 2.7013819217681885
H17 H18 3.7525882720947266
H17 H31 2.562046527862549
H17 H29 2.791614055633545
H17 H30 3.668734550476074
H17 H32 3.025036334991455
H17 H33 3.5101168155670166
H17 H34 2.4005305767059326
H17 H35 3.8300156593322754
#H18
H18 H1 4.020094871520996
H18 H15 3.1970043182373047
H18 H17 3.7525882720947266
H18 H19 3.0326104164123535
H18 H20 2.4628570079803467
H18 H21 3.7981488704681396
H18 H22 2.7431766986846924
H18 H23 2.4123756885528564
H18 H24 3.0266263484954834
H18 H33 4.519864082336426
H18 H34 4.75467586517334
H18 H35 5.0253825187683105
#H19
H19 H1 2.7779102325439453
H19 H18 3.0326104164123535
H19 H20 1.7652992010116577
H19 H23 3.3458595275878906
H19 H24 2.443786144256592
#H20
H20 H1 3.8382232189178467
H20 H18 2.4628570079803467
H20 H19 1.7652992010116577
H20 H23 3.0342538356781006
H20 H24 3.124263048171997
H20 H25 4.502316951751709
H20 H26 5.103431701660156
#H33
H33 H15 3.041111707687378
H33 H17 3.5101168155670166
H33 H18 4.519864082336426
H33 H21 2.650505304336548
H33 H22 3.7817606925964355
H33 H31 4.8817620277404785
H33 H34 1.754231333732605
H33 H35 1.7633055448532104
#H34
H34 H15 2.509404420852661
H34 H17 2.4005305767059326
H34 H18 4.75467586517334
H34 H21 3.683234930038452
H34 H22 4.249947547912598
H34 H31 4.009983539581299
H34 H33 1.754231333732605
H34 H35 1.7541545629501343
#H35
H35 H15 2.4456374645233154
H35 H17 3.8300156593322754
H35 H18 5.0253825187683105
H35 H21 2.5953474044799805
H35 H22 3.522171974182129
H35 H31 4.587160587310791
H35 H33 1.7633055448532104
H35 H34 1.7541545629501343

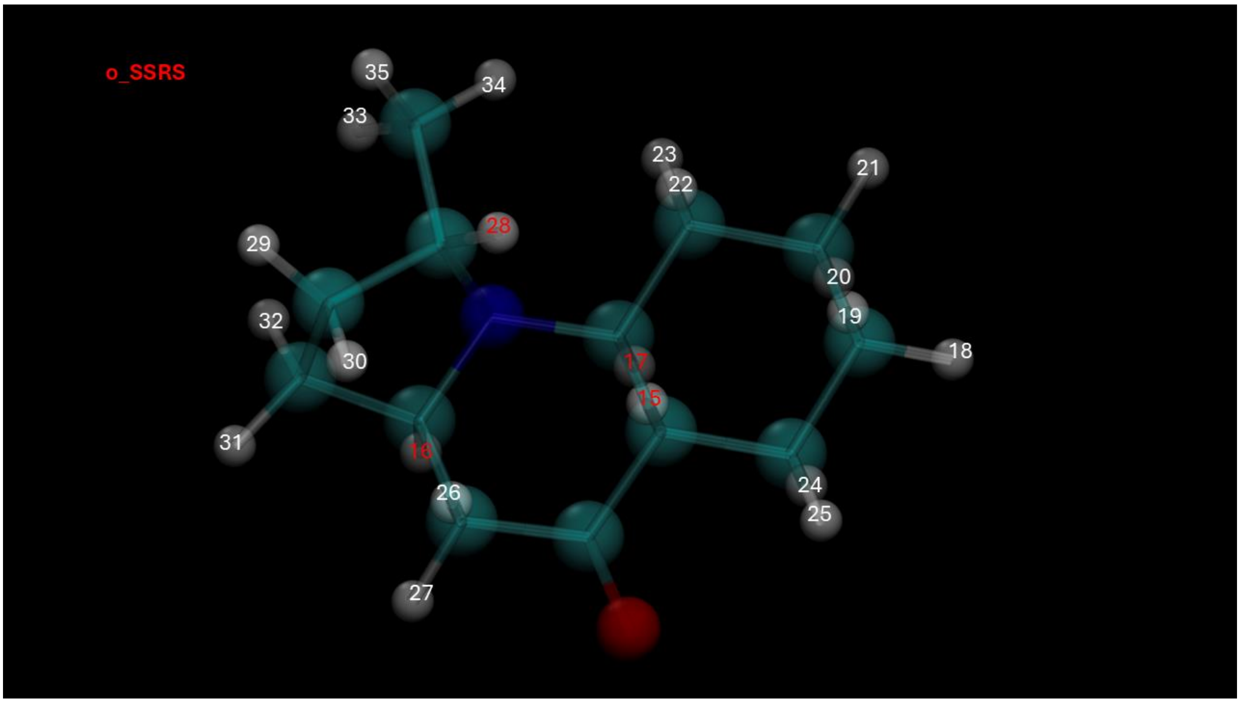

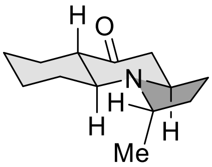

(o) SSRS

```
#H-H distances (o)
#H15
H15 H16 4.088494777679443
H15 H17 3.0854458808898926
H15 H18 2.5609021186828613
H15 H19 3.7810637950897217
H15 H22 3.830350160598755
H15 H23 2.6344478130340576
H15 H24 3.098076105117798
H15 H25 2.4952094554901123
H15 H26 3.8041324615478516
H15 H27 2.547654628753662
H15 H33 5.158360958099365
H15 H34 4.4919633865356445
H15 H35 5.061049938201904
#H16
H16 H15 4.088494777679443
H16 H17 2.6723804473876953
H16 H26 2.457061767578125
H16 H27 3.1132326126098633
H16 H28 4.133126258850098
H16 H29 4.286613464355469
H16 H30 3.7701027393341064
H16 H31 2.4294681549072266
H16 H32 2.701629161834717
H16 H33 3.8762779235839844
H16 H34 4.606853485107422
H16 H35 5.088926315307617
#H17
H17 H15 3.0854458808898926
H17 H16 2.6723804473876953
H17 H19 4.125362873077393
H17 H22 2.475929021835327
H17 H23 3.0856380462646484
H17 H24 2.5437567234039307
H17 H25 3.7647597789764404
H17 H26 4.123026371002197
H17 H27 4.008006572723389
H17 H28 3.814561367034912
H17 H33 4.2830095291137695
H17 H34 3.925229549407959
H17 H35 5.183022499084473
#H28
H28 H15 2.629917621612549
H28 H16 4.133126258850098
H28 H17 3.814561367034912
H28 H29 2.7859625816345215
H28 H30 2.331979990005493
H28 H31 3.781179428100586
H28 H32 4.209290504455566
H28 H33 3.101209878921509
H28 H34 2.5152978897094727
H28 H35 2.4944443702697754
#H33
H33 H22 3.5590481758117676
H33 H23 4.051008224487305
H33 H28 3.101209878921509
H33 H29 2.8443870544433594
H33 H30 3.8305044174194336
H33 H34 1.7990068197250366
H33 H35 1.8054871559143066
#H34
H34 H22 2.3016841411590576
```

H34 H23 2.6235876083374023
H34 H28 2.5152978897094727
H34 H29 3.740640163421631
H34 H30 4.278548240661621
H34 H35 1.7898082733154297
#H35
H35 H22 4.0069732666015625
H35 H23 3.7968342304229736
H35 H28 2.4944443702697754
H35 H29 2.4996337890625
H35 H30 3.567727565765381
H35 H34 1.7898082733154297

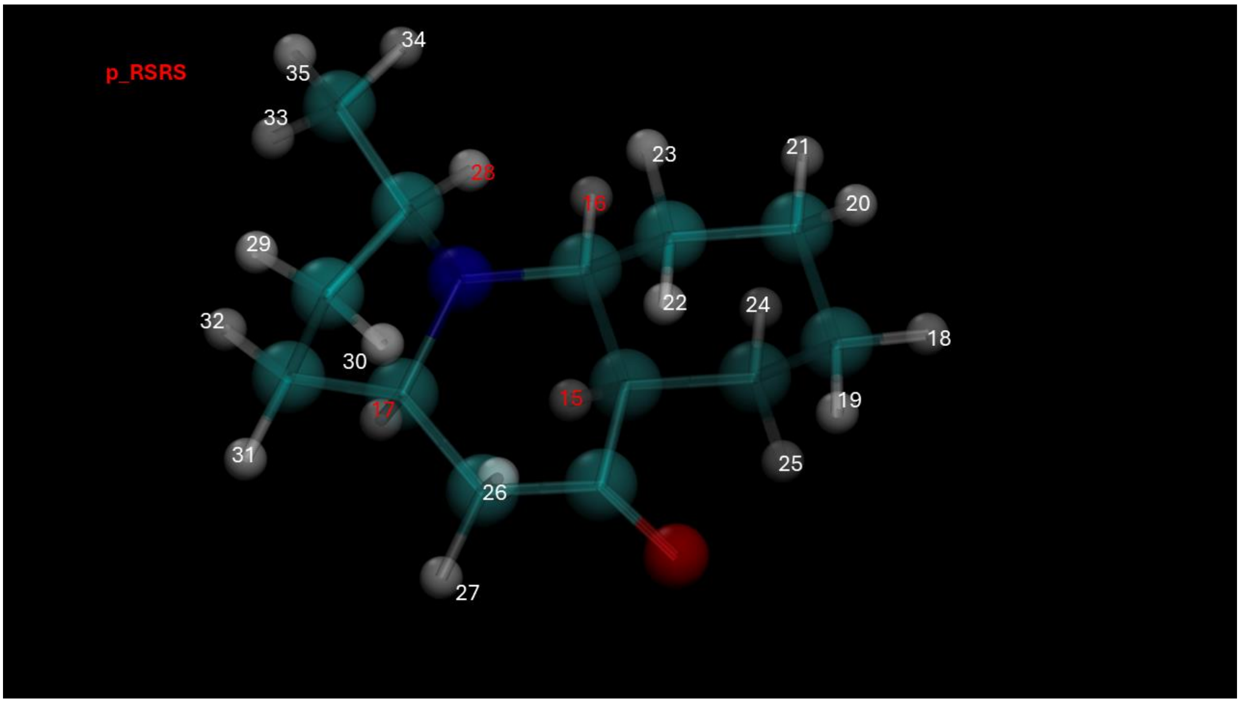

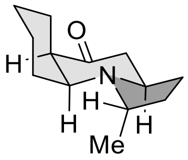

(p) RSRS

#H-H distances (p)
#H15
H15 H16 2.4710044860839844
H15 H17 2.3540866374969482
H15 H18 3.8630640506744385
H15 H19 4.317279815673828
H15 H22 4.349453449249268
H15 H23 3.861372947692871
H15 H24 2.439171552658081
H15 H25 2.522521734237671
H15 H26 3.4504435062408447
H15 H27 3.8187146186828613
#H16
H16 H15 2.4710044860839844
H16 H17 3.4939305782318115
H16 H26 4.917600154876709
H16 H27 4.387388706207275
H16 H28 3.2910819053649902
H16 H29 5.287528991699219
H16 H30 4.724786281585693
H16 H31 4.619485855102539
H16 H32 5.442877769470215
#H17
H17 H15 2.3540866374969482
H17 H16 3.4939305782318115
H17 H19 6.318894386291504
H17 H22 4.877645015716553
H17 H23 4.378373146057129
H17 H24 4.688466548919678
H17 H25 4.701684951782227
H17 H26 2.4232780933380127
H17 H27 3.094139814376831
H17 H28 4.180204391479492
#H28
H28 H15 4.754049301147461
H28 H16 3.2910819053649902
H28 H17 4.180204391479492
H28 H29 2.688046932220459
H28 H30 2.3461854457855225
H28 H31 3.7424983978271484
H28 H32 4.238032817840576
H28 H33 3.0876715183258057
H28 H34 2.478321075439453
H28 H35 2.4635419845581055
#H33
H33 H22 4.516806602478027
H33 H23 5.191020488739014
H33 H28 3.0876715183258057

H33 H29 3.00779390335083 H33 H30 3.887315511703491

12. Atomic coordinates

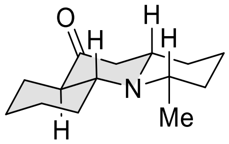

(a) RRSS

(a) RRSS

C	1.9382000000	-0.4002000000	-1.1740000000
C	1.8362000000	1.0707000000	-0.7443000000
C	0.4565000000	1.6441000000	-1.0991000000
C	-0.6599000000	0.8074000000	-0.4337000000
C	-0.5495000000	-0.6938000000	-0.8351000000
C	0.8517000000	-1.2362000000	-0.4825000000
N	-2.0334000000	1.3550000000	-0.6508000000
C	-3.0633000000	0.5531000000	0.0801000000
C	-3.0576000000	-0.9300000000	-0.3450000000
C	-1.6623000000	-1.4423000000	-0.1468000000
C	-2.4053000000	1.9160000000	-1.9936000000
C	-3.8048000000	2.5605000000	-1.9405000000
C	-4.8516000000	1.5828000000	-1.4029000000
C	-4.4560000000	1.2043000000	0.0206000000
O	-1.4441000000	-2.3805000000	0.6063000000
C	-2.3094000000	0.9470000000	-3.1921000000
H	-2.7964000000	0.5442000000	1.1629000000
H	-0.4704000000	0.8642000000	0.6641000000
H	-0.6914000000	-0.8217000000	-1.9211000000
H	2.9397000000	-0.7967000000	-0.9010000000
H	1.8227000000	-0.4761000000	-2.2778000000
H	2.6268000000	1.6648000000	-1.2510000000
H	1.9984000000	1.1451000000	0.3537000000
H	0.3545000000	1.6358000000	-2.2026000000

H	0.3977000000	2.6974000000	-0.7494000000
H	0.9401000000	-2.2967000000	-0.8043000000
H	1.0091000000	-1.1908000000	0.6184000000
H	-3.3568000000	-1.0630000000	-1.3956000000
H	-3.7725000000	-1.5123000000	0.2769000000
H	-1.7095000000	2.7484000000	-2.2338000000
H	-4.0995000000	2.9359000000	-2.9447000000
H	-3.7689000000	3.4416000000	-1.2612000000
H	-5.8428000000	2.0849000000	-1.3805000000
H	-4.9392000000	0.6840000000	-2.0474000000
H	-5.2166000000	0.5412000000	0.4866000000
H	-4.4267000000	2.1424000000	0.6193000000
H	-1.2765000000	0.6038000000	-3.3675000000
H	-2.5897000000	1.4900000000	-4.1199000000
H	-2.9844000000	0.0808000000	-3.1226000000

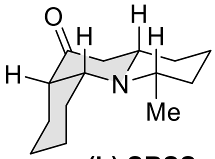

(b) SRSS

(b) SRSS

C	2.5818000000	-1.6546000000	1.1264000000
C	3.0597000000	-0.5968000000	0.1202000000
C	2.1428000000	0.6259000000	0.1933000000
C	0.6566000000	0.2871000000	-0.0524000000
C	0.1490000000	-0.9457000000	0.7667000000
C	1.1575000000	-2.1189000000	0.7985000000
N	0.1864000000	0.3343000000	-1.4620000000
C	-1.2965000000	0.3207000000	-1.4979000000
C	-1.8665000000	-1.0129000000	-1.0013000000
C	-1.1969000000	-1.4805000000	0.2772000000
C	0.9420000000	-0.1657000000	-2.6522000000

C	0.3352000000	0.4413000000	-3.9361000000
C	-1.1700000000	0.1570000000	-4.0334000000
C	-1.8575000000	0.8158000000	-2.8425000000
O	-1.7069000000	-2.4145000000	0.8785000000
C	1.0213000000	-1.6954000000	-2.8125000000
H	-1.6788000000	1.0635000000	-0.7588000000
H	0.1428000000	1.1646000000	0.4057000000
H	0.0625000000	-0.5979000000	1.8212000000
H	2.6035000000	-1.2254000000	2.1526000000
H	3.2677000000	-2.5286000000	1.1048000000
H	3.0948000000	-1.0131000000	-0.9010000000
H	4.0941000000	-0.2868000000	0.3827000000
H	2.4773000000	1.4430000000	-0.4810000000
H	2.2344000000	1.0072000000	1.2361000000
H	1.1614000000	-2.6612000000	-0.1522000000
H	0.8360000000	-2.8655000000	1.5575000000
H	-1.7867000000	-1.8212000000	-1.7509000000
H	-2.9516000000	-0.8561000000	-0.8133000000
H	1.9880000000	0.2042000000	-2.6041000000
H	0.8606000000	0.0545000000	-4.8365000000
H	0.4862000000	1.5438000000	-3.9202000000
H	-1.5677000000	0.6027000000	-4.9705000000
H	-1.3808000000	-0.9313000000	-4.0620000000
H	-2.9605000000	0.6854000000	-2.8822000000
H	-1.6525000000	1.9079000000	-2.9162000000
H	1.6448000000	-2.1579000000	-2.0493000000
H	1.5447000000	-1.9383000000	-3.7619000000
H	0.0388000000	-2.1925000000	-2.8447000000

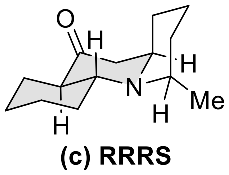

(c) RRRS

(c) RRRS

C	0.1787015857	-1.0415525276	0.5882036830
C	-0.0509734954	-0.9203794373	2.0968454767
C	1.0258169363	-1.7011584291	2.8559992147
C	2.4471673127	-1.2406701107	2.4487672950
N	2.6573500462	-1.3444576576	0.9699831155
C	1.6073143888	-0.5555888751	0.2379387067
C	2.7860815074	-2.7585039094	0.4574361844
C	3.0673304593	-2.7546658778	-1.0747965857
C	2.0017565328	-1.9462588314	-1.7517945949
C	1.8594216164	-0.5459377275	-1.2768425079
C	3.9277862281	-3.5621127563	1.1466995818
C	4.0336098305	-4.9847051376	0.5750828586
C	4.2869836970	-4.9508624388	-0.9319983519
C	3.1621616272	-4.1885565695	-1.6341794398
O	1.2740420788	-2.4006619107	-2.6228780594
C	2.7781624286	0.1719118620	2.9711334014
H	1.6634879971	0.5129424488	0.5284973002
H	4.0390491893	-2.2426910580	-1.2611910126
H	1.8407781693	-3.3133783058	0.6329807757
H	-0.5775623877	-0.4214677504	0.0597824476
H	0.0258508717	-2.0931458270	0.2694558370
H	-1.0588372456	-1.3065360143	2.3603926622
H	-0.0158850939	0.1500020923	2.3897319694
H	0.9099312277	-2.7804335672	2.6233905325
H	0.8795032599	-1.5864744237	3.9517717987
H	3.1745521969	-1.8651651375	2.9933062788
H	1.0496662824	-0.0047998430	-1.8139420228
H	2.8184321804	-0.0202522925	-1.4782383543
H	3.7476220837	-3.6965774140	2.2293193234
H	4.8946564764	-3.0304441855	1.0068003372

H	3.0879085242	-5.5347884145	0.7779326710
H	4.8635389983	-5.5274106811	1.0768541621
H	4.3402813847	-5.9881306132	-1.3268175687
H	5.2599949725	-4.4508103341	-1.1339533386
H	2.1976235439	-4.7216924096	-1.4786318905
H	3.3750972888	-4.1582415495	-2.7250904718
H	2.1084746144	0.9625757177	2.5858588426
H	3.8216114332	0.4348771863	2.6960710325
H	2.6987818048	0.1893169148	4.0792752735

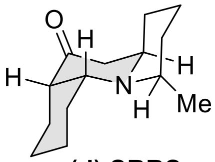

(d) SRRS

(d) SRRS

C	1.2205000000	0.8265000000	1.4478000000
C	1.0037000000	2.2632000000	0.9661000000
C	2.1376000000	3.1614000000	1.4684000000
C	3.5218000000	2.6139000000	1.0410000000
N	3.7205000000	1.2022000000	1.4955000000
C	2.6099000000	0.3243000000	0.9813000000
C	3.9280000000	1.0554000000	2.9846000000
C	4.2232000000	-0.4256000000	3.3475000000
C	3.1098000000	-1.2646000000	2.7987000000
C	2.8348000000	-1.1560000000	1.3377000000
C	5.0874000000	1.9144000000	3.5674000000
C	6.4445000000	1.4912000000	2.9922000000
C	6.7146000000	0.0194000000	3.3286000000
C	5.5942000000	-0.8664000000	2.7763000000
O	2.4144000000	-1.9569000000	3.5261000000
H	4.2594000000	-0.5422000000	4.4550000000
H	3.0092000000	1.3645000000	3.5233000000

H	2.5953000000	0.3383000000	-0.1273000000
C	3.7692000000	2.7606000000	-0.4734000000
H	0.4191000000	0.1830000000	1.0237000000
H	1.1277000000	0.7819000000	2.5519000000
H	0.0256000000	2.6430000000	1.3316000000
H	0.9750000000	2.2793000000	-0.1438000000
H	2.0878000000	3.2065000000	2.5766000000
H	1.9977000000	4.1984000000	1.0935000000
H	4.2952000000	3.2742000000	1.4687000000
H	1.9674000000	-1.7797000000	1.0290000000
H	3.7189000000	-1.5309000000	0.7831000000
H	5.1165000000	1.7736000000	4.6712000000
H	4.9253000000	2.9989000000	3.4267000000
H	7.2489000000	2.1179000000	3.4336000000
H	6.4502000000	1.6418000000	1.8904000000
H	6.7700000000	-0.1037000000	4.4330000000
H	7.6898000000	-0.2926000000	2.8969000000
H	5.7947000000	-1.9254000000	3.0489000000
H	5.6127000000	-0.7826000000	1.6693000000
H	3.0471000000	2.2079000000	-1.1021000000
H	4.7914000000	2.4018000000	-0.7191000000
H	3.7008000000	3.8316000000	-0.7617000000

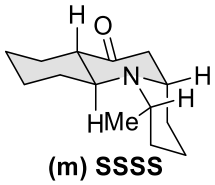

(m) SSSS

(m) SSSS - Geph-6Mem

C	1.7019000000	-2.2265000000	-1.0553000000
C	2.8555000000	-2.5626000000	-1.9931000000
C	3.8042000000	-1.3385000000	-2.1164000000
C	3.9017000000	-0.4843000000	-0.8126000000

C	3.6901000000	-1.4088000000	0.4021000000
C	2.2408000000	-1.9815000000	0.3799000000
N	5.1780000000	0.2953000000	-0.7632000000
C	5.6366000000	0.7037000000	0.6130000000
C	4.4954000000	0.7053000000	1.6415000000
C	3.9821000000	-0.7023000000	1.6964000000
C	5.3018000000	1.3423000000	-1.8178000000
C	6.7371000000	1.9443000000	-1.8495000000
C	7.4524000000	1.9734000000	-0.4878000000
C	6.4484000000	2.0248000000	0.6501000000
O	3.9217000000	-1.3023000000	2.7592000000
C	4.2123000000	2.4418000000	-1.7099000000
H	3.0381000000	0.2108000000	-0.7546000000
H	4.4231000000	-2.2415000000	0.3028000000
H	6.3594000000	-0.0823000000	0.9352000000
H	0.9189000000	-3.0139000000	-1.0271000000
H	1.2321000000	-1.2994000000	-1.4534000000
H	2.5158000000	-2.8691000000	-3.0053000000
H	3.3935000000	-3.4269000000	-1.5435000000
H	3.4488000000	-0.6579000000	-2.9196000000
H	4.7958000000	-1.7402000000	-2.4186000000
H	1.5111000000	-1.3019000000	0.8734000000
H	2.2685000000	-2.9280000000	0.9628000000
H	3.6993000000	1.4050000000	1.3085000000
H	4.8478000000	1.0179000000	2.6492000000
H	5.1450000000	0.9054000000	-2.8270000000
H	6.6806000000	2.9633000000	-2.2906000000
H	7.3967000000	1.3563000000	-2.5252000000
H	8.1885000000	2.8051000000	-0.4633000000
H	8.0265000000	1.0277000000	-0.3576000000
H	5.8316000000	2.9360000000	0.5597000000

H	6.9737000000	2.0964000000	1.6275000000
H	4.1924000000	2.9690000000	-0.7430000000
H	3.2008000000	2.0263000000	-1.8814000000
H	4.3615000000	3.1974000000	-2.5101000000

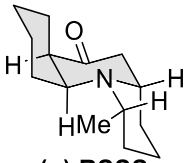

(n) RSSS

(n) RSSS - Geph-6Mem

C	2.6489999099	-0.0179128786	-0.7993792931
C	2.3377696412	-1.5189650841	-0.8880700284
C	0.9770169255	-1.7768428076	-0.3157044998
C	0.6521395677	-1.1934655797	1.0441089957
C	1.1781506035	0.2852544948	1.2098055900
N	2.5145205877	0.5037271864	0.5887720283
C	3.6856296194	0.3712898122	1.5123023331
C	4.9947063380	0.7685564220	0.8042962509
C	5.1750985110	0.0041121830	-0.5081243270
C	4.0060479331	0.3562010248	-1.4238308573
C	-0.8620454188	-1.2013262564	1.3136362256
C	-1.6392440428	-0.3634349349	0.2649911235
C	-0.8229437444	0.8206840530	-0.3058574013
C	0.1451524025	1.3656203250	0.7442563161
C	3.8271574627	-0.9993596880	2.2045711271
O	0.3718645758	-2.7674034819	-0.6996998748
H	1.1545999179	-1.8478334238	1.7811907214
H	1.2672468676	0.5042410427	2.2985150822
H	1.9013042167	0.4858473279	-1.4427769975
H	3.0776414866	-2.1411991900	-0.3612146834
H	2.3568964313	-1.8373964260	-1.9538056756

H	3.5548080103	1.0994724685	2.3434408442
H	5.8655117180	0.6087444941	1.4763262433
H	4.9578168251	1.8559259412	0.5696183685
H	6.1229414443	0.3273144509	-0.9903059310
H	5.2357031317	-1.0901243734	-0.3358089819
H	4.1204505564	-0.1208006023	-2.4215123978
H	4.0244963599	1.4579376679	-1.5827783459
H	-0.9948171564	-0.7362088543	2.3162646909
H	-1.2963303098	-2.2223669054	1.3748298577
H	-2.5664886655	-0.0109476936	0.7669031141
H	-1.9472518340	-0.9843958442	-0.6040094639
H	-1.5175718726	1.5969652730	-0.6916782172
H	-0.2309468843	0.4730140701	-1.1709504174
H	-0.4997408627	1.6540327652	1.6055935005
H	0.6696194902	2.2888949465	0.4165958095
H	2.9475655544	-1.2178212171	2.8368964306
H	4.6958256423	-0.9698350986	2.8963339626
H	4.0058412579	-1.8323735555	1.5086062779

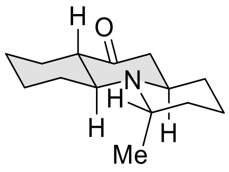

(g) SSRS

(g) SSRS

C	-1.8340000000	0.1926000000	1.2608000000
C	-1.6573000000	-1.2720000000	0.8296000000
C	-0.2775000000	-1.8018000000	1.2587000000
C	0.8263000000	-0.8929000000	0.6686000000
C	0.6620000000	0.5801000000	1.0892000000
C	-0.7319000000	1.0775000000	0.6504000000
N	2.2293000000	-1.3763000000	0.8306000000

C	3.0263000000	-0.6925000000	-0.2281000000
C	3.0658000000	0.8512000000	-0.0290000000
C	1.7838000000	1.4408000000	0.5327000000
C	2.9245000000	-1.2740000000	2.1579000000
C	4.2991000000	-1.9777000000	2.0501000000
C	5.1641000000	-1.3209000000	0.9727000000
C	4.4194000000	-1.3347000000	-0.3629000000
O	1.7385000000	2.6491000000	0.7096000000
C	2.1616000000	-1.9111000000	3.3244000000
H	2.5399000000	-0.8645000000	-1.2160000000
H	0.5927000000	-0.9406000000	-0.4223000000
H	0.7194000000	0.6620000000	2.1969000000
H	-1.7920000000	0.2597000000	2.3703000000
H	-2.8291000000	0.5586000000	0.9285000000
H	-1.7543000000	-1.3369000000	-0.2768000000
H	-2.4563000000	-1.8997000000	1.2796000000
H	-0.1541000000	-2.8403000000	0.8829000000
H	-0.2782000000	-1.8336000000	2.3563000000
H	-0.8108000000	1.0549000000	-0.4594000000
H	-0.8849000000	2.1260000000	0.9863000000
H	3.3417000000	1.3283000000	-0.9942000000
H	3.8422000000	1.1370000000	0.7062000000
H	3.0996000000	-0.2133000000	2.4399000000
H	4.1439000000	-3.0502000000	1.7959000000
H	4.8459000000	-1.9239000000	3.0171000000
H	5.4044000000	-0.2818000000	1.2765000000
H	6.1273000000	-1.8660000000	0.8732000000
H	4.2826000000	-2.3957000000	-0.6694000000
H	5.0165000000	-0.8248000000	-1.1495000000
H	1.8837000000	-2.9619000000	3.0948000000
H	1.2814000000	-1.3026000000	3.5951000000

H	2.7912000000	-1.9031000000	4.2401000000

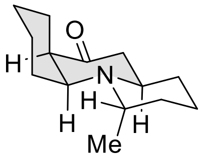

(h) RSRS

(h) RSRS

C	2.8161211422	1.1287868919	0.6076854662
C	3.2910945564	-0.0955975143	-0.1734921204
C	2.1826053321	-0.6191107004	-1.1004945906
C	0.9128226918	-1.0416827356	-0.2745119720
C	0.4328032323	0.2148870326	0.5182009249
C	1.5556827475	0.7872153257	1.4138159183
N	-0.2154277289	-1.6537009901	-1.0880069176
C	-1.4327955221	-0.7808065640	-1.2154937633
C	-1.0722926331	0.6790081219	-1.5448006474
C	-0.1147078129	1.1965232128	-0.5079122902
C	0.2157142885	-2.4011056596	-2.3125849324
C	-0.9603975452	-2.9040921857	-3.1754106447
C	-1.9463936669	-1.7875103972	-3.4739021052
C	-2.5221942105	-1.3443960228	-2.1422987546
O	0.2583965766	2.3587825285	-0.5580939232
C	1.0379912813	-3.6511041657	-1.9236121580
H	-1.9365011069	-0.7399012122	-0.2241041889
H	1.2411998540	-1.8121067803	0.4574041231
H	-0.3960010029	-0.0831993103	1.1954016183
H	2.6151954684	1.9540047916	-0.1051970946
H	3.6185974528	1.4716991593	1.2954989678
H	3.5865989896	-0.9001960114	0.5357942102
H	4.1844976573	0.1800023988	-0.7746031392
H	2.6332977626	-1.4580957233	-1.6568993194

H	1.9238944322	0.1610077007	-1.8485051181
H	1.8233048628	0.0187947183	2.1725012318
H	1.1957031439	1.6876889797	1.9568968921
H	-1.9950989220	1.2985983599	-1.5225980462
H	-0.6049012834	0.7595011992	-2.5488958836
H	0.8024975837	-1.7226005553	-2.9676024242
H	-1.5049050442	-3.7209043265	-2.6484950774
H	-0.5701034829	-3.3233014782	-4.1289952542
H	-1.4580979663	-0.9468980106	-4.0121010484
H	-2.7657001093	-2.1858999071	-4.1106015904
H	-2.9849029014	-2.2330005983	-1.6555999245
H	-3.3334012502	-0.5975955716	-2.2844020773
H	0.4116014584	-4.3455973276	-1.3233976612
H	1.9298040467	-3.4284979336	-1.3115960292
H	1.3830976280	-4.1832987392	-2.8359906564

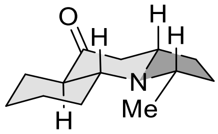

(i) RRSS

(i) RRSS - Geph-6Mem

C	-0.0261045148	-0.6277346179	-0.1163794872
C	-0.3074400433	0.9041626875	-0.2228261252
N	-1.7664965982	1.0333144479	-0.4458446446
C	-2.5076651744	0.6119228383	0.7623677743
C	-2.4284829212	-0.9135230053	0.8255295098
C	-0.9517898864	-1.2266515902	0.9329048444
C	1.4664703028	-0.8901696655	0.1609665725
C	2.3249580627	-0.2653662548	-0.9469232315
C	2.0537557215	1.2412690874	-1.0516808081
C	0.5670129907	1.5068535445	-1.3365479576
C	-2.5022367098	2.1777897683	-0.9958420905

C	-3.9510458420	1.7348142143	-0.7623927126
C	-3.8943890599	1.2557469499	0.6678424803
C	-2.2879199310	2.4084257586	-2.4992889500
O	-0.5205896001	-1.8614823292	1.8843571336
H	-0.2952879213	-1.0979131604	-1.0896890042
H	-0.0259996228	1.4133524810	0.7288214520
H	-2.0510911540	1.0038718534	1.7043432734
H	-2.8491752605	-1.3563620774	-0.1031631750
H	-2.9845089404	-1.3088668661	1.7031555266
H	1.6608467708	-1.9839536108	0.2006844831
H	1.7495919883	-0.4458535317	1.1410669675
H	2.0867989065	-0.7510972381	-1.9190389775
H	3.4004289568	-0.4377031813	-0.7274788564
H	2.6717896628	1.6743380209	-1.8672218164
H	2.3433519218	1.7356558512	-0.0979149100
H	0.2998232252	1.0538312070	-2.3165798854
H	0.4206225747	2.6060781306	-1.3746346136
H	-2.2722661397	3.1009053022	-0.4154807864
H	-4.1688004070	0.8513028695	-1.4064681560
H	-4.7191478959	2.5160677441	-0.9484191826
H	-4.7475471737	0.6446559779	1.0314473283
H	-3.8659557576	2.1883100738	1.2785667221
H	-2.4862495694	1.4763329114	-3.0710121302
H	-1.2726818465	2.7629187877	-2.7397591051
H	-2.9845284544	3.1976658293	-2.8537934201

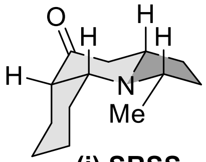

(j) SRSS

(j) SRSS - Geph-6Mem

C	1.4866239403	0.4311651320	0.8665116296

C	1.2834685863	-0.8098124250	-0.0684254592
N	1.5027788286	-0.3913234660	-1.4790695757
C	2.8784193605	0.1104487907	-1.6627271081
C	3.0889138289	1.4201991014	-0.9088353022
C	2.8087364822	1.0842370522	0.5331707686
C	0.3428046930	1.4552931685	0.6937475028
C	-1.0068921839	0.8034510767	0.9998962145
C	-1.2420372551	-0.3671504072	0.0438783159
C	-0.1220746751	-1.4110702413	0.1852155519
C	1.3262171373	-1.3943504614	-2.5609623296
C	2.5732678133	-1.2731290437	-3.4609942247
C	3.0643447982	0.1328850931	-3.1645225981
C	0.0688130632	-1.0586271004	-3.3708553953
O	3.6668793045	1.2358942317	1.3891011438
H	1.4822751877	0.1031153523	1.9308593479
H	2.0352035134	-1.5923523898	0.1859307227
H	3.6155871028	-0.6254865130	-1.2471149461
H	2.3856905514	2.1955492953	-1.2777281474
H	4.1287378920	1.7910454295	-1.0389815231
H	0.3143489202	1.8502365506	-0.3447235111
H	0.5000083738	2.3114542755	1.3855048829
H	-1.8191929547	1.5518242598	0.8769849886
H	-1.0222267372	0.4428688480	2.0521694191
H	-1.2820266735	0.0281206034	-0.9897775583
H	-2.2207740918	-0.8410246320	0.2720006874
H	-0.1512231239	-1.8025117603	1.2262751424
H	-0.3144164102	-2.2704672584	-0.4888801732
H	1.2378254800	-2.4412448609	-2.1954872628
H	2.4270198245	-1.5293423264	-4.5323923569
H	3.3475623902	-1.9731215492	-3.0675943420
H	2.3817182606	0.8850104037	-3.6191564653

H	4.1075324899	0.3220161846	-3.4961735016
H	0.1044691714	-0.0165007361	-3.7532586708
H	-0.8402561163	-1.1899993396	-2.7566649178
H	-0.0218169538	-1.7532801391	-4.2329435588

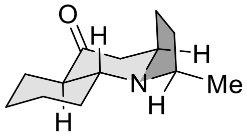

(k) RRRS

(k) RRRS - Geph-6Mem

C	2.8314824951	-1.8887565795	0.6716770196
C	-0.7528134634	-6.1680915832	-1.7355521107
C	-0.8143368210	-5.2672081155	-0.4959397188
C	0.4950547849	-4.4836440530	-0.3389962861
C	0.7842986337	-3.6252960404	-1.5864278688
C	0.7997564614	-4.5181137679	-2.8550597863
C	-0.5119318133	-5.3212465178	-2.9894732229
N	2.0939663114	-2.9410271256	-1.4173468368
C	2.9307669182	-2.7398735809	-2.6250092584
C	2.0922518163	-2.5384954873	-3.9145132012
C	1.0287544802	-3.5921352762	-4.0274784342
C	2.0886514305	-1.6741057007	-0.6551332873
C	2.8135982109	-0.6544405874	-1.5446096071
C	3.8000362690	-1.5280299813	-2.2990552075
O	0.2826973257	-3.5921858457	-4.9961861815
H	1.6519982363	-5.2300677377	-2.7743312556
H	-0.0323801754	-2.8750928878	-1.7073903059
H	3.5633813896	-3.6388143394	-2.7970610100
H	1.0689429737	-1.3035682732	-0.4061564229
H	3.8644697243	-2.2603646410	0.5136720557
H	2.2940655045	-2.6312479435	1.2956805126
H	2.8787025251	-0.9340623343	1.2374764032

H	-1.7094385415	-6.7236111328	-1.8401057135
H	0.0693572739	-6.9085445363	-1.6202297350
H	-0.9841009358	-5.8904913116	0.4081646764
H	-1.6643554756	-4.5561795052	-0.5929235666
H	1.3305849238	-5.2020458351	-0.1839753726
H	0.4197813696	-3.8361168623	0.5591835797
H	-0.4632598312	-5.9833888380	-3.8810846697
H	-1.3692013763	-4.6226717926	-3.1173347573
H	2.7705796457	-2.5647521861	-4.7943600902
H	1.5594652618	-1.5670911089	-3.9169467291
H	3.2510520884	0.2140985731	-1.0071223765
H	2.0833416621	-0.2480443343	-2.2743534670
H	4.2934914728	-1.0530154569	-3.1737449692
H	4.5932735187	-1.8436681959	-1.5876460506

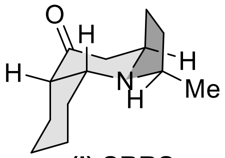

(l) SRRS

(l) SRRS - Geph-6Mem

C	0.5857601969	2.1264734317	-0.6169605617
C	2.6884506719	6.8872570672	-1.5120103028
C	1.5768888582	6.0842238445	-0.8174291380
C	2.1457178968	5.3218893083	0.3914875621
C	3.2740337574	4.3635665547	-0.0441868089
C	4.3817586082	5.1770495274	-0.7612439354
C	3.8221948844	5.9585134900	-1.9700922402
N	2.7282794765	3.2867081465	-0.9154129880
C	3.6726436511	2.6485125854	-1.8717297389
C	5.1581781447	2.7970970655	-1.4608773961
C	5.4921835659	4.2283464233	-1.1369325820

C	2.0561692251	2.1918558808	-0.1779422236
C	2.8250282255	0.9149778875	-0.5346229590
C	3.2389371338	1.1871629339	-1.9639403393
O	6.6473358284	4.5161306112	-0.8620676013
H	4.7901181955	5.9287431128	-0.0465931859
H	3.7298659999	3.9066251713	0.8651906778
H	3.5784830086	3.1125988120	-2.8750026676
H	2.0503552859	2.3263192530	0.9277840503
H	0.4792592420	1.9967365334	-1.7127127655
H	0.0673271337	3.0668304013	-0.3356343609
H	0.0747217962	1.2833702754	-0.1053037781
H	3.0937092465	7.6411574989	-0.8014940604
H	2.2712093902	7.4282848149	-2.3883674618
H	1.1261691075	5.3670713519	-1.5381291122
H	0.7786472128	6.7779190107	-0.4767671485
H	1.3366715654	4.7604157016	0.9038699528
H	2.5520716922	6.0585611507	1.1194694491
H	3.4214166828	5.2790867285	-2.7456539274
H	4.6302742932	6.5629709283	-2.4361423184
H	5.7916359872	2.4342652152	-2.2991029861
H	5.4046817768	2.1991199786	-0.5598715407
H	2.2681436711	-0.0318174791	-0.3677794801
H	3.7438123478	0.8748032391	0.0886154127
H	2.3317521838	1.1238732422	-2.6022783114
H	3.9981257956	0.4959681671	-2.3887412910

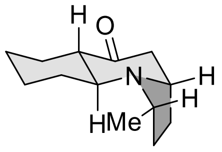

(e) SSSS

(e) SSSS

C	1.5369000000	0.0201000000	0.1472000000
C	0.0949000000	0.5054000000	-0.1368000000
N	-0.9692000000	-0.4069000000	0.4046000000
C	-0.7095000000	-1.8757000000	0.3105000000
C	0.7548000000	-2.3349000000	0.3957000000
C	1.6393000000	-1.3935000000	-0.3257000000
C	2.5618000000	0.9663000000	-0.5123000000
C	2.3988000000	2.3803000000	0.0603000000
C	0.9679000000	2.8872000000	-0.1565000000
C	-0.0532000000	1.9237000000	0.4701000000
C	-2.3292000000	-0.2406000000	-0.2405000000
C	-2.8075000000	-1.6572000000	-0.5907000000
C	-1.4949000000	-2.3516000000	-0.8963000000
H	1.7307000000	0.0190000000	1.2447000000
H	0.0303000000	0.5486000000	-1.2422000000
O	2.4031000000	-1.7595000000	-1.2068000000
H	-3.0365000000	0.2391000000	0.4701000000
H	-1.1926000000	-2.3689000000	1.1893000000
C	-2.4093000000	0.5941000000	-1.5529000000
H	0.8677000000	-3.3919000000	0.0704000000
H	1.0701000000	-2.2803000000	1.4615000000
H	2.4093000000	0.9889000000	-1.6145000000
H	3.5953000000	0.6106000000	-0.3090000000
H	3.1209000000	3.0703000000	-0.4266000000
H	2.6184000000	2.3622000000	1.1508000000
H	0.7669000000	2.9739000000	-1.2472000000
H	0.8598000000	3.8957000000	0.2972000000
H	-1.0661000000	2.3381000000	0.3137000000
H	0.1020000000	1.8729000000	1.5706000000
H	-3.5851000000	-1.7260000000	-1.3825000000
H	-3.2419000000	-2.1262000000	0.3234000000

H	-1.5919000000	-3.4566000000	-0.9601000000
H	-1.0379000000	-1.9645000000	-1.8333000000
H	-1.8031000000	0.1565000000	-2.3731000000
H	-2.1199000000	1.6501000000	-1.4147000000
H	-3.4619000000	0.6165000000	-1.9086000000

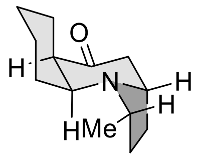

(f) RSSS

(f) RSSS

C	1.4903000000	-0.9682000000	0.4867000000
H	1.7267000000	-0.9492000000	1.5734000000
N	-0.3874000000	0.6699000000	0.6701000000
C	0.3128000000	1.6527000000	-0.1524000000
C	1.8041000000	1.5976000000	0.1612000000
C	2.2351000000	0.1959000000	-0.1679000000
C	-1.7837000000	1.1134000000	0.8125000000
C	-1.8397000000	2.4711000000	0.0883000000
C	-0.3979000000	2.9554000000	0.1766000000
C	1.9316000000	-2.3295000000	-0.1490000000
C	0.8047000000	-3.3490000000	-0.0437000000
C	-0.4160000000	-2.8700000000	-0.8818000000
C	-0.6047000000	-1.3255000000	-0.8941000000
C	-0.0708000000	-0.7506000000	0.4229000000
O	3.1028000000	0.0153000000	-1.0104000000
H	-2.5279000000	0.4138000000	0.3757000000
C	-2.0953000000	1.2573000000	2.3049000000
H	-0.5616000000	-1.3387000000	1.2315000000
H	0.1502000000	1.4295000000	-1.2346000000
H	1.9782000000	1.7975000000	1.2407000000

H	2.3626000000	2.3456000000	-0.4427000000
H	-2.6209000000	3.1743000000	0.4484000000
H	-2.0416000000	2.2732000000	-0.9902000000
H	-0.1527000000	3.7964000000	-0.5062000000
H	-0.1728000000	3.2541000000	1.2247000000
H	2.1150000000	-2.2272000000	-1.2422000000
H	2.8776000000	-2.6721000000	0.3221000000
H	0.5351000000	-3.4195000000	1.0335000000
H	1.1069000000	-4.3640000000	-0.3791000000
H	-1.3051000000	-3.3772000000	-0.4483000000
H	-0.3317000000	-3.1891000000	-1.9431000000
H	-1.6721000000	-1.1025000000	-1.0968000000
H	-0.0060000000	-0.8762000000	-1.7163000000
H	-1.3876000000	1.9547000000	2.8006000000
H	-2.0186000000	0.2664000000	2.8005000000
H	-3.1296000000	1.6385000000	2.4417000000

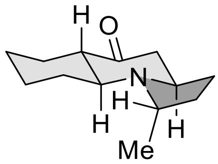

(o) SSRS

(o) SSRS - Geph-6Mem

C	-1.9049017287	-0.1239024995	0.6682743469
C	-1.5010007748	-1.5566145807	0.2954185730
C	-0.0386926488	-1.8244727633	0.6806826295
C	0.8972404800	-0.8048546545	-0.0042572643
C	0.4790463325	0.6423159622	0.3440228382
C	-0.9945680141	0.8858201604	-0.0431984019
N	2.3369639804	-1.0515960283	0.2788566564
C	3.2811109573	-0.0879079858	-0.3195963764
C	2.8748733969	1.3880891695	-0.0369183564

C	1.4329220345	1.5644025904	-0.3694410762
C	2.8280177434	-1.3853020534	1.6356541067
C	4.1845644644	-0.6689768133	1.7809171283
C	4.6099044350	-0.4555472317	0.3343909893
O	1.0651854108	2.3932449297	-1.1892830637
C	3.0253690713	-2.9040992434	1.7323466899
H	0.5876629916	0.8034870913	1.4402726636
H	3.3196286321	-0.2226166470	-1.4232364053
H	0.7634420872	-0.9335129305	-1.1037180430
H	-1.8254802635	0.0120695293	1.7694794850
H	-2.9606664034	0.0538698405	0.3709829125
H	-1.6279953781	-1.7007683261	-0.8003804980
H	-2.1622022066	-2.2806765701	0.8181489805
H	0.2272412135	-2.8546423332	0.3633111626
H	0.0537049373	-1.7531403668	1.7852258127
H	-1.1215648124	0.7740990503	-1.1434012837
H	-1.2948775747	1.9179070957	0.2403062539
H	3.5272318397	2.0853085655	-0.6060626748
H	2.9533413264	1.6565045919	1.0318285688
H	2.1444914718	-1.0602883016	2.4497872979
H	4.9281369313	-1.1740177175	2.4345531434
H	4.0113634602	0.3216895840	2.2427128791
H	4.9338771411	-1.4383723544	-0.0748468625
H	5.4314024411	0.2773125077	0.1858992408
H	3.7144902207	-3.2686969026	0.9412552501
H	2.0602452738	-3.4361220753	1.6280840670
H	3.4493686066	-3.1683521920	2.7244240051

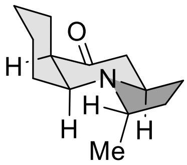

(p) RSRS

(p) RSRS - Geph-6Mem

C	2.6075664079	0.6779215150	0.7362971070
C	2.9072703910	-0.5679230577	-0.1020914917
C	1.7487925122	-0.8762156229	-1.0638758413
C	0.4283373332	-1.1011808915	-0.2778875907
C	0.1174695253	0.1457327923	0.6038504469
C	1.3066243673	0.4819706769	1.5307767134
N	-0.7455233147	-1.4113992543	-1.1476145202
C	-1.8537602871	-0.4413431138	-1.2447248701
C	-1.3129979162	0.9981335405	-1.3970601424
C	-0.2991839586	1.2805059565	-0.3195533515
C	-0.6458895371	-2.1530641672	-2.4207129420
C	-1.6073524293	-1.4275672303	-3.3732727624
C	-2.6846852548	-0.9201738563	-2.4334456467
O	0.2232337011	2.3835237695	-0.2601784007
C	-1.0503721073	-3.6106532991	-2.1754113517
H	-0.7586642783	-0.0902584101	1.2468506414
H	0.5939556654	-1.9733980079	0.3924778858
H	-2.4744284988	-0.4624614265	-0.3214164720
H	2.5292083169	1.5579483867	0.0652271662
H	3.4470554090	0.8651519378	1.4397238566
H	3.0648700782	-1.4372123955	0.5742894266
H	3.8418800330	-0.4083265275	-0.6817415819
H	2.0356300334	-1.7918058456	-1.6191517939
H	1.6298378896	-0.0398789171	-1.7866882953
H	1.4536500090	-0.3655244100	2.2363904311
H	1.0825632287	1.3881560760	2.1339244583

H	-2.1603196885	1.7144839215	-1.3383572695
H	-0.7962760496	1.1405768152	-2.3678043593
H	0.3578712861	-2.1599074856	-2.8848326038
H	-1.9626416656	-2.0314580193	-4.2355289629
H	-1.0809200206	-0.5410034089	-3.7867850526
H	-3.2853692770	-1.7976059223	-2.1092695725
H	-3.3812365360	-0.1650558992	-2.8559637980
H	-2.0622526976	-3.6884395913	-1.7271794037
H	-0.3252541663	-4.0837059277	-1.4797410296
H	-1.0380612070	-4.1746067993	-3.1321808011

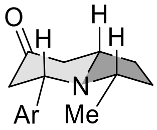

(q) SSS

(q) SSS - Dimeth-5Mem

C	-1.6902342120	0.2412633273	-1.3233032302
C	-0.8601941898	-0.9339670926	-0.7342494974
N	0.4492449820	-0.3999200146	-0.2880454930
C	0.2769360716	0.6052835247	0.7736461054
C	-0.4007699558	1.8538410664	0.2141639679
C	-1.7201329542	1.3724981011	-0.3293235580
C	1.4788920676	-1.3355997141	0.2198286694
C	1.9900405499	-0.7175372023	1.5367399813
C	1.6647788346	0.7583305389	1.3601874551
O	-2.7771887692	1.8245949128	0.0827849565
C	-0.7025929616	-2.0290314341	-1.7739928457
C	-1.2158199466	-3.3099477973	-1.5256691563
C	-1.0815622443	-4.3243080277	-2.4742450894
C	-0.4260920675	-4.0787764330	-3.6967973985
C	0.0948994464	-2.8007982051	-3.9570204812
C	-0.0490204647	-1.7817613097	-2.9935459467

O	-0.2758936507	-5.0415415333	-4.6448200441
O	0.7278143763	-2.5924069787	-5.1422323479
C	1.3082198324	-1.4111426101	-5.5551091252
C	-0.7294804129	-6.3412051580	-4.5586210779
C	2.6232471731	-1.4195449268	-0.7985442883
H	-0.3838520976	0.2008478008	1.5834617478
H	-1.4059183320	-1.3394567344	0.1495140460
H	-2.7168914674	-0.0905522585	-1.5922913017
H	-1.1963544127	0.6314783950	-2.2378811517
H	0.2115568892	2.2841521247	-0.6073730161
H	-0.5449332440	2.6210398208	1.0053376244
H	1.0969016269	-2.3650235182	0.4012996260
H	3.0389033760	-0.9617399802	1.8104633628
H	1.3436222592	-1.0927817177	2.3642162548
H	2.3452662982	1.2148546368	0.6076778923
H	1.7025815781	1.3447244131	2.3027113466
H	-1.7244181556	-3.5278974614	-0.5947192224
H	-1.4921985615	-5.2983861324	-2.2453972131
H	0.3523056470	-0.7957567161	-3.1824591875
H	1.7445378337	-1.5557722810	-6.5645439819
H	2.1211339994	-1.1185493008	-4.8569624281
H	0.5444778781	-0.6067078440	-5.6118319807
H	-0.4564870428	-6.8820100451	-5.4878021994
H	-1.8351417147	-6.3554372387	-4.4529499839
H	-0.2560025257	-6.8570923920	-3.6965808299
H	3.0427640414	-0.4149869228	-1.0163160520
H	2.2734275306	-1.8674643720	-1.7473888813
H	3.4342980119	-2.0672857429	-0.4031388554

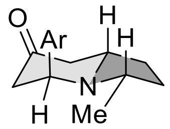

(r) RSS

(r) RSS - Dimeth-5Mem

C	-2.2874024305	-0.3042717372	-0.6992641693
C	-1.0051281764	-1.0367604689	-0.2318857688
N	0.1237223696	-0.0696974627	-0.2594328474
C	-0.0602624349	1.1449981154	0.5451728969
C	-1.2714600095	1.9412470126	0.0516967070
C	-2.4435817529	0.9929331578	0.0537552472
C	1.4752748825	-0.5015031645	0.0977143771
C	2.2437250353	0.8077483252	-0.0540933811
C	1.2978471460	1.8633795152	0.5297419214
O	-3.4589157853	1.2404944353	0.6859008652
C	-1.1815564180	-1.8071989690	1.0876612924
C	-0.8916595699	-3.1839933636	1.0988669548
C	-1.0423613265	-3.9450433196	2.2575172317
C	-1.4989674604	-3.3520066236	3.4473232812
C	-1.8063576495	-1.9840665877	3.4599048208
C	-1.6489105895	-1.2216285566	2.2831324739
O	-1.6632813235	-4.0618197934	4.5954490201
O	-2.2575889919	-1.4397471563	4.6219858925
C	-2.6144787275	-0.1217408135	4.8194150942
C	-1.4255353324	-5.4112698864	4.7553432331
C	2.0258516601	-1.5988930137	-0.8182702027
H	-0.2132118573	0.8490565643	1.6012037126
H	-0.7981850431	-1.7933788396	-1.0195758686
H	-3.1817323087	-0.9601239080	-0.6218316365
H	-2.1551026993	-0.0274584101	-1.7680357682
H	-1.0883187976	2.2792867445	-0.9910262262

H	-1.4577326996	2.8291218285	0.6935439189
H	1.5060825244	-0.8431806382	1.1593653883
H	2.2841640104	1.0087043007	-1.1505059704
H	3.2850071202	0.8348756013	0.3308104656
H	1.3368067520	2.7773916260	-0.1027161613
H	1.5670850514	2.1257338258	1.5770877308
H	-0.5413724236	-3.6823200290	0.2038660279
H	-0.8022357207	-4.9988707725	2.2144534755
H	-1.9130037560	-0.1821099024	2.3127018046
H	-2.9402047763	0.0112675202	5.8713807723
H	-3.4586149436	0.1519932390	4.1511392275
H	-1.7453363288	0.5441356294	4.6311432867
H	-1.6528797677	-5.6973894933	5.8025791941
H	-0.3584296155	-5.6407890442	4.5491056925
H	-2.0811506190	-5.9991790318	4.0780086676
H	1.9834205335	-1.2815977026	-1.8821657428
H	1.4410641653	-2.5327448541	-0.7015945538
H	3.0806080851	-1.8181838989	-0.5484723775

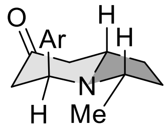

(r) RSS

(s) SRS – Dimeth-5Mem

C	-1.4923379848	-0.8070191073	0.8234676177
C	-0.2662233917	-1.5377550092	0.2242180379
N	0.7696561206	-0.5301224541	-0.1062818260
C	0.4129428697	0.6399971922	-0.9120303799
C	-0.7591522973	1.3958102845	-0.2767416760
C	-1.8507771973	0.3911989097	-0.0184464580
C	2.2109123716	-0.7914177450	-0.2675193545
C	2.6784402147	0.2658720941	-1.2812545472

C	1.7200861682	1.4155476495	-1.0033369942
O	-2.9674979449	0.5254649675	-0.4941512436
C	-0.6368958739	-2.4552236946	-0.9469406298
C	-1.2689095588	-1.9964936295	-2.1178058917
C	-1.5903291170	-2.8771841145	-3.1510179224
C	-1.2934947372	-4.2476399457	-3.0442373372
C	-0.6733585547	-4.7283580068	-1.8823741803
C	-0.3540924928	-3.8315752987	-0.8424103125
O	-1.5919439307	-5.1338036605	-4.0313657526
O	-0.4013315440	-6.0583860394	-1.8023097430
C	0.2039899865	-6.7014080721	-0.7424998803
C	-2.2020841503	-4.8343238996	-5.2318117204
C	2.5792804295	-2.2104313561	-0.7075626193
H	0.1457345843	0.3120372658	-1.9369531615
H	0.1334849276	-2.1765073942	1.0422746777
H	-2.3453960312	-1.5043885376	0.9720200176
H	-1.2017973065	-0.4057715048	1.8190726933
H	-1.0969513977	2.2286466096	-0.9303611277
H	-0.4443586260	1.8141823327	0.7039397943
H	2.6841140647	-0.5764321093	0.7160202560
H	2.4613681033	-0.0915501530	-2.3151774976
H	3.7592913082	0.5138596184	-1.2193434722
H	1.7242073636	2.2183519809	-1.7709889591
H	1.9684238212	1.8521117091	-0.0089233808
H	-1.5371547052	-0.9640783541	-2.2539305955
H	-2.0755765759	-2.4785655246	-4.0318151203
H	0.1214199797	-4.2038494854	0.0545894661
H	0.2880435751	-7.7826348201	-0.9753645414
H	-0.4072622527	-6.5845203114	0.1776869134
H	1.2246234047	-6.2943464283	-0.5786692275
H	-2.3098685638	-5.7651930660	-5.8253205802

H	-1.5817984636	-4.1161245297	-5.8092655438
H	-3.2129115810	-4.4095430659	-5.0533584680
H	2.1091266057	-2.4539836342	-1.6836314445
H	2.2535703102	-2.9460588546	0.0554350362
H	3.6814880697	-2.2943908079	-0.8139229210

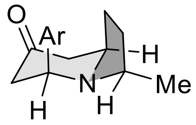

(t) RRS

(t) RRS – Dimeth-5Mem

C	-2.0660104916	-1.0508107366	-0.3468314530
C	-0.6743370515	-1.6438876143	0.0105194770
N	0.3698670250	-0.6841034631	-0.4419396328
C	0.1949139825	0.7645866970	-0.2078823998
C	-1.0033814300	1.0870240122	0.6973142933
C	-2.1909335705	0.3932784075	0.0895366655
C	1.8067160269	-0.9937487850	-0.3829915868
C	2.4797589811	0.3742981988	-0.5474896414
C	1.5571184750	1.2635946338	0.2729662155
O	-3.2050607912	1.0226670699	-0.1689636061
C	-0.6315345205	-2.0900344468	1.4843865147
C	0.2099535830	-1.5473926215	2.4728512204
C	0.1846027473	-2.0230735119	3.7843340700
C	-0.6819203284	-3.0666725985	4.1522935433
C	-1.5261433476	-3.6296198563	3.1862419228
C	-1.4948694294	-3.1382934080	1.8652715931
O	-0.7317497090	-3.5566343675	5.4196637732
O	-2.3508540789	-4.6426507392	3.5645742736
C	-3.2341412463	-5.3189895329	2.7487658576
C	0.0254594586	-3.1206618480	6.4871824946
C	2.2305821431	-1.9887690732	-1.4621293319
H	0.0057851492	1.2174174201	-1.2090704414

H	-0.5424313515	-2.5790599024	-0.5756890699
H	-2.8947358880	-1.6657803610	0.0606742752
H	-2.1665829924	-1.0456418902	-1.4540015081
H	-1.1670547066	2.1828498256	0.7864373648
H	-0.8388508032	0.6932717333	1.7152389416
H	2.0771516639	-1.4073102960	0.6068485045
H	2.4010071842	0.6999184677	-1.6109274048
H	3.5485296302	0.3988961520	-0.2460077888
H	1.7135739809	2.3549908090	0.1391942763
H	1.7086438679	1.0219885396	1.3437826009
H	0.8874664039	-0.7468848084	2.2650732926
H	0.8478133703	-1.5678407068	4.5076652999
H	-2.1410042272	-3.5789196817	1.1181198601
H	-3.7621223272	-6.0894559373	3.3470183129
H	-2.6857612803	-5.8253169476	1.9258723626
H	-3.9873234724	-4.6169291021	2.3318778861
H	-0.2405320566	-3.7138743404	7.3859262723
H	-0.1845514876	-2.0498148619	6.6949786236
H	1.1064511847	-3.2644378269	6.2754922632
H	1.9697380470	-1.6034469978	-2.4708239991
H	1.7190425650	-2.9614082823	-1.3073153198
H	3.3270417913	-2.1577274921	-1.4108239566

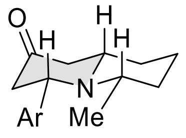

(u) SSS

(u) SSS – Dimeth-6Mem

C	2.2447398932	0.8478851198	-0.9540325221
C	1.0183020943	1.4327085398	-0.2123055669
N	-0.1531016481	0.5095566831	-0.3323060074
C	0.1702672083	-0.8504739902	0.2224851178

C	1.3279195207	-1.4858115808	-0.5611715099
C	2.4821357130	-0.5438317052	-0.4736887764
O	3.5490428789	-0.8798719209	0.0169537870
C	0.7315798626	2.8158132896	-0.7822338380
C	0.8375321476	3.9475821907	0.0390679870
C	0.5755716208	5.2201873355	-0.4690367679
C	0.1983602675	5.3895220484	-1.8156892106
C	0.0867422718	4.2638922512	-2.6488393577
C	0.3559693468	2.9822720311	-2.1262402788
O	-0.0660512549	6.6136443222	-2.3452830721
O	-0.2834520411	4.4544578749	-3.9433229197
C	-0.4509385162	3.4674510098	-4.8926452626
C	-0.0050905440	7.8173620399	-1.6739162008
H	0.4961865892	-0.7728038342	1.2864027427
H	1.2826242661	1.5242158795	0.8670254521
C	-1.4533257984	1.0848137467	0.1581059820
C	-2.0189267880	0.3065931530	1.3591797063
C	-2.1996155055	-1.1907405460	1.0548117864
C	-1.0746633674	-1.7454743480	0.1603058524
C	-2.4669443469	1.1406106466	-0.9930026077
H	3.1429559938	1.4849224757	-0.7968801165
H	2.0419483419	0.7875935580	-2.0443913220
H	1.0423280133	-1.6160484276	-1.6279420461
H	1.5953875910	-2.4781641030	-0.1359224193
H	1.1241850313	3.8475194161	1.0785638710
H	0.6688746845	6.0675563937	0.1966872671
H	0.2686551911	2.1084560271	-2.7571323951
H	-0.7566360696	3.9348765078	-5.8510068821
H	-1.2464069416	2.7599366733	-4.5747917252
H	0.5036713092	2.9227189485	-5.0536585508
H	-0.2744474517	8.6357639348	-2.3725524523

H	1.0252648962	7.9968715858	-1.2995639247
H	-0.7255806739	7.8228469410	-0.8284005089
H	-1.3244338436	2.1295601939	0.5141508652
H	-2.9621635712	0.7406375424	1.7557525306
H	-1.2608489838	0.4083551947	2.1673019435
H	-3.1679414117	-1.4057240913	0.5532402604
H	-2.2126105673	-1.7055826175	2.0402563365
H	-1.4051613760	-1.7340097246	-0.9012884894
H	-0.8796200405	-2.8058342208	0.4312121887
H	-2.6828506942	0.1330986129	-1.4004334659
H	-2.0778099949	1.7681001008	-1.8192613851
H	-3.4195210312	1.5905637153	-0.6407090671

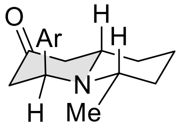

(v) RSS

(v) RSS – Dimeth-6Mem

C	2.3127803377	0.9708949731	-0.4631630238
C	1.1348305541	1.5475551977	0.3528486000
N	-0.1083486772	0.7354388113	0.2075250083
C	0.0106902902	-0.7579475723	0.2670058060
C	1.1917151456	-1.3021729790	-0.5585212575
C	2.4123390127	-0.5020618119	-0.2424781524
O	3.4302998795	-1.0215733655	0.1885332046
C	1.4782519403	1.7626025783	1.8284815079
C	1.4017251284	3.0632165293	2.3586945790
C	1.7023787101	3.3135189675	3.6972786607
C	2.0938698115	2.2657783525	4.5497542681
C	2.1825256037	0.9614290272	4.0414819055
C	1.8753871586	0.7179006530	2.6863348861
O	2.3946199734	2.4705662859	5.8598719903

O	2.5683634338	-0.0317710672	4.8869019621
C	2.7101854271	-1.3656238732	4.5638185388
C	2.3592807606	3.6815903021	6.5195526451
H	0.1801071562	-1.0041378631	1.3336585316
H	0.9568580086	2.5606463697	-0.0726050093
C	-1.1887446188	1.3013077307	-0.6297456875
C	-2.5632316249	0.6866806627	-0.2312702430
C	-2.4654265595	-0.6940370697	0.4517499587
C	-1.3033103873	-1.4840415121	-0.1251014826
C	-0.8815122494	1.1486424101	-2.1322994226
H	3.2585747008	1.5049842080	-0.2250442073
H	2.1118269646	1.1118988501	-1.5451181779
H	0.9843981417	-1.1800894245	-1.6420028956
H	1.3475752811	-2.3837139358	-0.3529304954
H	1.1030541920	3.8974647241	1.7359491600
H	1.6262230659	4.3300717114	4.0589904598
H	1.9591333366	-0.2845071472	2.3126079427
H	3.0360484113	-1.9237722312	5.4651984648
H	3.4808885596	-1.4897280518	3.7733360845
H	1.7394735406	-1.7832011797	4.2208046917
H	2.6556128215	3.5265933561	7.5771306918
H	1.3306996582	4.1006611908	6.4980380949
H	3.0724279667	4.3941256810	6.0529772090
H	-1.2784776387	2.3987551440	-0.4663349032
H	-3.2044067561	0.6396747253	-1.1384850654
H	-3.0941517651	1.3434317612	0.4922029314
H	-3.4374959312	-1.2233361801	0.3558971607
H	-2.2602323915	-0.5640915553	1.5385817556
H	-1.4508914591	-1.5588604606	-1.2208281078
H	-1.2760368080	-2.5221346549	0.2719014928
H	-0.7876546687	0.0978804265	-2.4552943274

H	0.0574619493	1.6784869678	-2.3870675896
H	-1.6923912770	1.6164228686	-2.7301783159

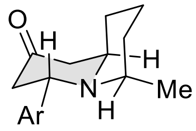

(w) SRS

(w) SRS

C	-2.2764141922	1.0483007945	-0.8761360319
C	-0.9277313962	1.7013789992	-0.5224652783
N	0.1412067459	0.6610869212	-0.3565765023
C	-0.1631948035	-0.4926206993	0.5459328682
C	-1.5531245622	-1.1010426105	0.2350692565
C	-2.5742398551	-0.0231693176	0.1172696667
O	-3.5864429583	-0.0107807963	0.8008635833
C	-1.0906543399	2.6838443884	0.6451459899
C	-0.8685056607	4.0528149244	0.4040240578
C	-1.0038607264	4.9944334159	1.4234103999
C	-1.3720986182	4.5952434068	2.7205147501
C	-1.6068903440	3.2374848818	2.9826139600
C	-1.4674605992	2.2915891796	1.9455772242
O	-1.5129230811	5.4836914840	3.7400918327
O	-1.9662978631	2.8807865879	4.2450049669
C	-2.2348372313	1.6003377303	4.6831234757
C	-1.3201224838	6.8472498738	3.6589922185
H	-0.1258683855	-0.1527841728	1.5989119598
H	-0.6604604528	2.3011451158	-1.4210341057
C	1.5395439440	1.1787089163	-0.2529581289
C	2.5315076187	0.0236605955	-0.4979410502
C	2.3058343050	-1.1169227922	0.4987939742
C	0.8754603151	-1.6234148968	0.3517963278
C	1.8737894644	1.8836197743	1.0850077637
H	-2.1922046855	0.5602787712	-1.8719394663

H	-3.0854357334	1.8095543388	-0.9342069039
H	-1.8344681246	-1.8586234609	0.9989105124
H	-1.5231018915	-1.6023775015	-0.7577381957
H	-0.5828269021	4.4006200555	-0.5809493062
H	-0.8185343452	6.0340773147	1.1895619879
H	-1.6584068691	1.2593771688	2.1651298601
H	-2.4985873962	1.6340283796	5.7599604592
H	-1.3386173767	0.9557134371	4.5583162741
H	-3.0938456841	1.1743903719	4.1219302228
H	-1.5093983040	7.2994965522	4.6539920176
H	-2.0277409223	7.2924596625	2.9275075020
H	-0.2729543840	7.0699419935	3.3624570404
H	1.7163920641	1.9206287451	-1.0629717112
H	3.5793997705	0.3887201538	-0.4295851371
H	2.3799137493	-0.3667546163	-1.5289402879
H	2.4893818527	-0.7816522887	1.5398848551
H	3.0152835382	-1.9454254958	0.2860545381
H	0.6914273934	-2.4478886477	1.0747960254
H	0.7755148040	-2.0390854325	-0.6764489968
H	1.6807450491	1.2446331590	1.9680345697
H	1.3243708697	2.8267776893	1.2173727676
H	2.9461138364	2.1731723584	1.0934616423

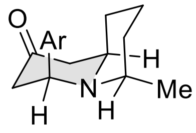

(x) RRS

(x) RRS – Dimeth-6Mem

C	-1.7780058878	0.5224092494	0.5194783528
C	-0.6050610988	1.0849684540	-0.3160477532
N	0.6221705199	0.2291058647	-0.2353326139
C	0.5268418158	-0.9972328844	0.6149788138

C	-0.7423868548	-1.8155901425	0.2716579380
C	-1.9541855005	-0.9319917796	0.2344822751
O	-3.0444439120	-1.3819688210	-0.0820292776
C	-0.9650317599	1.3202774926	-1.7777726472
C	-0.9987071382	2.6327929376	-2.2785688718
C	-1.3194614228	2.8812408371	-3.6131676612
C	-1.6142848848	1.8184275832	-4.4873322271
C	-1.5815441639	0.5006473213	-4.0056567174
C	-1.2555912664	0.2604130933	-2.6547227523
O	-1.9307615950	2.0207928027	-5.7940679105
O	-1.8677943840	-0.5079319896	-4.8719374141
C	-1.8866806966	-1.8546381447	-4.5725388472
C	-2.0052285422	3.2429041906	-6.4296339026
H	0.4694496607	-0.6960896209	1.6836510739
H	-0.4250061530	2.0836254137	0.1286198503
C	1.8759492834	1.0075411030	-0.0277913634
C	3.0954363253	0.0963791161	-0.2729692666
C	3.0597679986	-1.1345544591	0.6393981079
C	1.7605934223	-1.9006256067	0.3989795262
C	1.9630853541	1.6859458299	1.3640717122
H	-2.7131482868	1.1054133935	0.3707257443
H	-1.5014751089	0.6072956374	1.5930598330
H	-0.8797419737	-2.6384931987	1.0058176530
H	-0.6311364793	-2.2668906283	-0.7369394339
H	-0.7764818028	3.4751520909	-1.6351701734
H	-1.3345837278	3.9073458543	-3.9549742809
H	-1.2243005797	-0.7504021695	-2.2936513925
H	-2.1555441739	-2.4247194472	-5.4852376875
H	-2.6458249829	-2.0632028748	-3.7886164535
H	-0.8830822862	-2.1866267816	-4.2308882329
H	-2.2850432946	3.0826170838	-7.4909468767

H	-1.0191301616	3.7534063375	-6.3969259148
H	-2.7808390651	3.8781118847	-5.9511519994
H	1.9261532807	1.8198787289	-0.7872533835
H	4.0407095383	0.6608941231	-0.1198791300
H	3.0745512570	-0.2468637949	-1.3312633300
H	3.1341753578	-0.8384369285	1.7057678097
H	3.9267807025	-1.7925198521	0.4153962516
H	1.7169810052	-2.7853028028	1.0708206458
H	1.7659774982	-2.2637968756	-0.6533903381
H	1.9218862940	0.9618902412	2.1995463843
H	1.1540371662	2.4224593998	1.5264112138
H	2.9196549820	2.2447910870	1.4433480204

13. References

[1] L. Bettoni, S. Gaillard, J.-L. Renaud, *Chemical Communications,* **2020**, *56*, 12909-12912.
[2] T. K. Hutton, K. W. Muir, D. J. Procter, *Organic Letters,* **2003**, *5*, 4811-4814.
[3] J. Ryan, M. Šiaučiulis, A. Gomm, B. Maciá, E. O'Reilly, V. Caprio, *Journal of the American Chemical Society,* **2016**, *138*, 15798-15800.
[4] H. Kusama, Y. Onizawa, N. Iwasawa, *Journal of the American Chemical Society,* **2006**, *128*, 16500-16501.
[5] M. Adeva, H. Sahagún, E. Caballero, R. Peláez-Lamamié de Clairac, M. Medarde, F. Tomé, *The Journal of Organic Chemistry,* **2000**, *65*, 3387-3394.
[6] B. Nie, W. Wu, Q. Ren, Z. Wang, J. Zhang, Y. Zhang, H. Jiang, *Organic Letters,* **2020**, *22*, 7786-7790.
[7] T. Kawasaki, Y. Nonaka, M. Akahane, N. Maeda, M. Sakamoto, *Journal of the Chemical Society, Perkin Transactions,* **1993**, 1777-1781.
[8] R. Grigg, J. Markandu, T. Perrior, S. Surendrakumar, W. J. Warnock, *Tetrahedron,* **1992**, *48*, 6929-6952.
[9] Y. Xing, W. Cen, J. Lan, Y. Li, Y. Li, *Journal of the Chinese Chemical Society,* **1999**, *46*, 595-600.
[10] A. P. Ramirez, A. M. Thomas, K. Woerpel, *Organic Letters,* **2009**, *11*, 507-510.
[11] F. Orsini, E. Di Teodoro, M. Ferrari, *Synthesis,* **2002**, *2002*, 1683-1688.
[12] M. A. M. Mathieu P. Lalonde, Naomi S. Rajapaksa, Eric N. Jacobsen, *Journal of the American Chemical Society,* **2013**, *135*, 1891−1894.
[13] Frisch, M.J., Trucks, G.W., et al., **2016**, Gaussian 16 Revision A.03.
